# Simulation study to evaluate when Plasmode simulation is superior to parametric simulation in comparing classification methods on high-dimensional data

**DOI:** 10.1371/journal.pone.0322887

**Published:** 2025-06-02

**Authors:** Marieke Stolte, Nicholas Schreck, Alla Slynko, Maral Saadati, Axel Benner, Jörg Rahnenführer, Andrea Bommert, for the topic group “High-dimensional data” (TG9) of the STRATOS Initiative

**Affiliations:** 1 Department of Statistics, TU Dortmund University, Dortmund, North Rhine-Westphalia, Germany; 2 Division of Biostatistics, German Cancer Research Center, Heidelberg, Baden-Wuerttemberg, Germany; 3 Faculty of Liberal Arts and Sciences, Technical University of Applied Sciences Augsburg, Augsburg, Bavaria, Germany; 4 Department of Statistics and Actuarial Science, University of Waterloo, Waterloo, Ontario, Canada; National Chengchi University, TAIWAN

## Abstract

Simulation studies, especially neutral comparison studies, are crucial for evaluating and comparing statistical methods as they investigate whether methods work as intended and can guide an appropriate method choice. Typically, the term simulation refers to parametric simulation, i.e. computer experiments using pseudo-random numbers. For these, the full data-generating process (DGP) and outcome-generating model (OGM) are known within the simulation. However, the specification of realistic DGPs might be difficult in practice leading to oversimplified assumptions. The problem is more severe for higher-dimensional data as the number of parameters to specify typically increases with the number of variables in the data. Plasmode simulation, which is a combination of resampling covariates from a real-life dataset from the DGP of interest together with a specified OGM is often claimed to solve this problem since no explicit specification of the DGP is necessary. However, this claim is not well supported by empirical results. Here, parametric and Plasmode simulations are compared in the context of a method comparison study for binary classification methods. We focus on studies conducted with some specific data type or application in mind whose true, unknown data-generating mechanism is mimicked. The performance of Plasmode and parametric comparison studies for estimating classifier performance is compared as well as their ability to reproduce the true method ranking. The influence of misspecifications of the DGP on the results of parametric simulation and of misspecifications of the OGM on the results of parametric and Plasmode simulation are investigated. Moreover, different resampling strategies are compared for Plasmode comparison studies. The study finds that misspecifications of the DGP and OGM negatively influence the ability of the comparison studies to estimate the classification performances and method rankings. The best choice of the resampling strategy in Plasmode simulation depends on the concrete scenario.

## Introduction

Simulation studies are a crucial tool in evaluating and comparing the performance of statistical methods. They can provide useful insights into the behavior of the methods in certain situations. Neutral method comparison studies, i.e. comparison studies evaluating existing methods outside the context of proposing a new method, are particularly important to ensure that methods work as expected and for making an informed method choice for an analysis task at hand [1,2].

Most commonly, the term simulation is used to refer to parametric simulation. That is, computer experiments based solely on pseudo-random data generation according to data-generating processes (DGP) and outcome-generating models (OGM) specified by the researchers conducting the simulation study. Often, covariate data is generated from a specified distribution using a pseudo-random number generator. Then, the specified OGM is applied to the generated covariate data to generate observations of a target variable. This step might again include some pseudo-random number generation, e.g. to produce some noise in the target variable. This procedure has the advantage of full control over the data generation and full knowledge of all parameters within the simulation, which enables the calculation of performance measures that rely on knowledge of the true parameters like the bias of an estimator [3,4]. However, the specifications of the DGP and the OGM might be oversimplified and therefore unrealistic as the specification of complicated DGPs and OGMs is often hard in practice, especially for large numbers of variables. For example, the specification of a complex correlation structure becomes tedious for large numbers of variables [5].

Plasmode simulations [6] are often claimed as a solution to the problem of unrealistic assumptions made in parametric simulations. For statistical Plasmode simulation, the covariate data is generated by resampling from a real-world dataset that is drawn from the true DGP of interest. Therefore, no explicit DGP specification is needed. Moreover, the resampling from the real-world dataset is expected to accurately reflect the true DGP, assuming that the dataset is representative and possibly additional assumptions on the resampling scheme [5]. As for parametric simulation, the target observations are generated using an OGM specified by the researchers. Therefore, some truth is still known in the data generation and all performance measures, such as the bias, that need knowledge of parameters in the OGM can still be calculated. Thus, Plasmode simulation seems like a good alternative to parametric simulation for investigating complex DGPs while still being able to evaluate the performance of statistical methods of interest [5].

However, [5] noted that this often-made claim of Plasmode simulation producing more realistic data is not well supported by any empirical results. Moreover, they point out potential pitfalls when conducting Plasmode simulation studies. For example, they mention the importance of choosing an appropriate dataset to resample from, the difficulties of small sample sizes for resampling, and the choice of the resampling strategy itself. In addition, they highlight the importance of the choice of an appropriate OGM and question, for example, the practice of nullifying existing associations between covariates and the target variable. Therefore, a comparison of parametric and Plasmode simulation is required to find out in which situations Plasmode simulation is actually preferable.

As a first step to close this gap, [7] empirically compared parametric and Plasmode simulations for the example of estimating the mean squared error of the least squares estimator in linear regression. They found that, as expected, parametric simulation performs best if the DGP and OGM are specified correctly, but it quickly gets worse when some aspects of the DGP or OGM are misspecified. The performance of the Plasmode simulation also deteriorated in case of misspecifications of the OGM. Moreover, the performance of the simulations, especially for Plasmode, got worse when increasing the number of variables or decreasing the number of observations in the generated datasets. Regarding the resampling step in Plasmode simulations, often subsampling with low resampling proportions outperformed the other options in the comparison, but this required a larger dataset to resample from. However, that study was limited to only one specific example case of a method evaluation study.

Here, we want to expand on this by comparing parametric and Plasmode simulation in the context of method comparison studies, using the example of comparing multiple binary classification methods. The comparison of multiple methods is more complex as not only the performance of each method but also their ranking with respect to the performance is of interest. We focus on the case where researchers designing a simulation study have a certain type of data or a certain application in mind as in this case, Plasmode is a reasonable alternative to parametric simulation. Therefore, we assume there is some true but typically unknown data-generating mechanism that researchers try to mimic through their simulation. Here, we compare how well the true classification performance and method ranking can be recovered for parametric simulation studies and for Plasmode simulation studies with different resampling strategies. Under the true scenario, it is expected that parametric simulation performs best. However, the truth is typically unknown to researchers conducting simulation studies and instead, they have to make assumptions trying to approximate this truth. These assumptions are likely to deviate from the truth. Therefore, we analyze how performance estimation and method ranking are affected by misspecifications of the DGP (for parametric comparison studies) and of the OGM (for parametric and Plasmode comparison studies). In comparison to the previous study, we use a higher-dimensional setup and additional deviations. Note that we do not aim to perform a neutral comparison study of classification methods but to compare how well such a study would perform using different simulation approaches and assumptions within the comparison study.

The remaining article is structured as follows. In the Simulation setup section, the setup of the simulation study is explained. In the Results section, the results for the comparison of parametric and Plasmode, and the influence of misspecifications of the DGP and OGM are described. First, results regarding the estimation of the classification performance measures are shown. Then, results regarding the method ranking are presented. Last, an overall comparison based on the proportion of acceptable simulation results is performed. Precisely, this is the proportion of simulation results whose relative errors in estimating the classification performance fall into the 2.5% to 97.5%-quantile interval of the relative errors for parametric simulation using the true DGP and OGM. In the Discussion section, the results are summarized and discussed.

## Simulation setup

**Fig 1:**
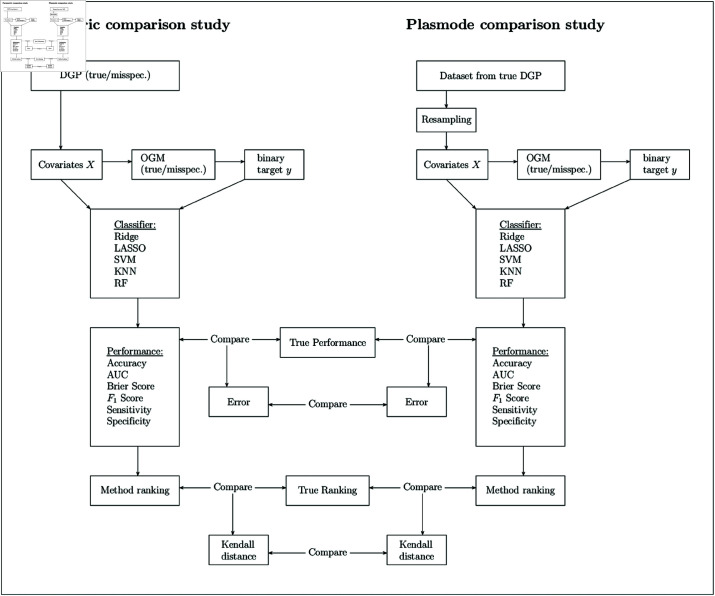
Schematic process of the simulation study.

In the following, we describe the simulation setup following the ADEMP (Aims, Data-generating mechanism, Estimands, Methods, Performance measures) structure [3]. The overall procedure for the simulation is visualized in Fig 1.

### Aims

The aims of our simulation study are:

1. Compare how well parametric and Plasmode simulation can estimate the performance and method ranking for several classification methods.
2. Find out how deviations from the true DGP and OGM affect parametric simulation in terms of estimating the performance and ranking classification methods.
3. Find out how deviations from the true OGM and different resampling strategies affect Plasmode simulation in terms of estimating the performance and ranking of classification methods.
4. Find out how the dimension of the datasets, i.e. the number of covariates, affects 1. to 3.

Note that the study does not aim to perform a neutral method comparison of classification methods. Instead, it is of interest how well such a method comparison study can recover the true method performances under certain simulation approaches and possibly misspecified assumptions in the comparison study.

### Data-generating mechanism

Since we want to compare how well comparison studies can recover the true method performances, we first have to define a DGP and OGM that are considered the truth for our study. Additionally, we have to specify the assumptions on the DGP and OGM within the comparison study. These do not have to coincide with the truth as the truth is typically unknown to the researchers performing such comparison studies. However, the assumptions on the DGP and OGM made within the comparison studies are chosen fairly close to the truth based on the assumption that researchers conducting the study would try to mimic the truth as well as possible.

#### True scenarios.

The true scenarios consist of a true data-generating process (DGP) and a true outcome-generating model (OGM). We must have full knowledge of both. At the same time, in practice, the true DGP and OGM are typically complicated, which we try to reflect here as well. We fix the sample sizes for all generated datasets at *n* = 100. For larger *n* the classification problem becomes easier. For smaller *n* the training datasets become very small. The number of variables for each sample is varied as p=2,10,50,150. This means that we have one true scenario for each *p*. However, we try to keep the true scenarios for different *p*s as comparable as possible. Larger values of *p* quickly result in infeasibly long runtimes of the classification models. Smaller *n* leads to deficient true classification performances of the classifiers, which makes the comparison of different simulation strategies pointless. In this case, often the model is random guessing under the true scenario, and the model in the comparison study is also random guessing. Consequently, the simulated performances are close to the classification performances under the true scenario by chance.

**True DGPs.** We specify the distribution of 150 variables. For the other values of *p*, subsets of the marginal distributions will be chosen as described below and the correlation matrix is reduced to the corresponding entries. This ensures that the DGPs for different *p* are comparable.

Here, for the true DGP, the marginal distributions and the correlation structure of the 150 variables have to be specified. For the marginal distributions, different distribution families are chosen including normal distribution, log-normal distribution representing a skewed distribution, Gaussian mixture distributions representing bimodal distributions, and a contamination model for outliers, respectively. Table 1 gives an overview of the numbers of variables per distribution class for each value of *p*.

**Table 1:** Number of variables generated from each distribution class per number of variables *p.*

*p*	Normal	Log-normal	Bimodal	Outlier
150	50	50	25	25
50	15	15	10	10
10	3	3	2	2
2	1	0	1	0

For *p* = 150, for normal distributions, we generate 50 variables for which the means and variances are randomly sampled such that the expected parameter for the mean is zero and the expected parameter for the variance is one (see Section A of S1 Appendix). For log-normal distributions, 50 variables are generated and the parameters μ and σ are randomly sampled in the same way as for the normal variables. For Gaussian mixture distributions, also 50 variables are generated. Half of these are generated from bimodal distributions and the other half is generated from a contamination model. The parameters are sampled as follows. For the first component of these variables, parameters are drawn such that on average standard normal parameters are achieved. For the bimodal distributions, the second component has an expected μ of 4. For the outlier distributions, the second component has an expected variance of 10. For details see Section A of S1 Appendix. The distribution of the first few variables of each type is visualized in Fig A.2 in Section A of S1 Appendix. Drawing the parameters produces more diverse marginal distributions than specifying the values by hand.

The correlation matrix is also generated randomly. Fig A.3 in Section A of S1 Appendix shows the distribution of pairwise correlations. For details on the random generation, see Section A of S1 Appendix. All marginal distributions with generated parameters can be found in S3 Appendix. The full correlation matrix is given in S4 Appendix.

The parameters for all distributions and the correlations are drawn only once and set as the true parameters for these true distributions for the whole simulation.

For *p* = 50, we select the first 15 of the normal distributions, the first 15 of the log-normal distributions, and the first 10 for each of the bimodal and outlier Gaussian mixture distributions and the corresponding entries from the true correlation matrix.

For *p* = 10, we select the first three of the normal distributions, the first three of the log-normal distributions, and the first two for each of the bimodal and outlier Gaussian mixture distributions and the corresponding entries from the true correlation matrix.

For *p* = 2, we select the first of the normal distributions, the first of the bimodal Gaussian mixture distributions, and the corresponding entries from the true correlation matrix.

Note that in these cases we are not selecting parts from the same dataset with *p* = 150 variables but instead, we are drawing data from the respective subsets of the 150 distributions.

Since it helps with constructing the deviation scenarios, we rescale all variables in the generated datasets from the true DGP to [0,1] using a min-max transformation


$$x_{i,\text{rescaled}}=\frac{x_{i}-x_{\min}}{x_{\max}-x_{\min}},$$

where *x*_*i*_ denotes the *i*th observation of variable *x* and $x_{\min}$ and $x_{\max}$ denote the minimum and maximum of *x*, respectively.

**True OGMs.** We use a logistic model as true OGM since it allows us to control the true separation of the two classes most efficiently. Note that this choice can give an unfair advantage to linear classification methods like Ridge and LASSO logistic regression. Since we are not inherently interested in the method comparison of the classification methods but in how well the simulation studies reconstruct the true comparison, we can give up the fairness in comparing classification methods to some extent. It might even be advantageous here to have a slightly unfair classification method comparison since then the differences between the classifiers are expected to be more distinct and therefore the method order is clearer and easier to reconstruct in the simulations. If the true order is ambiguous since all methods perform equally well, it is expected that the simulation studies cannot reconstruct this order well. For a discussion of an alternative approach and its disadvantages that made us not consider it and instead led to our choice, see Section B in S1 Appendix.The coefficients for the logistic model for *p* = 150 are chosen as follows. The 100 coefficients for the normal, log-normal, and outlier variables are drawn at random either from a *U*(–8, –3) or from a *U*(3, 8) distribution. The remaining 50 coefficients for the bimodal variables are drawn at random either from a *U*(–15, –10) or from a *U*(10, 15) distribution. The choice of larger absolute coefficients for the bimodal distributions ensures a clear separation of the data into the two classes that is necessary to achieve reasonable performances of the classification methods for larger *p*. The intercept is set to adjust the predicted probabilities such that the target variable is nearly balanced. Own analyses showed that extreme unbalance results in many generated datasets with either no generated zero responses or no generated responses of one, which makes the classification unnecessary. Note that the coefficients are seemingly very large but the data is rescaled to [0,1] before applying the OGM. Therefore, odds ratios (OR) for a variable increase of 0.1 are more realistic than the typical increase of 1. The ORs for an increase of 0.1 and the positive coefficients of normal, log-normal, and outlier variables are between 1.35 and 2.23, and for the coefficients of bimodal variables between 2.72 and 4.48. The resulting distribution of predicted probabilities (Fig D.1 in Section D of S1 Appendix) shows a clear separation between the two classes and is approximately symmetric, resulting in an approximately balanced binary target variable. The exact coefficients can be retrieved from the R code available on Zenodo (https://doi.org/10.5281/zenodo.13707473). Fig C.1 in Section C of S1 Appendix shows the distribution of coefficients.

For *p* < 150, the coefficients corresponding to the respective variables chosen from the true DGP are used and modified slightly if necessary to achieve good separation. For details see Section D of S1 Appendix.

As with the true DGP, the coefficients for *p* = 150 are drawn exactly once in the beginning and then kept constant during the whole simulation process.

The *i*th target observation for a given simulated covariate dataset is generated by drawing from a Bernoulli distribution with the success probability set to the probability predicted by the true OGM as


$${\hat{\pi }}_{i}=\frac{1}{1+\exp(-x_{i}^{T}\beta )},$$

where $x_{i}^{T}$ is the *i*th row of the simulated dataset supplemented by a leading one for the intercept, i=1,…,100, and β is the coefficient vector generated as described above.

Note that the true OGMs are constructed such that each feature influences the outcome.

#### Deviations.

In the following, it is described how the DGP and the OGM are misspecified within the comparison studies. In addition to the misspecifications described below, the true DGP and OGM are always used once for a parametric and for a Plasmode comparison study, respectively. Table 2 gives an overview of all applied misspecifications.

**Table 2:** Misspecifications of the DGP in parametric and of the OGM in parametric and Plasmode simulation.

Type of misspecification		Values
DGP	Shift	δ∈{−0.5,−0.25,−0.125,0.125,0.25,0.5}
	Scale	s∈{0.25,0.5,0.75,1.33,2,4}
	Correlation	ρ∈{−0.2,−0.1,0,0.1,0.2}
	Distribution	N(\boldmath0,I)
OGM	Scaled	c∈{0.5,2,0}

#### Misspecifications of the DGP.

The DGP in parametric simulations can be misspecified by changing some characteristics of the distribution. Shift, scale, correlation, and the whole distribution are misspecified as follows one at a time. The concrete parameter values are given in Table 2. Note that the generated data from the true DGP is first rescaled to [0,1] and then for shift the value of δ is added to all observations and for scale the data is multiplied by *s*.

The parameter settings were chosen as follows. As all data is scaled to [0,1], a shift of ±0.5 is already extreme. Too extreme values of the shift might result in the generation of extremely imbalanced classes which in the extreme case makes the application of the classification models or the performance estimation impossible. Therefore, the extreme shift values were considered together with less extreme values. For scale, the most extreme values of *s* = 0.25 and *s* = 4 were chosen such that still reasonable proportions of zeroes and ones are generated. As especially *s* = 0.25 turned out as too extreme in certain scenarios, the less extreme values of 3/4 and 4/3 were added. For misspecifying the correlation, all pairwise correlations are fixed as ρ. Larger absolute correlations are infeasible for many pairs of marginal distributions, see the discussion in Section A of S1 Appendix.

Lastly, the distribution is completely misspecified as standard normal. This case is included because researchers with no prior knowledge about the true DGP often use standard normal data in their comparison study by default.

**Misspecification of the OGM.** A very general approach to modify classification models applicable to all models that output predicted probabilities is described in [8]. The predicted probabilities $\hat{\pi }$ of the model are transformed into log-odds $\log(\hat{\pi }/(1\;-\;\hat{\pi }))$. These log-odds are multiplied by a constant *c* to get stronger or weaker associations. The new log-odds are then transformed back to the probability scale


$${\hat{\pi }}_{\text{new}}=\frac{1}{1+\exp(c\cdot \log(\hat{\pi }/(1-\hat{\pi })))}.$$

These new probabilities are used to generate observations of the target variable. We adopt this approach here with a factor of


c∈{0.5,2}

to get models with weaker and stronger associations, respectively. Values of |c|>1 correspond to stronger associations and lead to better-separated classes while values of |c|<1 lead to weaker associations and less separated classes in the simulated responses. For the special case of the logistic model as the true OGM, this is equivalent to multiplying each coefficient by *c*. Note that for *c* < 0 it holds:


$$c\log\left(\frac{\hat{\pi }}{1-\hat{\pi }}\right)=\log\left({\left(\frac{\hat{\pi }}{1-\hat{\pi }}\right)}^{c}\right)=\log\left({\left(\frac{1-\hat{\pi }}{\hat{\pi }}\right)}^{\left|c\right|}\right)=|c|\log\left(\frac{1-\hat{\pi }}{\hat{\pi }}\right).$$

Therefore, using negative factors is equivalent to changing the roles of zeroes and ones and using the absolute value of the factor. Changing the roles of zeroes and ones does not affect the classification performance measured by accuracy, AUC, and the Brier score but changes the roles of sensitivity and specificity (see Section on performance measures). For the *F*_1_-score, it is unclear how the performance changes. As it is clear how the use of negative factors affects most of the performance measures used, only positive values are used. In addition, we use a logistic model with constant coefficients of 0, i.e. no effect of the covariates on the response, as this might be done in many simulations to illustrate a null situation. This is included as [5] pointed out that nullifying true existing effects is potentially problematic. Overall we misspecify the OGM once by scaling by 0.5 to achieve weaker associations, once by scaling by 2 to achieve stronger associations, and once by setting all coefficients to 0 as discussed above.

### Targets

The targets of the study are the classification performance on the simulated data and performance rankings for comparing classification methods obtained by parametric and Plasmode simulation.

### Methods

In the following, parametric and Plasmode simulation and the classification methods for the method comparison studies are briefly explained.

#### Parametric simulation.

Parametric simulation refers to simulations where the whole data consists of pseudo-random numbers drawn from a data-generating process (DGP) and an outcome-generating model (OGM) specified by the researcher. Therefore, both the DGP and OGM are fully known. The choice of these can be hard in practice. Researchers might try to set up their parametric simulation to be as close as possible to certain data of interest. Alternatively, researchers might want to cover as many situations as possible including extreme scenarios. The first case is the one where Plasmode simulations might be a reasonable alternative. In the latter case, parametric simulation would be the obvious choice as it allows specification of all aspects of the DGP and OGM. Therefore, we focus on the first case here. When the DGP and OGM are specified, a large number of covariate datasets can be generated using a pseudo-random number generator to draw observations from the DGP. Then, the OGM is applied to this generated covariate data to generate corresponding observations of the target variable. This process mimics repeatedly drawing samples from a large population with the specified DGP and OGM. For method comparison, the methods are then applied to the generated datasets, and their performance is evaluated with regard to performance metrics of interest. Since all aspects of the true DGP and OGM are known, performance metrics depending on these (e.g. bias) can be assessed. The results can help to understand how the methods perform for datasets similar to the chosen DGPs and OGMs and which method to prefer in which situations. This is of great use for an adequate method choice in practice [1,4]. For more details on how to design, perform, analyze, and report parametric simulation studies, refer to [3]. For method comparison studies, see also [1].

Here, we perform the parametric simulation studies as follows. In each of the 100 iterations for a scenario consisting of a combination of *p*, the choice of the DGP, and the choice of the OGM, we draw 100 observations from the chosen DGP. Then, the chosen OGM is applied to this generated covariate data to generate observations of the binary target variable. Subsequently, all classification methods are applied using 5-fold nested cross-validation for hyper-parameter tuning and performance estimation. Last, the methods are ranked according to their performance with regard to each performance measure.

#### Plasmode simulation.

The main difference between Plasmode simulation and parametric simulation is the generation of the covariate datasets. In Plasmode simulation studies, instead of specifying the DGP like in the parametric case, data is resampled from a real-life dataset from the true DGP of interest. Therefore, no explicit assumptions on the DGP are made. However, it is required to have a representative real-life dataset from the true DGP at hand. The OGM is then applied to the resampled covariate datasets and the method comparison is performed analogously to the parametric simulation. Plasmode can be seen as a semi-parametric approach as it combines the resampling from a real-life dataset in non-parametric simulation with the use of a specified OGM in parametric simulation. This has the advantage that some control over the data generation is given and some aspect of the truth within the simulation is known while at the same time, the problem of unrealistic specifications of the DGP in parametric simulation is avoided [5]. When using only real data, certain quantities depending on unknown parameters (e.g. bias) cannot be assessed [4]. For a more detailed discussion of the advantages and disadvantages of Plasmode simulations as well as guidance on how to perform them refer to [5].

There are multiple options for the resampling step. Here we use all resampling techniques that are commonly used according to [5]:

- *m* out of *n* Bootstrap [9–12] with resampling proportions 0.632 and 1, i.e. drawing with replacement m≤n observations of the original dataset.
- Subsampling with resampling proportions 0.632 and 1, i.e. drawing without replacement *m* < *n* observations of the original dataset or using the whole dataset.

These values for the resampling proportions were chosen for comparability with the previous study [7] where the values of 0.632 and 1 were used as they were identified as relevant special cases from the literature. Additionally, smaller resampling proportions like 0.1 were previously used and showed good performance. Here, nested 5-fold cross-validation will be applied to the datasets later on (see Subsection Performance measures). For *n* = 100, the training datasets have size 100·4/5·4/5=64 in the inner cross-validation loop. If we apply subsampling or Bootstrapping this number of training datapoints reduces accordingly. For a resampling proportion of 0.632, there are about 40 training points left which is already few. Therefore no smaller resampling proportions are used. Another solution would be to increase the number of folds in the cross-validation, but the runtime increases roughly quadratically in the number of folds. Therefore, the number of folds is kept low and the resampling proportions higher.

For each specific scenario, consisting of the number of variables *p*, a chosen resampling strategy, and a chosen OGM, a dataset of size 100 is generated from the true DGP. This dataset is then used to resample from it, for the 100 iterations of the Plasmode simulation. After resampling from this dataset from the true DGP, the next steps are the same as for the parametric simulation, applying the OGM and analyzing the generated data.

#### Classification methods.

Within our parametric or Plasmode method comparison studies, we compare several methods for binary classification including

- Ridge logistic regression [13],
- LASSO logistic regression [14],
- Support vector machine (SVM) [15],
- *k*-nearest neighbors (KNN) [16,17], and
- random forest (RF) [18].

As we are not primarily interested in the method comparison itself we do not include boosting or neural nets due to their high runtimes and sensitivity to tuning. We concentrate on commonly used classification methods for the low to high-dimensional regime that we investigate here. For even higher-dimensional data, specialized classification methods might be needed [19,20]. We use 5-fold nested, stratified cross-validation (see Subsection) and random search with a budget of 100 evaluations for hyperparameter tuning of each method. We tune with respect to classification accuracy. The low budget is chosen as we are not primarily interested in the method comparison itself and runtime is an issue in this study. The hyperparameter spaces are chosen as suggested in [21].

### Performance measures

For judging the performance of a binary classification method, typically its predicted outcome values are compared to the true outcome values. These can be summarized in a confusion matrix counting the numbers of observations for all possible combinations of true and predicted outcomes (see Table 3).

**Table 3:** Confusion matrix for binary classification methods. *y*, true outcome; $\hat{y}$, predicted outcome; TN, number of true negatives; FN, number of false negatives; FP, number of false positives; TP, number of true positives; *N*, the number of observations.

	y=0	y=1	∑
$\hat{y}=0$	TN	FN	TN + FN
$\hat{y}=1$	FP	TP	FP + TP
∑	TN + FP	FN + TP	*N*

For the method comparison within each simulated simulation study,

- Accuracy $=\frac{\text{TN}+\text{TP}}{N}$,
- *F*_1_-Score $=\frac{2\text{TP}}{2\text{TP}+\text{FP}+\text{FN}}$,
- Sensitivity $=\frac{\text{TP}}{\text{TP}+\text{FN}}$,
- Specificity $=\frac{\text{TN}}{\text{FP}+\text{TN}}$
- AUC (Area under the Receiver Operating Curve that is the diagram of Sensitivity against 1–Specificity for different cutoff values for the predicted probabilities corresponding to a prediction of a 1), and
- Brier score $=\frac{1}{N}\sum_{i=1}^{N}({\hat{\pi }}_{i}-y_{i}{)}^{2}$, where ${\hat{\pi }}_{i}$ is the predicted probability for a 1 for the *i*th observation and $y_{i}\in \{0,1\}$ the corresponding true outcome value,

are used to judge the performance of the classification methods. Subsequently, the methods are ranked according to each measure. All measures return values in [0,1]. For all except the Brier score, high values indicate good performance. For the Brier score, low values indicate good performance [22,23]. 5-fold nested cross-validation [24] is applied for performance estimation and hyperparameter tuning. Note that the performance measures are chosen because they are commonly used performance measures for binary classification methods rather than recommendations. For instance, only the Brier score is a proper measure, AUC is semi-proper and all other measures are improper measures.

We calculate performance measures, based on scoring rules to assess the quality of probabilistic predictions by assigning a numerical score to compare predictions and the occurring event. A scoring rule is proper if the best predictor is the true probability of the event. A strictly proper scoring rule such as the Brier score guarantees that the best value is only achieved when we get as close as possible to the true probability. A semi-proper measure not only does not guarantee that the best performance is achieved by a predictor whose predictions are closest to the true probabilities, but it is also possible to improve the values of the measure by moving the predicted probabilities away from their true values. An improper scoring rule, such as ’Accuracy’, does not predict probabilities as close as possible to the true probabilities [25].

If a method fails and an error is thrown, a fallback learner that always predicts the majority class is used instead to calculate the performance. Using a fallback learner is recommended over excluding the iterations with method failure or penalizing method failure by imputing the worst possible score [26].

For the parametric and Plasmode simulation, 100 datasets are generated per scenario on which the method comparison is performed. This number is mainly motivated by runtime. If only ones or only zeros are generated in an iteration, the whole data including the covariates is redrawn up to 50 times. It might still happen that during cross-validation some of the folds have only ones or only zeroes as response values. Then, the sensitivity or specificity cannot be calculated and consequently also the AUC cannot be calculated for this fold. In this case, the values of the measure in the remaining folds are averaged and the fold with only ones or only zeroes as response values is left out (for the affected measures only). In the case of sensitivity and specificity, this procedure gives similar results to calculating the measure on all predicted responses across the folds as the proportions of ones and zeroes are similar in all folds since we use stratified cross-validation. For the AUC, the results when first pooling the predictions over the folds could differ notably if the classifiers in the different folds are calibrated differently. Therefore, pooling would not be a good idea and we choose the approach of averaging over the remaining folds. If there are no true or predicted ones for a certain fold, the *F*_1_-score cannot be calculated. In case of no true ones, the same approach as for sensitivity and AUC is chosen. In case of no predicted ones, a value of zero is assigned as the *F*_1_-score for that fold which corresponds to the worst possible value. If there are ones, but the classifier does not predict any, then its performance regarding predicting ones is as bad as possible.

To judge the performance of the simulation studies themselves we calculated the differences between the estimated performance values and their true values for each measure. The true performances and rankings are approximated using datasets drawn from the true DGP and responses generated by the true OGM and benchmarking all five classification methods with regard to all performance measures on these simulated datasets, as described before. This is done 500 times for the true model for each value of *p*. The mean performance of each classification method is calculated as its true performance for each measure. The method ranking based on these mean performances is used as the true ranking. Ranks are always assigned such that lower ranks indicate better performance regardless of whether high or low values of the corresponding performance measure indicate good performance. Moreover, the Kendall distance [27] of the simulated and the true ranking according to each measure is calculated. It is a standard metric for comparing permutations [28]. The Kendall distance is defined as the number of swaps of neighboring values required to transform the simulated ranking into the true one. Kendall distance values are normalized to [0,1] where 0 corresponds to equal rankings (best possible value) and 1 corresponds to reversed rankings (worst possible value). Ties in the method rankings are broken at random as average ranks are not permitted for the calculation of Kendall distance. Since the true ranks are in {1,2,3,4,5} for example the ranking 1, 2.5, 4, 2.5, 5 cannot be transformed to the true ranks via permuting adjacent numbers in the ranking. Ideally, the estimated method ranking should be similar to the true ranking as method rankings established by simulation studies should be used as guidance for choosing a suitable method in practice [1]. Therefore, a wrong method ranking in a simulation study can result in non-optimal method choices in practice.

Even if the classification performance measure values for the classifiers are estimated precisely, still the method ranking might differ from the true ranking as often already small differences in the classification performance can change the rank of a method. Conversely, the estimation of the method ranking can still be good if all estimates of the classification performance measures are biased in the same direction and by roughly the same amount. A good simulation study should recover the true method ranking without under- or overestimating the true classification performances. Therefore, both the errors in estimating the classification performance as well as the Kendall distances of the method rankings are taken into account.

To summarize the results of the comparison studies, the proportions of acceptable simulation results are calculated as follows. First, relative errors of one minus the respective measure with respect to one minus the true measure are calculated for each iteration as


$${\text{Relative Error}}_{i}=\frac{\left(1-{\hat{M}}_{i})-(1-M\right)}{1-M},$$

where *M* denotes the true measure value and ${\hat{M}}_{i}$ the simulated measure value in the *i*th iteration. This weighs errors for high true performance values higher than for moderate performance which is how we would judge the performance intuitively. For the Brier score, where low values correspond to good performance, the usual relative errors


$${\text{Relative Error}}_{\text{Brier Score},i}=\frac{{\text{Simulated Brier Score}}_{i}-\text{True Brier Score}}{\text{True Brier Score}},$$

are calculated. Then, the proportion of “acceptable” simulation results is calculated per simulation type, measure, classifier, and scenario. For a simulation result to be called acceptable, here, its relative error must lie within the 2.5% and 97.5% quantile interval of the relative errors for the parametric simulation for the true scenario for the corresponding measure, classifier, and *p*. Therefore, for the true scenario and parametric simulation, the proportion of acceptable iterations is 95% by design. The relative errors for the parametric simulation for the true scenario for a measure and classifier can be seen as the best results possible in a comparison study. The proportions are compared across the other simulation types and scenarios for each classifier, measure, and number of variables *p*. High proportions of acceptable estimates mean that comparison studies with the respective assumptions will perform comparably well as comparison studies under the true scenario which yield the best result we can achieve.

### Software

The true DGP was set up in julia 1.10.2 [29] using the packages Bigsimr.jl [30] and Distributions.jl [31,32]. All other calculations were performed using R 4.3.3 [33]. For data generation, the R package bigsimr [34] was used which is built on the Bigsimr.jl-package. For benchmarking the classifiers, the R package mlr3 0.18.0 [35] together with mlr3measures [36] was used. This uses the LASSO and Ridge implementation from glmnet [37], the SVM implementation of e1071 [38], the KNN implementation of kknn [39] and the random forest implementation of ranger [40]. Rankcluster [41] is used to calculate the Kendall distances. For visualization, ggplot2 3.5.0, ggh4x and ggmosaic [42–44] are used. The simulations were conducted on the local compute cluster of the Department of Statistics at TU Dortmund University. The batchtools 0.9.17 package [45] was used for distributed computing.

The full code and simulation results for the simulation study are available on Zenodo (https://doi.org/10.5281/zenodo.13707473).

## Results

In the following, the results are presented. First, the true values for the performance measures are presented. Then, the errors of the comparison studies in estimating the performance of the classifiers are discussed. Afterward, the errors of the comparison studies in estimating the method ranking are presented. Finally, the results are summarized by analyzing the proportion of acceptable estimates. This analysis of the proportion of acceptable estimates summarizes the results regarding the errors of the comparison studies in estimating the performance of the classifiers concisely and can be understood without the more detailed discussion of the results before.

### True performances and method rankings

In Tables 4 to 7, the true performance measures and rankings for the classifiers are presented for each value of p=2,10,50,150. For low *p*, all classifiers achieve high performance according to all measures. For the highest *p* = 150, the performance decreased to moderate performance measure values. In general, the differences between the methods are not large. The ranks are obtained based on the true performance measure values such that rank one always corresponds to the best performance regarding the respective measure.

**Table 4:** True values for performance measures for the five classifiers averaged over 500 runs under the true scenario for p=2 and n=100.

	Ridge	LASSO	SVM	KNN	Random Forest
True Accuracy	0.9421	0.9512	0.9431	0.9345	0.9231
True AUC	0.9910	0.9924	0.9854	0.9726	0.9804
True Brier score	0.0625	0.0397	0.0446	0.0503	0.0594
True *F*_1_-score	0.9243	0.9383	0.9280	0.9179	0.9032
True Sensitivity	0.9048	0.9326	0.9245	0.9184	0.9033
True Specificity	0.9652	0.9627	0.9539	0.9432	0.9330
True Rank Accuracy	3	1	2	4	5
True Rank AUC	2	1	3	5	4
True Rank Brier score	5	1	2	3	4
True Rank *F*_1_-score	3	1	2	4	5
True Rank Sensitivity	4	1	2	3	5
True Rank Specificity	1	2	3	4	5

For *p* = 2, LASSO performs best with regard to many performance measures, followed by SVM, Ridge, KNN, and RF. For *p* = 10, the method order is Ridge, LASSO, SVM, KNN, and RF except for the Brier score for which SVM and Ridge are swapped and for the sensitivity, for which LASSO, KNN, Ridge, and SVM are swapped. For *p* = 50, Ridge and SVM are performing best, followed by LASSO, RF, and KNN. For *p* = 150, SVM performs best with respect to all measures, and LASSO performs worst according to all except for the Brier score. The remaining ranking differs more between the performance measures.

**Table 5:** True values for performance measures for the five classifiers averaged over 500 runs under the true scenario for p=10 and n=100.

	Ridge	LASSO	SVM	KNN	Random Forest
True Accuracy	0.8982	0.8885	0.8832	0.8588	0.8565
True AUC	0.9628	0.9533	0.9510	0.9300	0.9286
True Brier score	0.0909	0.0897	0.0861	0.1134	0.1165
True *F*_1_-score	0.8693	0.8642	0.8565	0.8355	0.8112
True Sensitivity	0.8641	0.8776	0.8624	0.8694	0.7920
True Specificity	0.9136	0.8913	0.8944	0.8433	0.8934
True Rank Accuracy	1	2	3	4	5
True Rank AUC	1	2	3	4	5
True Rank Brier score	3	2	1	4	5
True Rank *F*_1_-score	1	2	3	4	5
True Rank Sensitivity	3	1	4	2	5
True Rank Specificity	1	4	2	5	3

**Table 6:** True values for performance measures for the five classifiers averaged over 500 runs under the true scenario for p=50 and n=100.

	Ridge	LASSO	SVM	KNN	Random Forest
True Accuracy	0.7968	0.7391	0.7944	0.7099	0.7213
True AUC	0.8889	0.7954	0.8698	0.7624	0.7888
True Brier score	0.1489	0.1756	0.1418	0.2025	0.1938
True *F*_1_-score	0.6744	0.5858	0.7271	0.5822	0.5719
True Sensitivity	0.6408	0.5610	0.7161	0.5626	0.5334
True Specificity	0.8636	0.8190	0.8283	0.7692	0.8066
True Rank Accuracy	1	3	2	5	4
True Rank AUC	1	3	2	5	4
True Rank Brier score	2	3	1	5	4
True Rank *F*_1_-score	2	3	1	4	5
True Rank Sensitivity	2	4	1	3	5
True Rank Specificity	1	3	2	5	4

**Table 7:** True values for performance measures for the five classifiers averaged over 500 runs under the true scenario for p=150 and n=100.

	Ridge	LASSO	SVM	KNN	Random Forest
True Accuracy	0.6628	0.6323	0.7103	0.6327	0.6617
True AUC	0.7378	0.6263	0.7676	0.6552	0.7072
True Brier score	0.2181	0.2237	0.1914	0.2439	0.2169
True *F*_1_-score	0.4809	0.4454	0.6467	0.5175	0.5588
True Sensitivity	0.5061	0.4813	0.6448	0.5031	0.5562
True Specificity	0.6839	0.6602	0.7167	0.6788	0.6755
True Rank Accuracy	2	5	1	4	3
True Rank AUC	2	5	1	4	3
True Rank Brier score	3	4	1	5	2
True Rank *F*_1_-score	4	5	1	3	2
True Rank Sensitivity	3	5	1	4	2
True Rank Specificity	2	5	1	3	4

### Method failure

Within the comparison studies, fitting the classification methods to the simulated data may fail, which typically results in a warning message in case of non-convergence or in an error message in case no fit could be obtained at all. For Ridge and LASSO, non-convergence is an issue. Moreover, both models can not be fit if the data does not contain observations of both classes. In that case, SVM also outputs an error message. The random forest is still fit but outputs a warning message. KNN did not encounter any errors or warnings. In case of an error message, the fallback learner that always predicts the majority class is used. The numbers of iterations out of the total 100 iterations in which any warning or error message per scenario and classifier are given in Table A.1 to Table A.7 in Section A of S2 Appendix. Note that not necessarily all folds are affected.

For *p* = 2, no error messages are encountered in any scenario. There are only a few warning messages for Ridge and LASSO (Table A.1 in Section A of S2 Appendix). For *p* = 10, LASSO encountered many warnings (some in every scenario) which might indicate convergence issues. Ridge also encountered many warnings in all scenarios except for correlation and shift alternatives. For data generated from a standard normal distribution or with a scale of 0.25 or 0.5 it happened that no zeroes or no ones were generated. These are the cases where SVM encounters an error and RF a warning (Table A.2, A.3 in Section A of S2 Appendix). For the scale of 0.25 these are all iterations. For *p* = 50, it looks similar, but there are more warnings for RF and Ridge. For the scale of 0.25 and 0.5, and for a shift of ±0.5, in almost all iterations either no ones or no zeroes are generated (Table A.4, A.5 in Section A of S2 Appendix). For the scale of 4, also many iterations are affected. For *p* = 150, again many warnings are encountered for Ridge and LASSO. For the scale of 0.25, again no zeroes or ones are generated in any iteration. For the scale of 0.5 and for a shift of ±0.5 this happens in around one-third of the iterations (Table A.6, A.7 in Section A of S2 Appendix).

### Errors in estimation of performance measures

In the following, the performance of parametric and Plasmode simulation regarding the estimation of the classification performance measures for all classifiers are compared under different scenarios and using different resampling types for Plasmode. First, the resampling types for the Plasmode simulation are compared. Afterwards, the influence of different misspecifications of the data-generating process (DGP) and outcome-generating model (OGM) are discussed. The results are always presented according to the number of variables *p* and according to the classification performance measure. For each combination of *p* and each classification performance measure, the errors for estimating this measure are visualized using boxplots stratified by the simulation type, classifier, and potentially by the type of misspecification. This section gives a detailed discussion of the results. A summary of the results can be found afterward in the presentation of the proportions of acceptable estimates. That section can also be understood independently of the following detailed analysis of the results.

#### Comparison of resampling strategies for Plasmode.

In the following, under the true scenario, the errors in estimating the classification performances are compared between parametric simulation and Plasmode simulation with different resampling types. This comparison shows how well each simulation approach can perform at best when no wrong assumptions are made in the comparison study. The differences between the simulated accuracy and the true accuracy are displayed in a boxplot over 100 iterations. Ideally, the errors should all be close to zero since the assumptions in the simulations coincide with the truth. Positive values correspond to overestimation, and negative values to underestimation. The columns in each plot correspond to the type of simulation. The rows correspond to the classifier.

**Fig 2:**
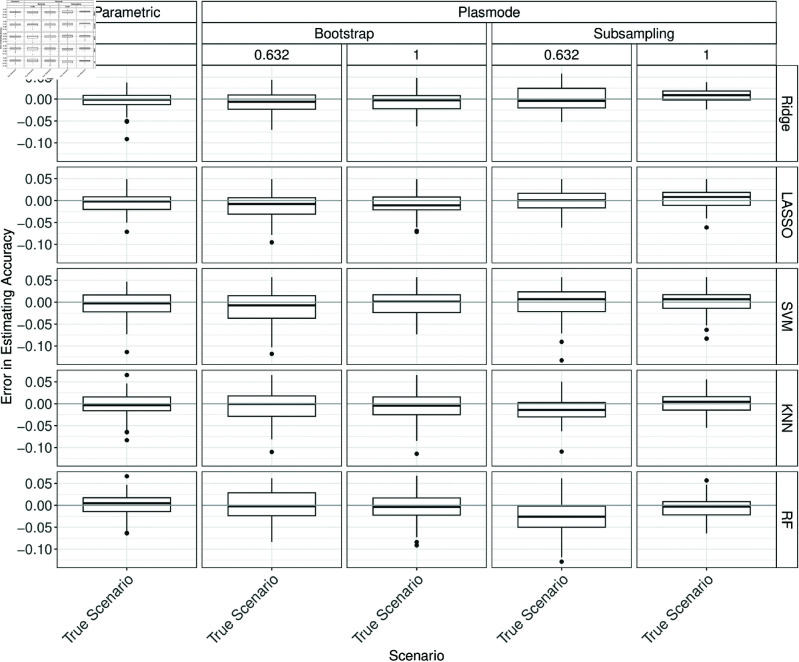
Errors in the estimation of accuracy in 100 iterations of a classification method comparison study per classifier for different simulation approaches under the true scenario for *p* = 2.

**Fig 3:**
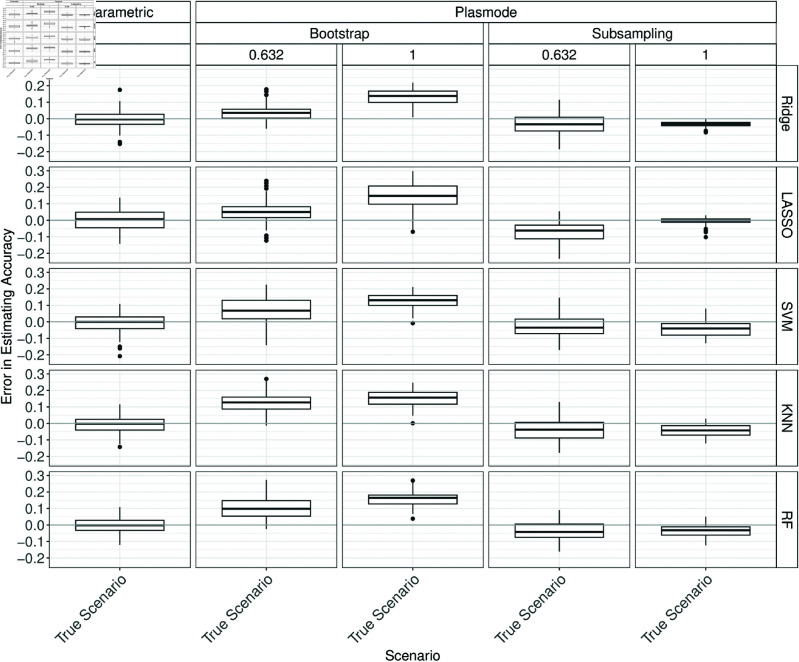
Errors in the estimation of accuracy in 100 iterations of a classification method comparison study per classifier for different simulation approaches under the true scenario for *p* = 150.

Overall, it can be seen that parametric simulation is often superior to Plasmode simulation as the median relative errors are typically closer to zero and the boxes are narrower indicating more stable estimation. In general, Plasmode seems to perform worse compared to parametric for increasing *p*. Compare for example the estimation errors for accuracy for *p* = 2 for the two Bootstrap types in Fig 2 to the corresponding ones for *p* = 150 in Fig 3.

In many cases, some Plasmode variant performs similarly well but no variant is consistently as good as parametric across all classifiers, measures, and values of *p*. There is no clear structure when which Plasmode variant performs well but often 0.632-subsampling performs worst for *p* = 10 (Figs B.6 to B.11 in Section B of S2 Appendix) and one of the Bootstrap types performs worst for *p* = 50 (Figs B.12 to B.22 in Section B of S2 Appendix). No resampling performs satisfactorily in most cases with few exceptions (e.g. for *p* = 150 and the *F*_1_-score, see Fig B.20). Which of the Bootstrap types to prefer depends on the concrete situation but often 0.632-Bootstrap is preferable over the ordinary Bootstrap. For example, 0.632-Bootstrap performs often well for *p* = 10, and often worse but still better than ordinary Bootstrap for *p* = 50, see Figs B.6 to B.11 and B.12 to B.17. Moreover, there is a tendency towards larger errors for all simulation types for the *F*_1_-score, specificity, and sensitivity, especially for high *p* and especially for Ridge and LASSO as classifiers. A reason for this might be that we observed that especially Ridge and LASSO tend to predict only ones or only zeroes when *p* gets larger and *n* is kept constant.

The plots for all combinations of *p* and classification performance measures can be found in Section B of S2 Appendix.

#### Shift.

In the following, it is discussed how misspecifying the shift in the DGP affects the ability of the parametric simulations to estimate the classification performances. For *p* = 2 and *p* = 10, almost no differences are visible between the errors for shifted data and the errors under the true scenario (see Figs C.1 to C.12 in Section C of S2 Appendix). For *p* = 2, one of the coefficients is positive and the other is negative with similar absolute values. Therefore, the effects of shifting both variables by the same amount might cancel out to some extent.

**Fig 4:**
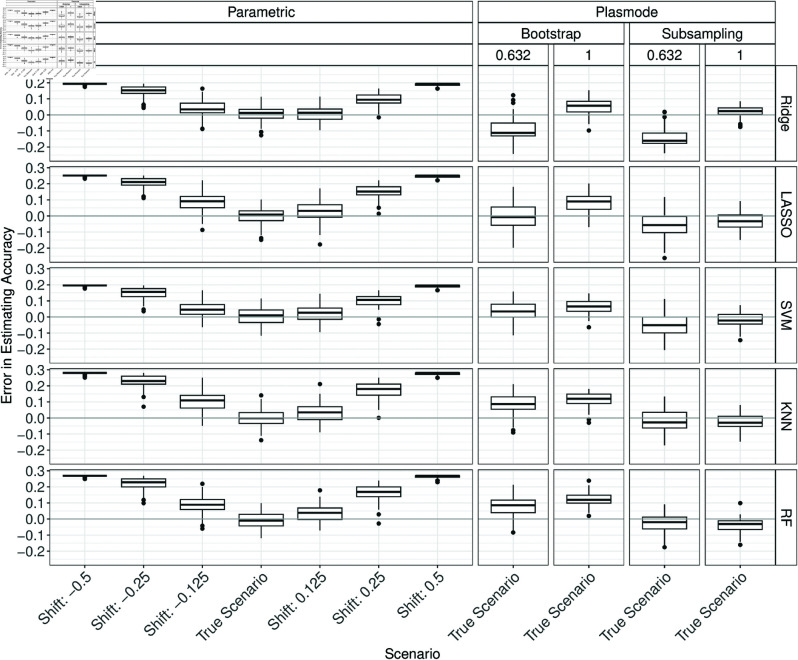
Errors in the estimation of accuracy in 100 iterations of a classification method comparison study per classifier for different simulation approaches with misspecified shift for parametric simulation for *p* = 50.

Fig 4 shows the errors in estimating the accuracy for *p* = 50 and misspecifications for shift for the parametric simulation. Note that for shifts of ±0.5, Ridge, LASSO, and SVM failed in almost all iterations and the fallback learner was used instead (see Table A.5 in Section A of S2 Appendix). An overestimation of the true accuracy can be observed for all shifts. The errors for parametric simulation based on shifted data quickly get worse than the well-performing Plasmode variants with resampling proportions of one.

**Fig 5:**
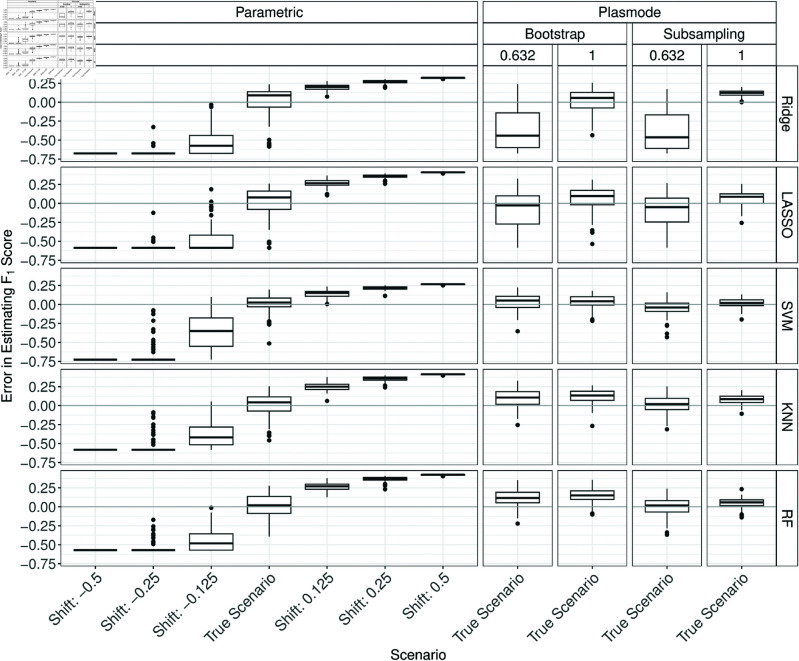
Errors in the estimation of the $F_{1}$ score in 100 iterations of a classification method comparison study per classifier for different simulation approaches with misspecified shift for parametric simulation for *p* = 50.

For AUC and the Brier score, an inverted pattern can be observed (see Figs C.13 and C.14 in Section C of S2 Appendix). The errors for the *F*_1_-score and sensitivity estimation increase with increasing shifts while the errors for specificity decrease with increasing shifts (see Fig 5, and Figs C.15 to C.16 in Section C of S2 Appendix). A possible explanation for these observations is that shifting the data seems to result in higher predicted probabilities and therefore more generated ones for positive shifts and lower predicted probabilities and therefore a higher proportion of zeroes. If a method then, e.g. for a positive shift, predicts mostly high probabilities it will achieve high performance with respect to accuracy, sensitivity, and *F*_1_ score as it correctly predicts the ones. On the other hand, the specificity decreases as true zeroes are often also predicted as one. For the extreme shifts and Ridge, LASSO, and SVM this happens since the fallback learner that always predicts the majority class is used in almost all iterations.

The results for *p* = 150 are similar to those for *p* = 50 (see Figs C.17 to C.22 in Section C of S2 Appendix).

#### Scale.

**Fig 6:**
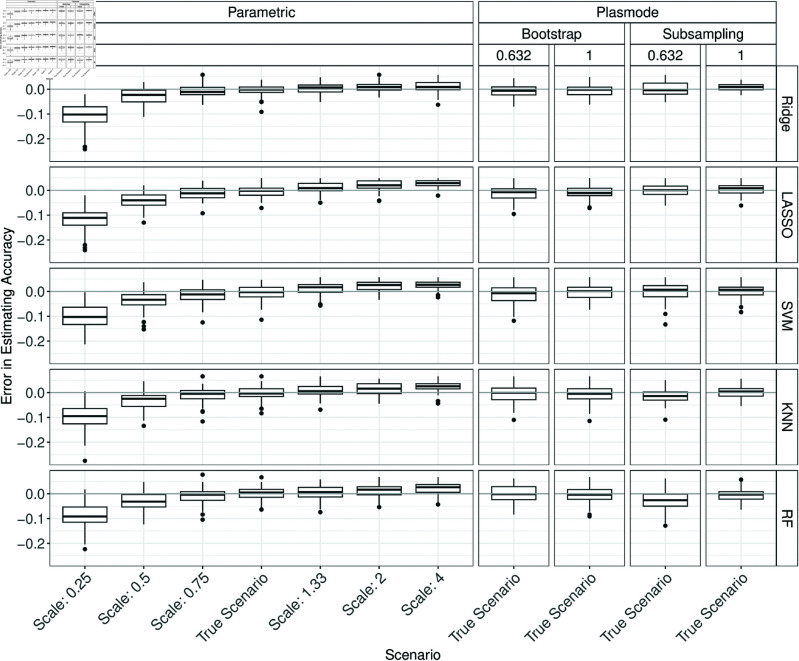
Errors in the estimation of accuracy in 100 iterations of a classification method comparison study per classifier for different simulation approaches with misspecified scale for parametric simulation for *p* = 2.

Fig 6 shows the errors in the estimation of accuracy when the scale in the parametric simulation is misspecified for *p* = 2. The true accuracy is underestimated for scales smaller than one and overestimated for scales larger than one.

For the AUC, the underestimation for small scales remains, but only slight differences can be observed for scales larger than one (see Fig D.1 in Section D of S2 Appendix), possibly because overestimation is almost impossible due to the true high AUCs. For the Brier score, the results are similar to the ones for accuracy but with inverted signs (Fig D.2 in Section D of S2 Appendix). The results for *F*_1_-score and sensitivity are similar to those for the AUC (Figs D.3 and D.4 in Section D of S2 Appendix). For specificity, an increase of the errors with increasing scale can be observed (Fig D.5 in Section D of S2 Appendix).

For *p* = 10, more extreme estimation errors compared to *p* = 2 can be observed for all measures, especially for the *F*_1_-score and sensitivity. The direction of over- and underestimation is not always consistent with that observed for *p* = 2 (see Figs D.6 to D.11 in Section D of S2 Appendix). Note that for small scales, especially for the scale of 0.25, Ridge, LASSO, and SVM failed in many up to all iterations, see Table A.3 in Section A of S2 Appendix.

For *p* = 50, and for a scale of 0.25, no AUCs and sensitivities could be estimated in any iteration, i.e. no ones were generated even after redrawing the data 50 times.

The true accuracy is overestimated for all scales, while the AUC and Brier score are underestimated (Figs D.13 to D.15 in Section D of S2 Appendix). For the *F*_1_-score and sensitivity, severe underestimation can be observed for small scales and overestimation for large scales (Figs D.15, D.16 in Section D of S2 Appendix). In the extreme case of small scales, the estimated measure is always close to zero, and for large scales always close to one. For specificity, the pattern observed for the *F*_1_-score and sensitivity is inverted (Fig D.17 in Section D of S2 Appendix).

The results regarding the estimation errors are very similar to those for *p* = 50 (see Figs D.18 to D.23 in Section D of S2 Appendix).

Overall, we observe that misspecifying the scale in the DGP for parametric simulation often results in errors that are larger than the errors for Plasmode simulation for which we cannot misspecify the DGP directly.

#### Correlation.

In the following, the effect of changing the correlation in the DGP for parametric simulation is discussed. For *p* = 2, there are almost no differences visible when changing the correlation structure except for slight deviations for fixed pairwise correlations of 0.2 (see Figs E.1 to E.6 in Section E of S2 Appendix).

For *p* = 10, almost no differences are visible (see Figs E.7 to E.12 in Section E of S2 Appendix).

**Fig 7:**
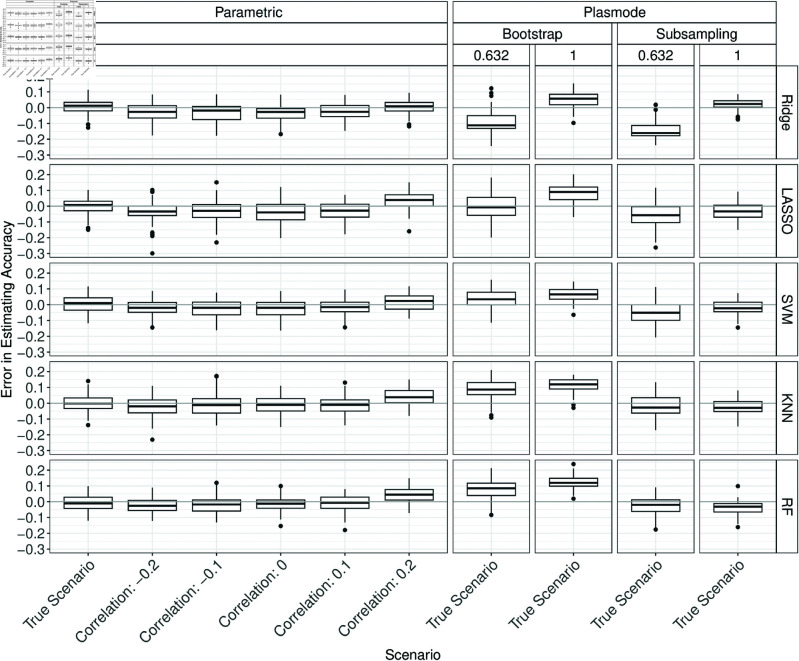
Errors in the estimation of accuracy in 100 iterations of a classification method comparison study per classifier for different simulation approaches with misspecified correlation for parametric simulation for *p* = 50.

For *p* = 50, there are slight differences visible except for specificity. The true measures are overestimated for a correlation of 0.2 and slightly underestimated for the other correlation values (vice versa for the Brier score). For accuracy, this is shown in Fig 7. The results for all other measures can be found in Figs E.13 to E.17 in Section E of S2 Appendix.

For *p* = 150, there are also slight errors in almost all cases (Figs E.18 to E.23 in Section E of S2 Appendix).

Overall, changing the correlation does not seem to affect the simulation results for parametric simulation in many cases. Often the errors made by misspecifying the correlation are still smaller than those of Plasmode simulation under the true scenario. However, it should be noted that the misspecifications here are not very large as the true correlations are mostly scattered around zero. Using correlations further away from the truth might lead to larger errors for parametric simulation but could not be investigated because of numerical problems as discussed in Section Deviations.

#### Complete misspecification as standard normal.

**Fig 8:**
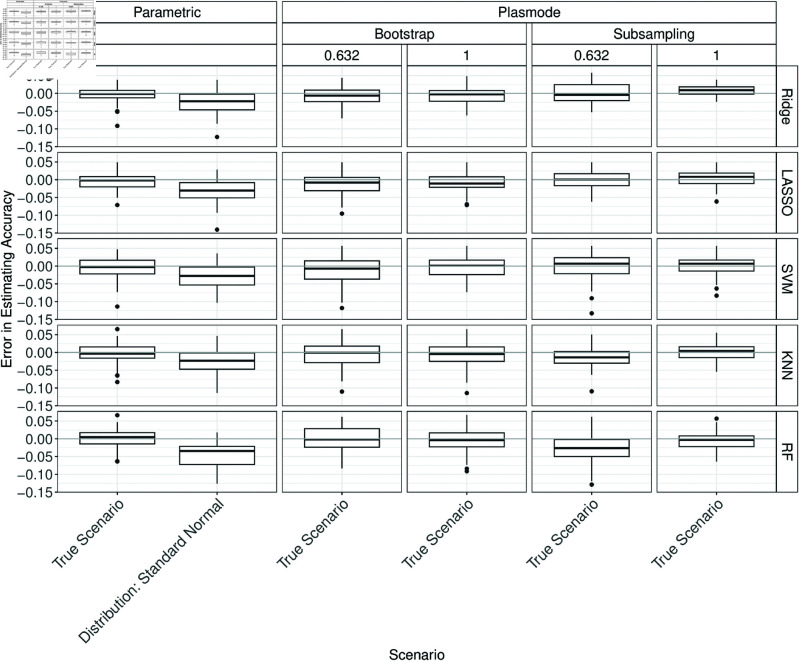
Errors in the estimation of accuracy in 100 iterations of a classification method comparison study per classifier for different simulation approaches with distribution misspecified as standard normal for parametric simulation for *p* = 2.

For *p* = 2, when misspecifying the distribution as a standard normal there are some differences visible for almost all measures and classifiers. The errors for accuracy, Brier score, and specificity are notable (Figs 8, and F.2, F.5 in Section F of S2 Appendix). For AUC, *F*_1_-score, and sensitivity only slight over- or underestimation occurs (Figs F.1, F.3, F.4 in Section F of S2 Appendix).

The true accuracies and specificities are overestimated notably while the AUCs, Brier scores, *F*_1_-scores, and sensitivities are underestimated notably (see Figs F.6 to F.11 in Section F of S2 Appendix).

For *p* = 50, slight errors are observed for accuracy, AUC, and Brier score (see Figs F.12 to F.14 in Section F of S2 Appendix). For *F*_1_-score and sensitivity, underestimation can be seen and for specificity, overestimation can be seen (see Figs F.15 to F.17 in Section F of S2 Appendix).

At least slight over- or underestimation can be observed in almost all cases for *p* = 150 (Figs F.18 to F.23 in Section F of S2 Appendix). For KNN, the median errors in estimating accuracy, AUC, and Brier score are almost zero. Note that the standard normal distribution approximates the distribution of many variables in the true DGP reasonably. This might explain why the resulting errors for parametric simulation are often not very large.

#### OGM.

**Fig 9:**
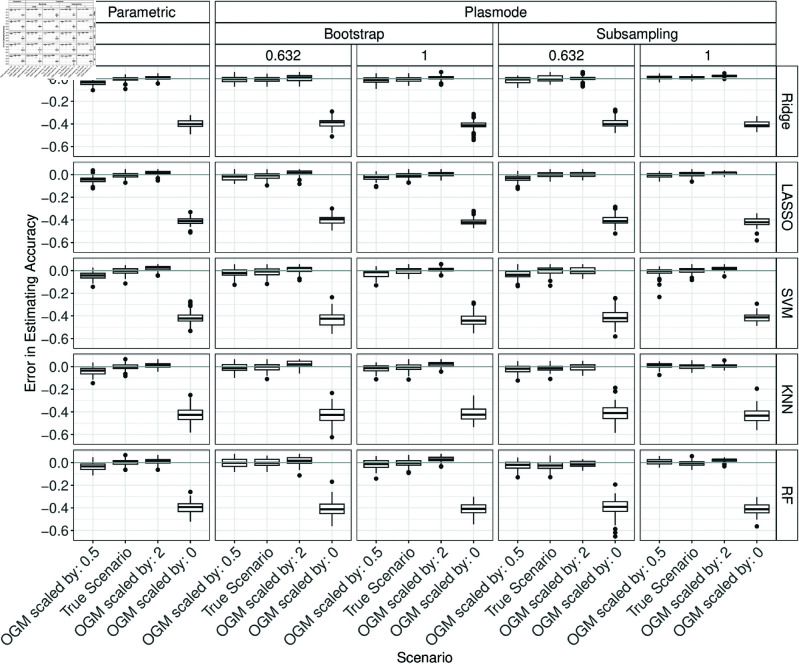
Errors in the estimation of accuracy in 100 iterations of a classification method comparison study per classifier for different simulation approaches with misspecifications of the OGM for *p* = 2.

In the following, the effect of misspecifying the OGM on the results of both parametric and Plasmode are presented. For *p* = 2, an increase in the errors from underestimation for OGMs scaled by 0.5 to (slight/very slight) overestimation for scaled by 2 and considerable underestimation for setting all coefficients to 0 can be observed for all measures except the Brier score for parametric as well as Plasmode simulation (Fig 9 below, and Figs G.1, G.3 to G.5 in Section G of S2 Appendix). For the Brier score, the pattern is inverted (Fig G.2 in Section G of S2 Appendix).

The results for *p* = 10 are similar to those for *p* = 2 (see Figs G.6 to G.11 in Section G of S2 Appendix).

For *p* = 50, the results are mostly similar to those for *p* = 2 and 10 (see Figs G.12 to G.17 in Section G of S2 Appendix). In part, overestimation for sensitivity and *F*_1_-score for the model with all coefficients set to zero can be seen, and the increase for 0.5 and 2 is less clear, in part even with a decrease for 2 again.

**Fig 10:**
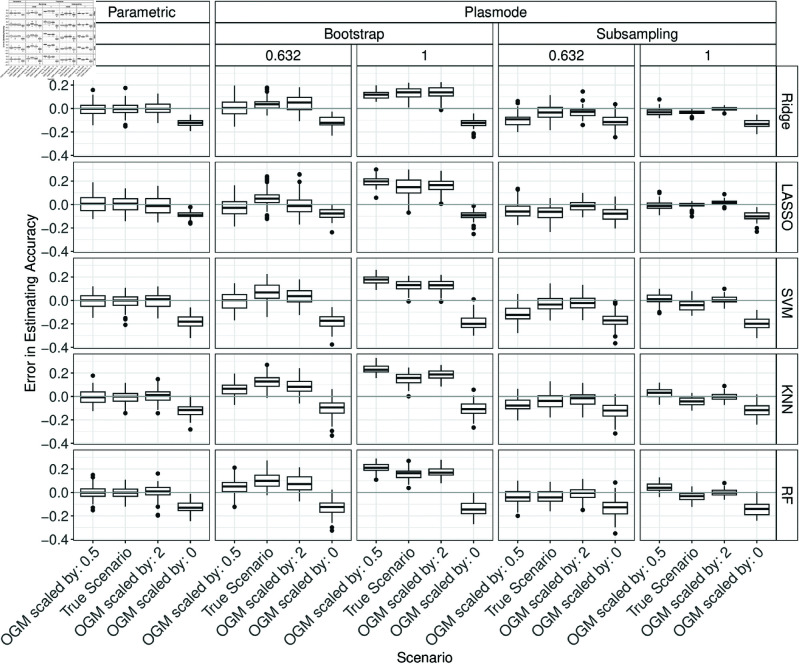
Errors in the estimation of accuracy in 100 iterations of a classification method comparison study per classifier for different simulation approaches with misspecifications of the OGM for *p* = 150.

For *p* = 150, results are again similar to those for p=2,10 but the pattern for scaled OGMs is less consistent (see Figs 10, and G.18 to G.22 in Section G of S2 Appendix). In the parametric case often no difference between these models scaled by 0.5 and 2 and the true OGM is visible. For Plasmode, typically some difference can be observed, but not necessarily an increase (see e.g. Fig 10).

### Errors in estimation of method ranking

In the following, the errors in estimating the true method ranking in the parametric or Plasmode comparison studies are discussed. Low ranks always correspond to good performance, independent of the measure. The Kendall distances of the simulated and true method rankings are displayed in boxplots analogously to the errors in the previous section. Fig H.1 in Section H of S2 Appendix shows the Kendall distances for 10000 pairs of randomly drawn rankings of 1,…,5 for comparison. The median Kendall distance is at 0.5 and the distribution is approximately symmetric around that value. Ideally, the simulated and true method rankings should show lower Kendall distances than these randomly drawn rankings.

**Fig 11:**
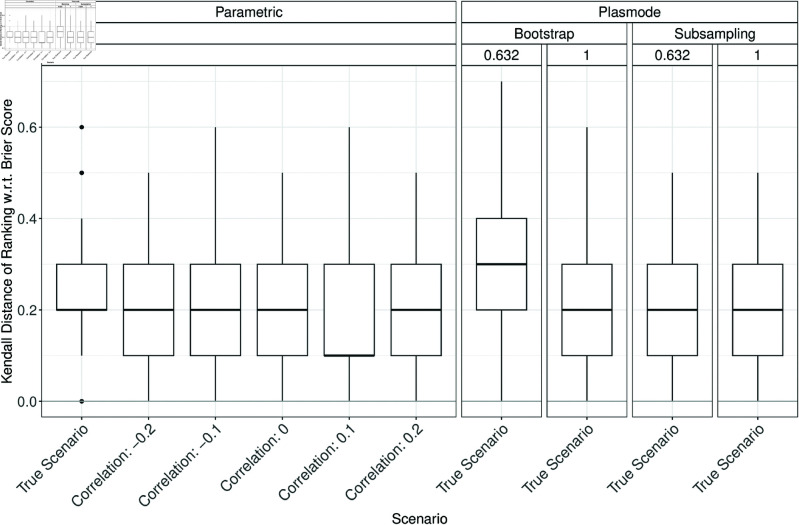
Kendall distance of the simulated and true method ranking based on the Brier score in 100 iterations of a classification method comparison study per classifier for different simulation approaches with misspecifications of the correlation for parametric simulation for *p* = 2.

**Fig 12:**
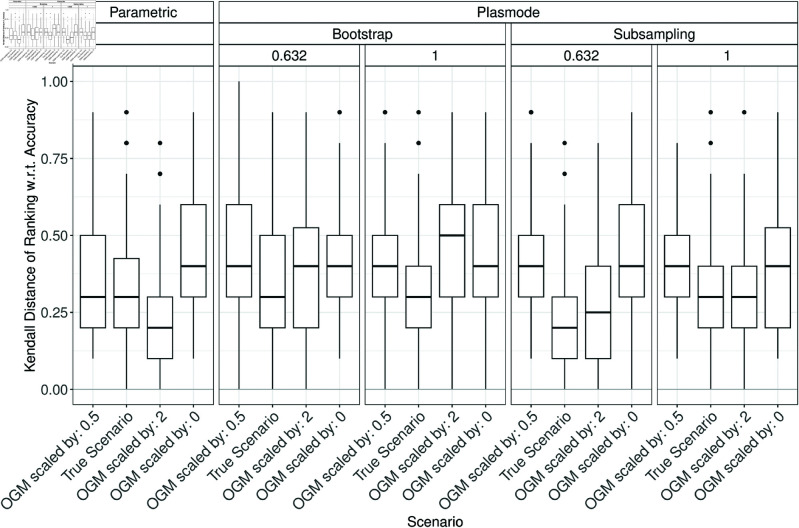
Kendall distance of the simulated and true method ranking based on accuracy in 100 iterations of a classification method comparison study per classifier for different simulation approaches with misspecifications of the OGM for *p* = 2.

**Fig 13:**
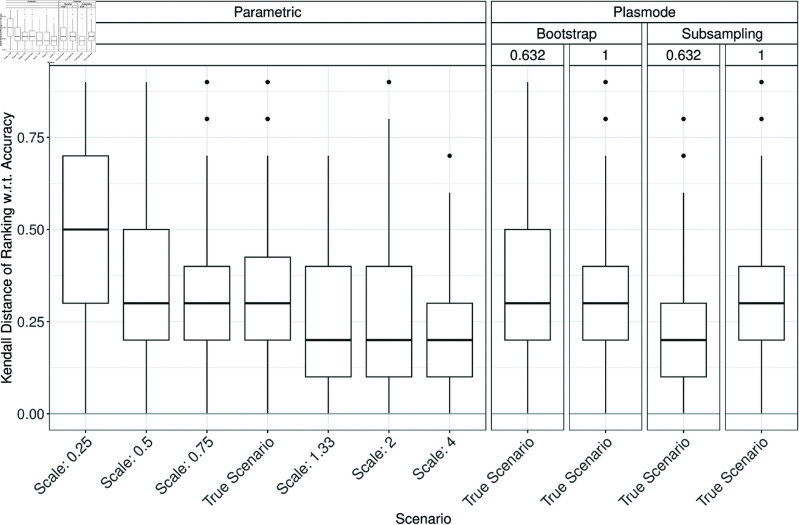
Kendall distance of the simulated and true method ranking based on accuracy in 100 iterations of a classification method comparison study per classifier for different simulation approaches with misspecifications of the scale for parametric simulation for *p* = 2.

When comparing the Kendall distances for *p* = 2, it can be seen that differences in the Kendall distance of the simulated and true rankings occur where notable errors in estimating the classification performance due to misspecifications of the DGP or OGM were observed previously. For example, the Kendall distance is only slightly influenced by changing the correlation as shown in Fig 11, but more heavily influenced by changing the OGM (Fig 12) or scale (Fig 13). Interestingly, the Kendall distance sometimes gets smaller for misspecifications than for the true scenario, see e.g. Fig 13. Also, the median Kendall distance of Plasmode simulation is often smaller than for parametric, especially for no resampling except for specificity (see Figs H.2 to H.16 in Section H of S2 Appendix). Overall, the results are more volatile than those for the estimation errors.

For *p* > 2, the results are similar to those for p=2 but Plasmode is usually performing worse than parametric again for increasing *p* under the true scenario (see Fig in Section H in S2 Appendix).

### Proportion of acceptable simulation results

To summarize the above findings, the proportions of acceptable estimates are discussed next. Therefore, the proportion of iterations with relative errors within the 2.5% to 97.5%-quantile interval of the relative errors for the parametric comparison studies under the true scenario are calculated, for each combination of *p*, simulation type, classifier, and measure for each deviation. The resulting proportions are displayed in heatmaps for all scenarios. Each cell corresponds to one combination of simulation type, measure, classifier, and scenario, as indicated by the facet and axis labels. Violet-colored cells indicate high proportions and thus a good performance. Pink, red, and orange colors already indicate increasingly worse performance, and yellow indicates that almost all iterations yielded unacceptable results.

**Fig 14:**
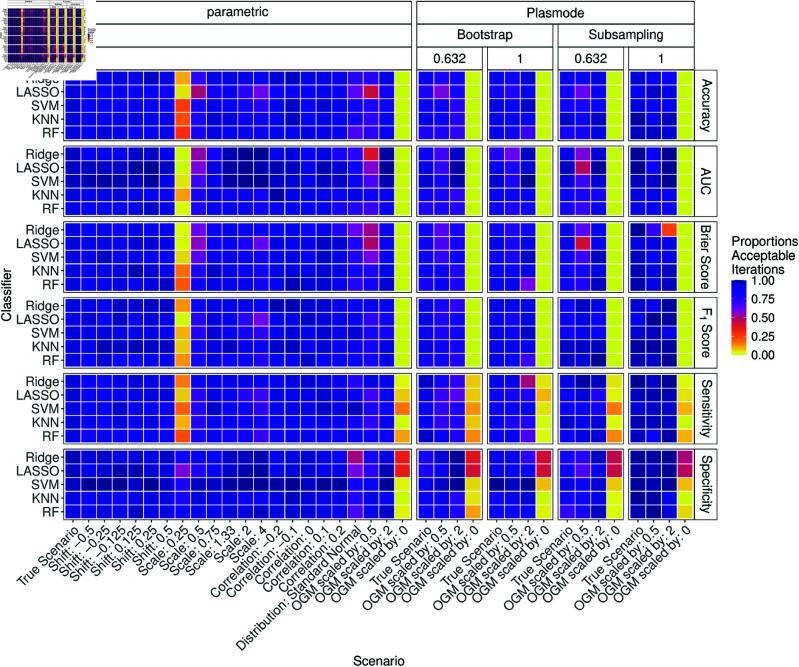
Proportion of acceptable iterations for 100 iterations of parametric and Plasmode method comparison studies under different scenarios for different classifiers and classification performance measures for *p* = 2. An iteration is defined as acceptable if its relative error for the respective measure lies within the 2.5% and 97.5% quantile of the parametric simulation error for the true scenario for that measure and classifier.

The results for p=2 are shown in Fig 14. It can be seen that in many cases the proportion of acceptable iterations is high. For all simulation types, the proportion of acceptable iterations is very low when all coefficients of the OGM are set to zero. Moreover, for all measures except for the specificity, the proportion is low for a scale of 0.25. Depending on the measure and classifier there are also moderate proportions for the higher values of scale, for setting the distribution to standard normal, and for the other modifications of the OGM. For Plasmode and the true scenario, we also observe some moderate values, especially for the 0.632 resamplings. No resampling performs very well here, except for the Brier score.

**Fig 15:**
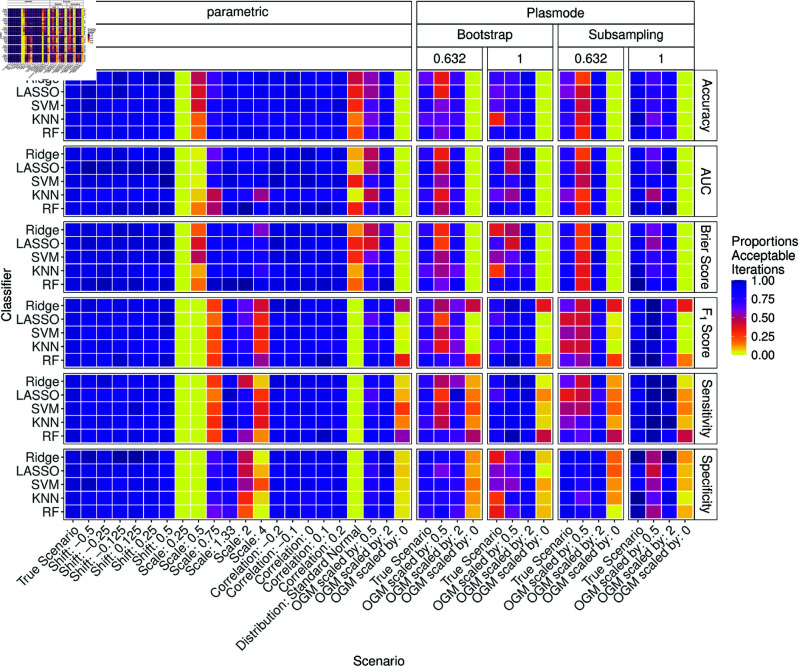
Proportion of acceptable iterations for 100 iterations of parametric and Plasmode method comparison studies under different scenarios for different classifiers and classification performance measures for *p* = 10. An iteration is defined as acceptable if its relative errors for the respective measure lie within the 2.5% and 97.5% quantile of the parametric simulation errors for the true scenario for that measure and classifier.

Fig 15 shows the results for *p* = 10. Compared to *p* = 2, many proportions of acceptable iterations decrease. Especially for scale alternatives and for setting the distribution to standard normal, smaller proportions are observed. Most Plasmode types also perform worse, except for no resampling for the true scenario.

**Fig 16:**
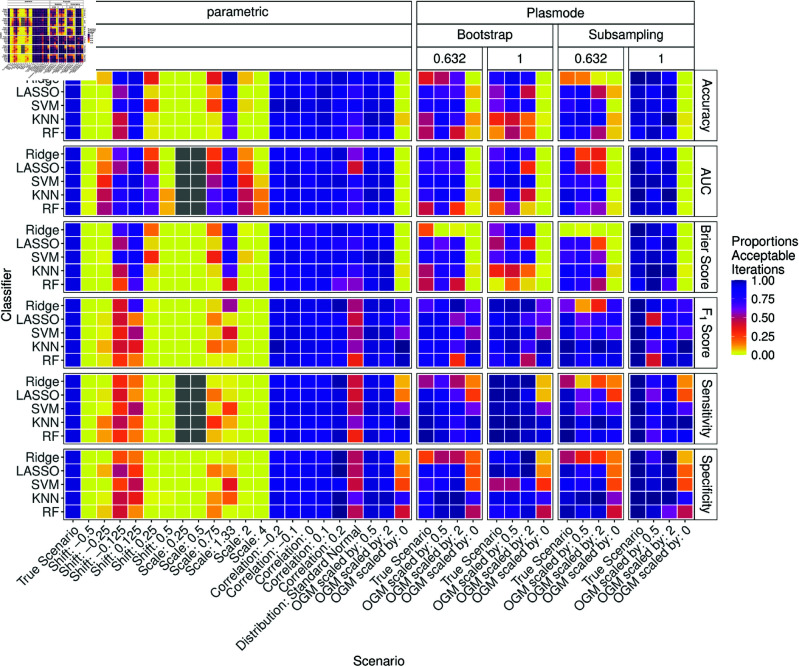
Proportion of acceptable iterations for 100 iterations of parametric and Plasmode method comparison studies under different scenarios for different classifiers and classification performance measures for *p* = 50. An iteration is defined as acceptable if its relative errors for the respective measure lie within the 2.5% and 97.5% quantile of the parametric simulation errors for the true scenario for that measure and classifier.

The results for *p* = 50 as shown in Fig 16 are even worse. For shift and scale alternatives, almost all results are unacceptable. For standard normal and for all coefficients of the OGM set to zero, the proportions of acceptable estimates are also often (very) low. The performance of Plasmode except for no resampling gets worse again, especially for the Ridge model and for some measures also for RF.

**Fig 17:**
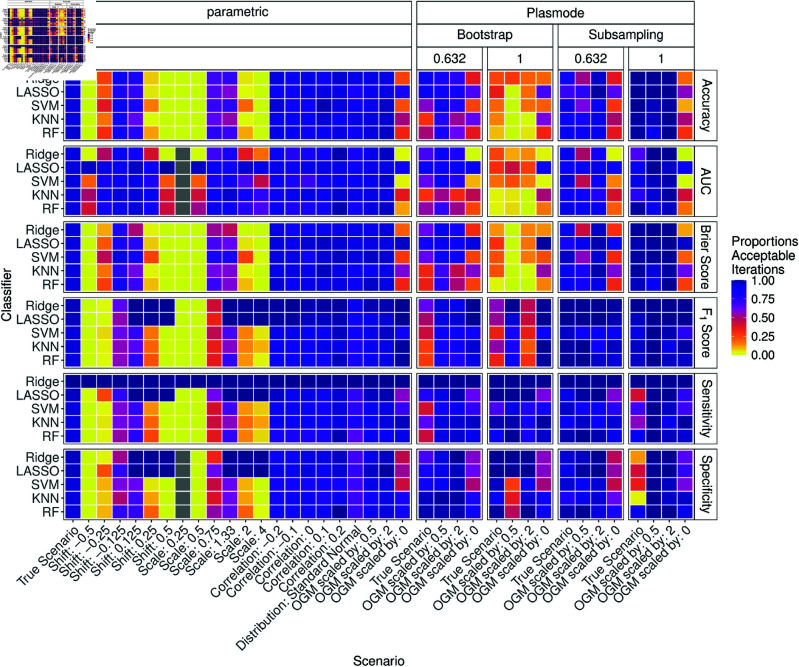
Proportion of acceptable iterations for 100 iterations of parametric and Plasmode method comparison studies under different scenarios for different classifiers and classification performance measures for *p* = 150. An iteration is defined as acceptable if its relative errors for the respective measure lie within the 2.5% and 97.5% quantile of the parametric simulation errors for the true scenario for that measure and classifier.

Fig 17 shows the results for *p* = 150. The results are similar to those for *p* = 50. The proportions for moderate shift and scale are not as low. The proportions of acceptable iterations for the ordinary Bootstrap and accuracy, AUC, and Brier score are now very low. The performance of the 0.632-Bootstrap and no resampling also drop, but not as much. 0.632-subsampling now performs comparably well. The results for Ridge and LASSO often show high proportions of acceptable simulation results.

## Discussion

We conducted a simulation study with the following tasks:

1. Compare how well parametric and Plasmode simulation can estimate the performance and method order for several classification methods.
2. Find out how misspecifications of the data-generating process (DGP) and outcome-generating model (OGM) affect parametric simulation in terms of estimating the performance and ranking of classification methods.
3. Find out how misspecifications of the OGM and different resampling strategies affect Plasmode simulation in terms of estimating the performance and order of classification methods.
4. Find out how the number of covariates affects the above.

Errors in the estimation of classification performance measured by accuracy, AUC, Brier score, *F*_1_-score, sensitivity, and specificity were compared as well as errors in the estimation of the resulting method ranking of five binary classification methods including Ridge and LASSO logistic regression, support vector machine (SVM), *K*-nearest neighbors (KNN), and random forest (RF). Additionally, the proportion of acceptable estimates was analyzed. An iteration was defined to be acceptable if its relative errors lie within the 2.5% and 97.5% quantile interval of the errors for parametric simulation assuming the true DGP and OGM. The analyses were each conducted for sample sizes *n* = 100 and numbers of variables p=2,10,50,150.

For all misspecifications, some errors could be observed for at least some combination of classification performance measure, classifier, and number of variables *p*. The magnitude and sign of the errors depended on the exact settings. Often, the errors observed for the estimation of the Brier score were similar to those for accuracy but with inverted signs. Errors for estimating *F*_1_-scores were similar to those for the sensitivity. A reason for this might be that the *F*_1_-score is the harmonic mean of precision and recall, where recall equals sensitivity. Errors in estimating specificities often showed inverted patterns to those for the *F*_1_-scores and sensitivities, probably since improved classification of true ones typically leads to a worse classification of true zeroes. In general, often, more extreme errors were observed for estimating the *F*_1_-score, sensitivity, and specificity, while only smaller errors were observed for estimating the AUC. Misspecifications of the OGM affect both parametric and Plasmode simulations similarly. For misspecifications of the DGP, only the parametric simulation is affected. Such misspecifications of the DGP can lead to severe errors in the parametric method comparison study. In those cases, Plasmode is the more robust choice. Overall, the observed errors were more severe for larger values of *p* in most cases.

With regard to the resampling strategies for Plasmode simulation, no clear conclusion could be drawn. Often, 0.632-subsampling led to comparably large errors and no resampling to comparably small errors, but this was not consistent across all classification performance measures and all values of *p*. Which of the Bootstrap types performed better was different depending on the specific scenario. The performance of the Plasmode simulations decreased for larger values of *p*.

It should be noted that the smaller the resampling proportion and the higher the number of duplicate observations in the Plasmode dataset, the less information is available for the classifier during training, which could affect its performance systematically for all resampling types except no resampling.In summary, we observed:

1. As expected, under the true scenario parametric simulation performs better than Plasmode with regard to estimating the classification performance.
2. Misspecifications of the DGP lead to errors in parametric simulation that quickly get larger than the errors for Plasmode, for which we cannot misspecify the DGP directly.
3. Misspecifications of the OGM affect parametric simulation and Plasmode simulation equally in terms of estimating the classification performance.
4. With regard to the resampling used for Plasmode, no resampling type consistently outperformed the others. However, often no resampling at all performed well and subsampling with a resampling proportion of 0.632 performed badly.
5. An increase in the number of variables decreases the ability to estimate the classification performance, especially for Plasmode simulations.

One limitation of the study conducted here is that it was infeasible to keep the true OGMs constant for different values of *p* and at the same time have reasonable true classification performances of the classifiers. Therefore, the true OGMs depend on *p* and the effects due to the OGM and due to the dimension cannot be separated. Nonetheless, the observed performance decrease of simulations, especially for Plasmode, is in line with previous results of a study for lower numbers of variables and the estimation of the MSE of the least squares estimator in linear regression [7]. In that study, the true models were kept constant across different values of *p*. Therefore, it seems reasonable that this effect can mainly be attributed to the value of *p* rather than the subtle differences in the OGMs. In contrast, the shift of variables had almost no effect for *p* = 2 and *p* = 10, but clear effects for larger *p*. This could be explained by the concrete coefficients of the models for the lower *p*s and therefore can probably be attributed to the differences in the models rather than to the dimension of the data. Overall, the scalability of Plasmode simulations seems questionable since we observed increasing errors for increasing numbers of variables in both studies. More research regarding this aspect is needed. On the other hand, in practical applications, the use of parametric simulation for high-dimensional data is hard as the number of marginal distributions and especially the number of pairwise correlations to be specified increases with the number of variables. Thus, it is reasonable to assume that the problem of over-simplification and therefore misspecifications of the DGP in parametric simulation also increases with the number of variables. The chosen numbers of variables *p* in this study represent the range of common numbers of variables of real-world datasets of low to moderately high dimensions. They are however not representative of ultra-high-dimensional data. Higher numbers of variables were infeasible to use within the simulation study due to runtime.

Another limitation of this study is that the number of samples and the true OGM and DGP were not varied which restricts the scope of this study. This limited number of scenarios, the comparably low number of iterations per scenario, and the exclusion of some classifiers are due to the high runtime and limited computing capacity. The effect of changing the number of samples and the true OGM and DGP are left open for further research.

## Supporting information

S1 Appendix Additional information on simulation setup. Detailed description of the true DGP, discussion of the use of fitted models as true models, coefficients for true OGM, and predicted probabilities for true OGMs.
(PDF)

S2 Appendix Additional Result Figures and Tables. Tables containing numbers of error and warning messages, additional figures for errors in performance estimation, and Kendall distances of true and simulated method rankings.
(PDF)

S3 File Marginal distributions of true DGP as CSV-file. (CSV)

S4 File Correlation matrix of true DGP as CSV-file. (CSV)
