# Supplementary material for: Simulation study to evaluate when Plasmode simulation is superior to parametric simulation in comparing classification methods on high-dimensional data
Source: PLoS One. 2025 Jun 2;20(6):e0322887. doi: 10.1371/journal.pone.0322887 (PMC12129352; doi:10.1371/journal.pone.0322887)
Supplement: S1 Appendix — Detailed description of the true DGP, discussion of the use of fitted models as true models, coefficients for true OGM, and predicted probabilities for true OGMs. (PDF) [file pone.0322887.s001.pdf]

Supplement 1: Additional Information on Simulation Setup for "When is Plasmode simulation superior to parametric simulation for comparing classification methods on high-dimensional data?"

Marieke Stolte[1*] Nicholas Schreck[2,3] Alla Slynko[4] Maral Saadati[2]

Axel Benner[2] Jörg Rahnenführer[1] Andrea Bommert[1]

and for the topic group "High-dimensional data" (TG9) of the STRATOS initiative

[1]Department of Statistics, TU Dortmund University
[2]Division of Biostatistics, German Cancer Research Center
[3]Faculty of Liberal Arts and Sciences, Technical University of Applied Sciences Augsburg
[4]Department of Statistics and Actuarial Science, University of Waterloo

A Detailed Description of True DGP

The sampling from the specified DGPs uses a normal-to-anything (NORTA) approach (Knudson and Schissler 2024). This approach can in theory handle diverse marginal distributions and correlation structures and is chosen for its flexibility here. Essentially, data is drawn from a multivariate normal first and then transformed to match the desired marginal distribution types. After this transformation step, the correlation structure is typically not equal to the one specified for the multivariate normal anymore. Therefore, Pearson matching is employed to calculate a correlation matrix for the multivariate normal such that the variables after transformation have the desired correlation structure. For a certain pair of marginal distributions, not the whole interval $[-1, 1]$ of correlations is feasible. The Pearson bounds give the interval of feasible correlations. These can be approximated numerically (Lebrun and Dutfoy 2009; Xiao and Zhou 2019). The correlation structure has to be specified such that it matches the feasible correlations for the specified marginal distributions. For the normal distribution, we generate 50 variables for which the means are randomly sampled from a normal distribution with a mean of 0 and variance of 2, and the standard deviations are randomly sampled from an inverse gamma distribution with parameters $\alpha = 14$ and $\beta = 15$. The parameters of the distributions we sample from are chosen such that the expected mean is zero and the expected variance is one. Moreover, the parameters of the inverse gamma distribution are chosen such that large standard deviations are sampled with very low probability since larger values led to numerical issues during the Pearson matching step in the data generation. The density of the Gamma(14,15)-distribution is displayed in Fig A.1.

*Corresponding author, e-mail: stolte@statistik.tu-dortmund.de

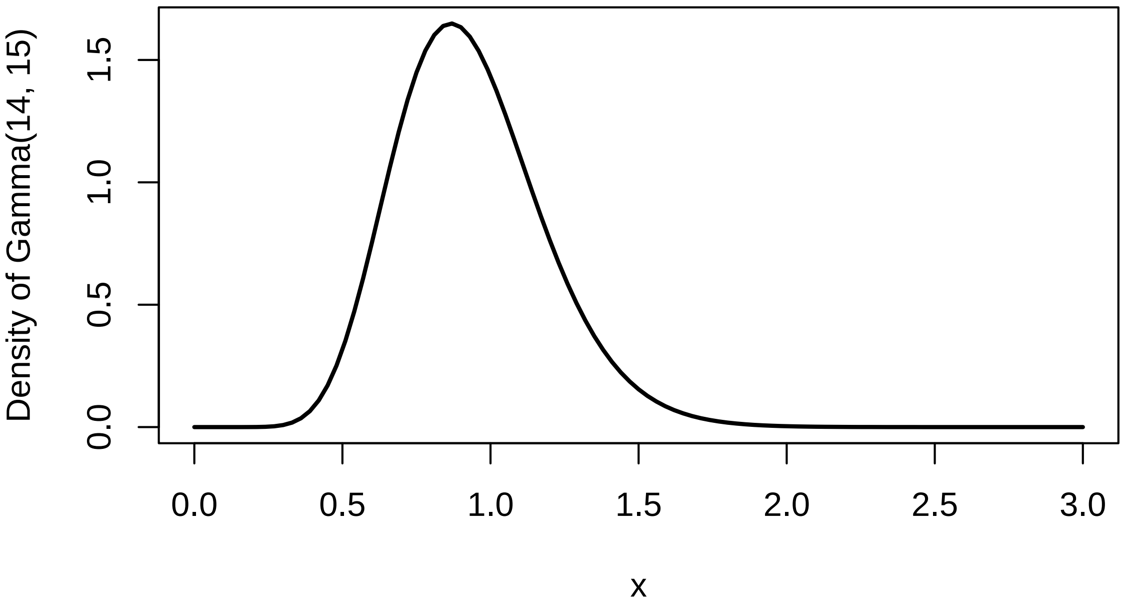

Fig A.1. Density of Gamma(14,15) distribution.

For the log-normal distribution, 50 variables are generated and the parameters μ and σ are generated in the same way as for the normal variables.

For the Gaussian mixture distribution, also 50 variables are generated. Half of these are generated from bimodal distributions and the other half is generated from a contamination model. The mixing proportions are drawn from a $U[0.05, 0.95]$-distribution in all cases. For the bimodal variables, the means for the first component are sampled from a standard normal distribution, but the means for the second component are sampled from a normal distribution with a mean of 4 instead. For both components, the variances are sampled from an inverse gamma distribution with parameters $\alpha = 3$ and $\beta = 2$, i.e. mean of one. For the contamination models, the means for both mixture components are sampled from a standard normal distribution. The variances for the first component are sampled from an inverse gamma distribution with parameters $\alpha = 3$ and $\beta = 2$ which again yields an expected variance of one. The variances for the second component are sampled from an inverse gamma distribution with $\alpha = 2.5$ and $\beta = 15$ which yields an expected variance of 10. Drawing the parameters produces more diverse marginal distributions than specifying the values by hand. The parameters for all distributions are drawn only once and set as the true parameters for these true distributions for the whole simulation.

For the generation of the correlation matrix, in the first step, the midpoint of the Pearson bounds (i.e. the interval of feasible correlations) for each pair of marginal distributions is calculated. This does not necessarily yield a positive definite matrix. Therefore, the resulting matrix of midpoints for all pairs of marginal distributions is then used as the expectation of an inverse Wishart distribution from which the true correlation matrix is drawn. The variance of the inverse Wishart is set to a low value chosen by trying out how small it has to be to get no generated correlations outside the Pearson bounds. This approach ensures drawing feasible correlations with high probability while still inducing

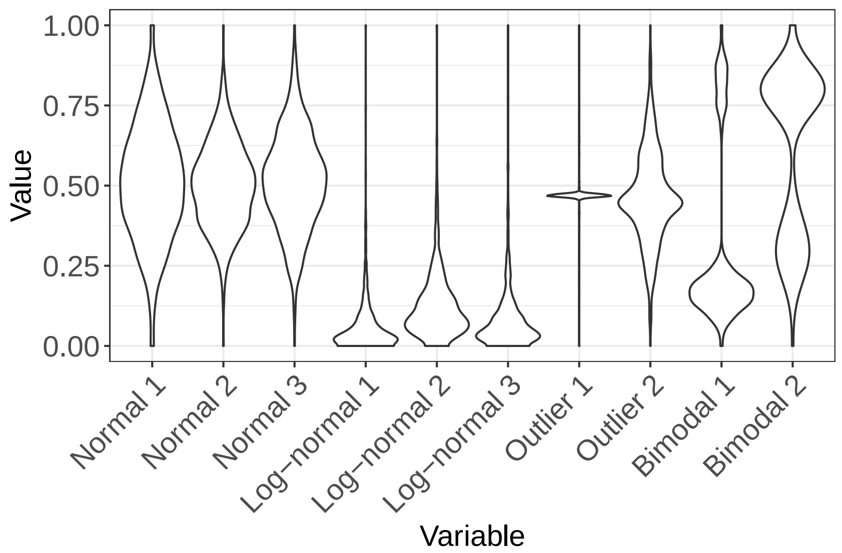

Fig A.2. Violin plot of 1000 observations drawn from the true DGP for $p = 10$.

some randomness and therefore creating a more complex correlation structure. Again, we only draw once and save the resulting matrix as our true correlation matrix.

We have to ensure that also deviations of the DGP in the comparison studies do not lead to numerical problems. Therefore, we try to perform the Pearson matching step for deviating correlation matrices with fixed pairwise correlations of -0.2, -0.1, 0, 0.1, and 0.2. Whenever a problem is encountered, we redraw the parameters of one of the affected distributions until there are no more problems. In total, we had to redraw the parameters for six distributions. Four of these were log-normal for which we redrew the parameter σ as these were untypically high values and reducing the value of this parameter solved the numerical issues. The remaining two distributions were Gaussian mixture distributions with a mixture component with high variance for which we redrew the means as these

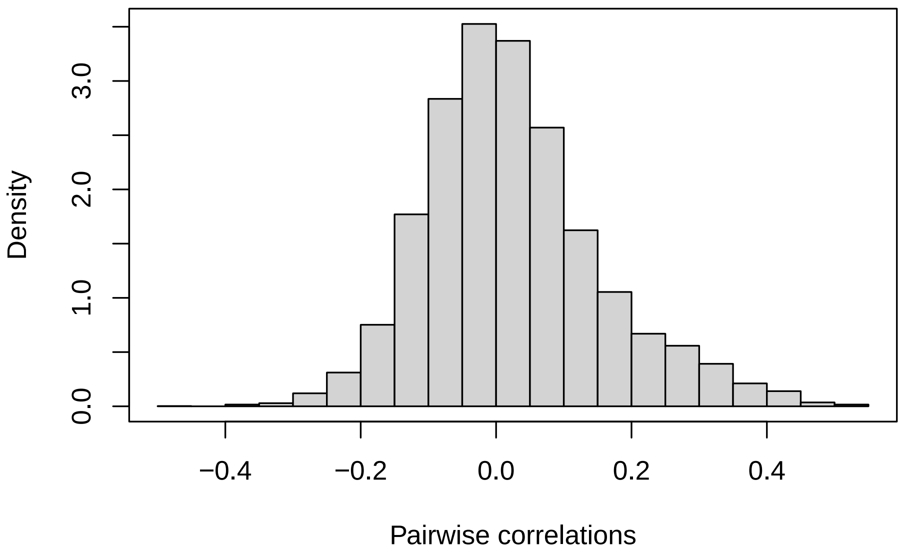

Fig A.3. Histogram of pairwise correlations in true DGP for $p = 150$.

seemed to be causing the problems. The marginal distributions after redrawing some of the parameters as described above were then saved as the true margins.

We originally intended also to include binary features in the true DGP, but this resulted in unsolvable numerical problems in the data generation. In many cases, only small intervals were feasible for the pairwise correlations with other variables and even within the intervals of feasibility returned by the Pearson bounds algorithm implemented in Bigsimr in julia (Knudson and Schissler 2024; Bezanson et al. 2017), the subsequent Pearson matching failed in many cases (hundreds to thousands depending on the choice of distribution from which the parameters for the Bernoulli variables were sampled), returning NaNs (not a number).

B Discussion of Use of Fitted Models as True Models

To ensure a fair comparison, it would be best to use each model included in the method comparison once the true model since the true OGM might favor certain classification methods in the comparison (e.g. a highly non-linear relationship might give an advantage to KNN and random forest over Ridge and LASSO). However, the explicit model specification is hard for more complicated models. E.g. specifying a random forest with hundreds of trees is infeasible in practice. An appealing solution to this would be to fit the models on a real dataset with a matching number of variables and output type (binary variable) and use this as the "true" model to generate the outcomes for the true scenario using simulated covariate data from the true DGP. The problem with this approach is that the performances of the models fitted to data generated from these fitted OGMs turned out to be much lower than the performance of these fitted OGMs, especially for low n and large p. Therefore, we discard this approach and use a logistic model as true OGM since with this approach we can control the true separation of the two classes easily.

C Coefficients for true OGM

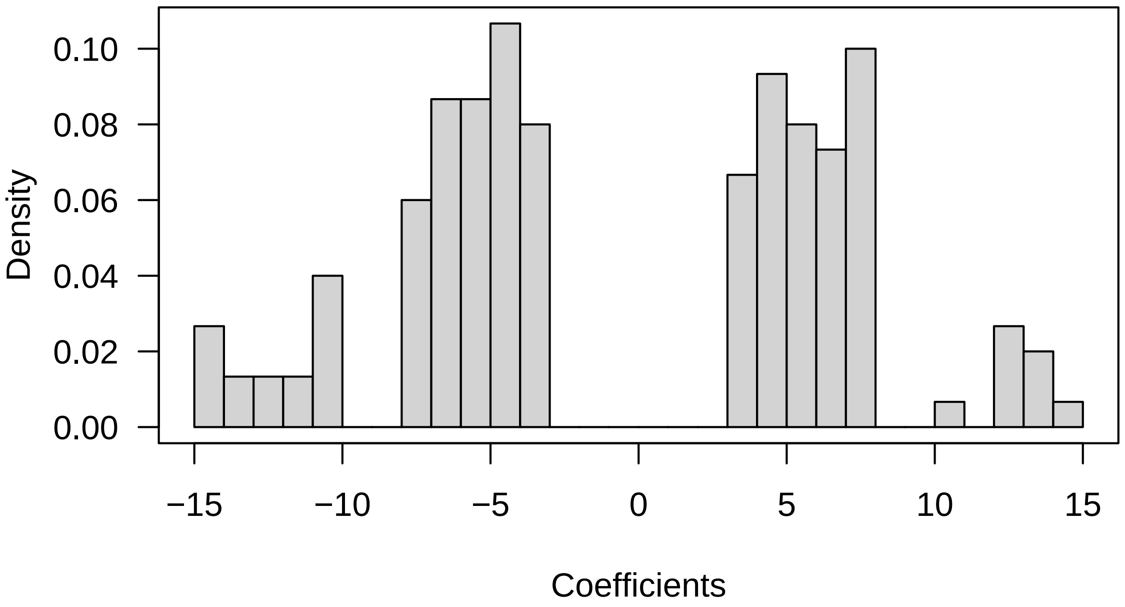

Fig C.1. Histogram of the generated true coefficients for $p = 150$.

D Predicted Probabilities for True OGMs

For $p = 50$, the coefficients for the first 15 normal and log-normal variables and the first 10 bimodal and outlier variables are chosen each, and a 4 is added or subtracted to all positive values and negative coefficients, respectively. For the bimodal variables, the coefficients are increased or decreased by 6, again, to improve separation. The intercept is set to -71.5, again to achieve balance. The resulting predicted probabilities again show a good separation and approximate balance (Fig D.2).

For $p = 10$, the coefficients for the first three normal and log-normal variables and the first two bimodal and outlier variables are chosen. The intercept is set to -9.6. The resulting predicted probabilities again show a good separation and approximate balance (Fig D.3).

For $p = 2$, the coefficients for the first normal and bimodal variables are chosen and multiplied by 6 and -2, respectively. The intercept is set to -1.5. The resulting predicted probabilities again show a good separation and approximate balance (Fig D.4).

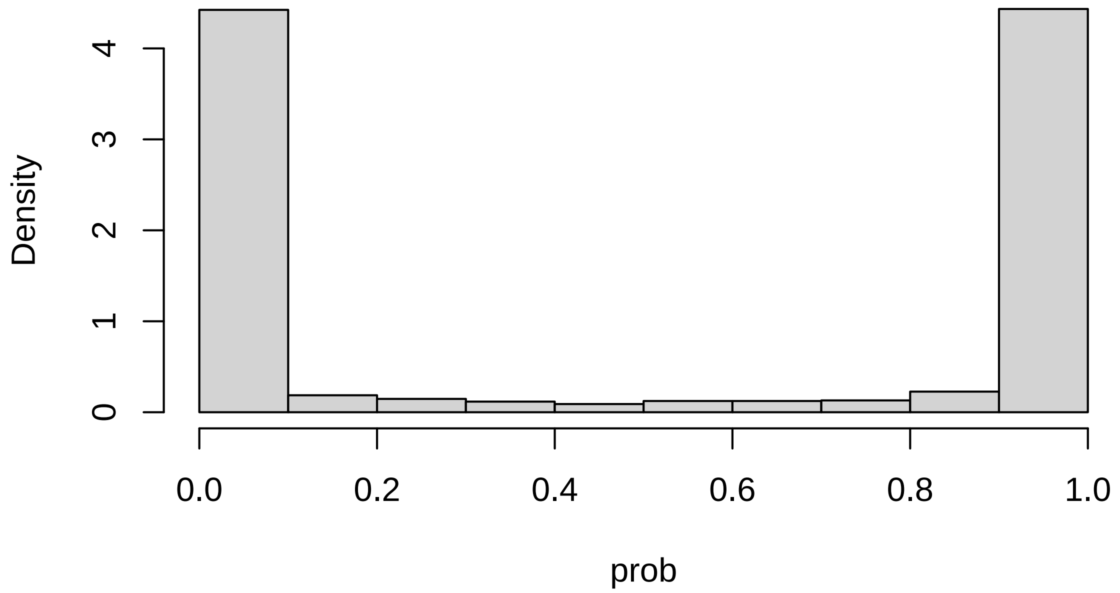

Fig D.1. Predicted probabilities for 3000 observations generated according to the true DGP and OGM for $p = 150$

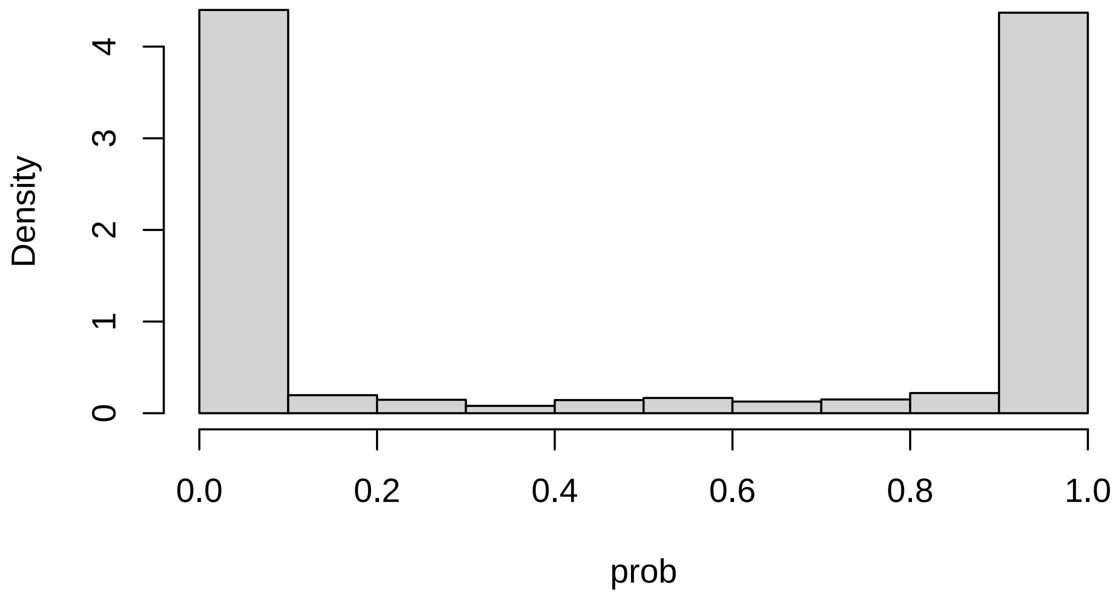

Fig D.2. Predicted probabilities for 3000 observations generated according to the true DGP and OGM for $p = 50$

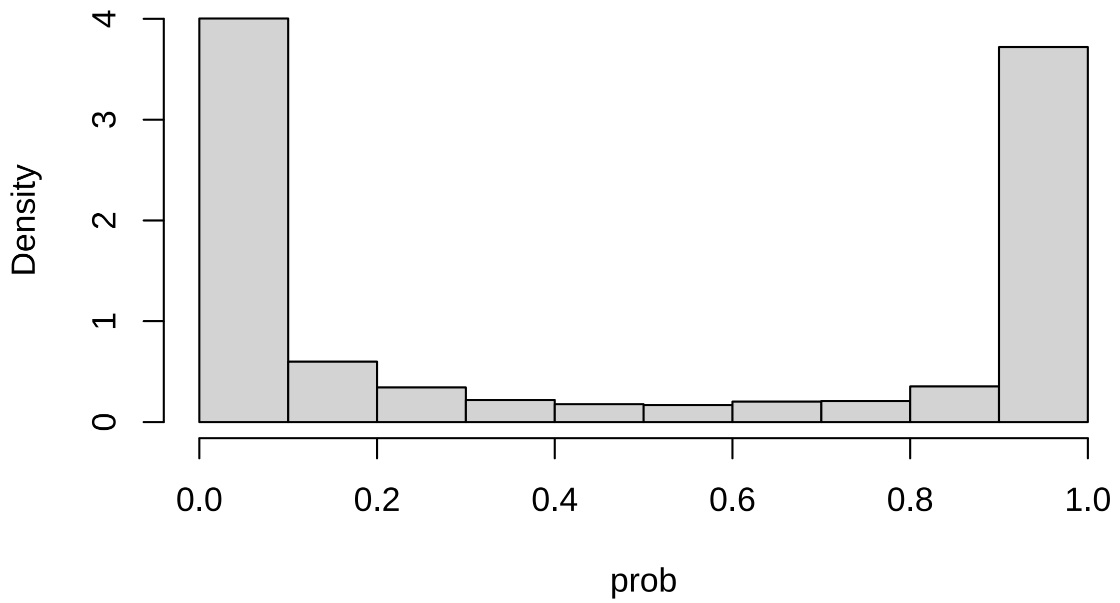

Fig D.3. Predicted probabilities for 3000 observations generated according to the true DGP and OGM for $p = 10$

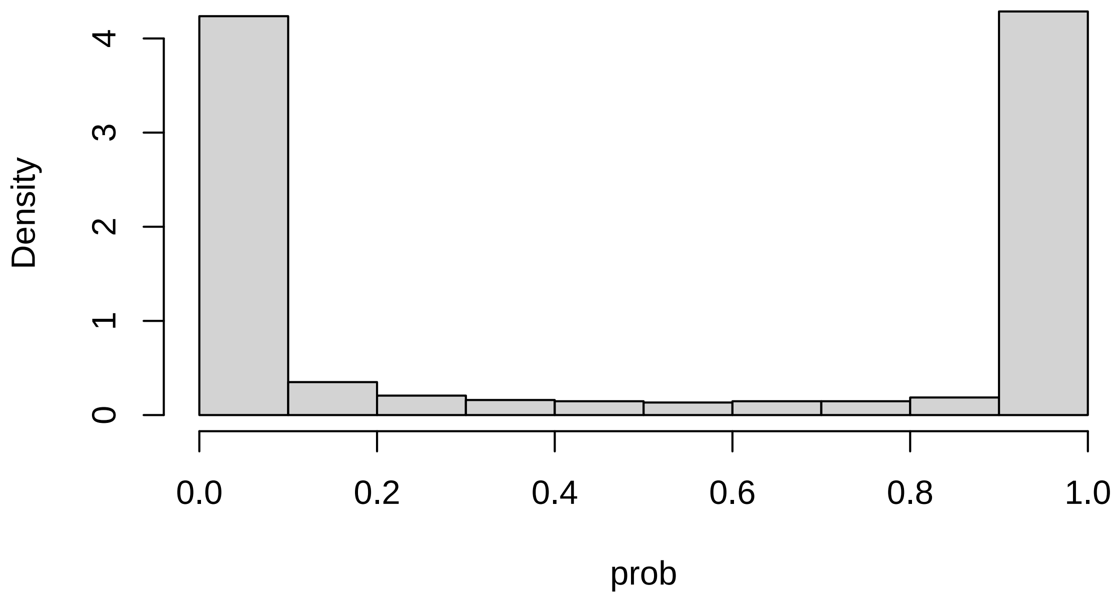

Fig D.4. Predicted probabilities for 3000 observations generated according to the true DGP and OGM for $p = 2$

References

Bezanson, J., A. Edelman, S. Karpinski, and V. B. Shah (2017). "Julia: A fresh approach to numerical computing". In: *SIAM review* 59 (1), pp. 65–98. URL: https://doi.org/10.1137/141000671.

Knudson, A. and G. Schissler (2024). *Bigsimr.jl: Simulate multivariate distributions with arbitrary marginals.* URL: https://github.com/SchisslerGroup/Bigsimr.jl.

Lebrun, R. and A. Dutfoy (July 2009). "An innovating analysis of the Nataf transformation from the copula viewpoint". In: *Probabilistic Engineering Mechanics* 24 (3), pp. 312–320. ISSN: 0266-8920. DOI: 10.1016/j.probengmech.2008.08.001. URL: https://www.sciencedirect.com/science/article/pii/S0266892008000660.

Xiao, Q. and S. Zhou (Apr. 2019). "Matching a correlation coefficient by a Gaussian copula". In: *Communications in Statistics - Theory and Methods* 48 (7), pp. 1728–1747. ISSN: 0361-0926. DOI: 10.1080/03610926.2018.1439962. URL: https://doi.org/10.1080/03610926.2018.1439962.
