# Supplementary material for: Simulation study to evaluate when Plasmode simulation is superior to parametric simulation in comparing classification methods on high-dimensional data
Source: PLoS One. 2025 Jun 2;20(6):e0322887. doi: 10.1371/journal.pone.0322887 (PMC12129352; doi:10.1371/journal.pone.0322887)
Supplement: S2 Appendix — Tables containing numbers of error and warning messages, additional figures for errors in performance estimation, and Kendall distances of true and simulated method rankings. (PDF) [file pone.0322887.s002.pdf]

Supplement 2: Additional Result Figures and Tables for "When is Plasmode simulation superior to parametric simulation for comparing classification methods on high-dimensional data?"

Marieke Stolte[1*] Nicholas Schreck[2,3] Alla Slynko[4] Maral Saadati[2]

Axel Benner[2] Jörg Rahnenführer[1] Andrea Bommert[1]

and for the topic group "High-dimensional data" (TG9) of the STRATOS initiative

[1]Department of Statistics, TU Dortmund University
[2]Division of Biostatistics, German Cancer Research Center
[3]Faculty of Liberal Arts and Sciences, Technical University of Applied Sciences Augsburg
[4]Department of Statistics and Actuarial Science, University of Waterloo

A Numbers of Warning and Error Messages

	Ridge	LASSO	SVM	KNN	Random Forest
Distribution: Standard Normal	0	1	0	0	0
OGM scaled by: 0.5	8	12	0	0	0
OGM scaled by: 2	11	11	0	0	0
Scale: 0.25	1	0	0	0	0
Shift: -0.5	0	1	0	0	0
True Scenario	0	5	0	0	0

Table A.1: Number of iterations with warning messages per scenario and classifier for $p = 2$. Scenarios with no warning messages are not displayed.

*Corresponding author, e-mail: stolte@statistik.tu-dortmund.de

	Ridge	LASSO	SVM	KNN	Random Forest
Correlation: -0.1	0	7	0	0	0
Correlation: -0.2	0	11	0	0	0
Correlation: 0	0	4	0	0	0
Correlation: 0.1	0	6	0	0	0
Correlation: 0.2	0	11	0	0	0
Distribution: Standard Normal	95	95	0	0	19
OGM scaled by: 0	0	1	0	0	0
OGM scaled by: 0.5	1	60	0	0	0
OGM scaled by: 2	4	5	0	0	0
Scale: 0.25	7	7	0	0	100
Scale: 0.5	91	91	0	0	23
Scale: 0.75	7	49	0	0	0
Scale: 1.33	0	5	0	0	0
Scale: 2	0	7	0	0	0
Scale: 4	19	20	0	0	0
Shift: -0.125	0	9	0	0	0
Shift: -0.25	0	10	0	0	0
Shift: -0.5	0	11	0	0	0
Shift: 0.125	0	9	0	0	0
Shift: 0.25	0	6	0	0	0
Shift: 0.5	0	5	0	0	0
True Scenario	12	47	0	0	0

Table A.2: Number of iterations with warning messages per scenario and classifier for $p = 10$. Scenarios with no warning messages are not displayed.

	Ridge	LASSO	SVM	KNN	Random Forest
Distribution: Standard Normal	62	59	19	0	0
Scale: 0.25	100	100	100	0	0
Scale: 0.5	63	59	23	0	0

Table A.3: Number of iterations with error messages per scenario and classifier for $p = 10$. Scenarios with no error messages are not displayed.

	Ridge	LASSO	SVM	KNN	Random Forest
Correlation: -0.1	0	1	0	0	0
Distribution: Standard Normal	19	20	0	0	0
OGM scaled by: 0	1	3	0	0	0
OGM scaled by: 0.5	3	2	0	0	0
Scale: 0.25	0	0	0	0	100
Scale: 0.5	0	0	0	0	100
Scale: 0.75	88	86	0	0	2
Scale: 1.33	15	18	0	0	0
Scale: 2	93	92	0	0	10
Scale: 4	42	42	0	0	83
Shift: -0.125	35	35	0	0	0
Shift: -0.25	95	94	0	0	16
Shift: -0.5	11	11	0	0	99
Shift: 0.125	2	2	0	0	0
Shift: 0.25	91	90	0	0	0
Shift: 0.5	35	35	0	0	96
True Scenario	3	2	0	0	0

Table A.4: Number of iterations with warning messages per scenario and classifier for $p = 50$. Scenarios with no warning messages are not displayed.

	Ridge	LASSO	SVM	KNN	Random Forest
Distribution: Standard Normal	0	1	0	0	0
Scale: 0.25	100	100	100	0	0
Scale: 0.5	100	100	100	0	0
Scale: 0.75	16	21	2	0	0
Scale: 2	53	45	10	0	0
Scale: 4	99	99	83	0	0
Shift: -0.125	2	1	0	0	0
Shift: -0.25	53	48	16	0	0
Shift: -0.5	100	100	99	0	0
Shift: 0.25	4	6	0	0	0
Shift: 0.5	100	100	96	0	0

Table A.5: Number of iterations with error messages per scenario and classifier for $p = 50$. Scenarios with no error messages are not displayed.

	Ridge	LASSO	SVM	KNN	Random Forest
Distribution: Standard Normal	0	1	0	0	0
OGM scaled by: 0.5	5	3	0	0	0
Scale: 0.25	0	0	0	0	100
Scale: 0.5	81	81	0	0	38
Scale: 0.75	5	4	0	0	0
Scale: 1.33	4	6	0	0	0
Scale: 2	60	64	0	0	0
Scale: 4	95	95	0	0	6
Shift: -0.125	0	1	0	0	0
Shift: -0.25	34	31	0	0	1
Shift: -0.5	89	89	0	0	34
Shift: 0.125	5	4	0	0	0
Shift: 0.25	60	59	0	0	0
Shift: 0.5	78	78	0	0	38
True Scenario	2	1	0	0	0

Table A.6: Number of iterations with warning messages per scenario and classifier for $p = 150$. Scenarios with no warning messages are not displayed.

	Ridge	LASSO	SVM	KNN	Random Forest
Scale: 0.25	100	100	100	0	0
Scale: 0.5	79	76	38	0	0
Scale: 2	3	4	0	0	0
Scale: 4	33	33	6	0	0
Shift: -0.25	1	1	1	0	0
Shift: -0.5	73	73	34	0	0
Shift: 0.25	1	0	0	0	0
Shift: 0.5	83	82	38	0	0

Table A.7: Number of iterations with error messages per scenario and classifier for $p = 150$. Scenarios with no error messages are not displayed.

B Resampling types

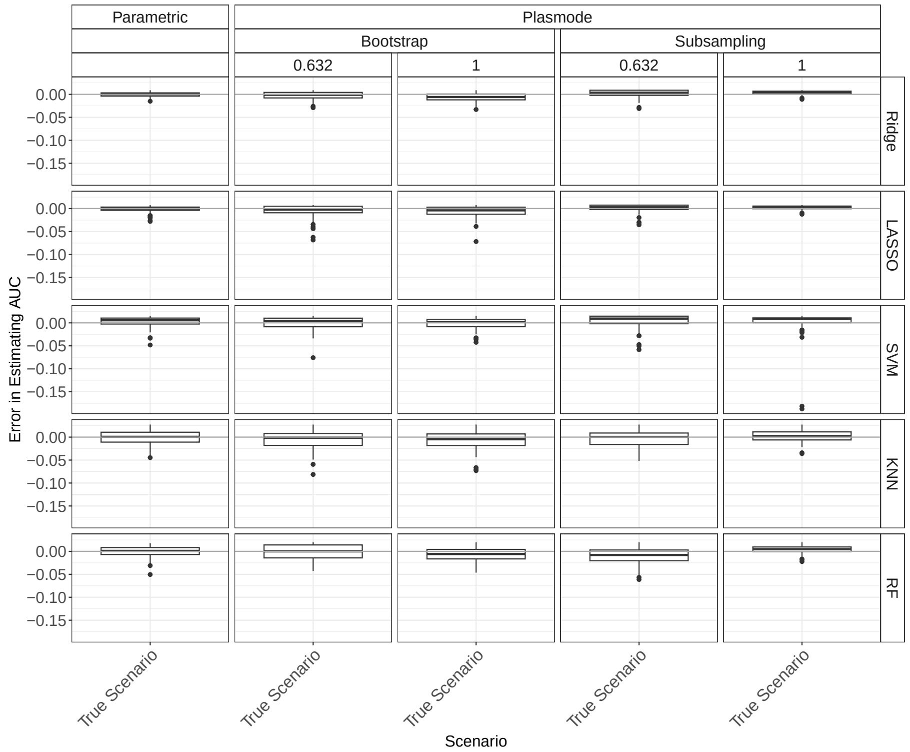

Fig B.1: Errors in the estimation of AUC in 100 iterations of a classification method comparison study per classifier for different simulation approaches under the true scenario for $p = 2$.

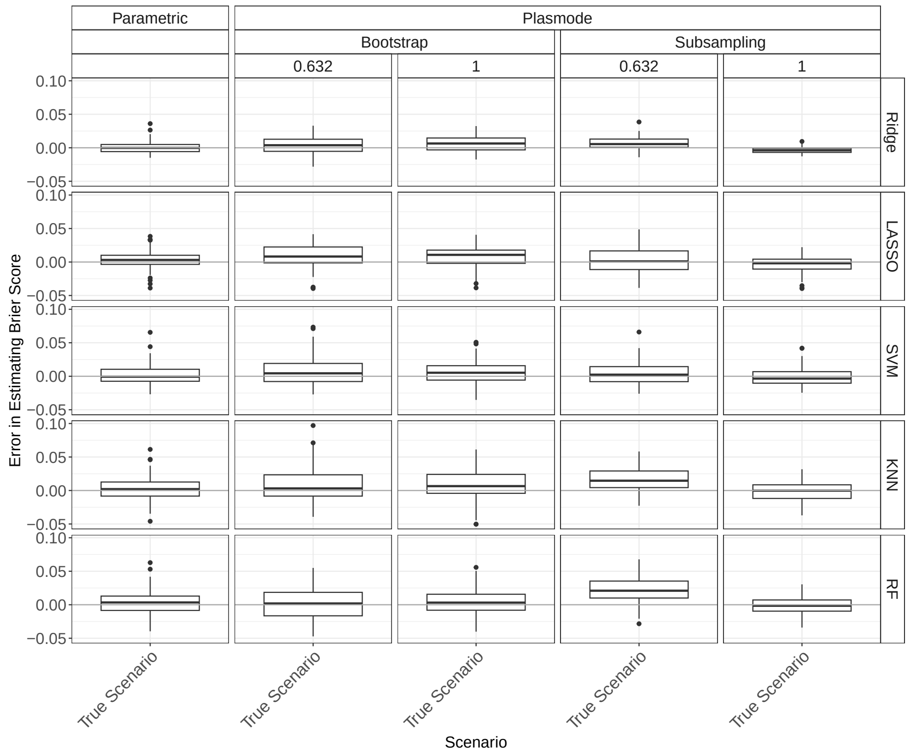

Fig B.2: Errors in the estimation of the Brier score in 100 iterations of a classification method comparison study per classifier for different simulation approaches under the true scenario for $p = 2$.

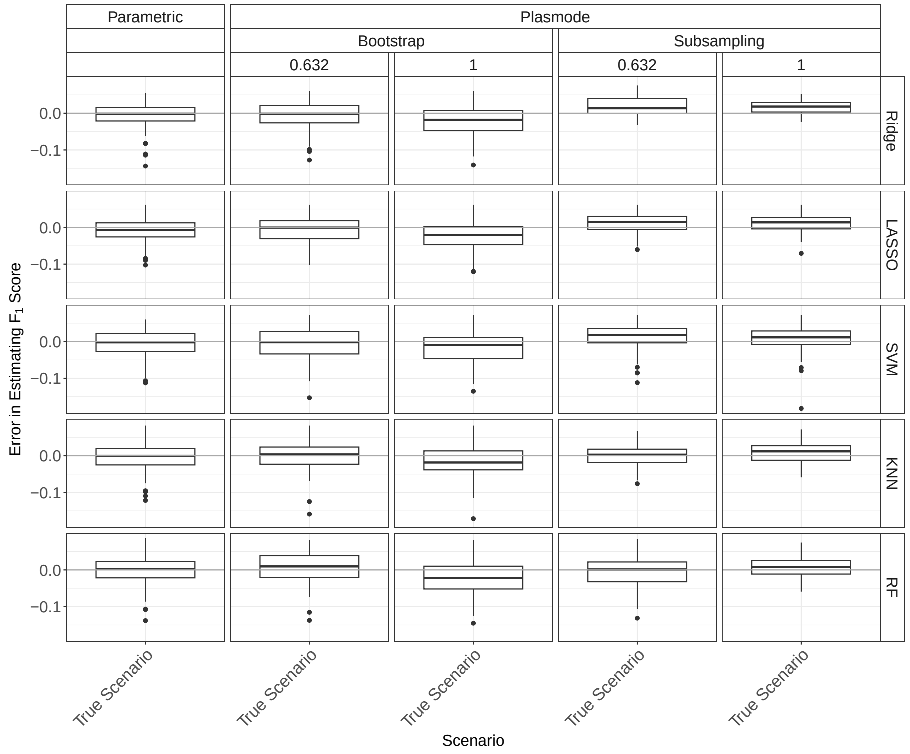

Fig B.3: Errors in the estimation of the F1 score in 100 iterations of a classification method comparison study per classifier for different simulation approaches under the true scenario for $p = 2$.

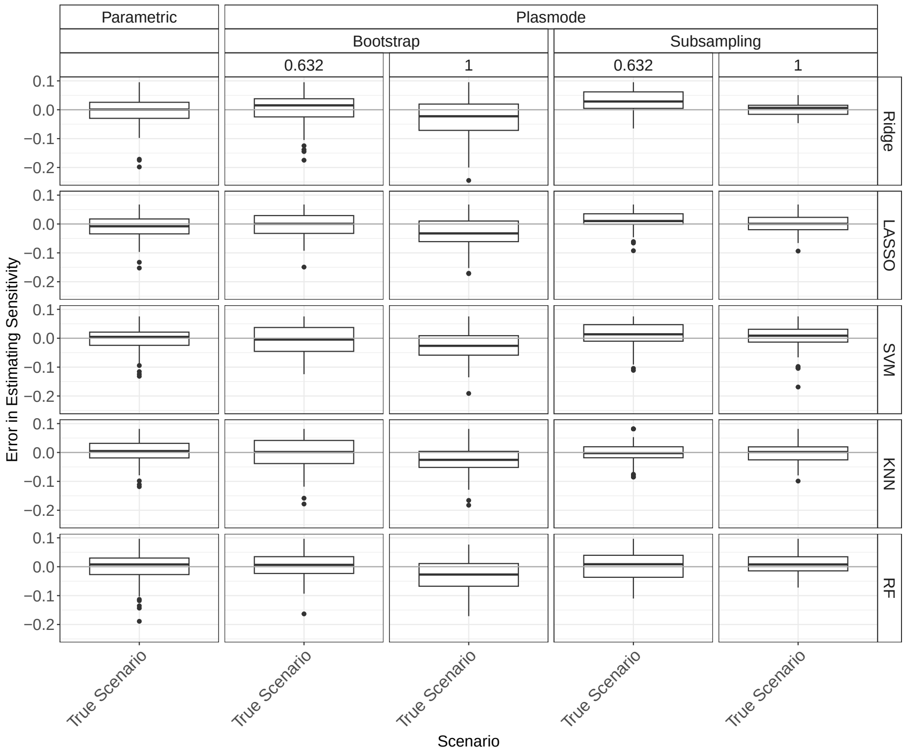

Fig B.4: Errors in the estimation of sensitivity in 100 iterations of a classification method comparison study per classifier for different simulation approaches under the true scenario for $p = 2$.

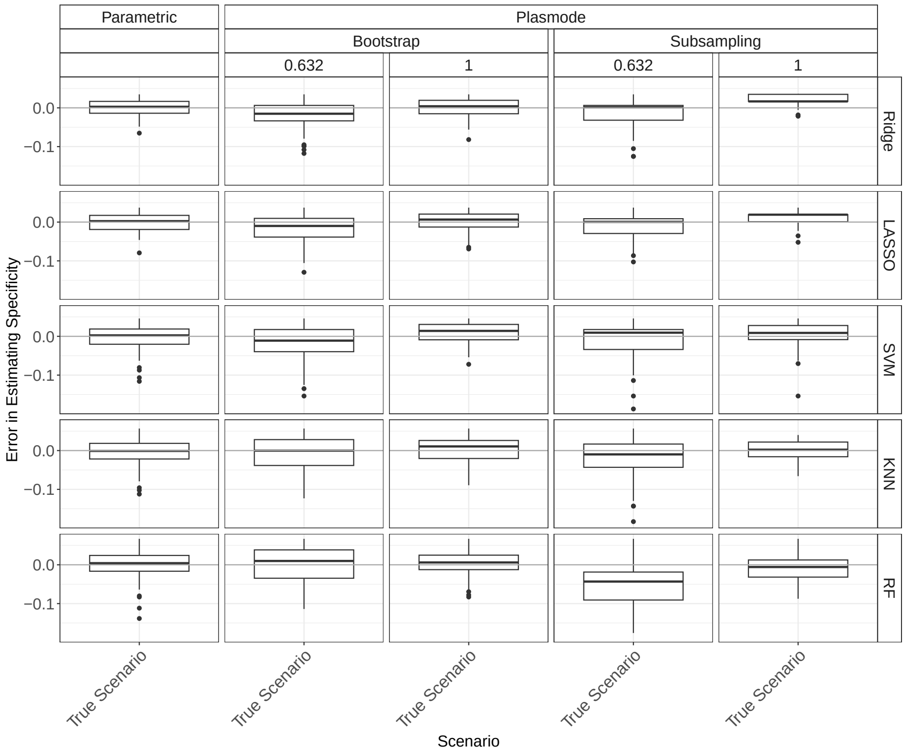

Fig B.5: Errors in the estimation of specificity in 100 iterations of a classification method comparison study per classifier for different simulation approaches under the true scenario for $p = 2$.

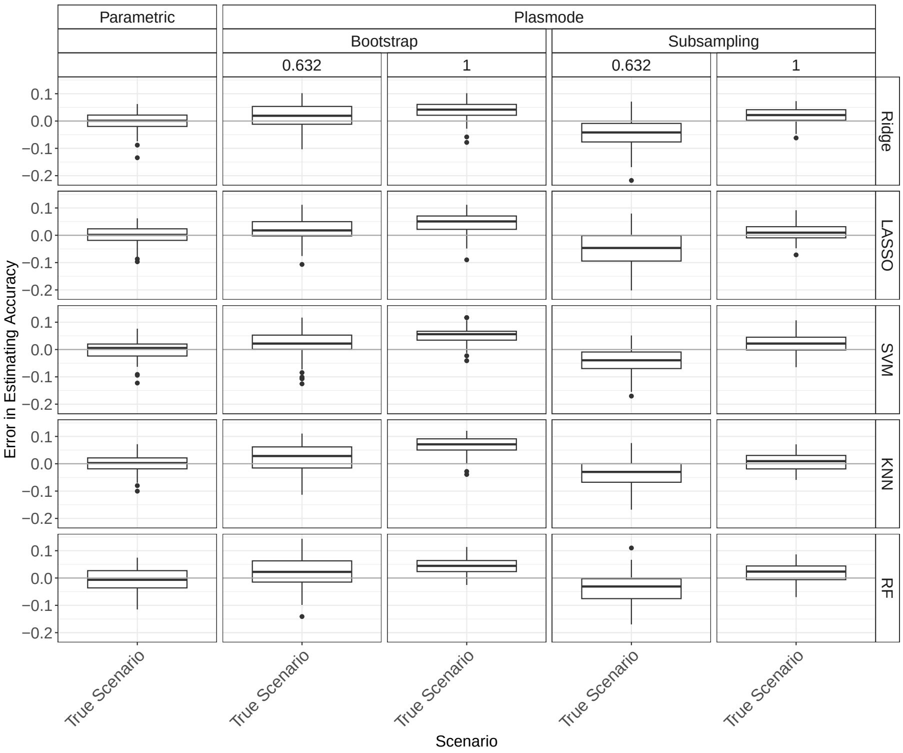

Fig B.6: Errors in the estimation of accuracy in 100 iterations of a classification method comparison study per classifier for different simulation approaches under the true scenario for $p = 10$.

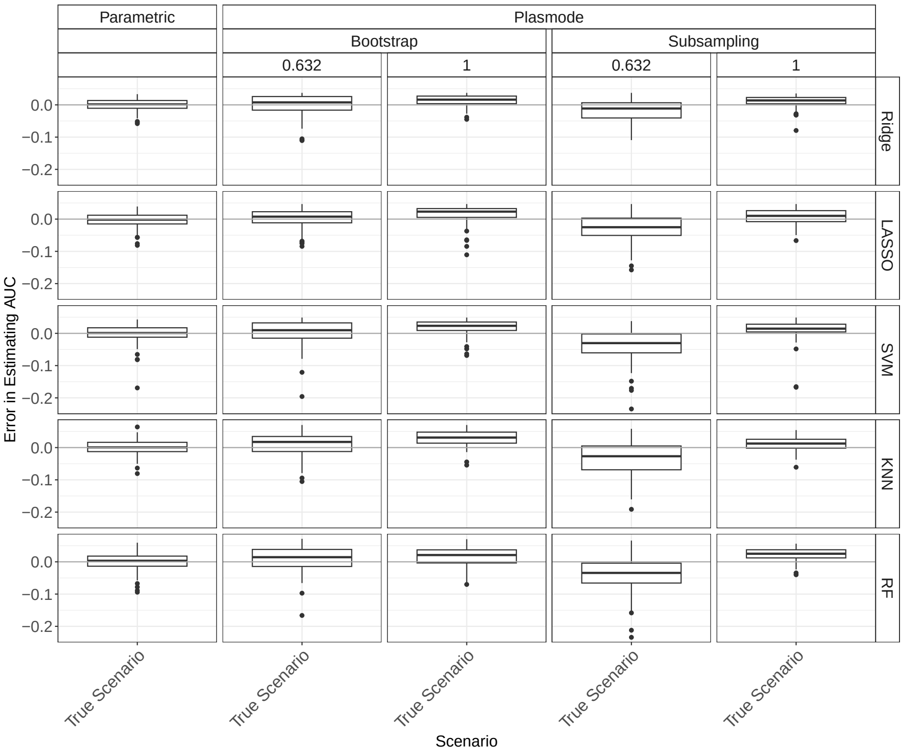

Fig B.7: Errors in the estimation of AUC in 100 iterations of a classification method comparison study per classifier for different simulation approaches under the true scenario for $p = 10$.

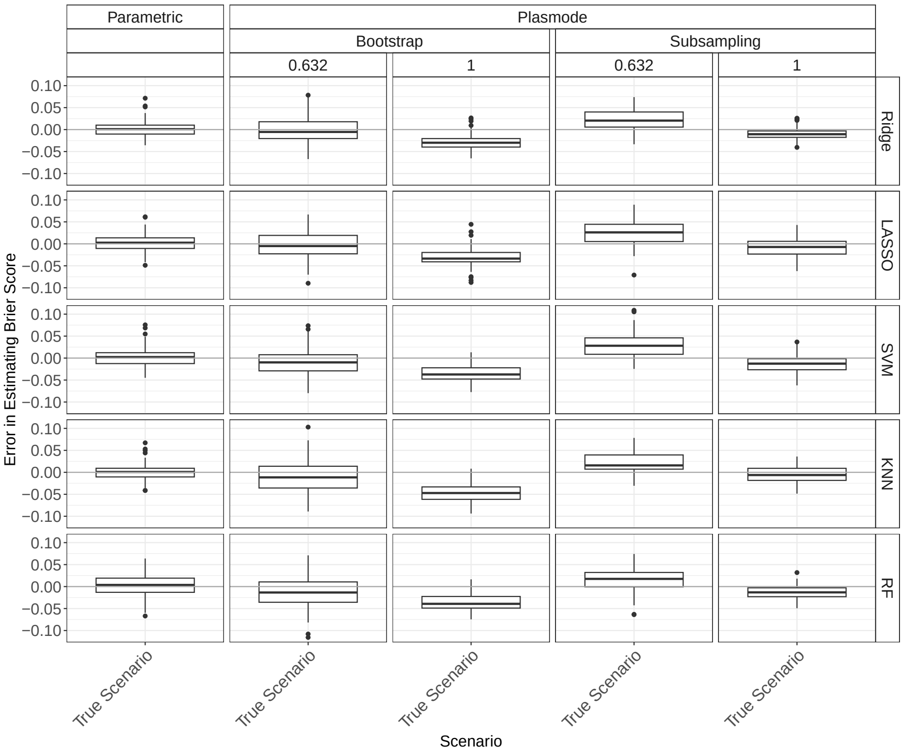

Fig B.8: Errors in the estimation of the Brier score in 100 iterations of a classification method comparison study per classifier for different simulation approaches under the true scenario for $p = 10$.

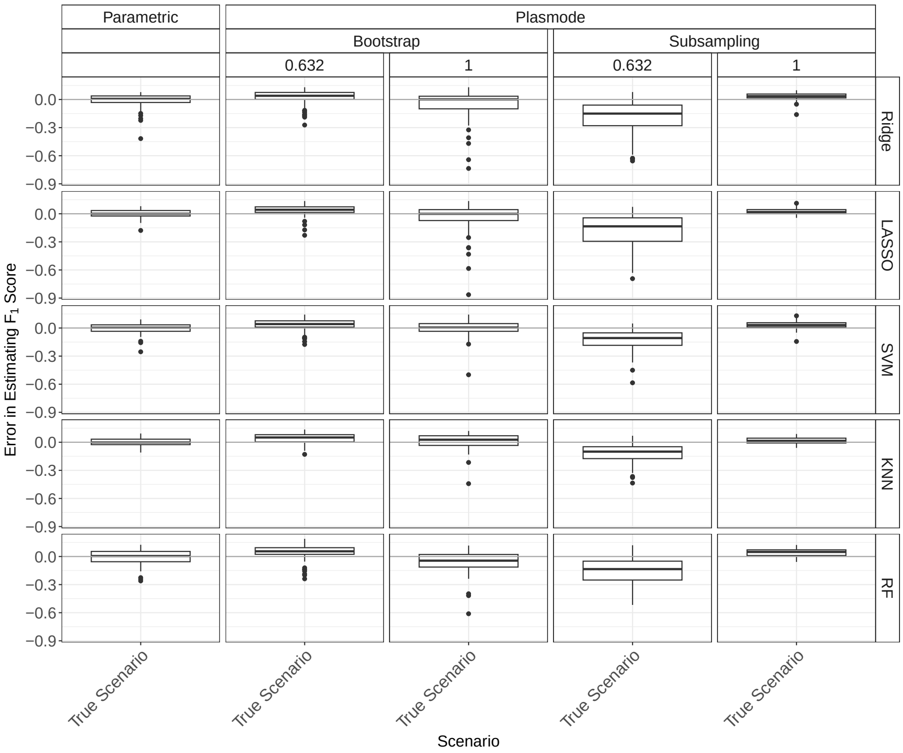

Fig B.9: Errors in the estimation of the F_1-score in 100 iterations of a classification method comparison study per classifier for different simulation approaches under the true scenario for $p = 10$.

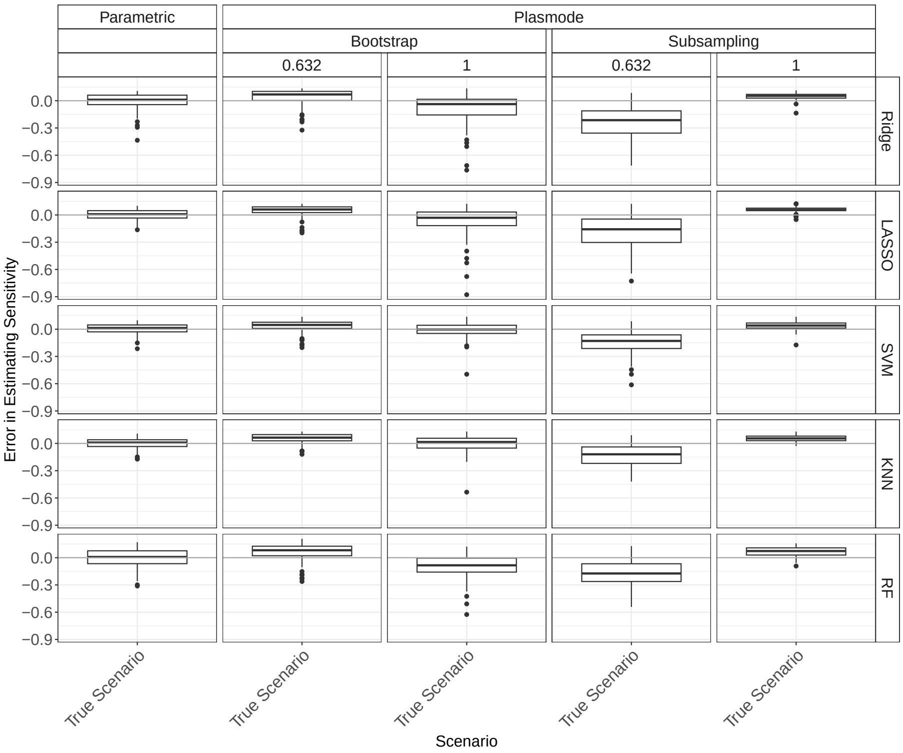

Fig B.10: Errors in the estimation of sensitivity in 100 iterations of a classification method comparison study per classifier for different simulation approaches under the true scenario for $p = 10$.

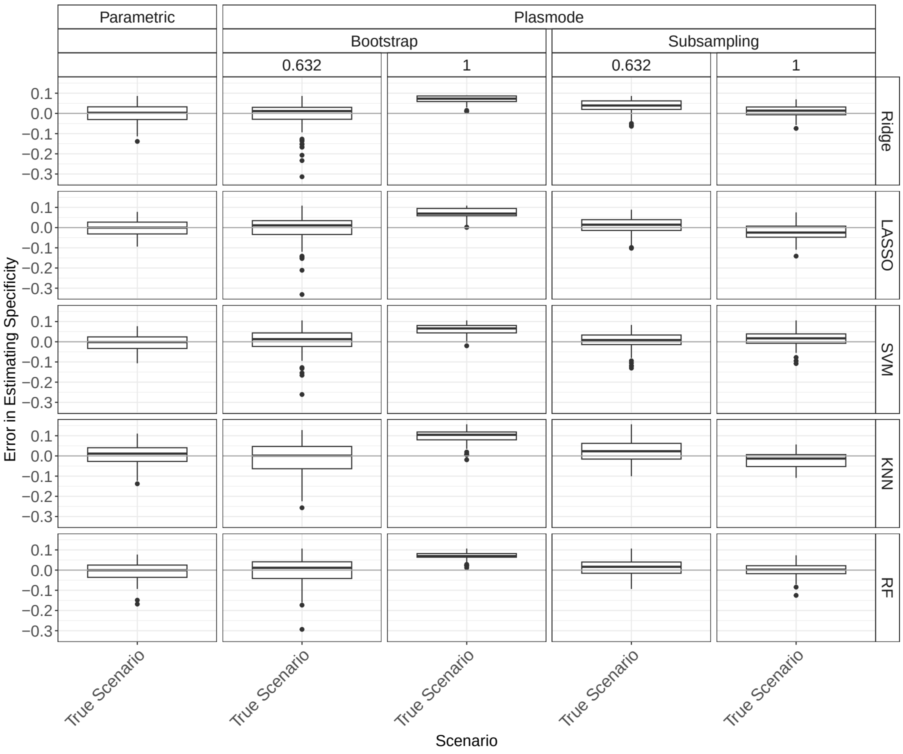

Fig B.11: Errors in the estimation of specificity in 100 iterations of a classification method comparison study per classifier for different simulation approaches under the true scenario for $p = 10$.

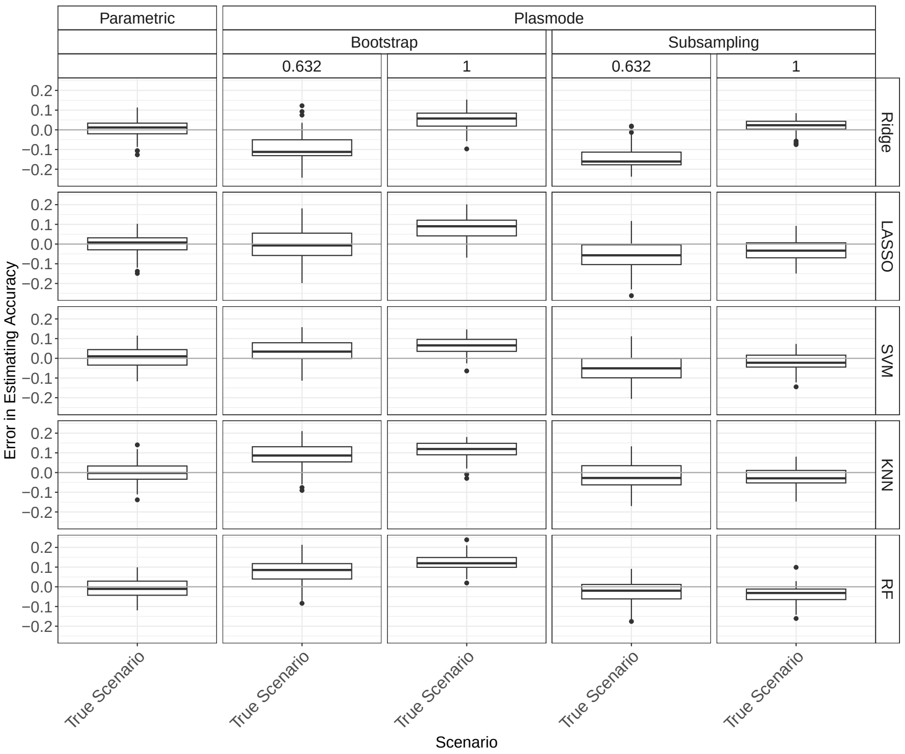

Fig B.12: Errors in the estimation of accuracy in 100 iterations of a classification method comparison study per classifier for different simulation approaches under the true scenario for $p = 50$.

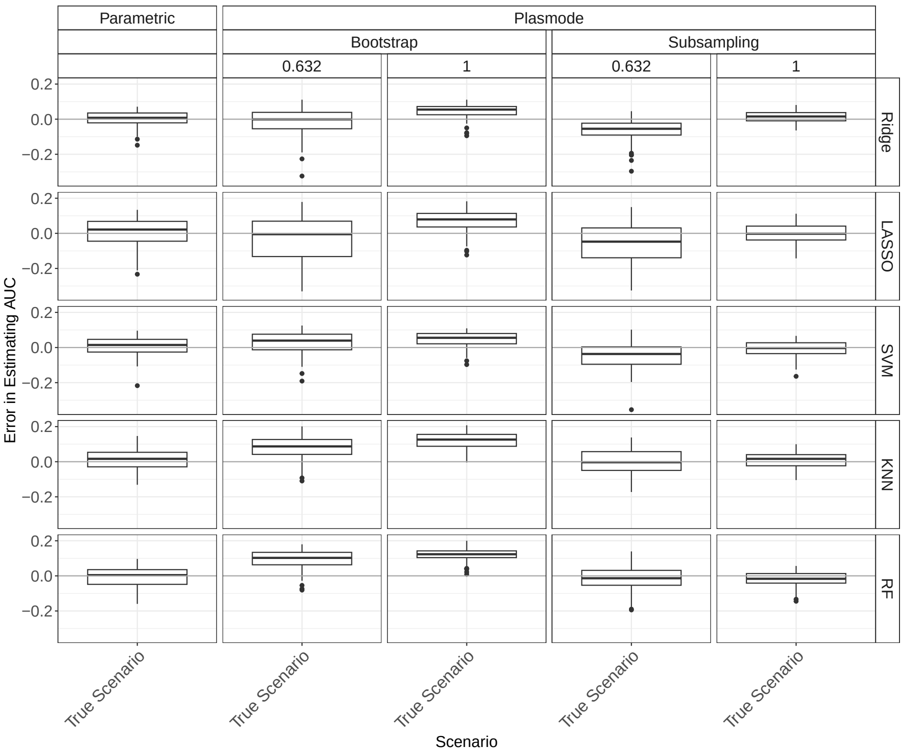

Fig B.13: Errors in the estimation of AUC in 100 iterations of a classification method comparison study per classifier for different simulation approaches under the true scenario for $p = 50$.

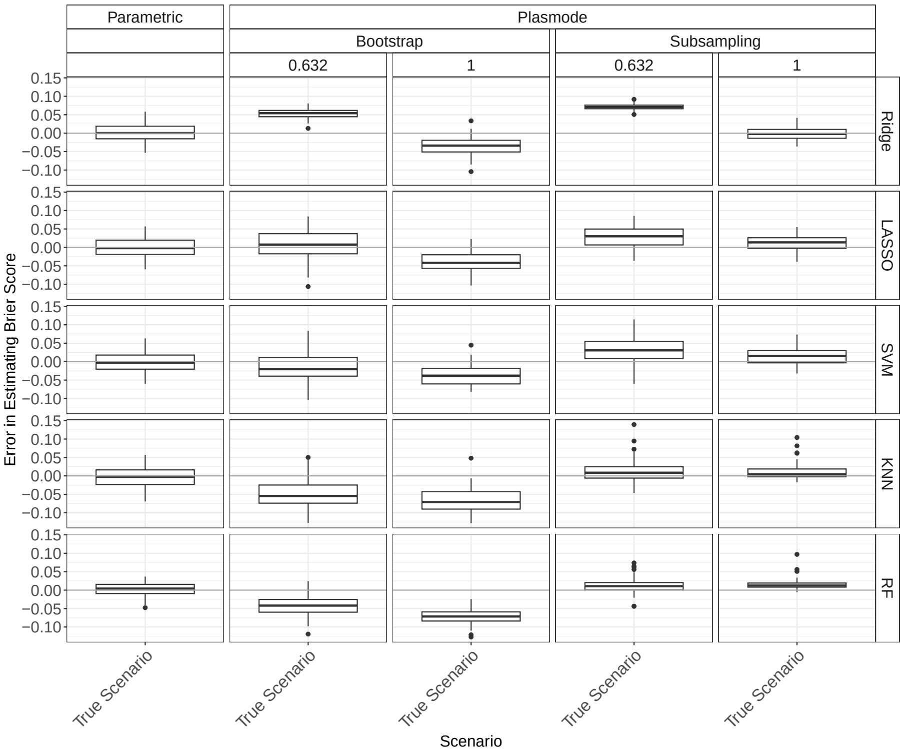

Fig B.14: Errors in the estimation of the Brier score in 100 iterations of a classification method comparison study per classifier for different simulation approaches under the true scenario for $p = 50$.

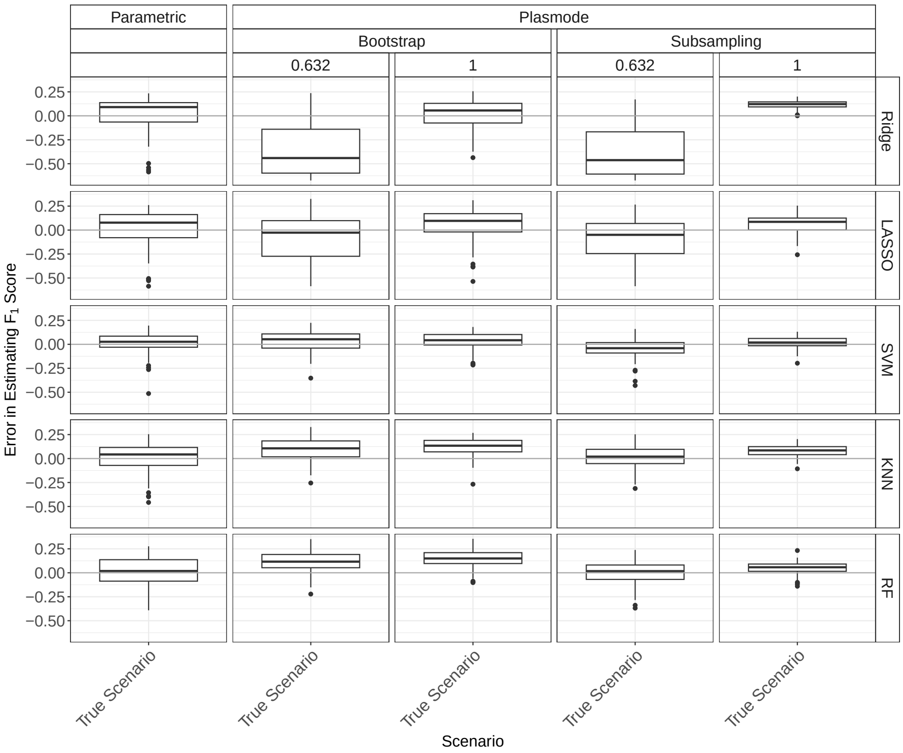

Fig B.15: Errors in the estimation of the F_1-score in 100 iterations of a classification method comparison study per classifier for different simulation approaches under the true scenario for $p = 50$.

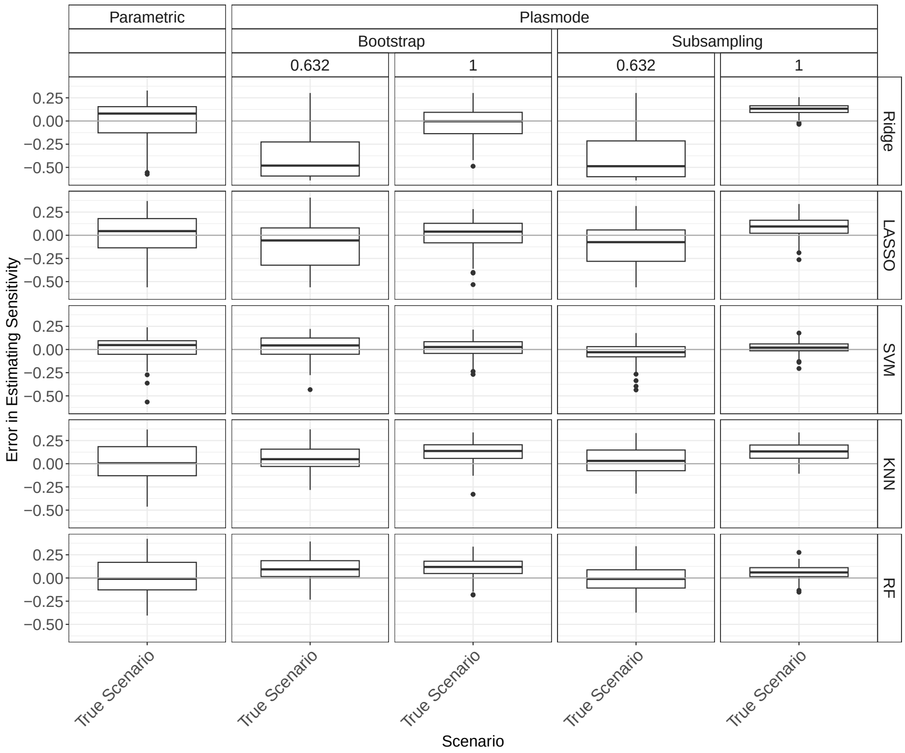

Fig B.16: Errors in the estimation of sensitivity in 100 iterations of a classification method comparison study per classifier for different simulation approaches under the true scenario for $p = 50$.

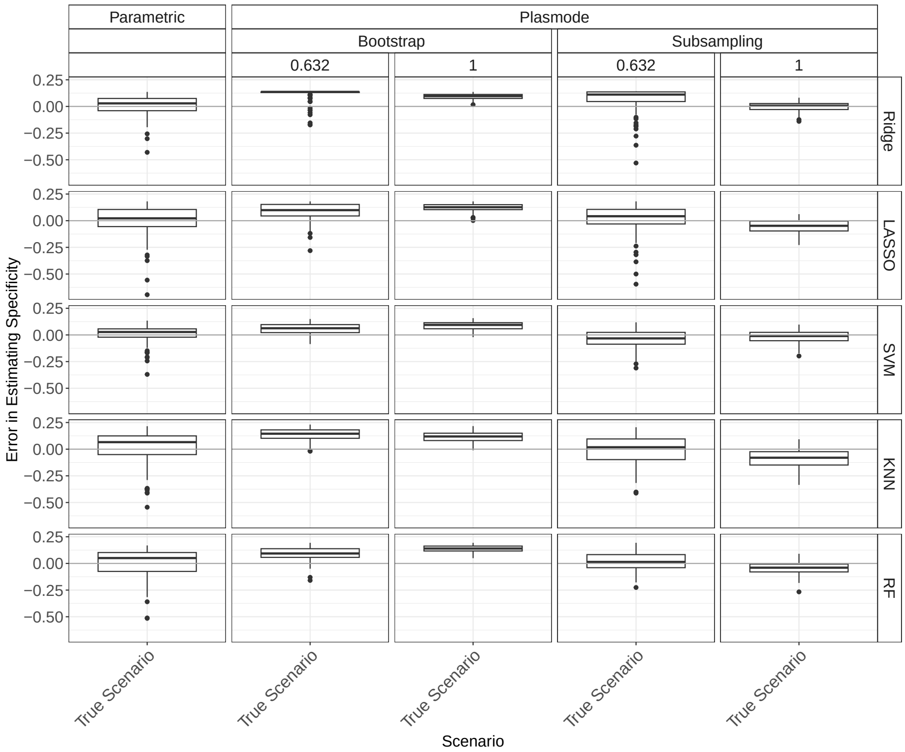

Fig B.17: Errors in the estimation of specificity in 100 iterations of a classification method comparison study per classifier for different simulation approaches under the true scenario for $p = 50$.

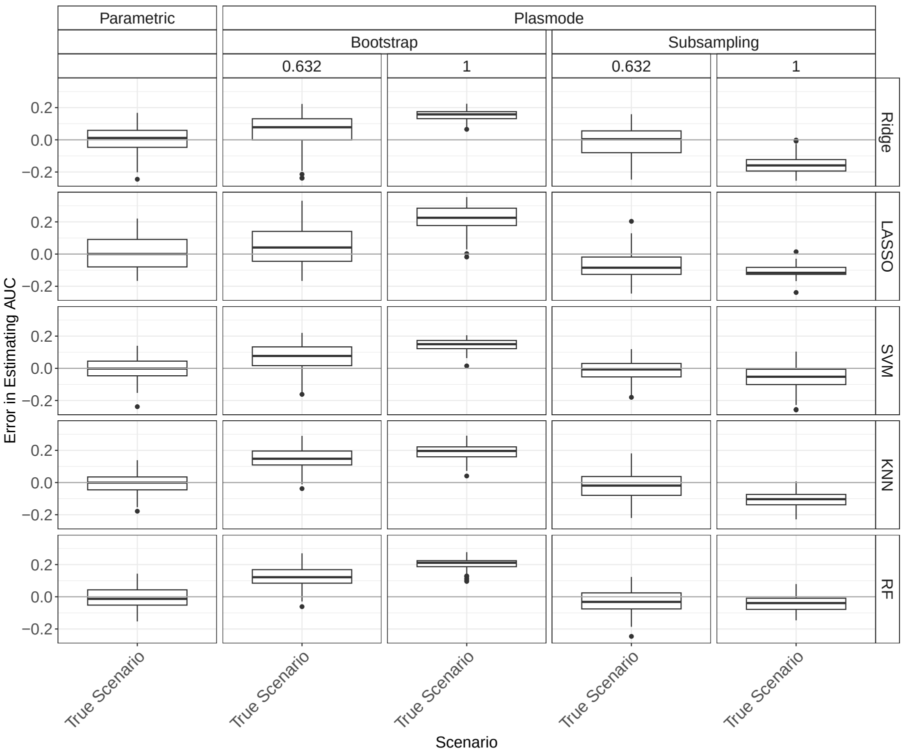

Fig B.18: Errors in the estimation of AUC in 100 iterations of a classification method comparison study per classifier for different simulation approaches under the true scenario for $p = 150$.

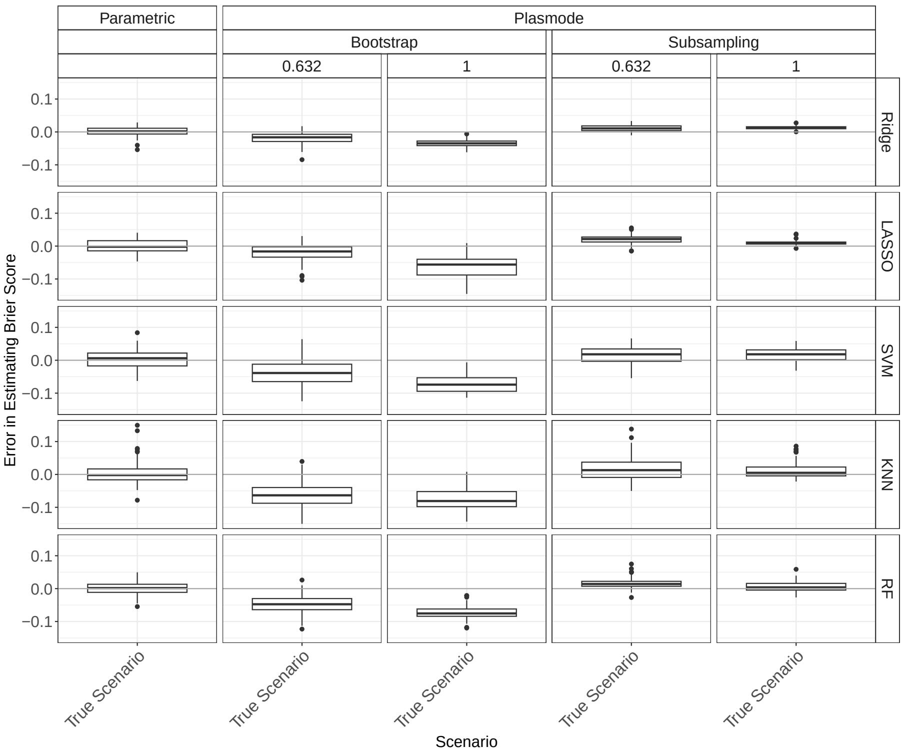

Fig B.19: Errors in the estimation of the Brier score in 100 iterations of a classification method comparison study per classifier for different simulation approaches under the true scenario for $p = 150$.

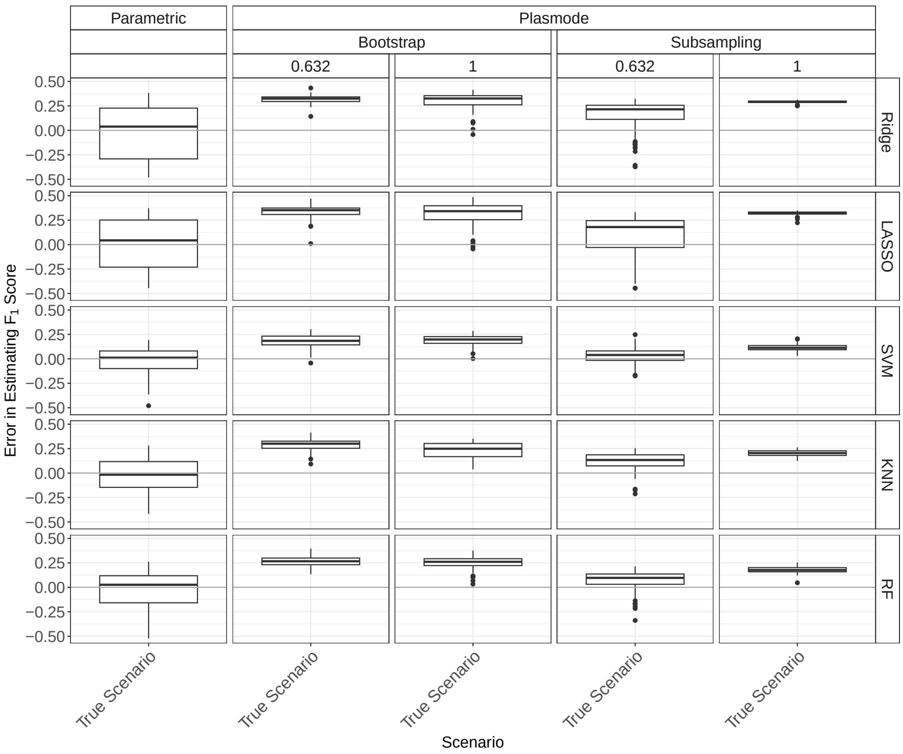

Fig B.20: Errors in the estimation of the F_1-score in 100 iterations of a classification method comparison study per classifier for different simulation approaches under the true scenario for $p = 150$.

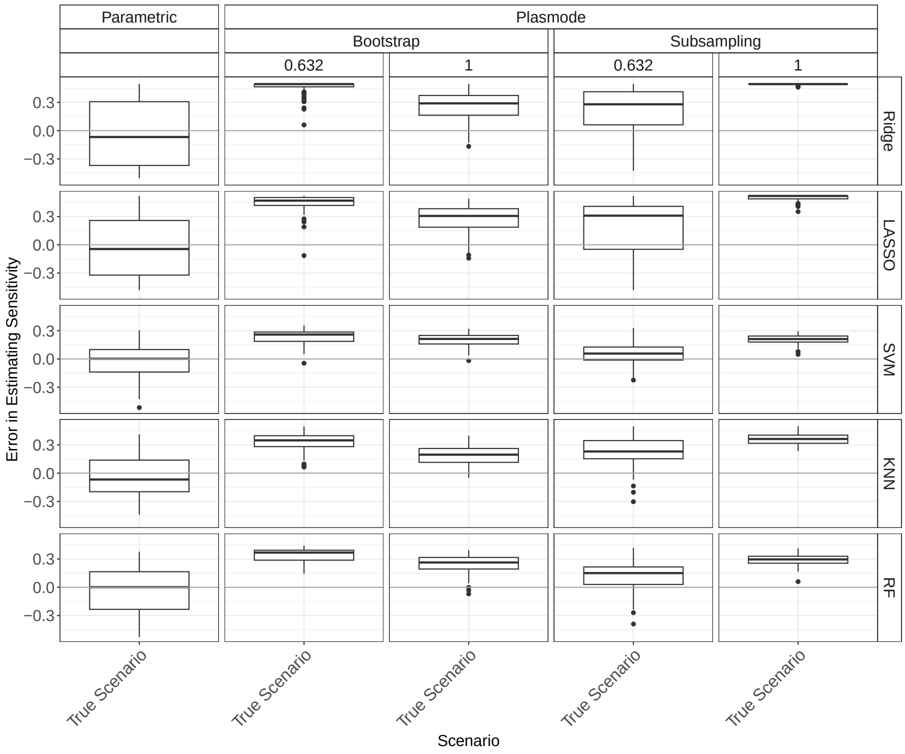

Fig B.21: Errors in the estimation of sensitivity in 100 iterations of a classification method comparison study per classifier for different simulation approaches under the true scenario for $p = 150$.

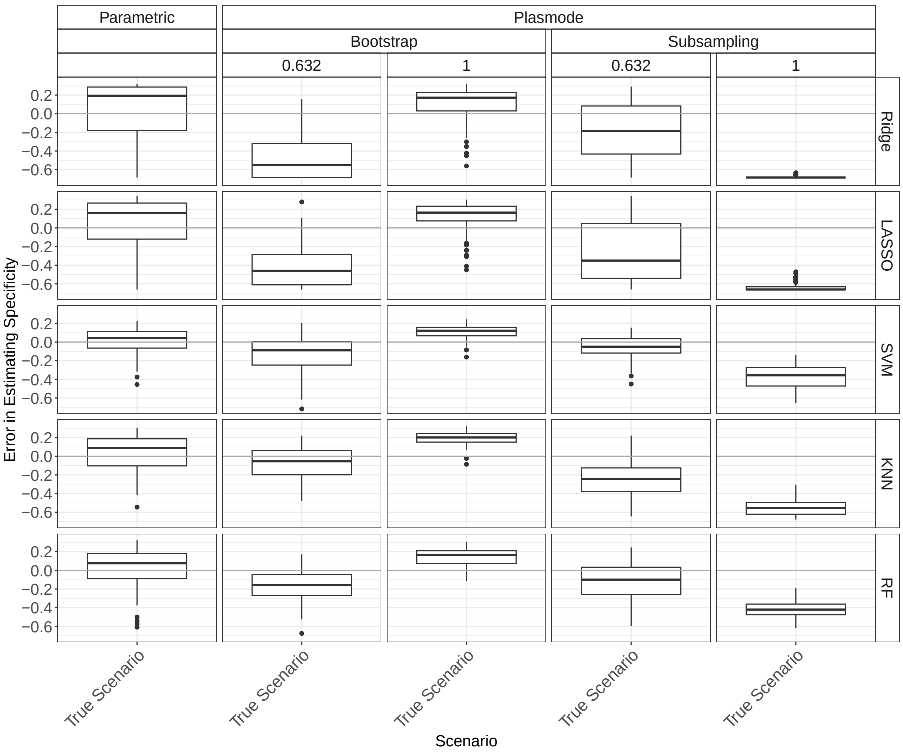

Fig B.22: Errors in the estimation of specificity in 100 iterations of a classification method comparison study per classifier for different simulation approaches with misspecified shift for parametric simulation for $p = 150$.

C Shift

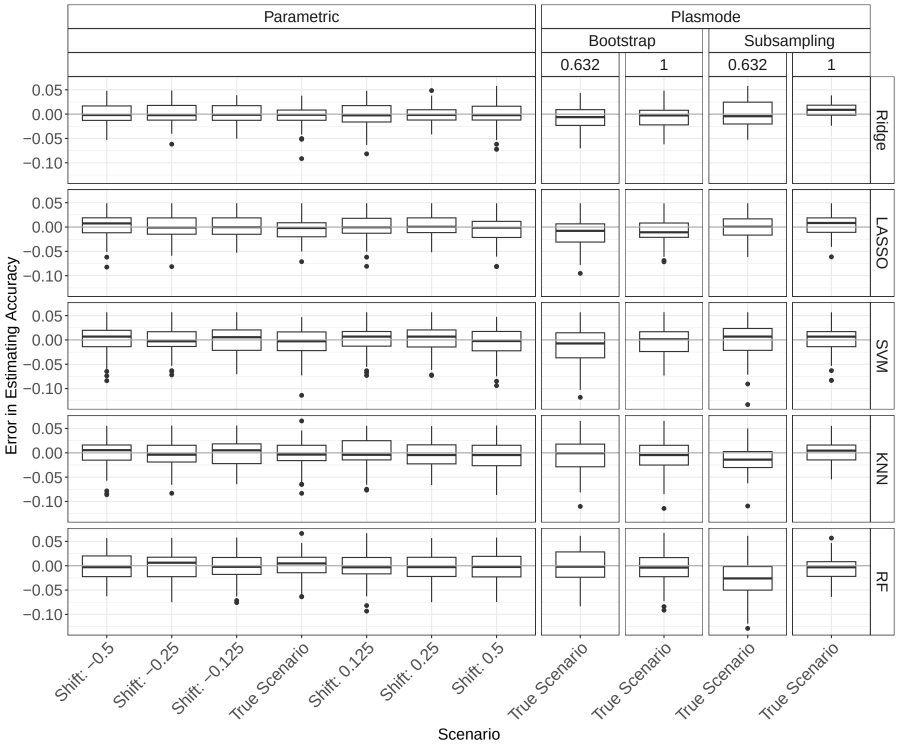

Fig C.1: Errors in the estimation of accuracy in 100 iterations of a classification method comparison study per classifier for different simulation approaches with misspecified shift for parametric simulation for $p = 2$.

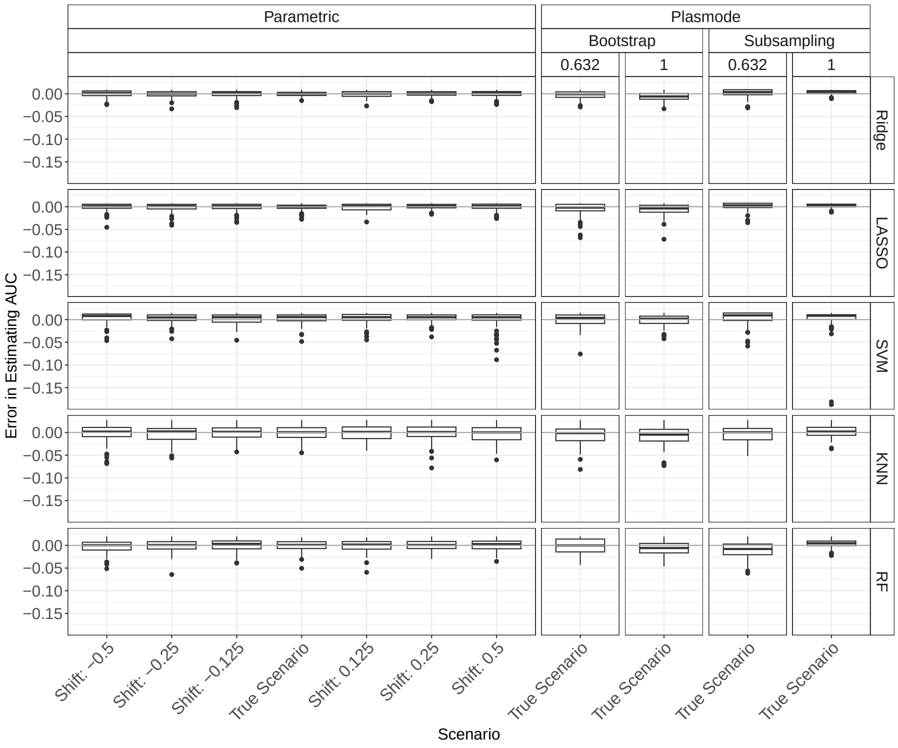

Fig C.2: Errors in the estimation of AUC in 100 iterations of a classification method comparison study per classifier for different simulation approaches with misspecified shift for parametric simulation for $p = 2$.

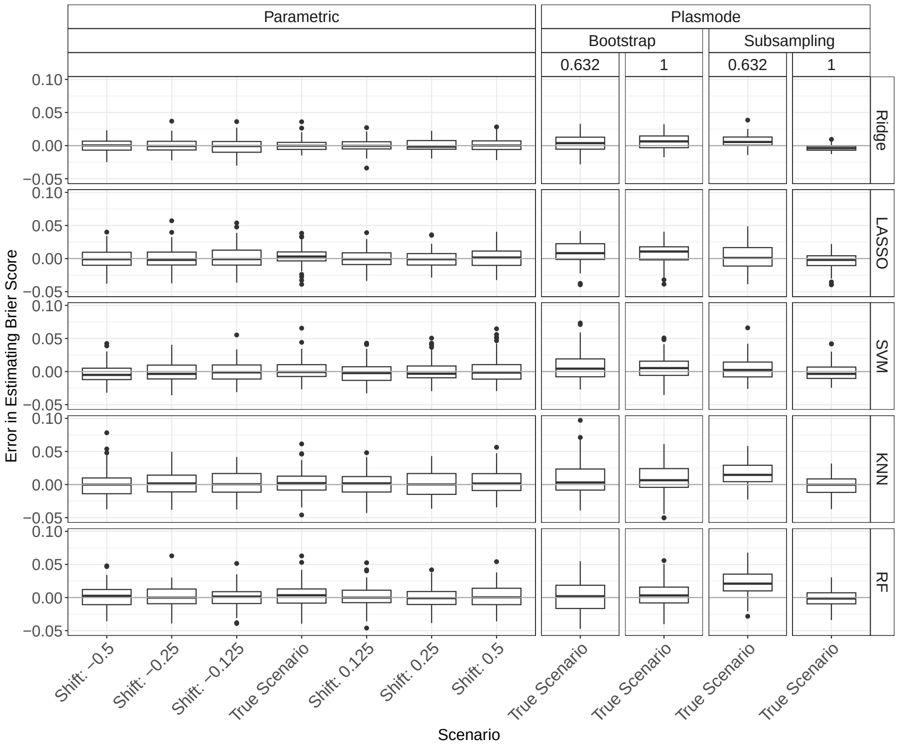

Fig C.3: Errors in the estimation of the Brier score in 100 iterations of a classification method comparison study per classifier for different simulation approaches with misspecified shift for parametric simulation for $p = 2$.

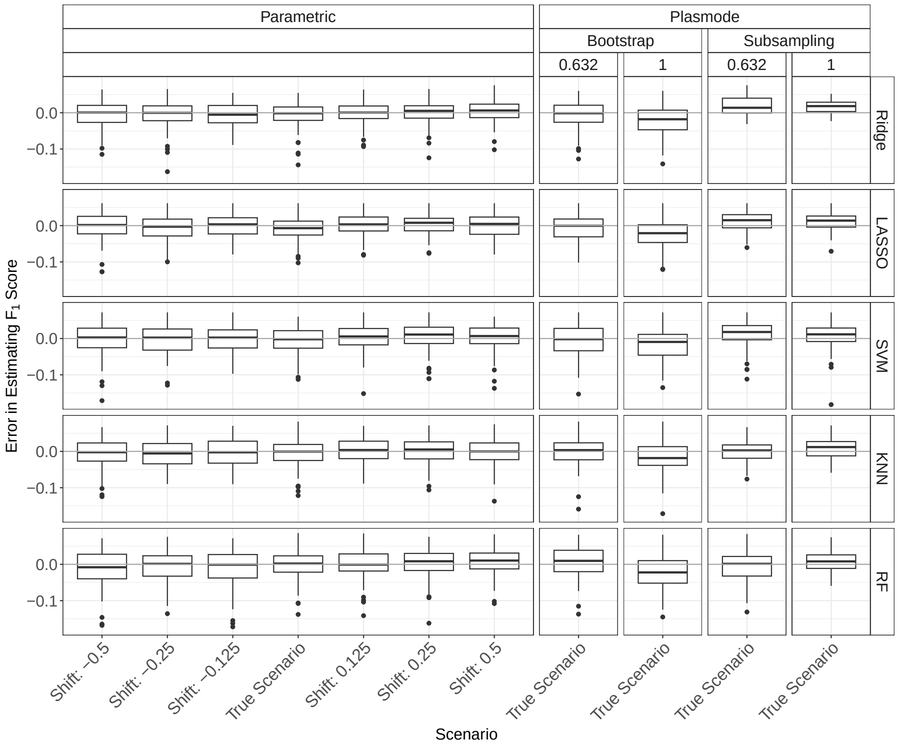

Fig C.4: Errors in the estimation of the F1 score in 100 iterations of a classification method comparison study per classifier for different simulation approaches with misspecified shift for parametric simulation for $p = 2$.

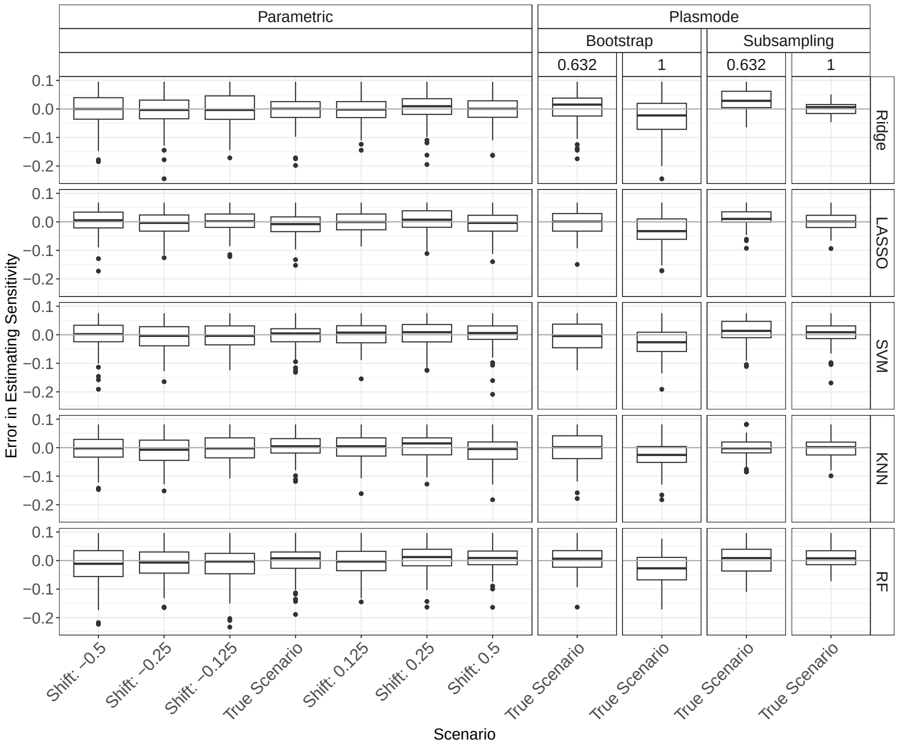

Fig C.5: Errors in the estimation of sensitivity in 100 iterations of a classification method comparison study per classifier for different simulation approaches with misspecified shift for parametric simulation for $p = 2$.

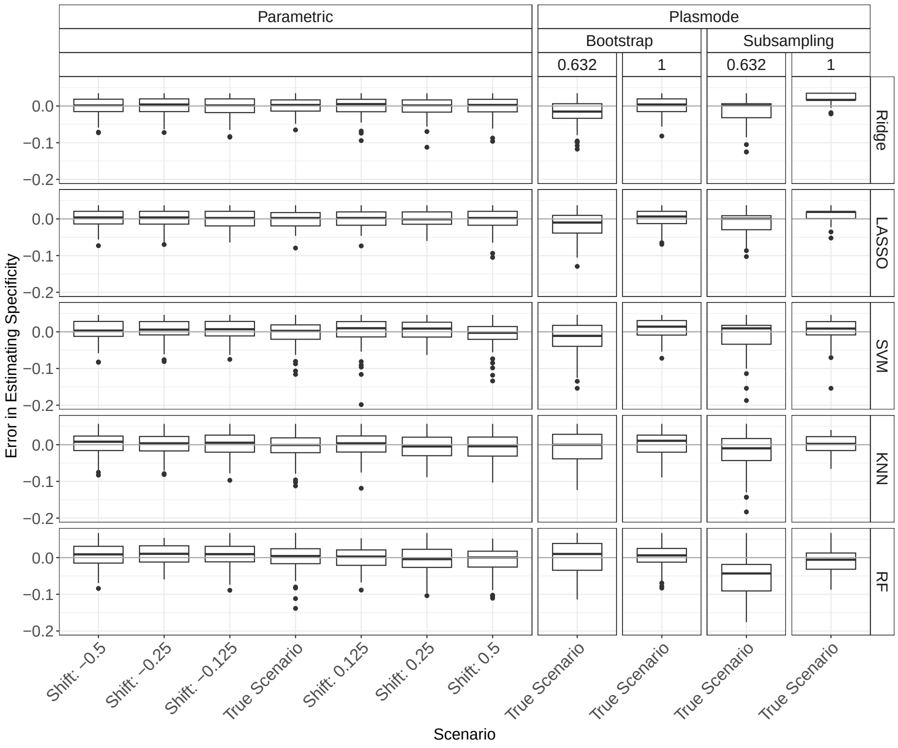

Fig C.6: Errors in the estimation of specificity in 100 iterations of a classification method comparison study per classifier for different simulation approaches with misspecified shift for parametric simulation for $p = 2$.

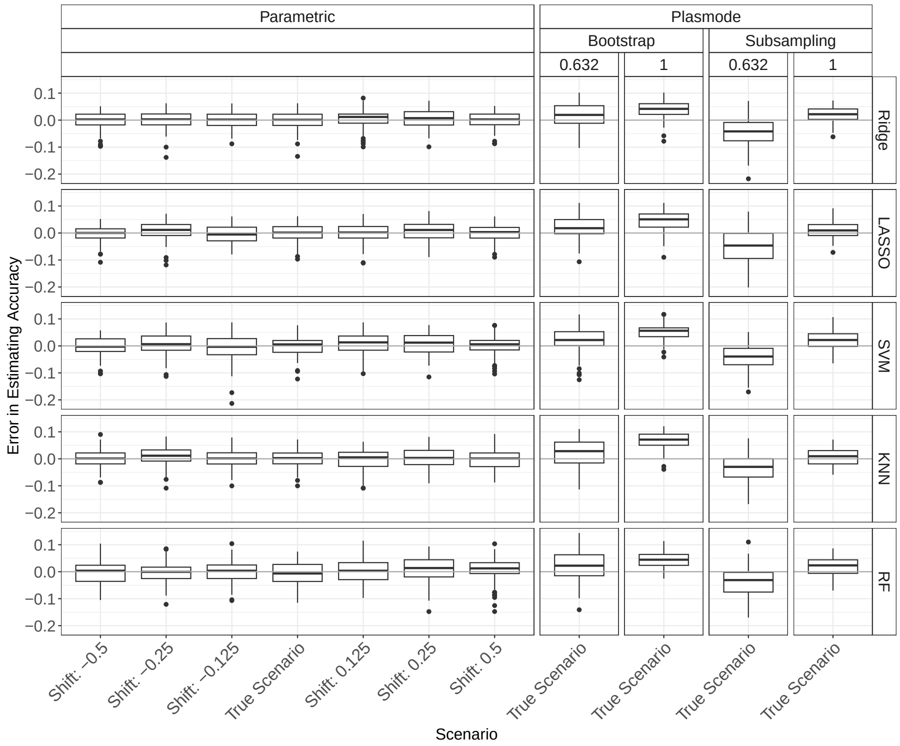

Fig C.7: Errors in the estimation of accuracy in 100 iterations of a classification method comparison study per classifier for different simulation approaches with misspecified shift for parametric simulation for $p = 10$.

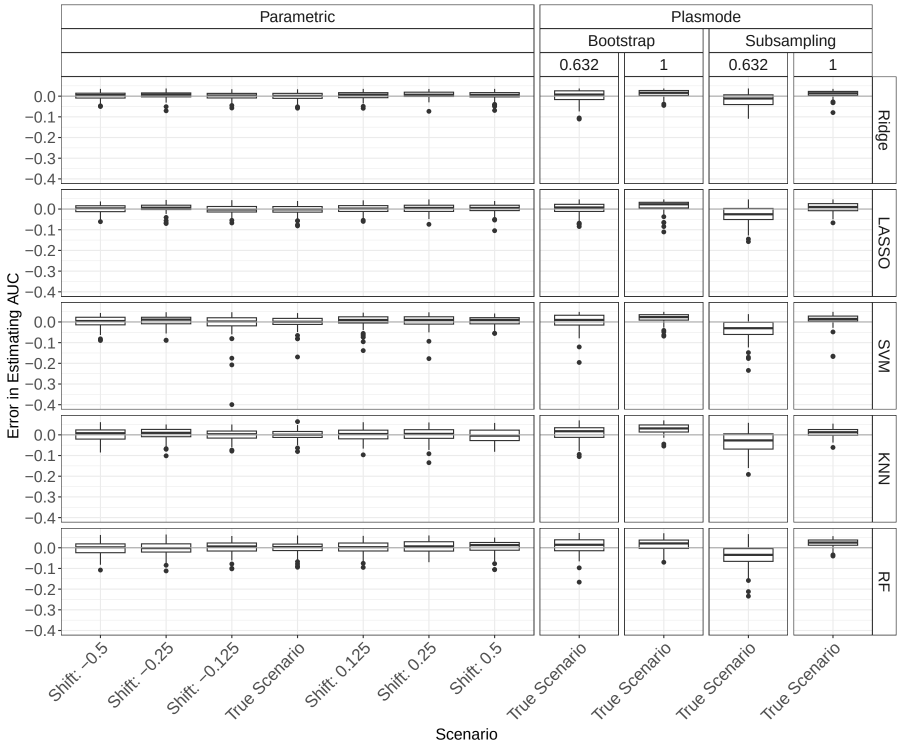

Fig C.8: Errors in the estimation of AUC in 100 iterations of a classification method comparison study per classifier for different simulation approaches with misspecified shift for parametric simulation for $p = 10$.

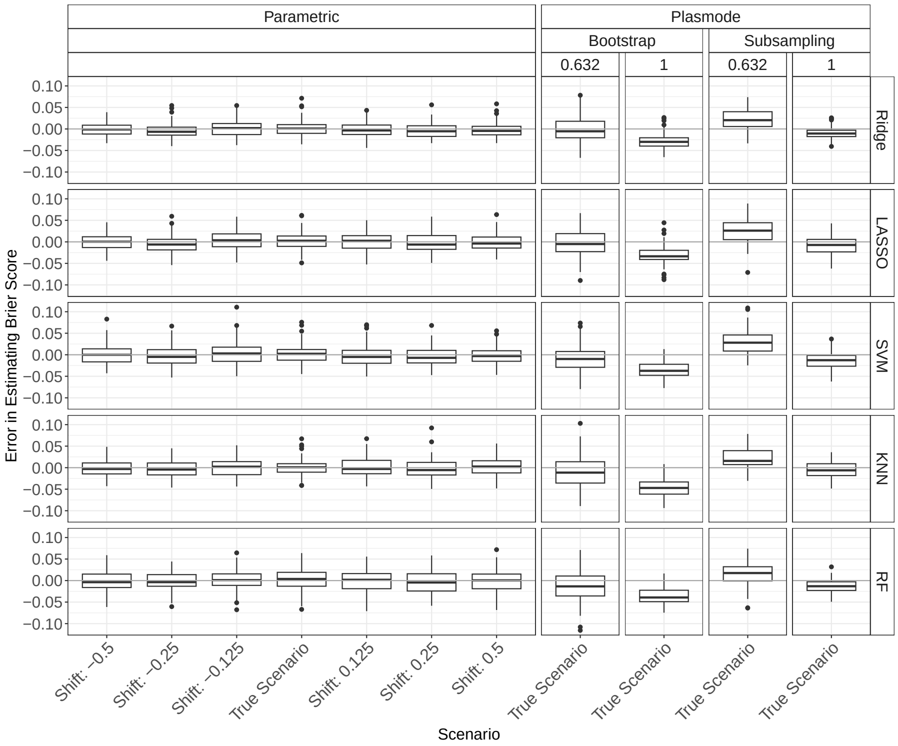

Fig C.9: Errors in the estimation of the Brier score in 100 iterations of a classification method comparison study per classifier for different simulation approaches with misspecified shift for parametric simulation for $p = 10$.

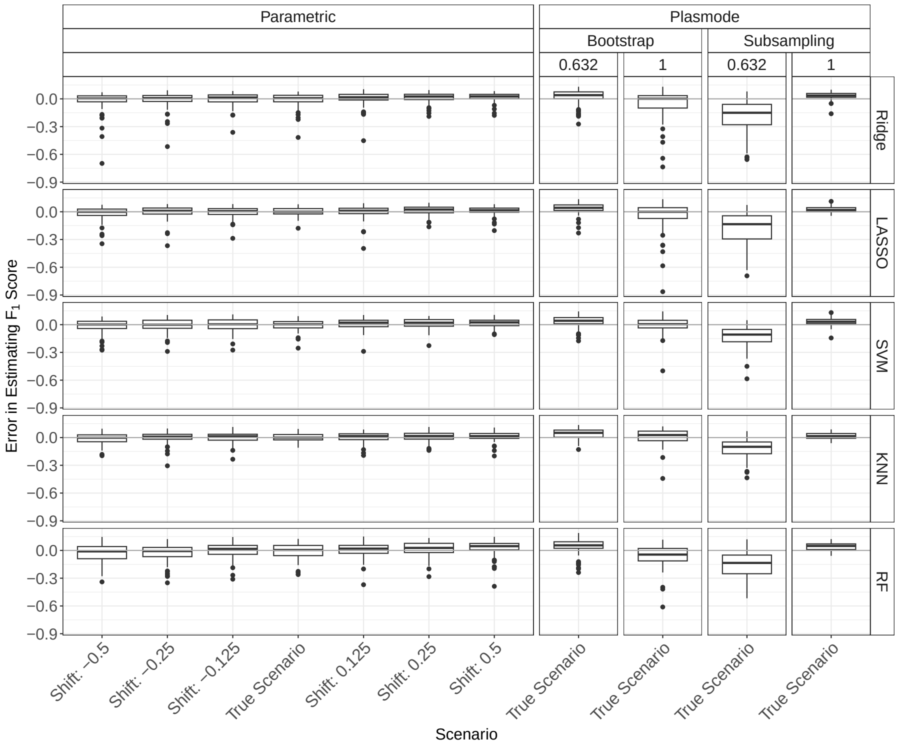

Fig C.10: Errors in the estimation of the F_1-score in 100 iterations of a classification method comparison study per classifier for different simulation approaches with misspecified shift for parametric simulation for $p = 10$.

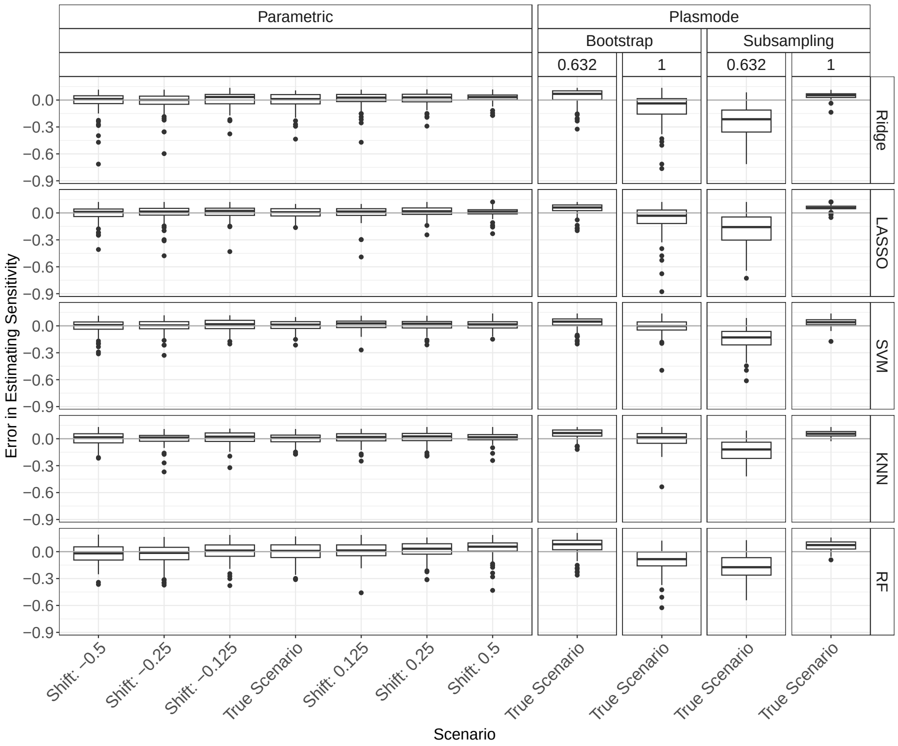

Fig C.11: Errors in the estimation of sensitivity in 100 iterations of a classification method comparison study per classifier for different simulation approaches with misspecified shift for parametric simulation for $p = 10$.

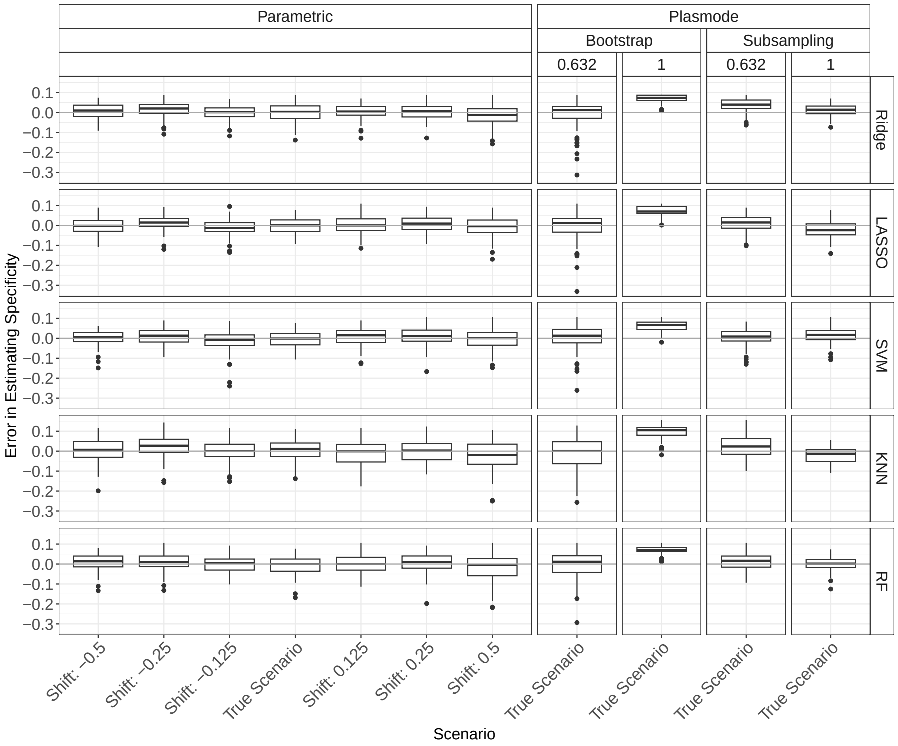

Fig C.12: Errors in the estimation of specificity in 100 iterations of a classification method comparison study per classifier for different simulation approaches with misspecified shift for parametric simulation for $p = 10$.

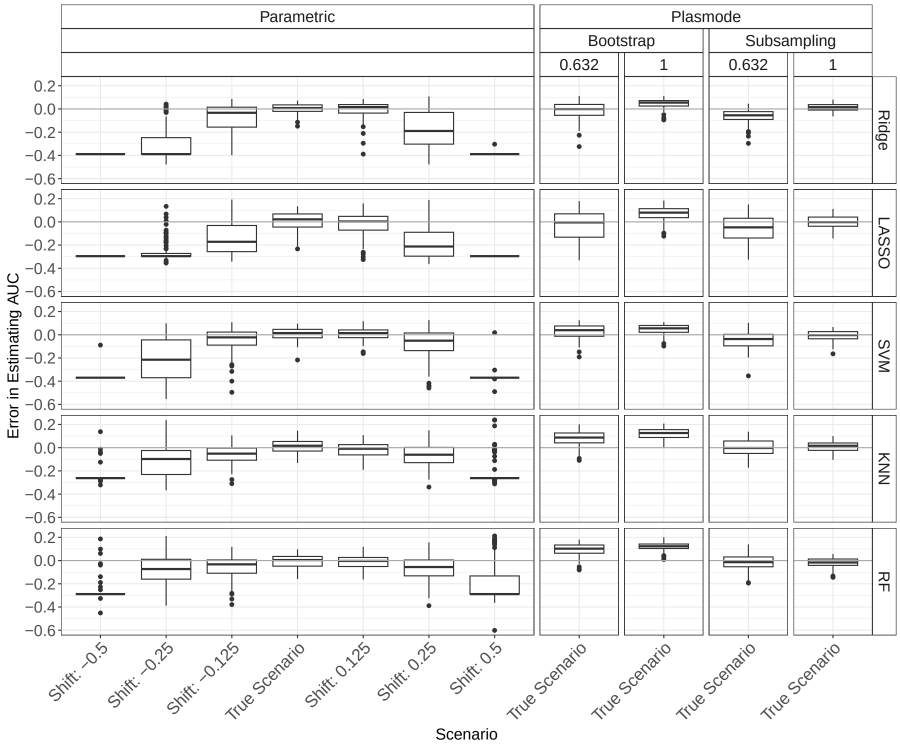

Fig C.13: Errors in the estimation of AUC in 100 iterations of a classification method comparison study per classifier for different simulation approaches with misspecified shift for parametric simulation for $p = 50$.

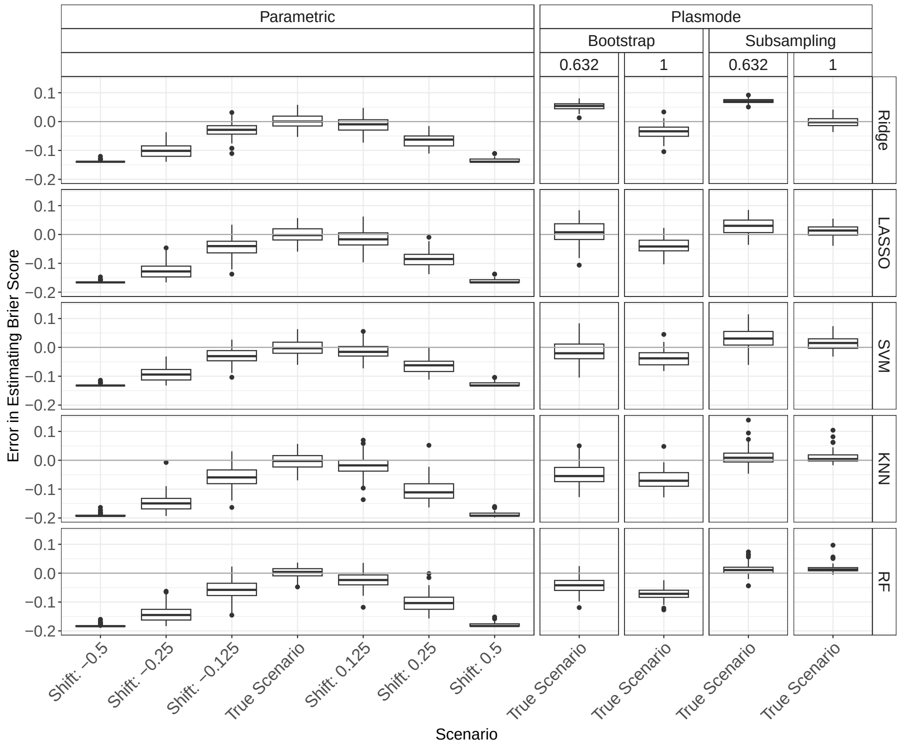

Fig C.14: Errors in the estimation of the Brier score in 100 iterations of a classification method comparison study per classifier for different simulation approaches with misspecified shift for parametric simulation for $p = 50$.

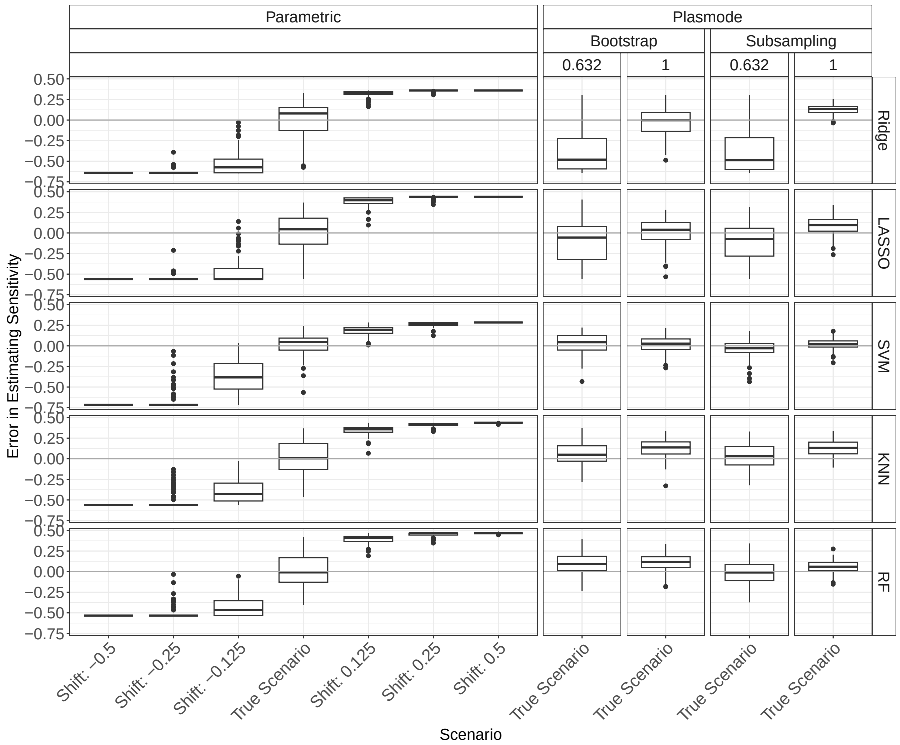

Fig C.15: Errors in the estimation of sensitivity in 100 iterations of a classification method comparison study per classifier for different simulation approaches with misspecified shift for parametric simulation for $p = 50$.

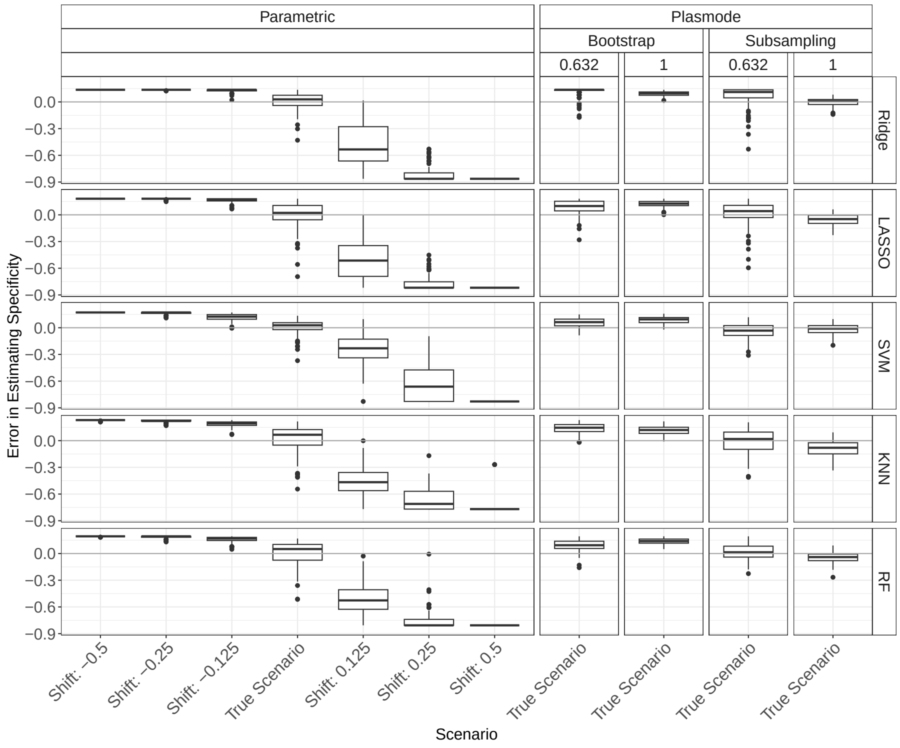

Fig C.16: Errors in the estimation of specificity in 100 iterations of a classification method comparison study per classifier for different simulation approaches with misspecified shift for parametric simulation for $p = 50$.

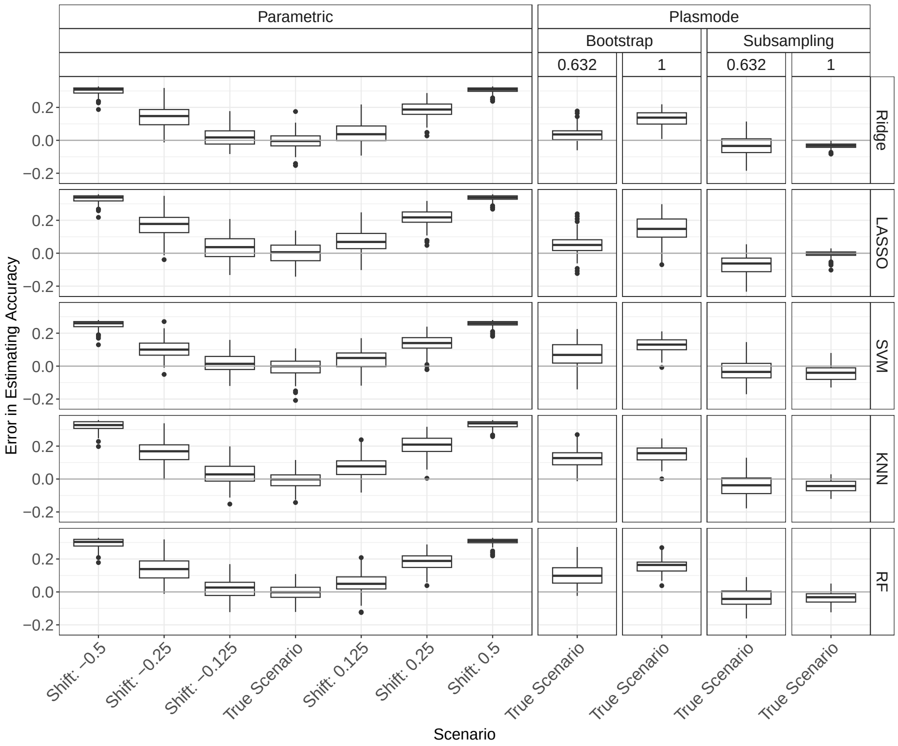

Fig C.17: Errors in the estimation of accuracy in 100 iterations of a classification method comparison study per classifier for different simulation approaches with misspecified shift for parametric simulation for $p = 150$.

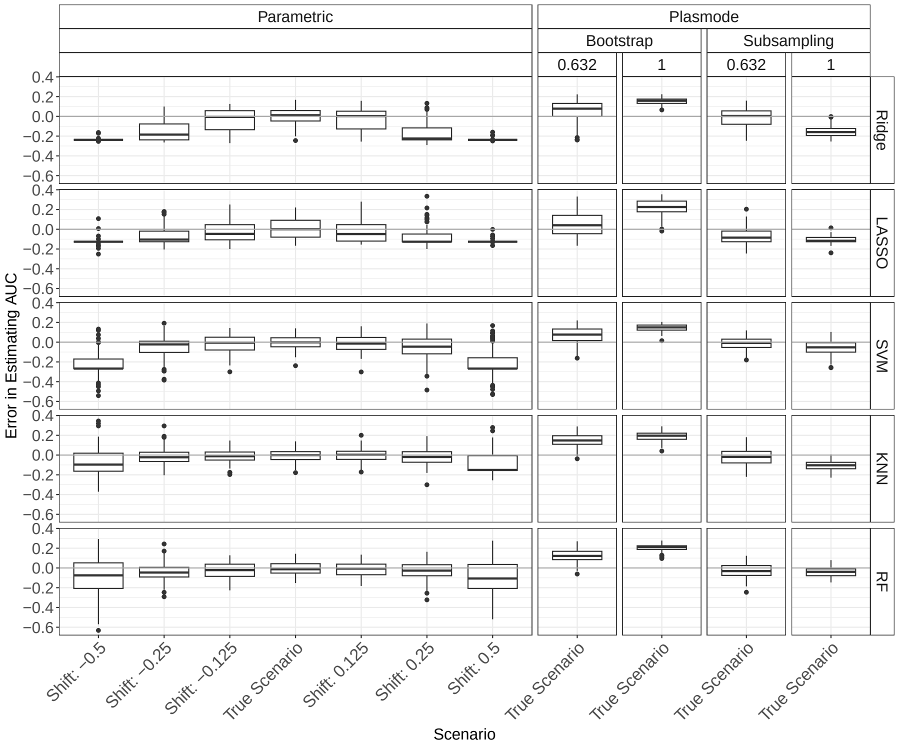

Fig C.18: Errors in the estimation of AUC in 100 iterations of a classification method comparison study per classifier for different simulation approaches with misspecified shift for parametric simulation for $p = 150$.

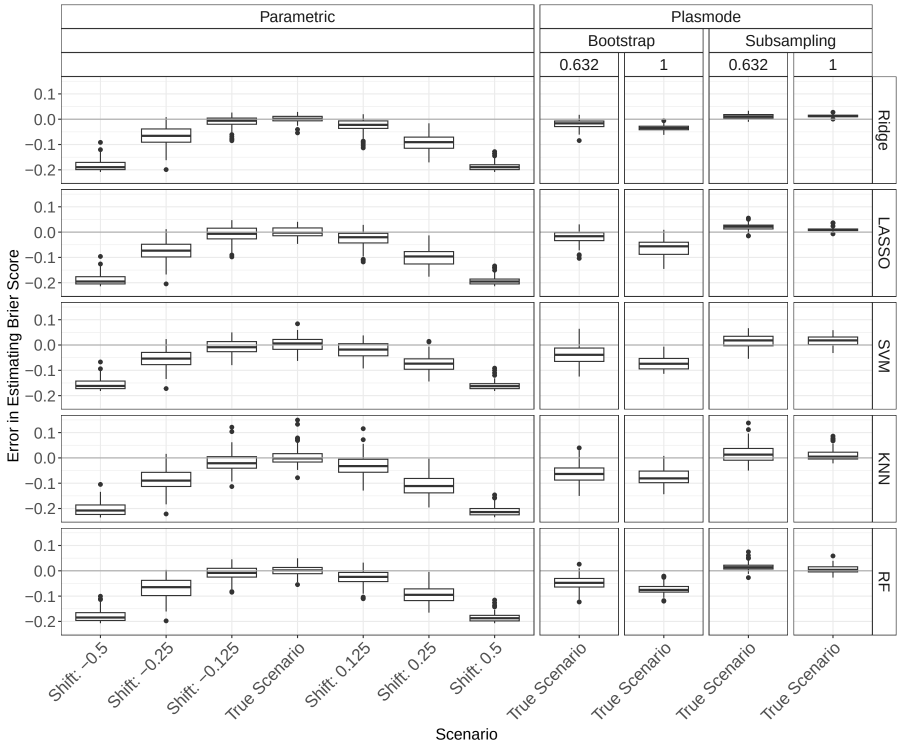

Fig C.19: Errors in the estimation of the Brier score in 100 iterations of a classification method comparison study per classifier for different simulation approaches with misspecified shift for parametric simulation for $p = 150$.

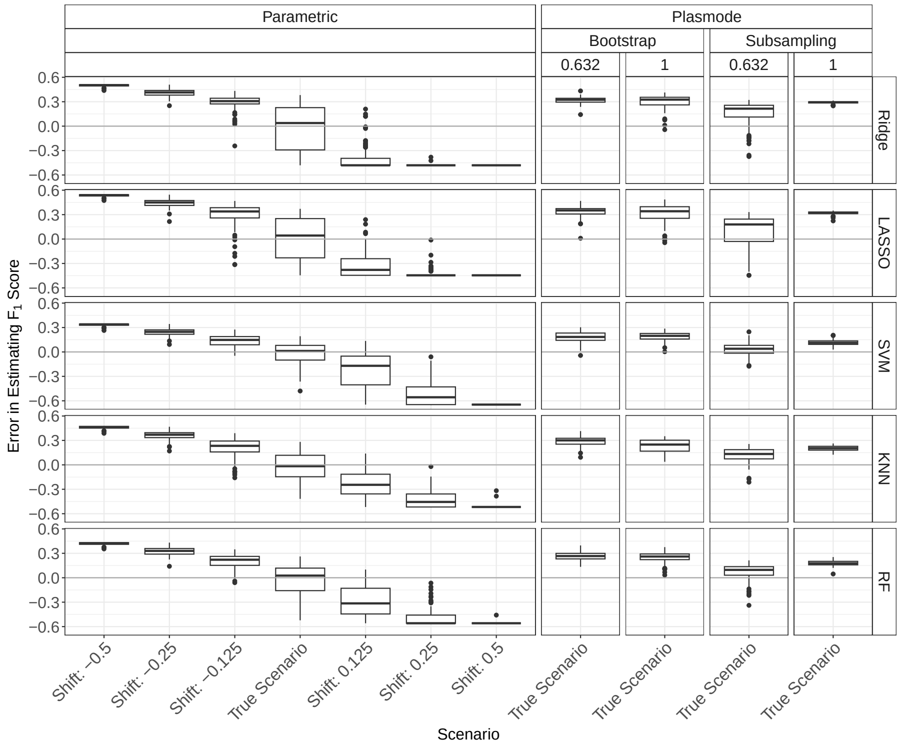

Fig C.20: Errors in the estimation of the F_1-score in 100 iterations of a classification method comparison study per classifier for different simulation approaches with misspecified shift for parametric simulation for $p = 150$.

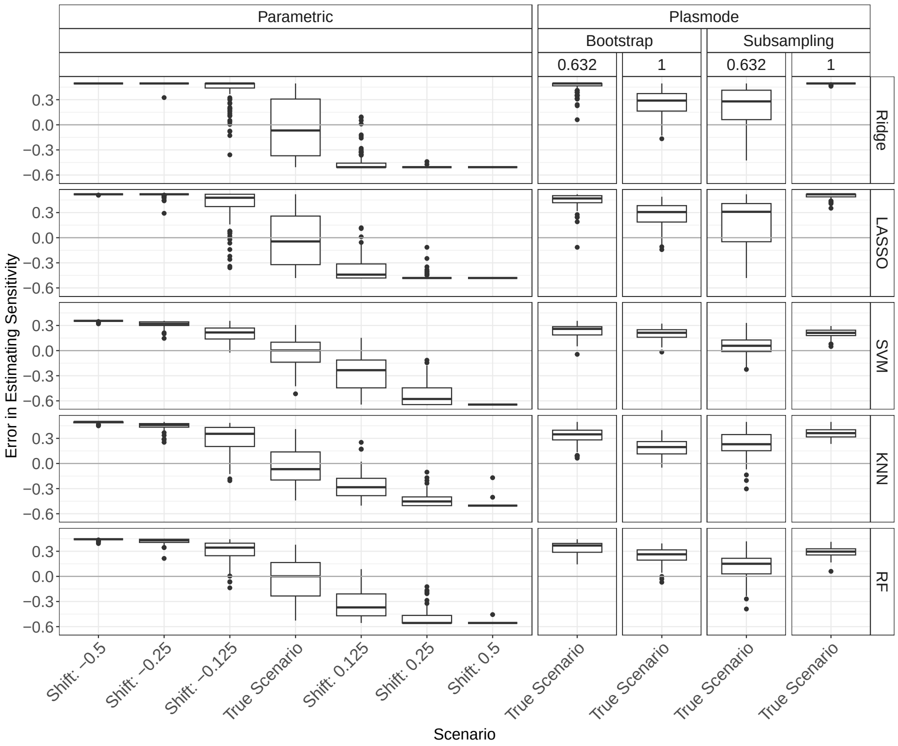

Fig C.21: Errors in the estimation of sensitivity in 100 iterations of a classification method comparison study per classifier for different simulation approaches with misspecified shift for parametric simulation for $p = 150$.

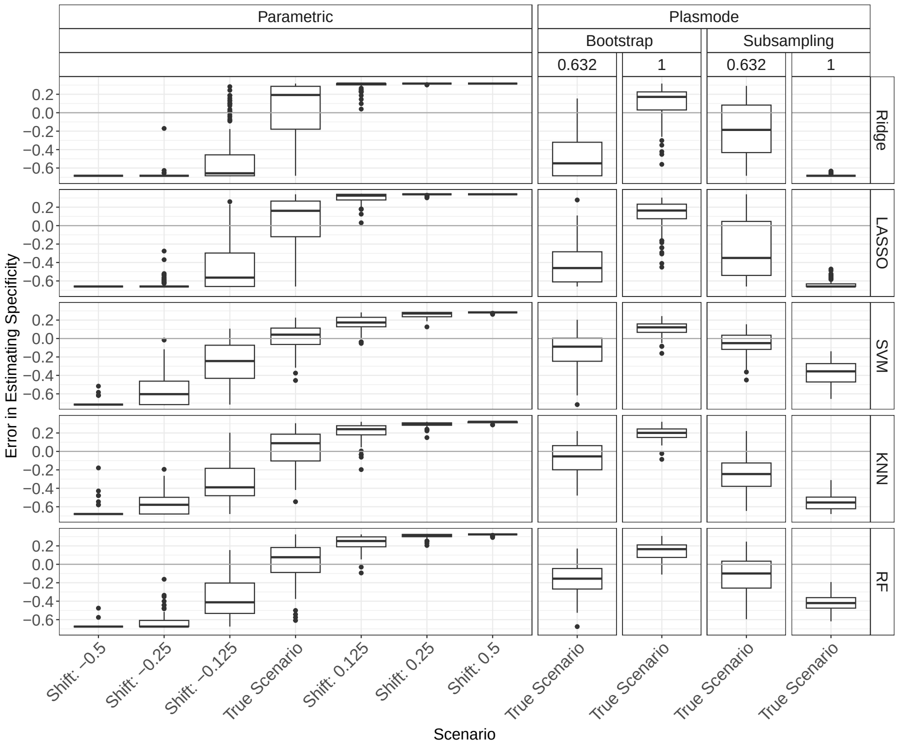

Fig C.22: Errors in the estimation of specificity in 100 iterations of a classification method comparison study per classifier for different simulation approaches with misspecified shift for parametric simulation for $p = 150$.

D Scale

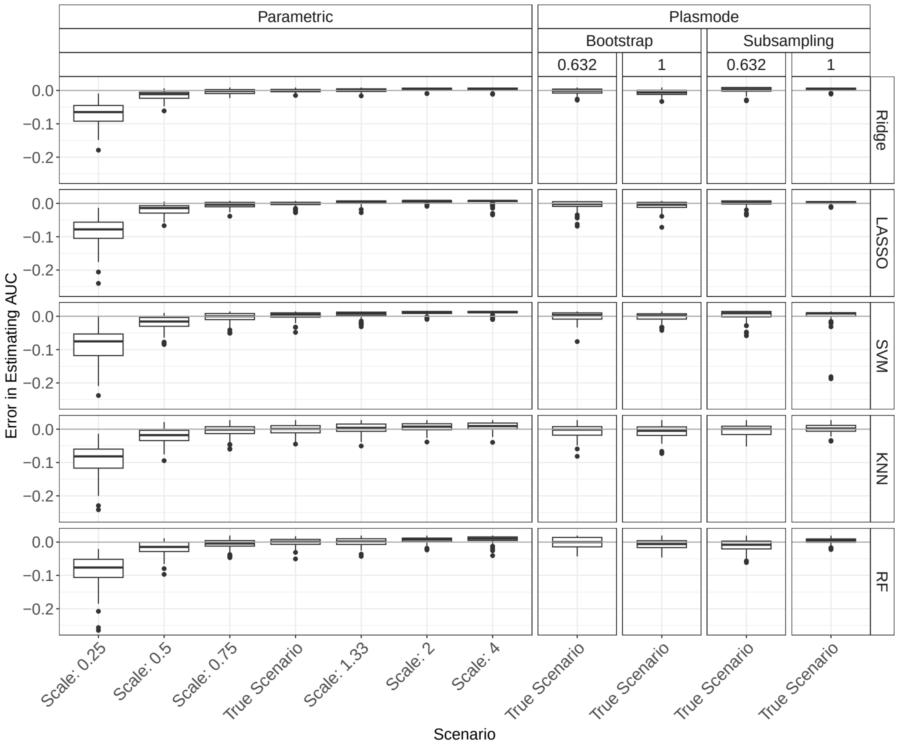

Fig D.1: Errors in the estimation of AUC in 100 iterations of a classification method comparison study per classifier for different simulation approaches with misspecified scale for parametric simulation for $p = 2$.

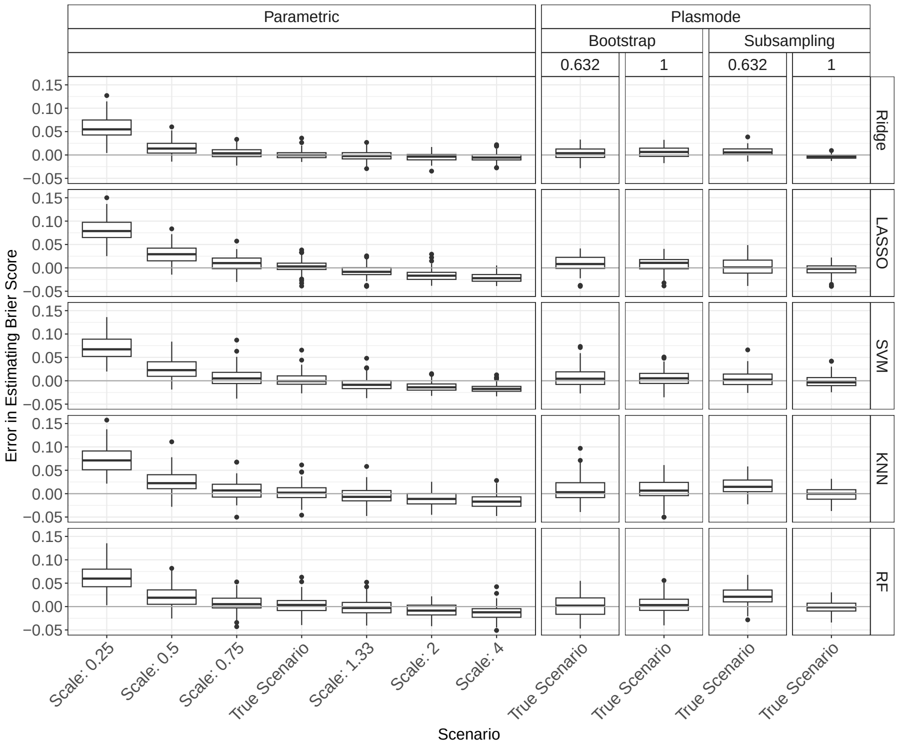

Fig D.2: Errors in the estimation of the Brier score in 100 iterations of a classification method comparison study per classifier for different simulation approaches with misspecified scale for parametric simulation for $p = 2$.

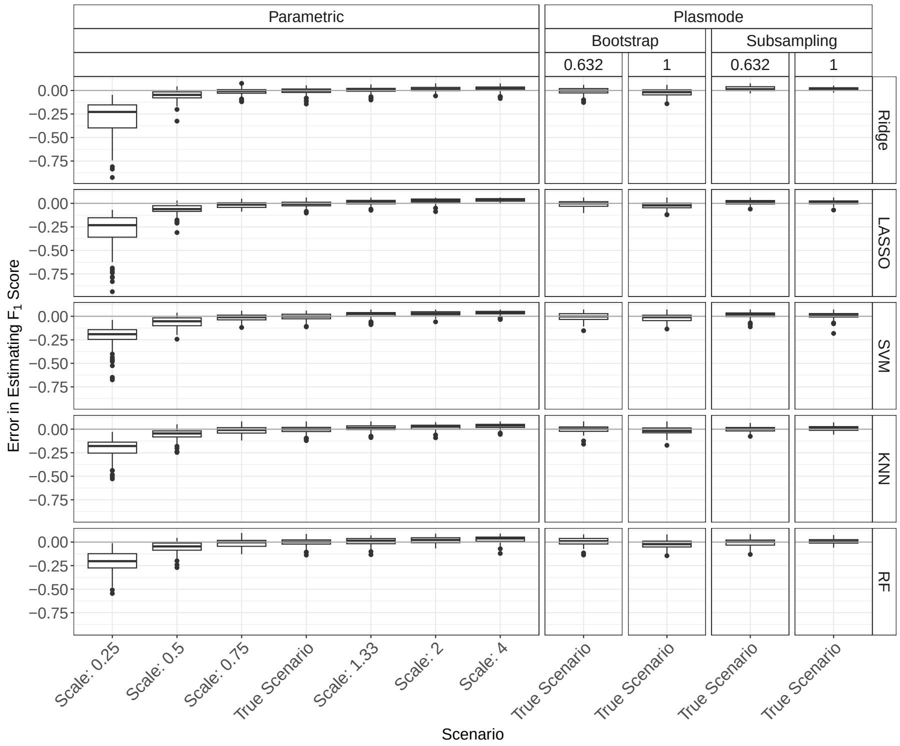

Fig D.3: Errors in the estimation of the F1 score in 100 iterations of a classification method comparison study per classifier for different simulation approaches with misspecified scale for parametric simulation for $p = 2$.

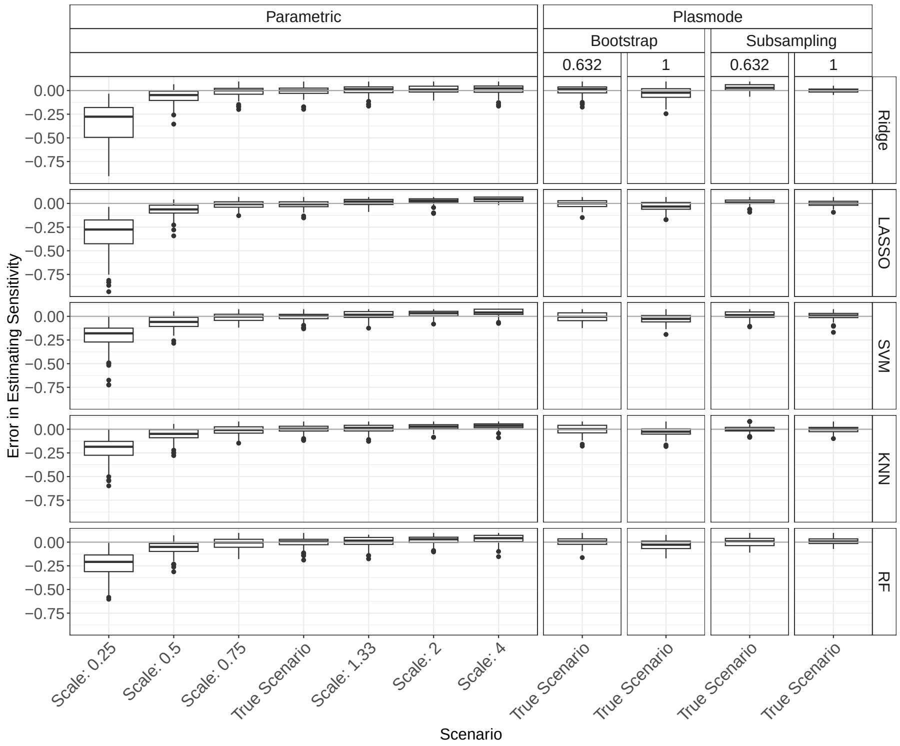

Fig D.4: Errors in the estimation of sensitivity in 100 iterations of a classification method comparison study per classifier for different simulation approaches with misspecified scale for parametric simulation for $p = 2$.

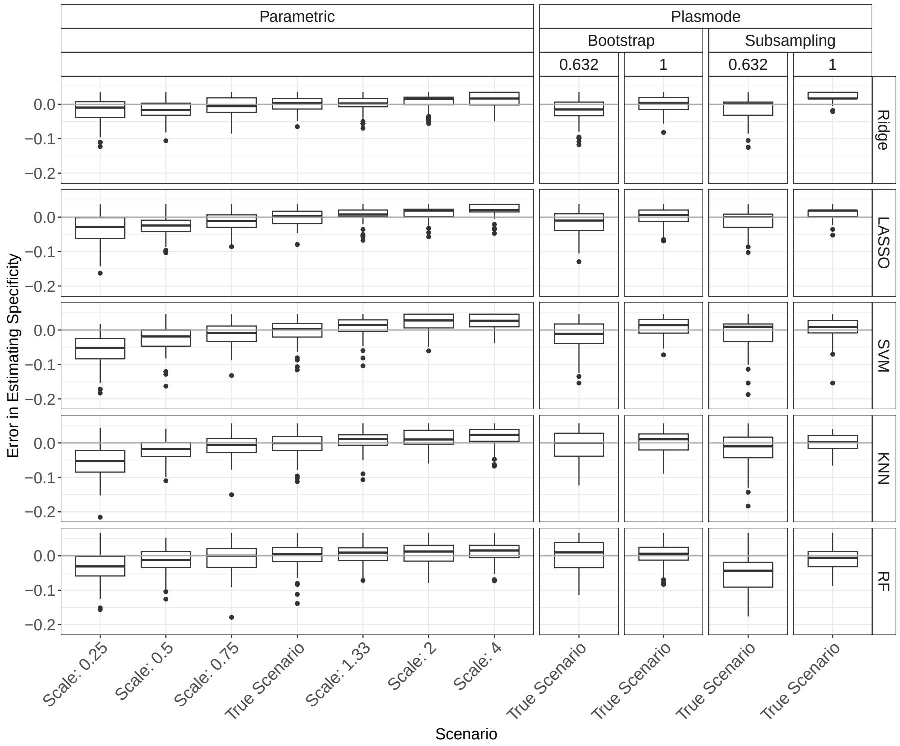

Fig D.5: Errors in the estimation of specificity in 100 iterations of a classification method comparison study per classifier for different simulation approaches with misspecified scale for parametric simulation for $p = 2$.

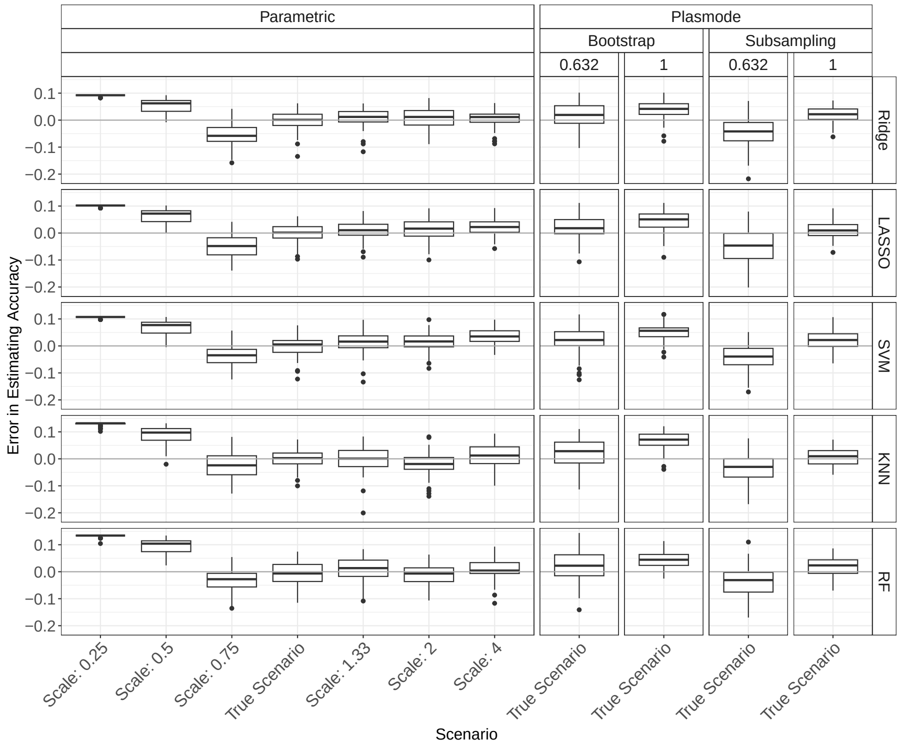

Fig D.6: Errors in the estimation of accuracy in 100 iterations of a classification method comparison study per classifier for different simulation approaches with misspecified scale for parametric simulation for $p = 10$.

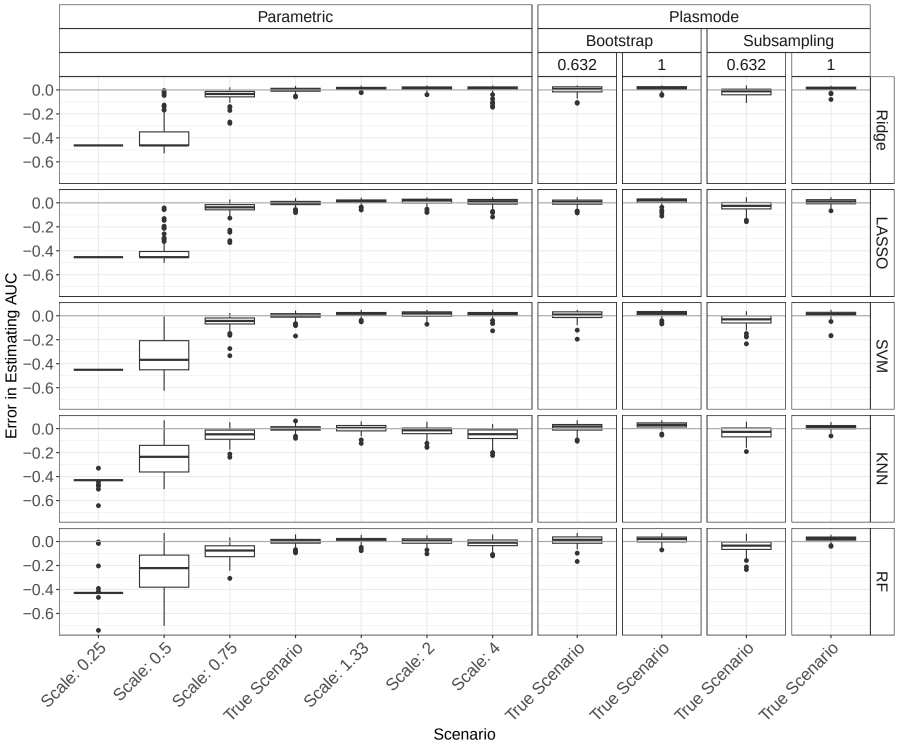

Fig D.7: Errors in the estimation of AUC in 100 iterations of a classification method comparison study per classifier for different simulation approaches with misspecified scale for parametric simulation for $p = 10$.

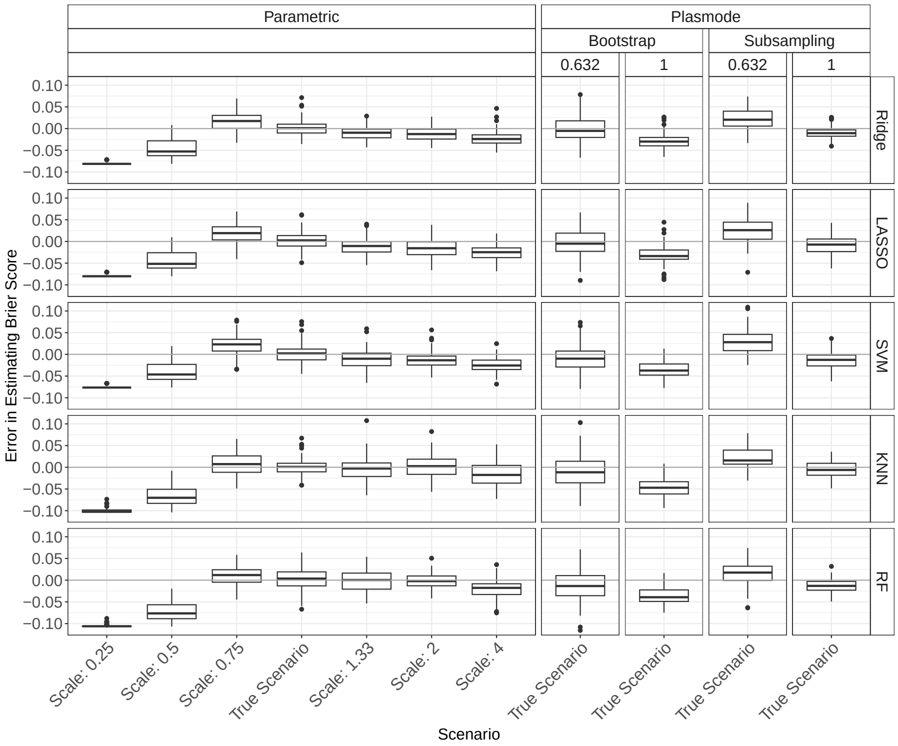

Fig D.8: Errors in the estimation of the Brier score in 100 iterations of a classification method comparison study per classifier for different simulation approaches with misspecified scale for parametric simulation for $p = 10$.

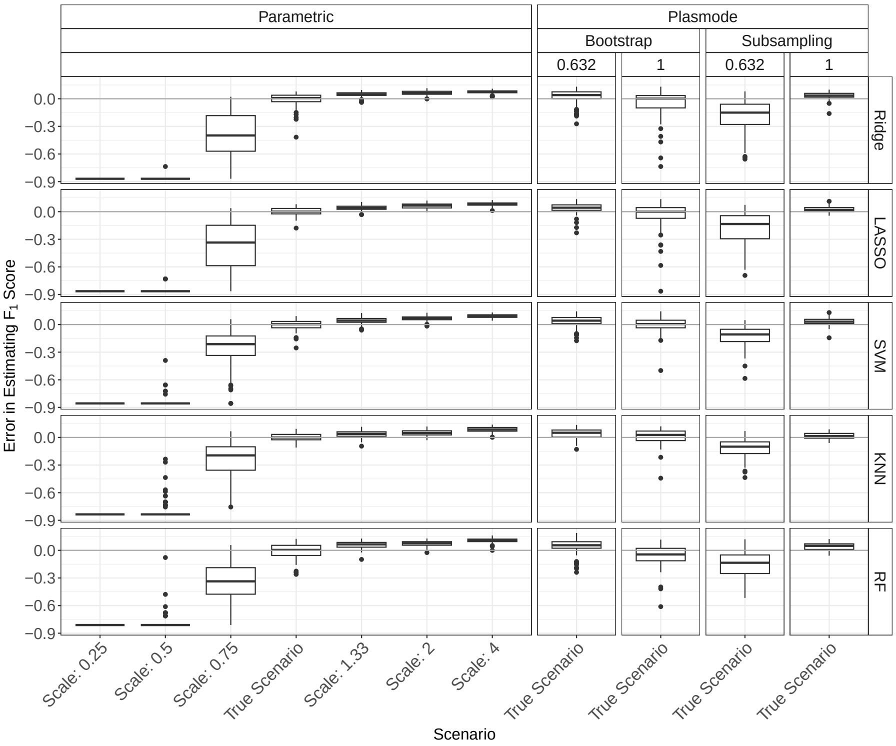

Fig D.9: Errors in the estimation of the F_1-score in 100 iterations of a classification method comparison study per classifier for different simulation approaches with misspecified scale for parametric simulation for $p = 10$.

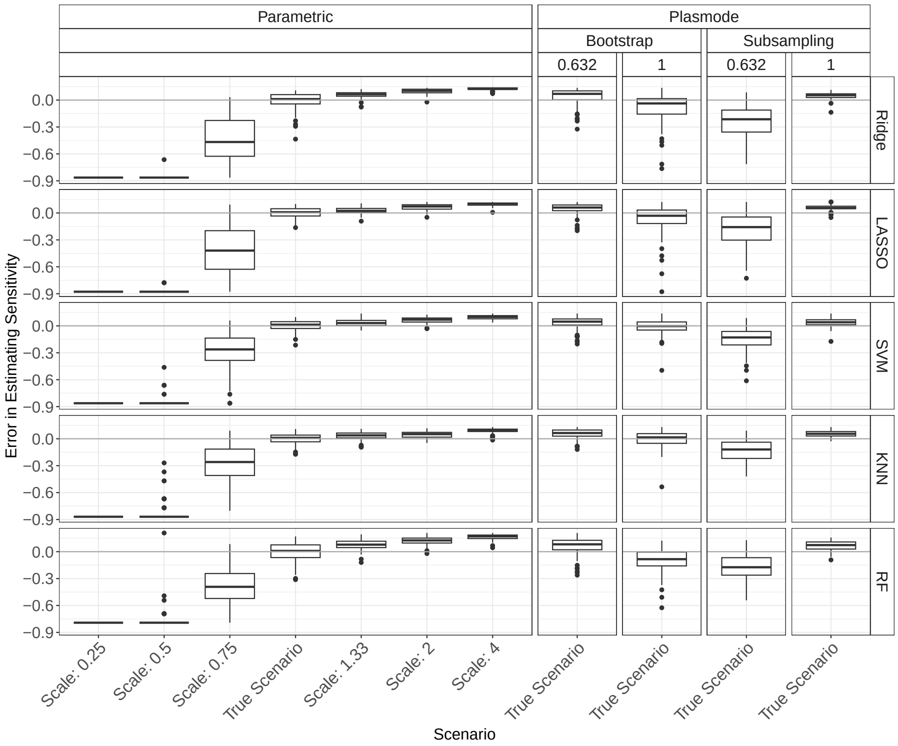

Fig D.10: Errors in the estimation of sensitivity in 100 iterations of a classification method comparison study per classifier for different simulation approaches with misspecified scale for parametric simulation for $p = 10$.

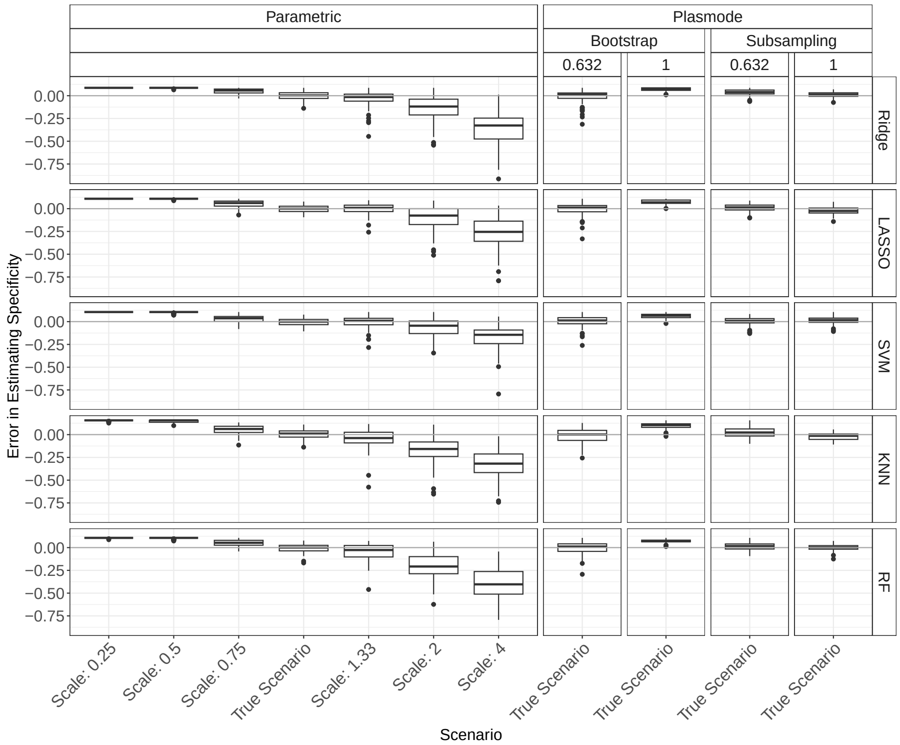

Fig D.11: Errors in the estimation of specificity in 100 iterations of a classification method comparison study per classifier for different simulation approaches with misspecified scale for parametric simulation for $p = 10$.

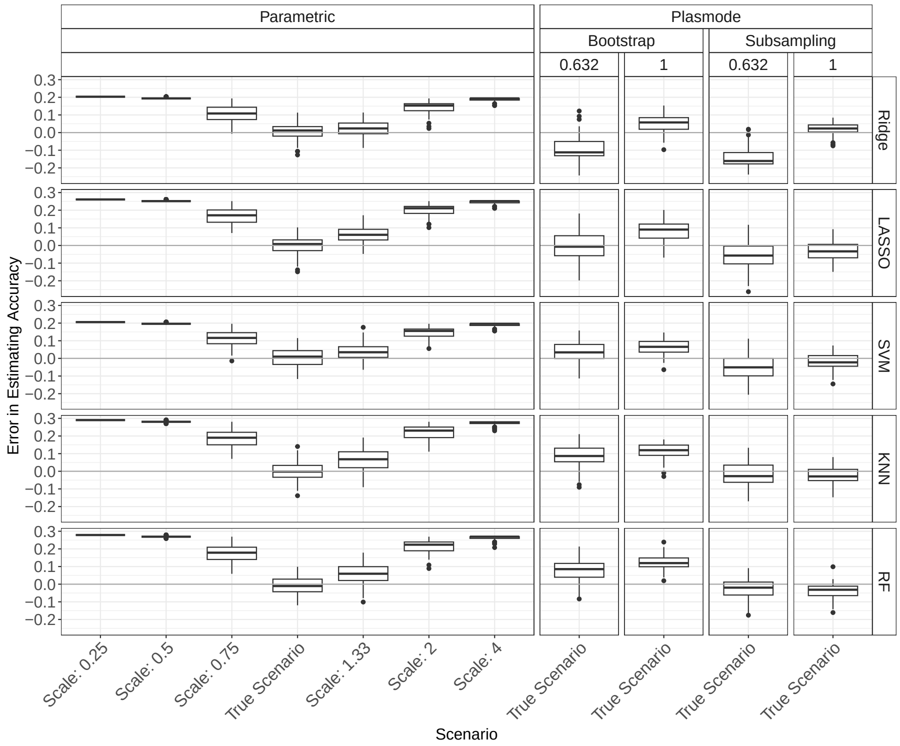

Fig D.12: Errors in the estimation of accuracy in 100 iterations of a classification method comparison study per classifier for different simulation approaches with misspecified scale for parametric simulation for $p = 50$.

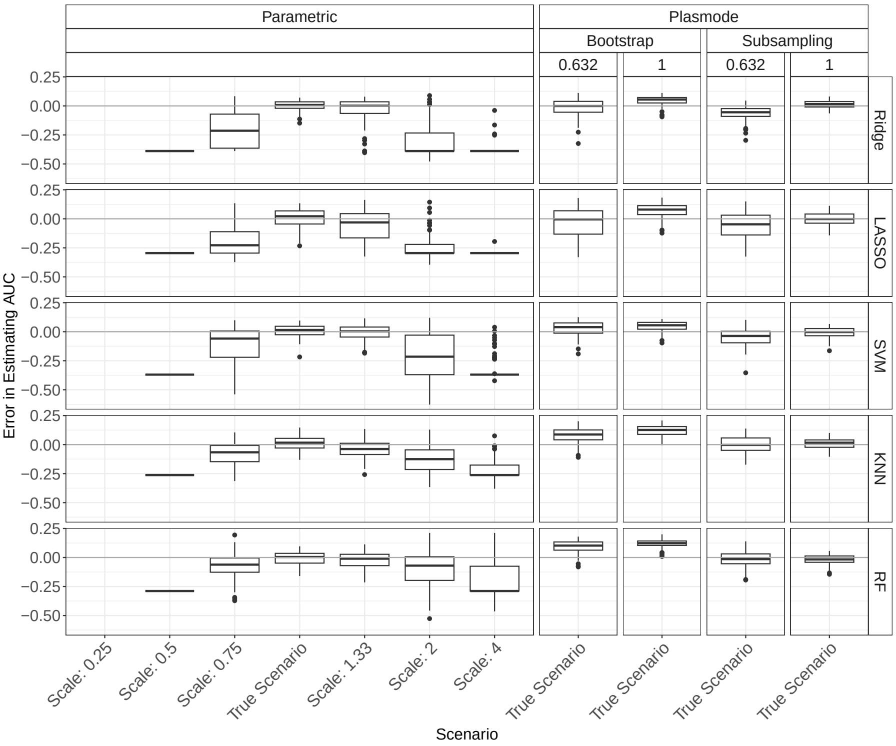

Fig D.13: Errors in the estimation of AUC in 100 iterations of a classification method comparison study per classifier for different simulation approaches with misspecified scale for parametric simulation for $p = 50$.

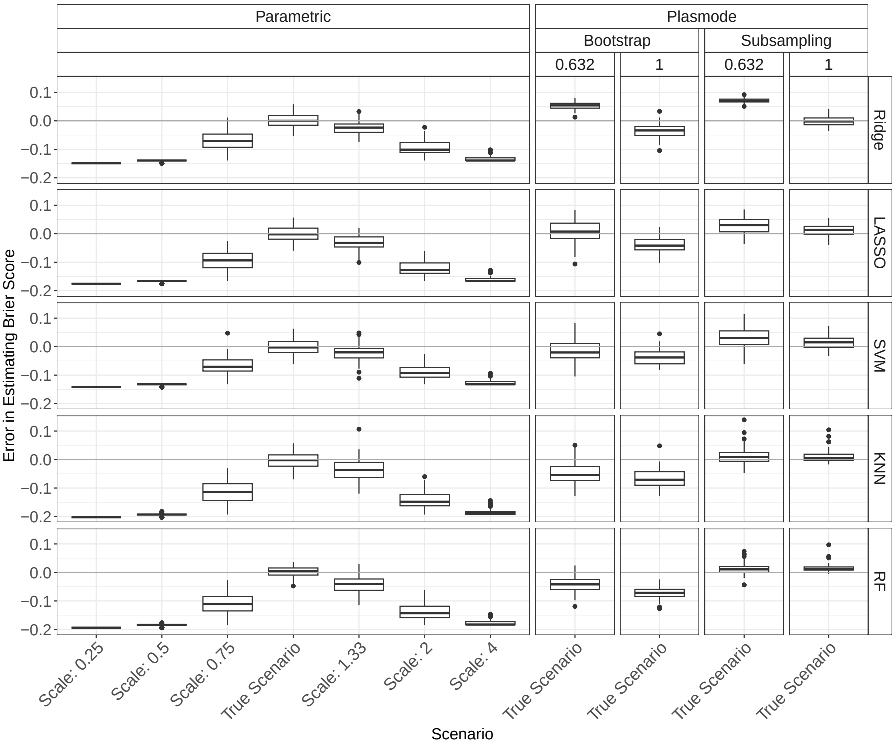

Fig D.14: Errors in the estimation of the Brier score in 100 iterations of a classification method comparison study per classifier for different simulation approaches with misspecified scale for parametric simulation for $p = 50$.

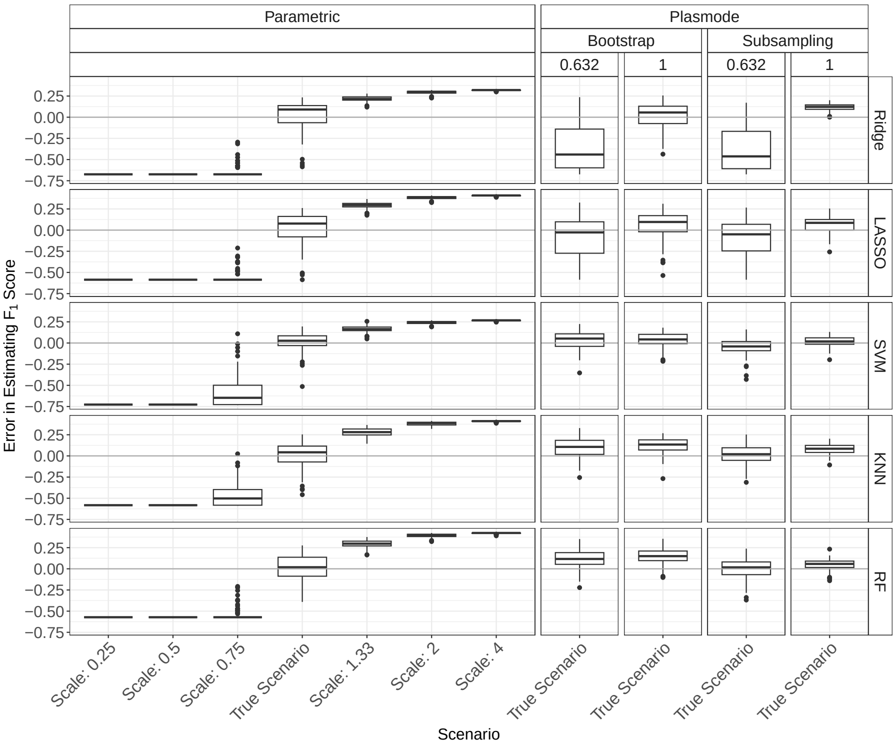

Fig D.15: Errors in the estimation of the F_1-score in 100 iterations of a classification method comparison study per classifier for different simulation approaches with misspecified scale for parametric simulation for $p = 50$.

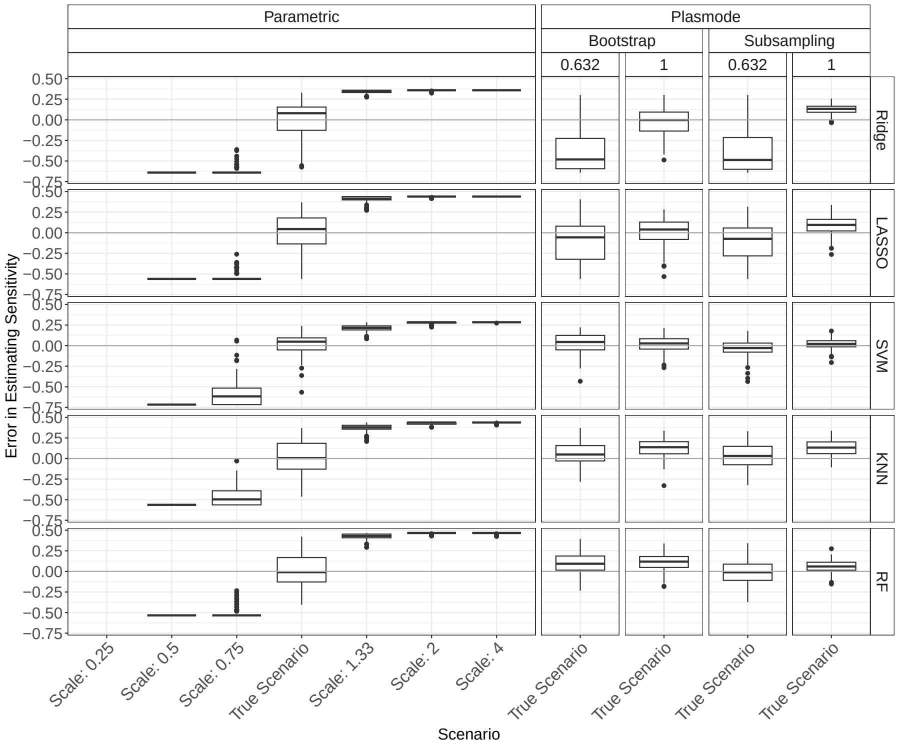

Fig D.16: Errors in the estimation of sensitivity in 100 iterations of a classification method comparison study per classifier for different simulation approaches with misspecified scale for parametric simulation for $p = 50$.

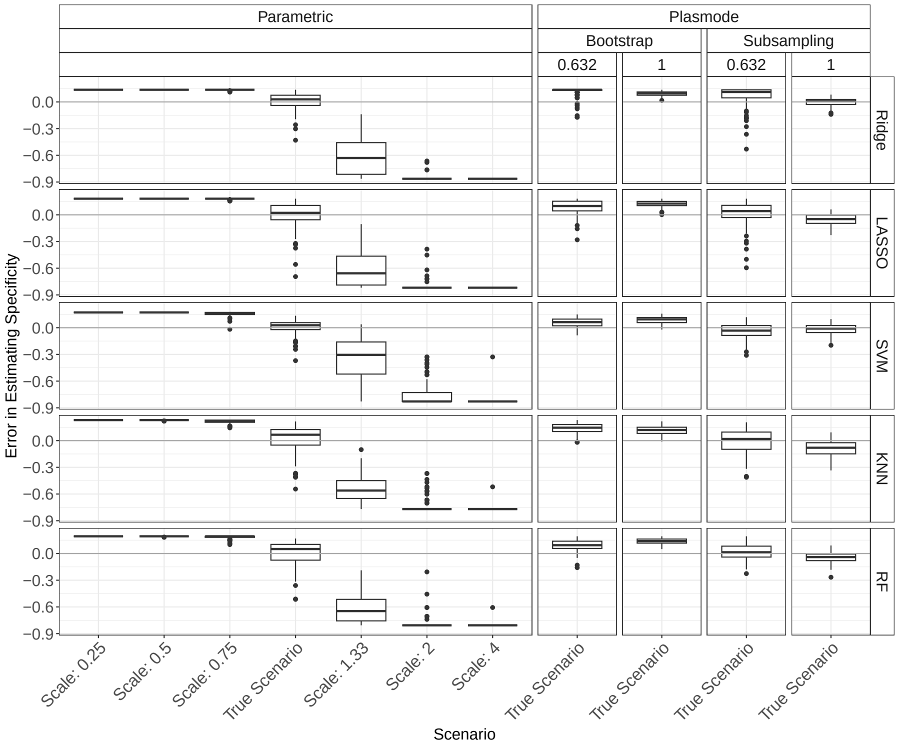

Fig D.17: Errors in the estimation of specificity in 100 iterations of a classification method comparison study per classifier for different simulation approaches with misspecified scale for parametric simulation for $p = 50$.

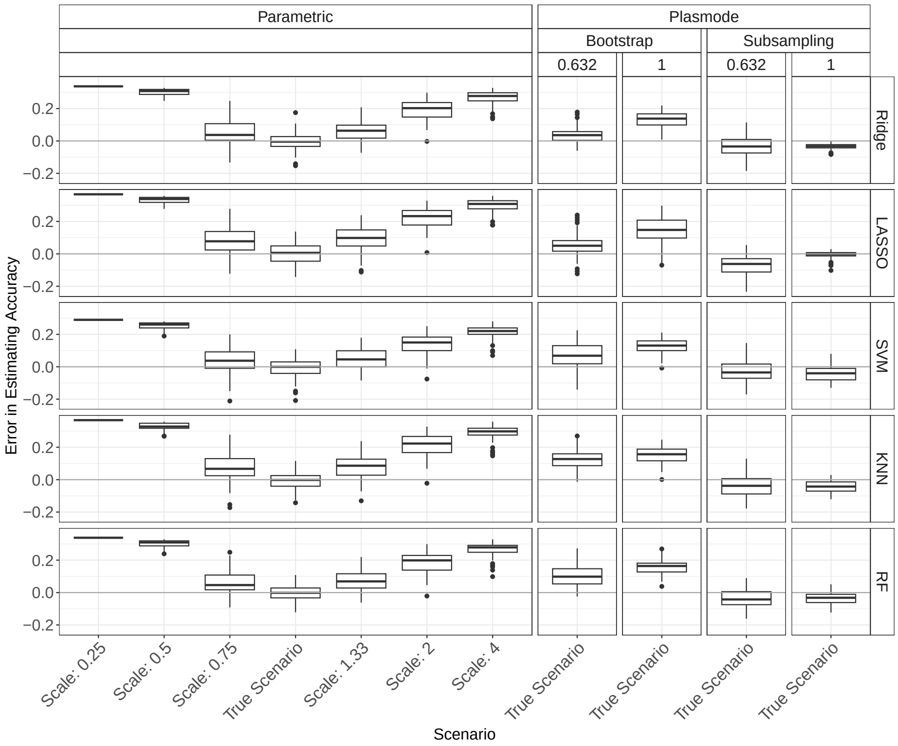

Fig D.18: Errors in the estimation of accuracy in 100 iterations of a classification method comparison study per classifier for different simulation approaches with misspecified scale for parametric simulation for $p = 150$.

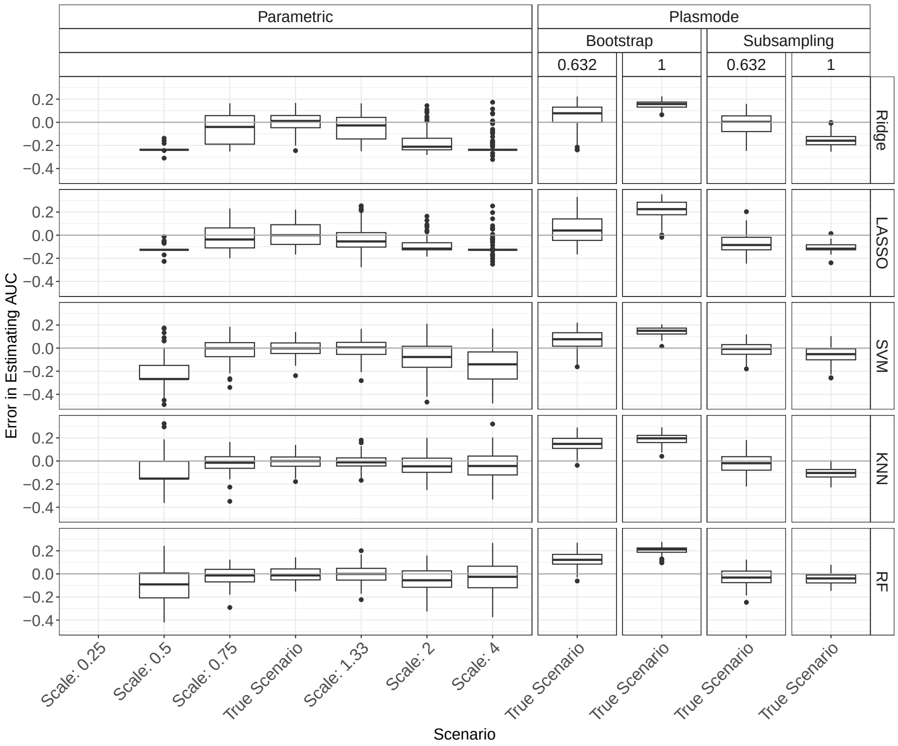

Fig D.19: Errors in the estimation of AUC in 100 iterations of a classification method comparison study per classifier for different simulation approaches with misspecified scale for parametric simulation for $p = 150$.

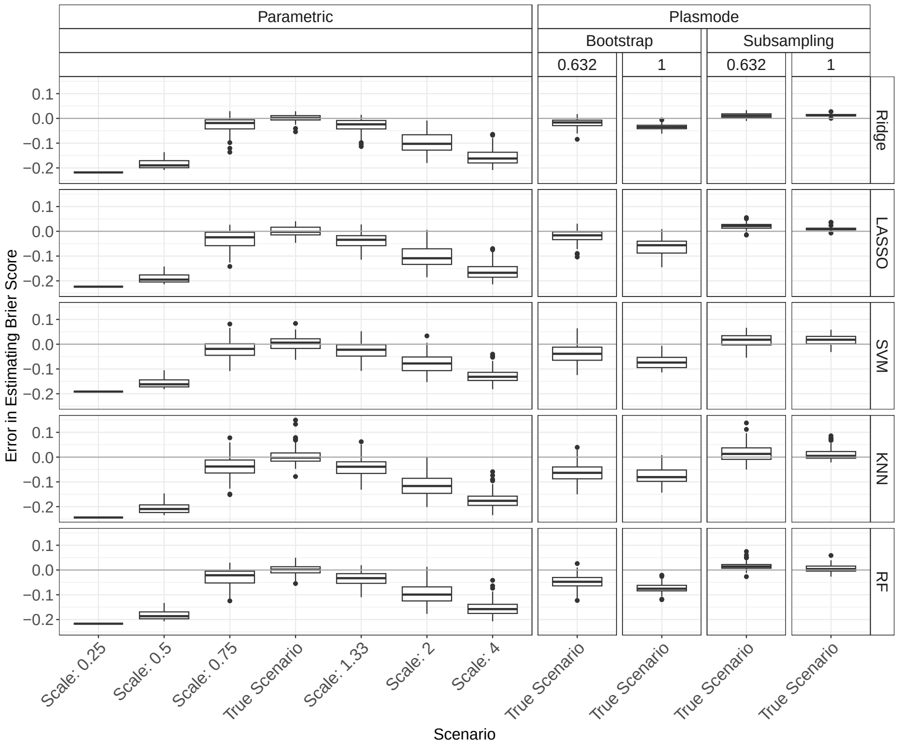

Fig D.20: Errors in the estimation of the Brier score in 100 iterations of a classification method comparison study per classifier for different simulation approaches with misspecified scale for parametric simulation for $p = 150$.

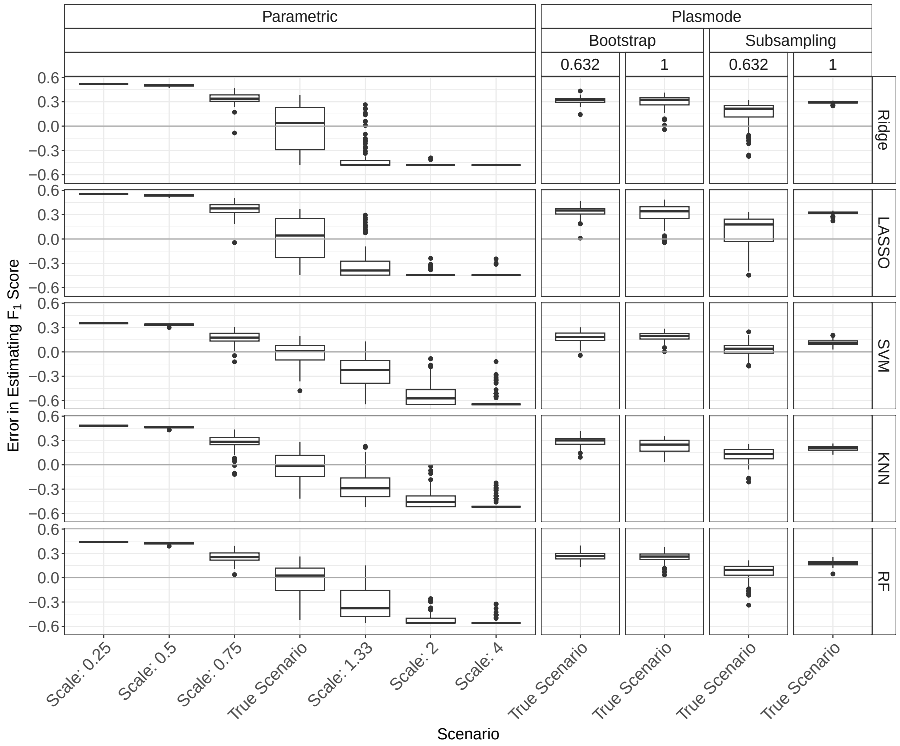

Fig D.21: Errors in the estimation of the F_1-score in 100 iterations of a classification method comparison study per classifier for different simulation approaches with misspecified scale for parametric simulation for $p = 150$.

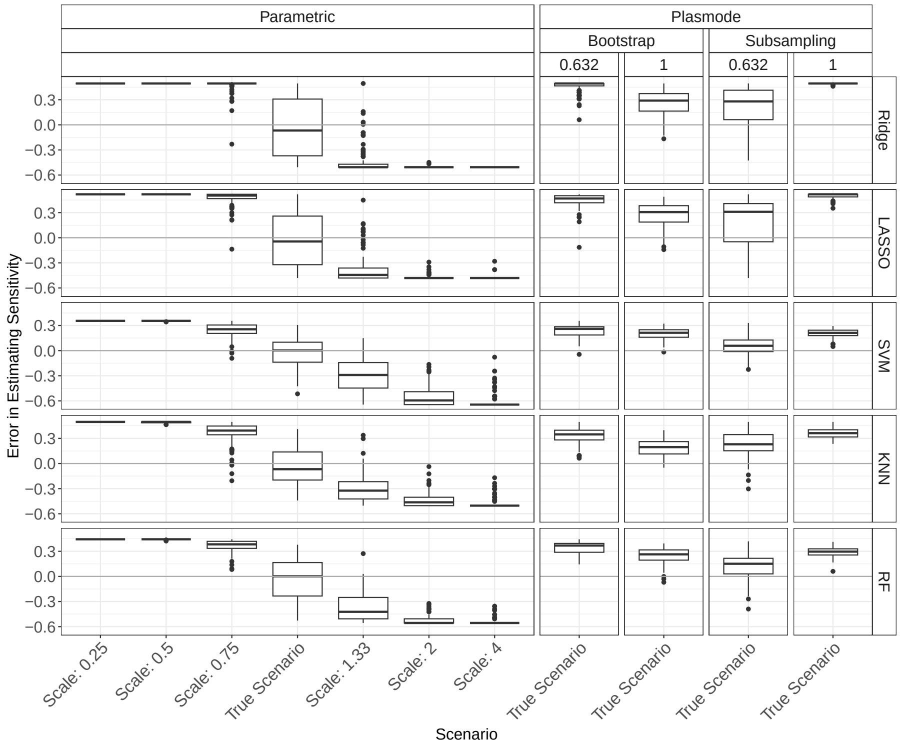

Fig D.22: Errors in the estimation of sensitivity in 100 iterations of a classification method comparison study per classifier for different simulation approaches with misspecified scale for parametric simulation for $p = 150$.

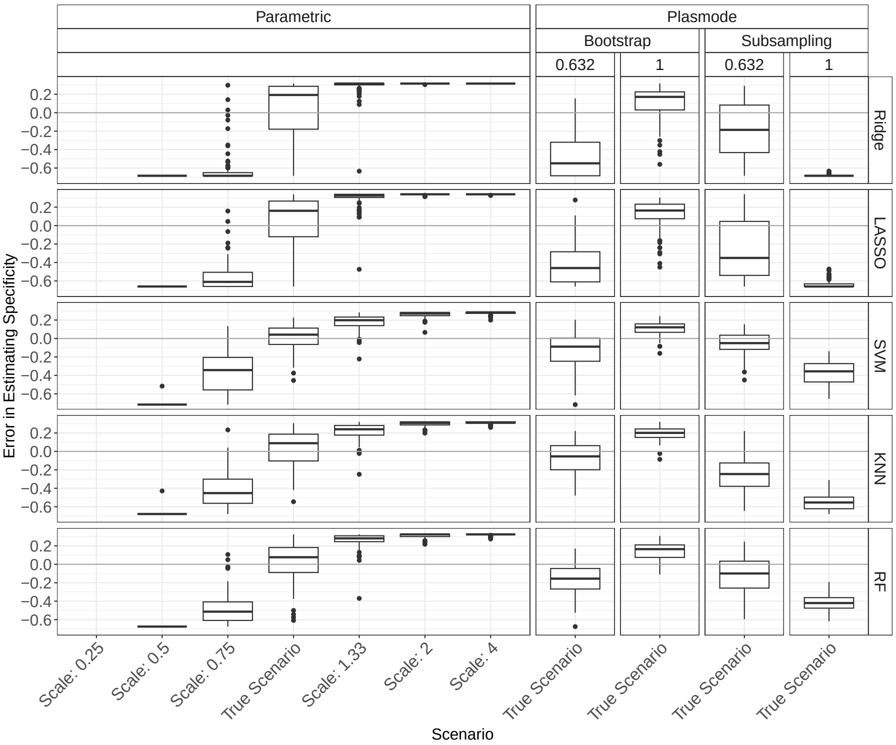

Fig D.23: Errors in the estimation of specificity in 100 iterations of a classification method comparison study per classifier for different simulation approaches with misspecified scale for parametric simulation for $p = 150$.

E Correlation

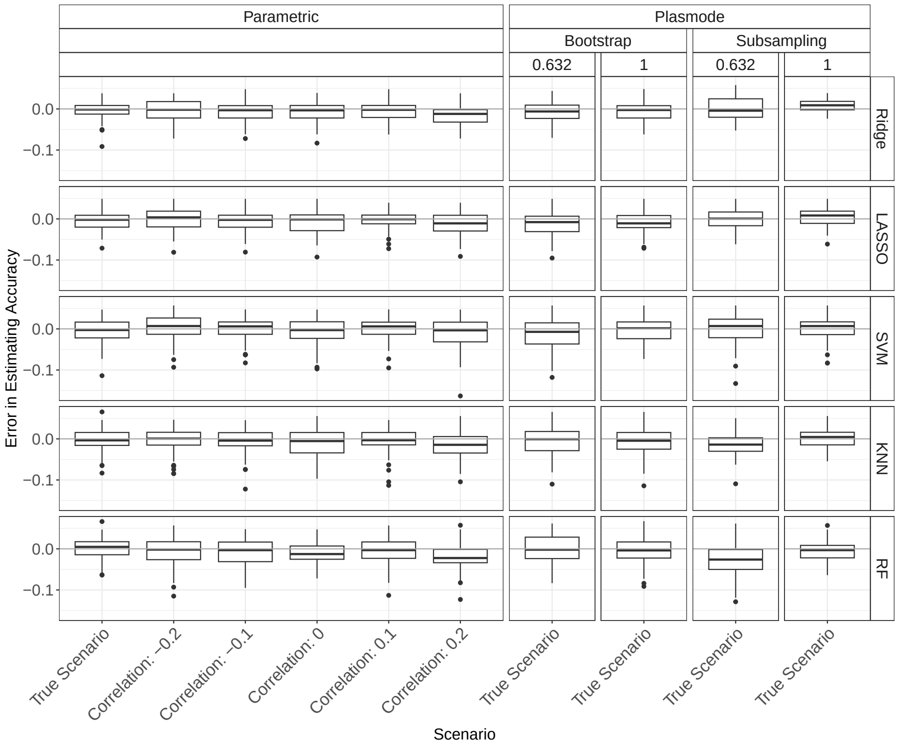

Fig E.1: Errors in the estimation of accuracy in 100 iterations of a classification method comparison study per classifier for different simulation approaches with misspecified correlation for parametric simulation for $p = 2$.

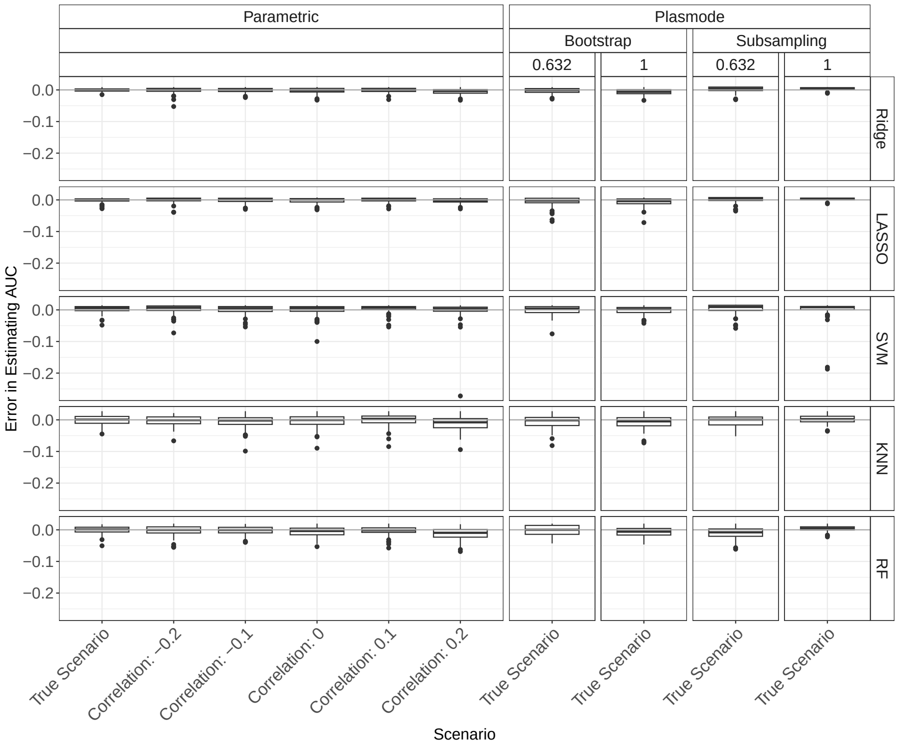

Fig E.2: Errors in the estimation of AUC in 100 iterations of a classification method comparison study per classifier for different simulation approaches with misspecified correlation for parametric simulation for $p = 2$.

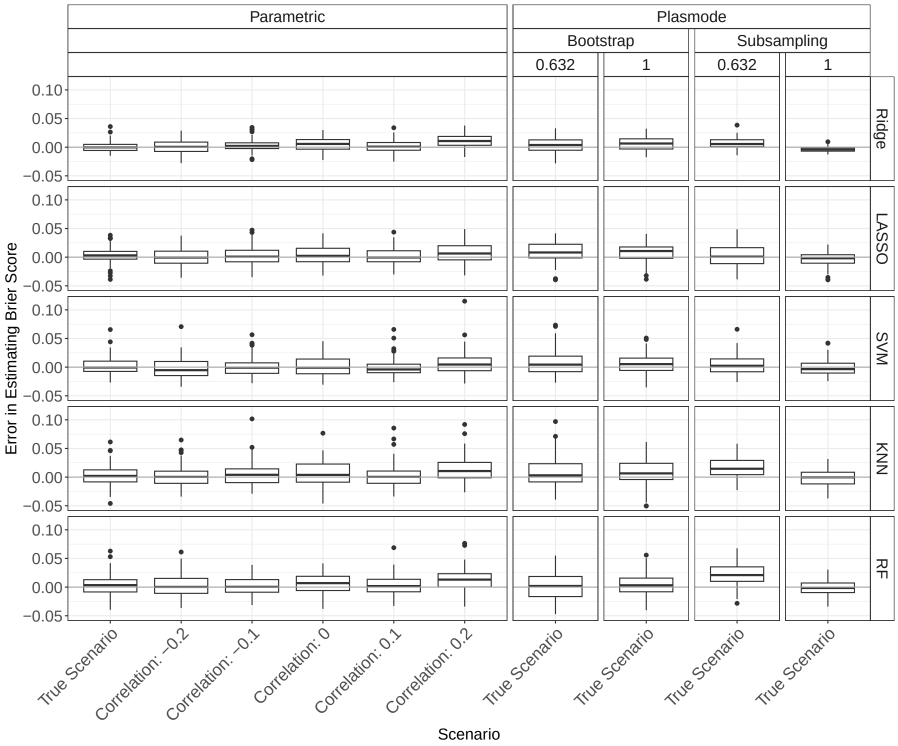

Fig E.3: Errors in the estimation of the Brier score in 100 iterations of a classification method comparison study per classifier for different simulation approaches with misspecified correlation for parametric simulation for $p = 2$.

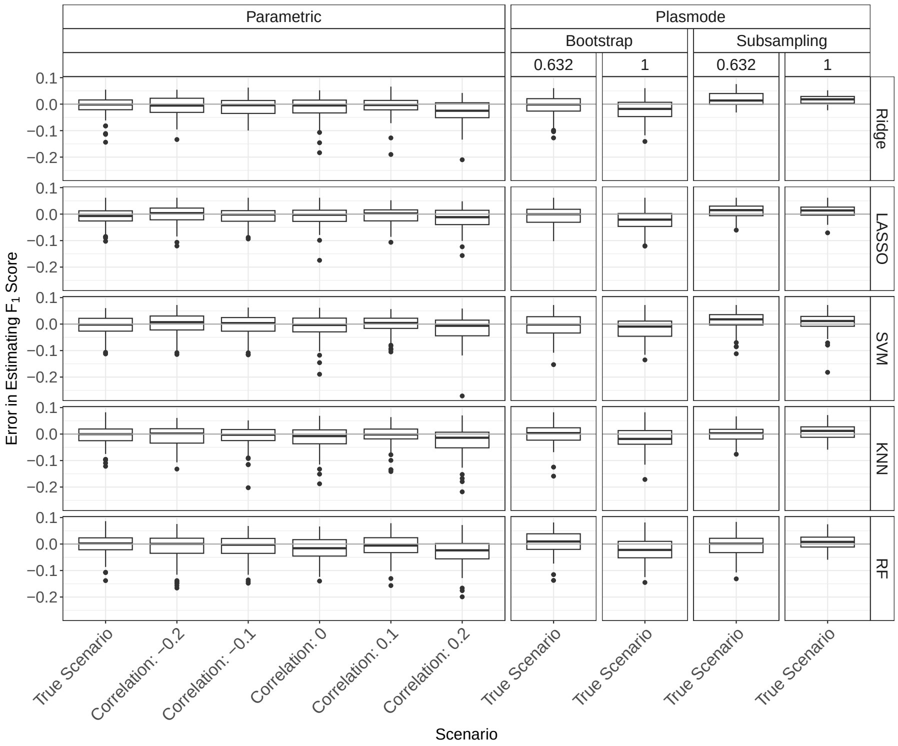

Fig E.4: Errors in the estimation of the F1 score in 100 iterations of a classification method comparison study per classifier for different simulation approaches with misspecified correlation for parametric simulation for $p = 2$.

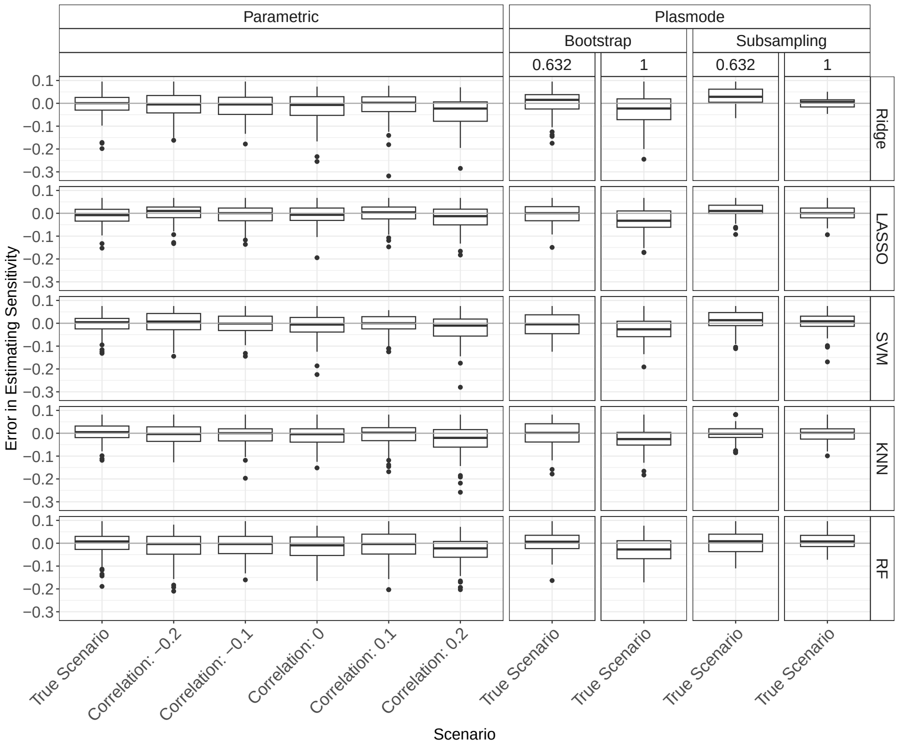

Fig E.5: Errors in the estimation of sensitivity in 100 iterations of a classification method comparison study per classifier for different simulation approaches with misspecified correlation for parametric simulation for $p = 2$.

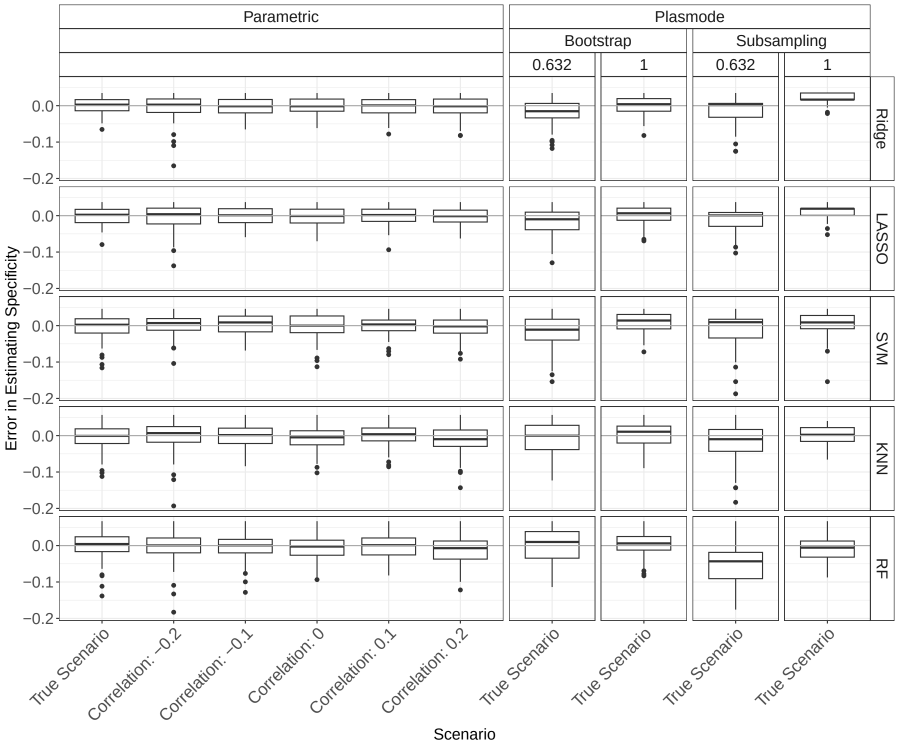

Fig E.6: Errors in the estimation of specificity in 100 iterations of a classification method comparison study per classifier for different simulation approaches with misspecified correlation for parametric simulation for $p = 2$.

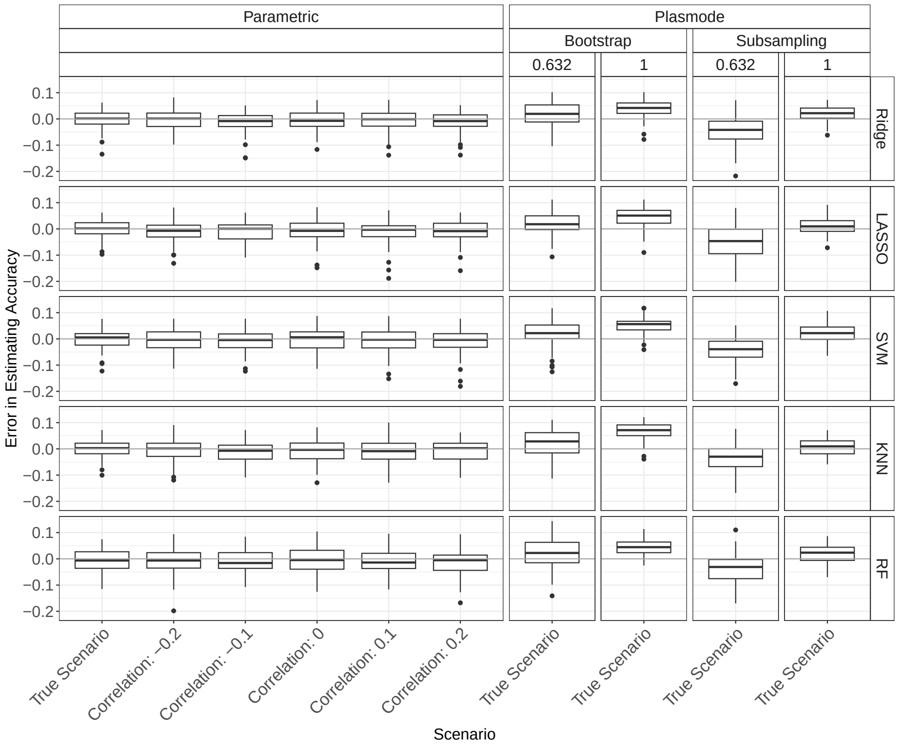

Fig E.7: Errors in the estimation of accuracy in 100 iterations of a classification method comparison study per classifier for different simulation approaches with misspecified correlation for parametric simulation for $p = 10$.

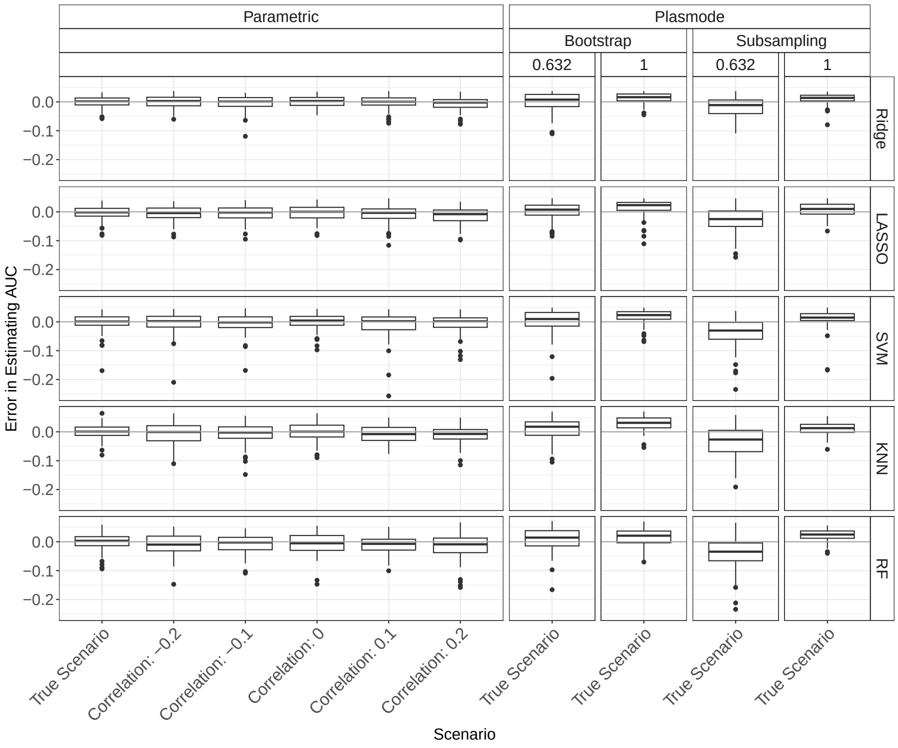

Fig E.8: Errors in the estimation of AUC in 100 iterations of a classification method comparison study per classifier for different simulation approaches with misspecified correlation for parametric simulation for $p = 10$.

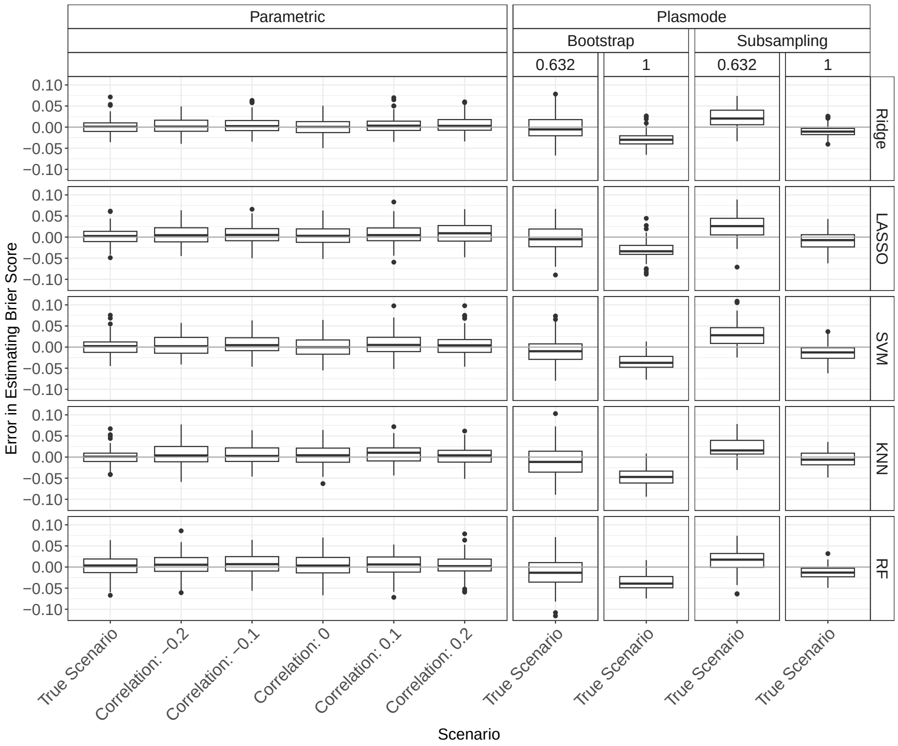

Fig E.9: Errors in the estimation of the Brier score in 100 iterations of a classification method comparison study per classifier for different simulation approaches with misspecified correlation for parametric simulation for $p = 10$.

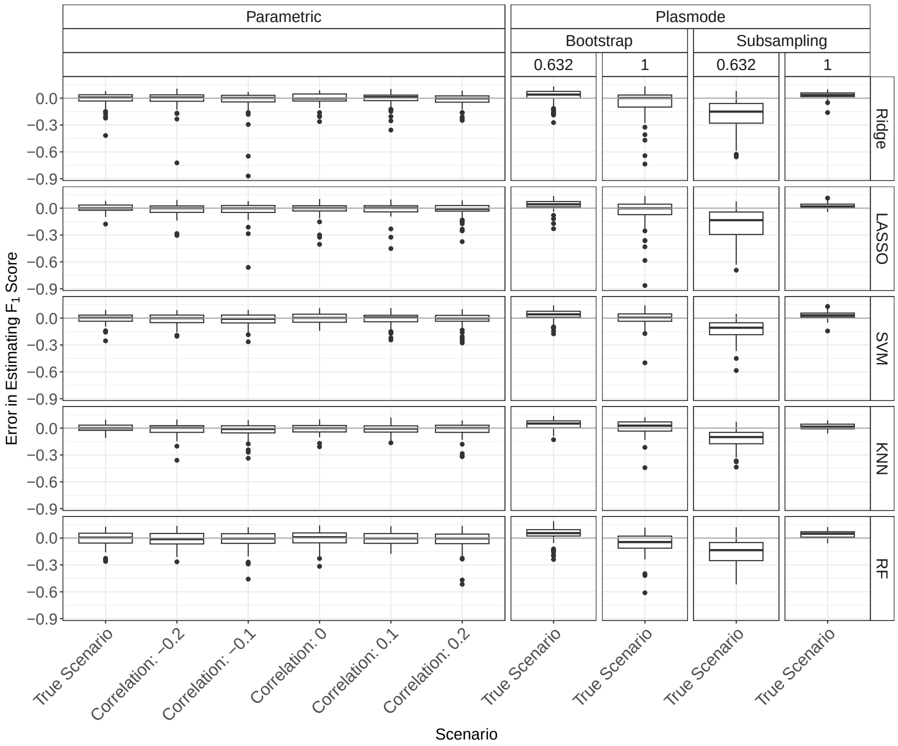

Fig E.10: Errors in the estimation of the F_1-score in 100 iterations of a classification method comparison study per classifier for different simulation approaches with misspecified correlation for parametric simulation for $p = 10$.

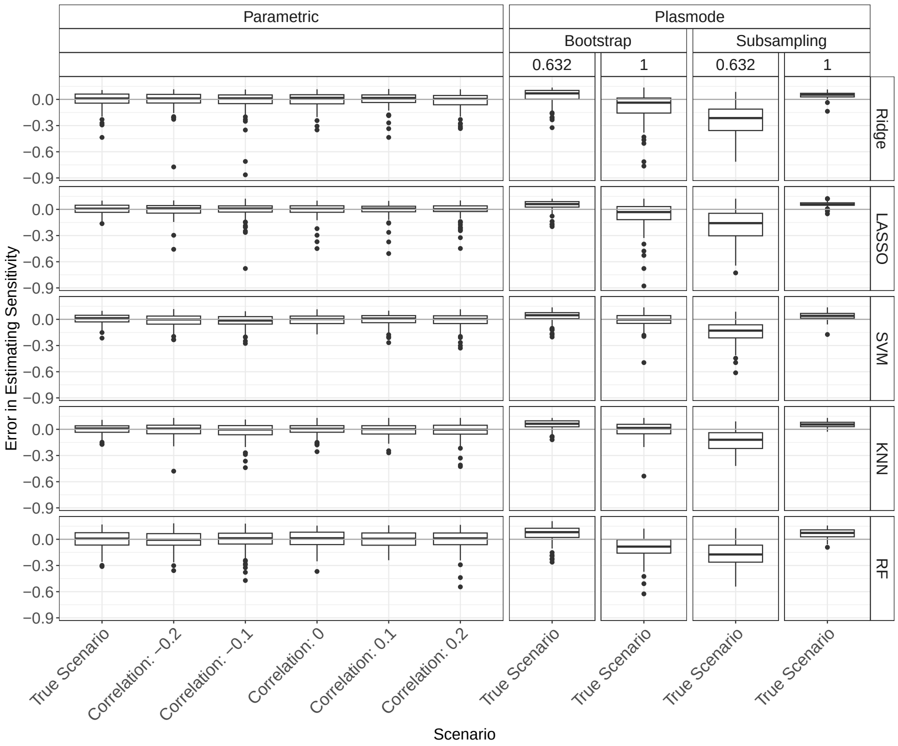

Fig E.11: Errors in the estimation of sensitivity in 100 iterations of a classification method comparison study per classifier for different simulation approaches with misspecified correlation for parametric simulation for $p = 10$.

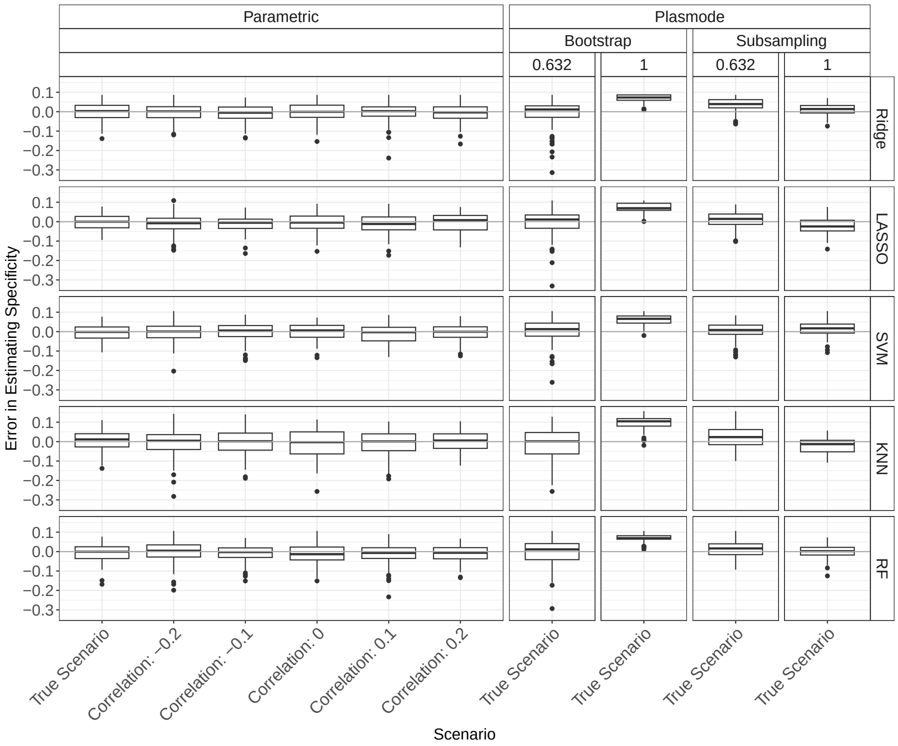

Fig E.12: Errors in the estimation of specificity in 100 iterations of a classification method comparison study per classifier for different simulation approaches with misspecified correlation for parametric simulation for $p = 10$.

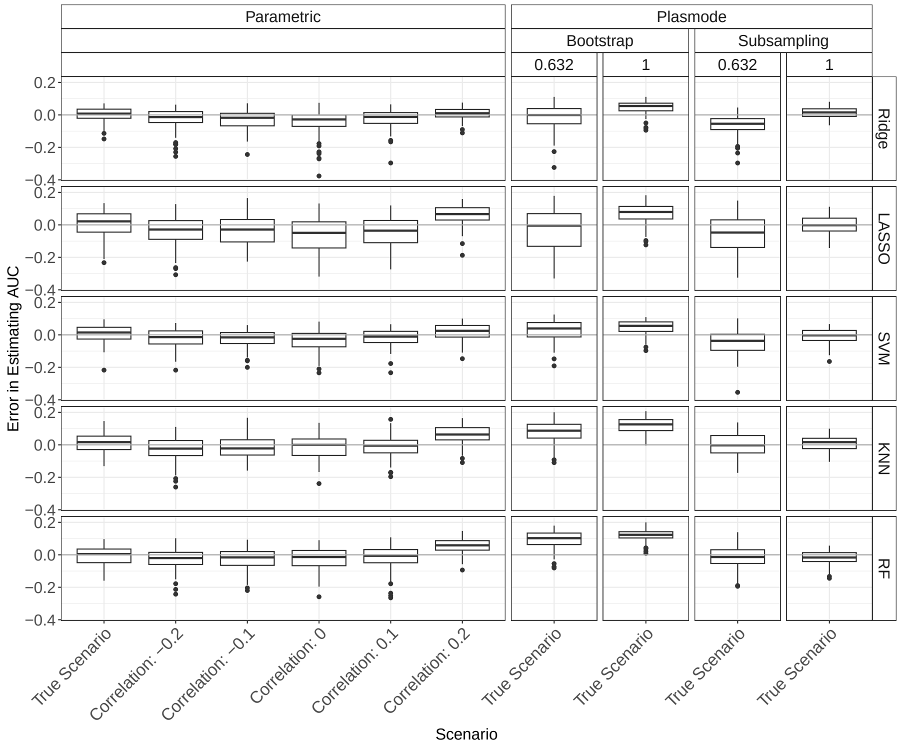

Fig E.13: Errors in the estimation of AUC in 100 iterations of a classification method comparison study per classifier for different simulation approaches with misspecified correlation for parametric simulation for $p = 50$.

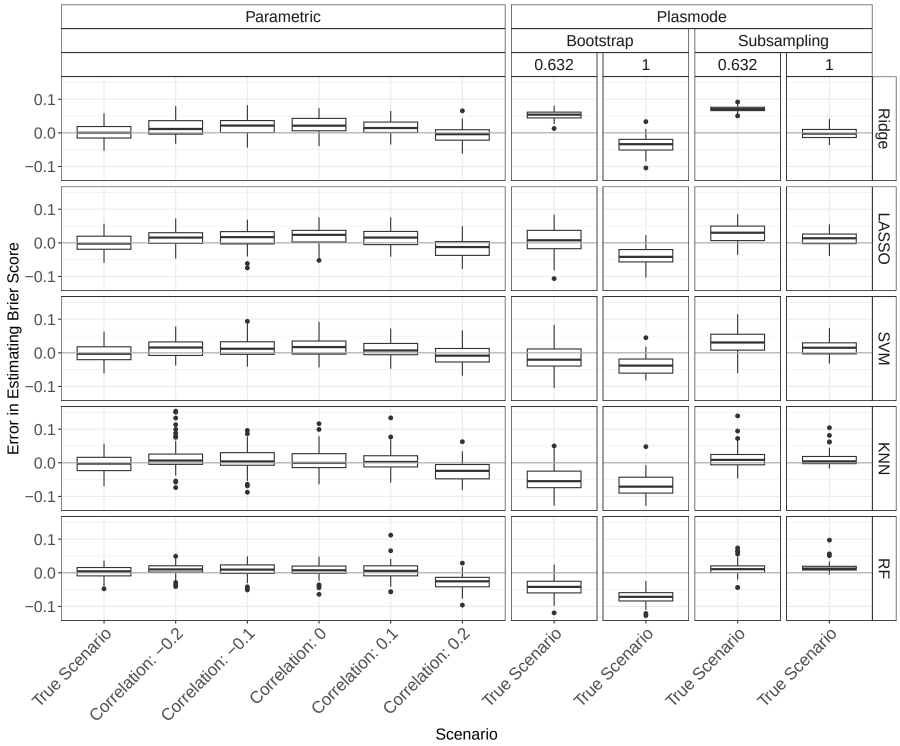

Fig E.14: Errors in the estimation of the Brier score in 100 iterations of a classification method comparison study per classifier for different simulation approaches with misspecified correlation for parametric simulation for $p = 50$.

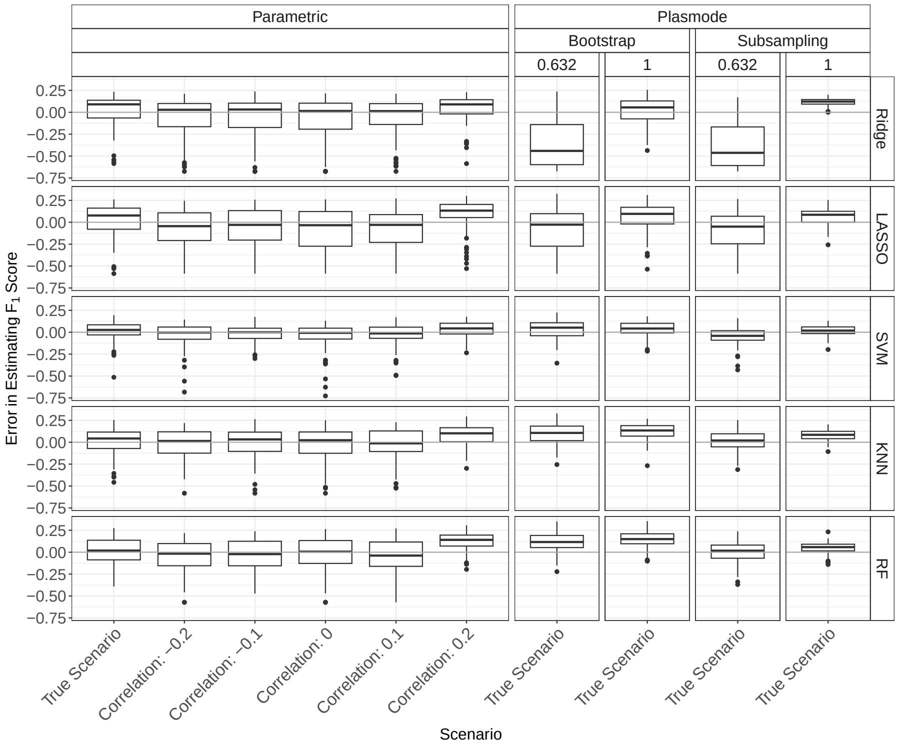

Fig E.15: Errors in the estimation of the F_1-score in 100 iterations of a classification method comparison study per classifier for different simulation approaches with misspecified correlation for parametric simulation for $p = 50$.

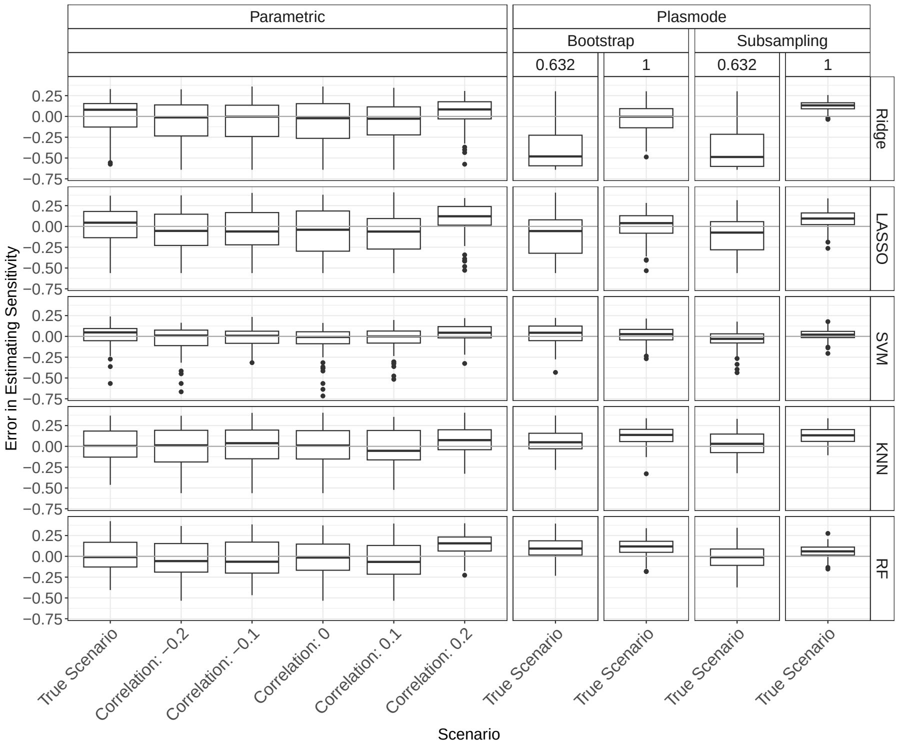

Fig E.16: Errors in the estimation of sensitivity in 100 iterations of a classification method comparison study per classifier for different simulation approaches with misspecified correlation for parametric simulation for $p = 50$.

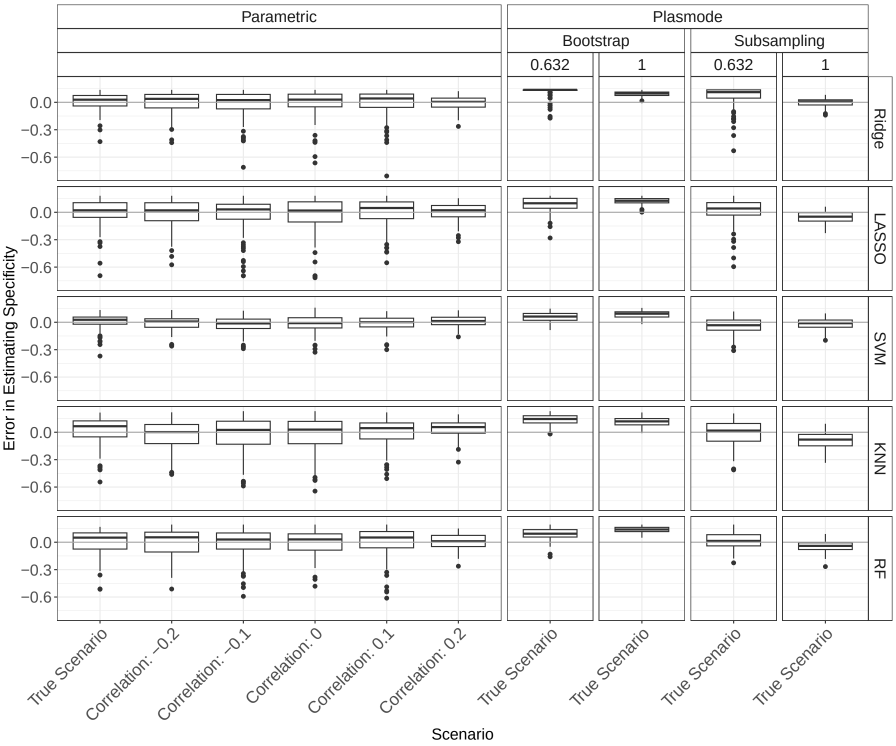

Fig E.17: Errors in the estimation of specificity in 100 iterations of a classification method comparison study per classifier for different simulation approaches with misspecified correlation for parametric simulation for $p = 50$.

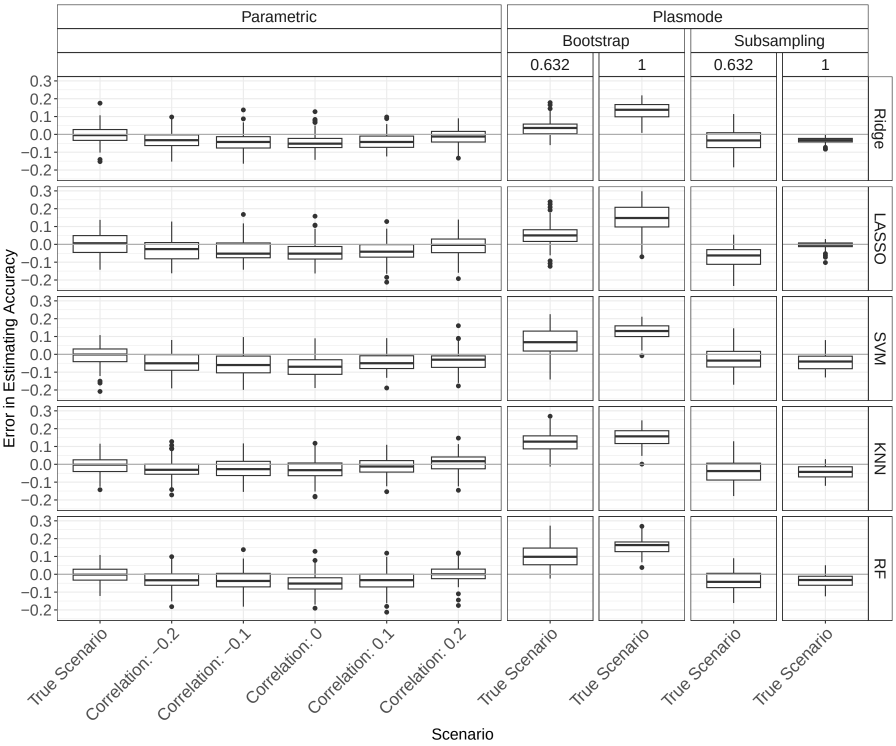

Fig E.18: Errors in the estimation of accuracy in 100 iterations of a classification method comparison study per classifier for different simulation approaches with misspecified correlation for parametric simulation for $p = 150$.

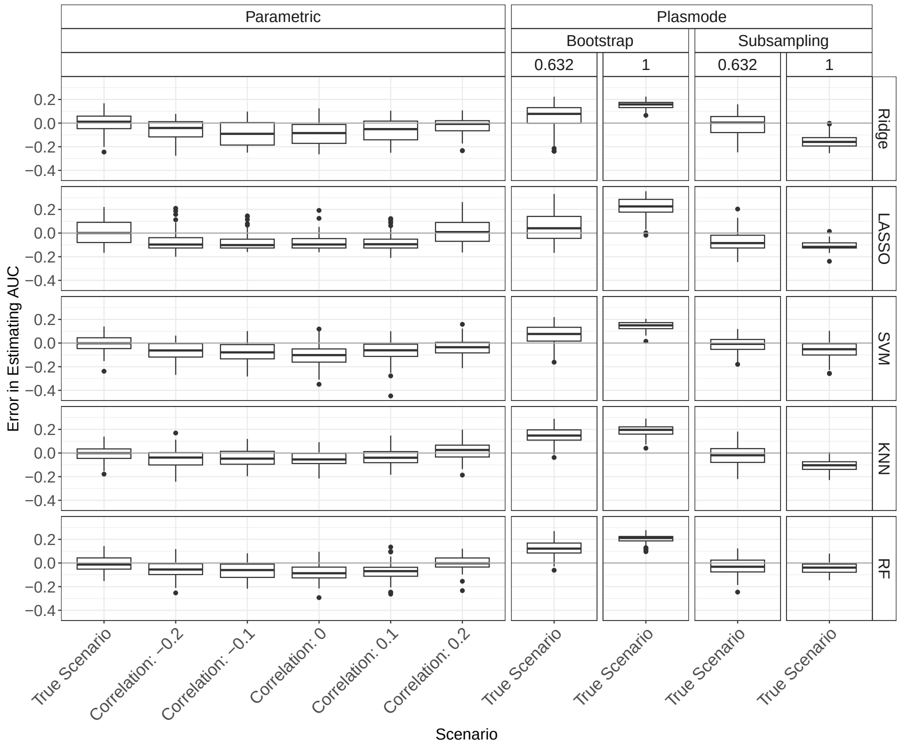

Fig E.19: Errors in the estimation of AUC in 100 iterations of a classification method comparison study per classifier for different simulation approaches with misspecified correlation for parametric simulation for $p = 150$.

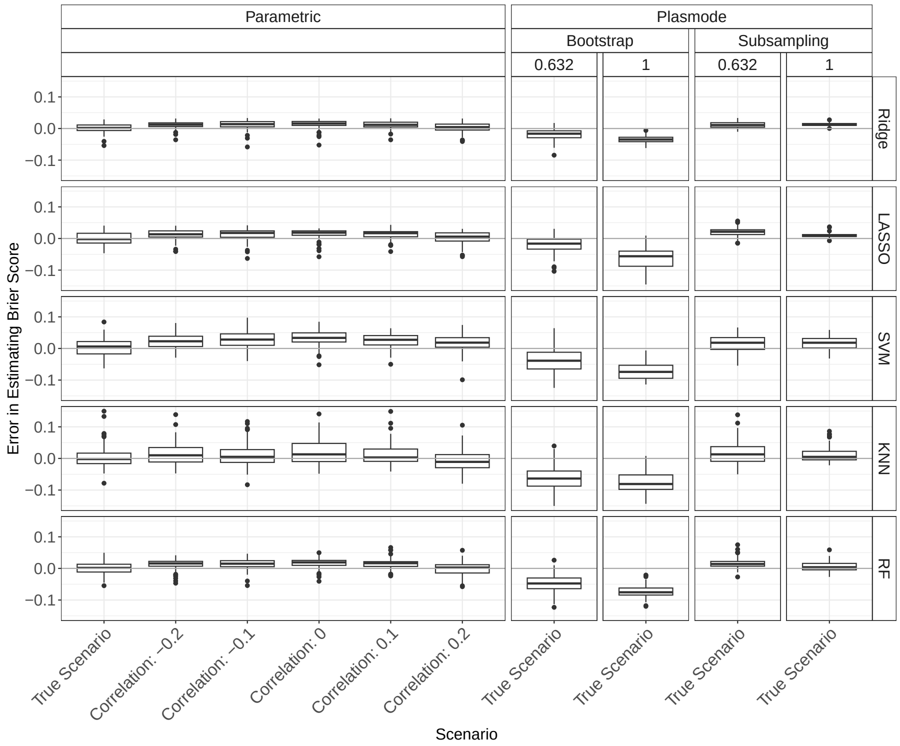

Fig E.20: Errors in the estimation of the Brier score in 100 iterations of a classification method comparison study per classifier for different simulation approaches with misspecified correlation for parametric simulation for $p = 150$.

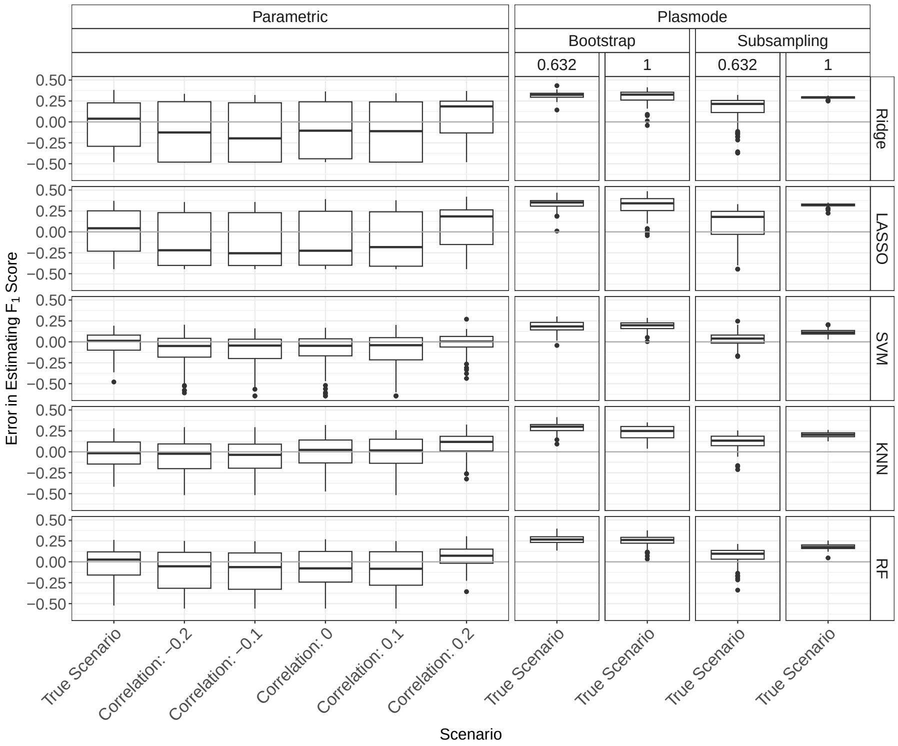

Fig E.21: Errors in the estimation of the F_1-score in 100 iterations of a classification method comparison study per classifier for different simulation approaches with misspecified correlation for parametric simulation for $p = 150$.

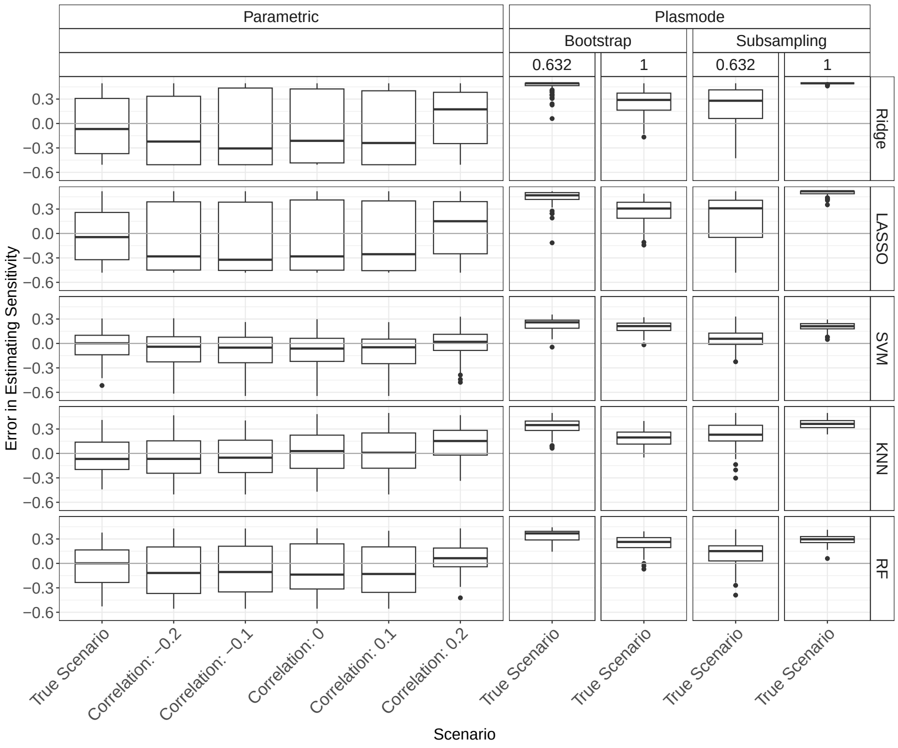

Fig E.22: Errors in the estimation of sensitivity in 100 iterations of a classification method comparison study per classifier for different simulation approaches with misspecified correlation for parametric simulation for $p = 150$.

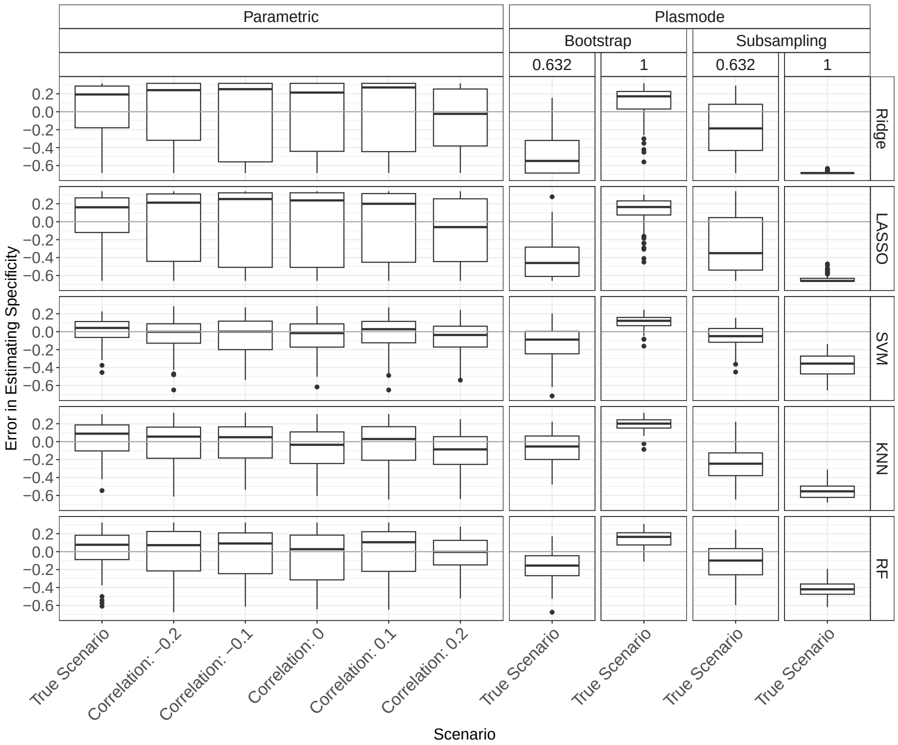

Fig E.23: Errors in the estimation of specificity in 100 iterations of a classification method comparison study per classifier for different simulation approaches with misspecified correlation for parametric simulation for $p = 150$.

F Standard Normal

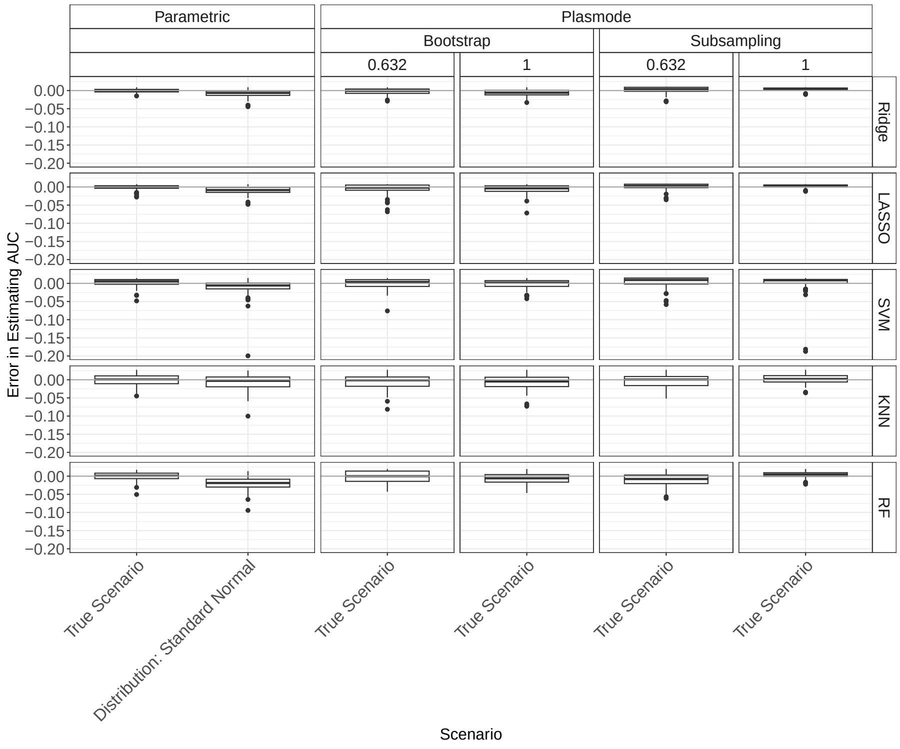

Fig F.1: Errors in the estimation of AUC in 100 iterations of a classification method comparison study per classifier for different simulation approaches with distribution misspecified as standard normal for parametric simulation for $p = 2$.

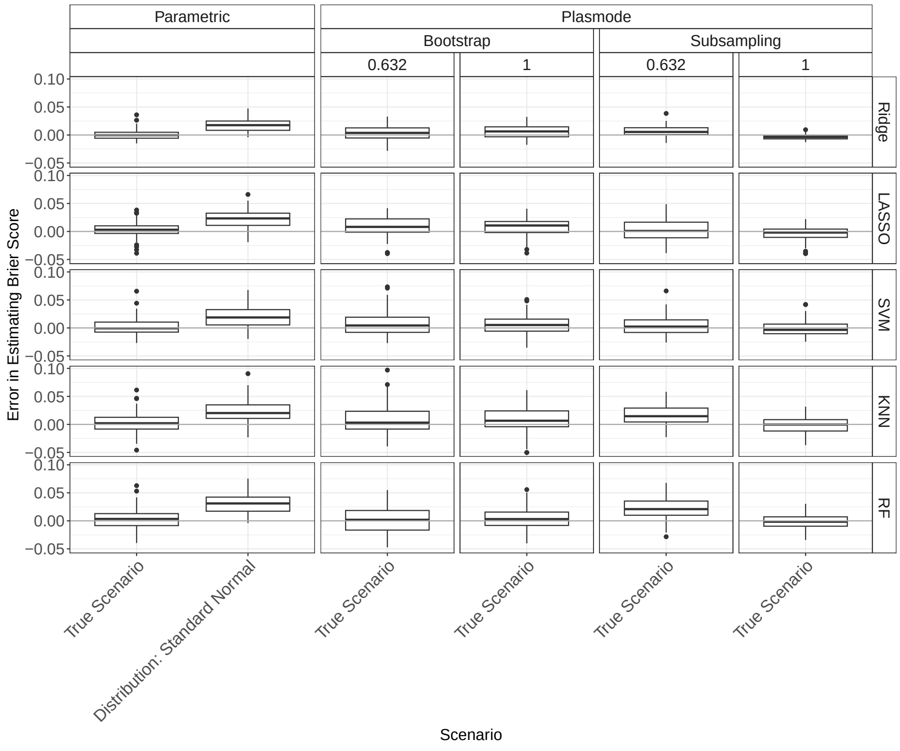

Fig F.2: Errors in the estimation of the Brier score in 100 iterations of a classification method comparison study per classifier for different simulation approaches with distribution misspecified as standard normal for parametric simulation for $p = 2$.

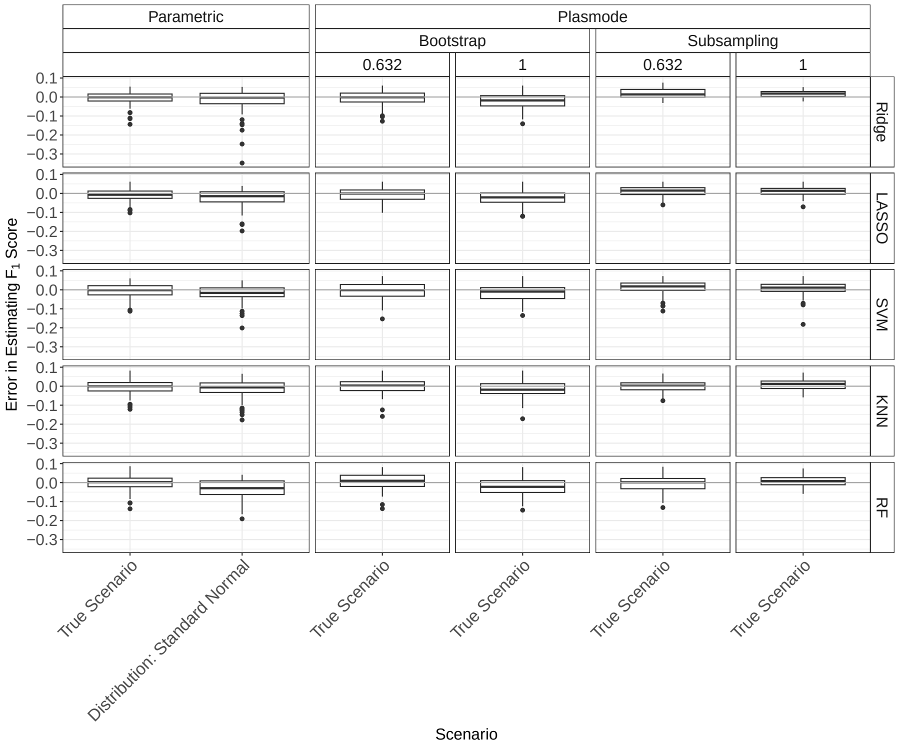

Fig F.3: Errors in the estimation of the F1 score in 100 iterations of a classification method comparison study per classifier for different simulation approaches with distribution misspecified as standard normal for parametric simulation for $p = 2$.

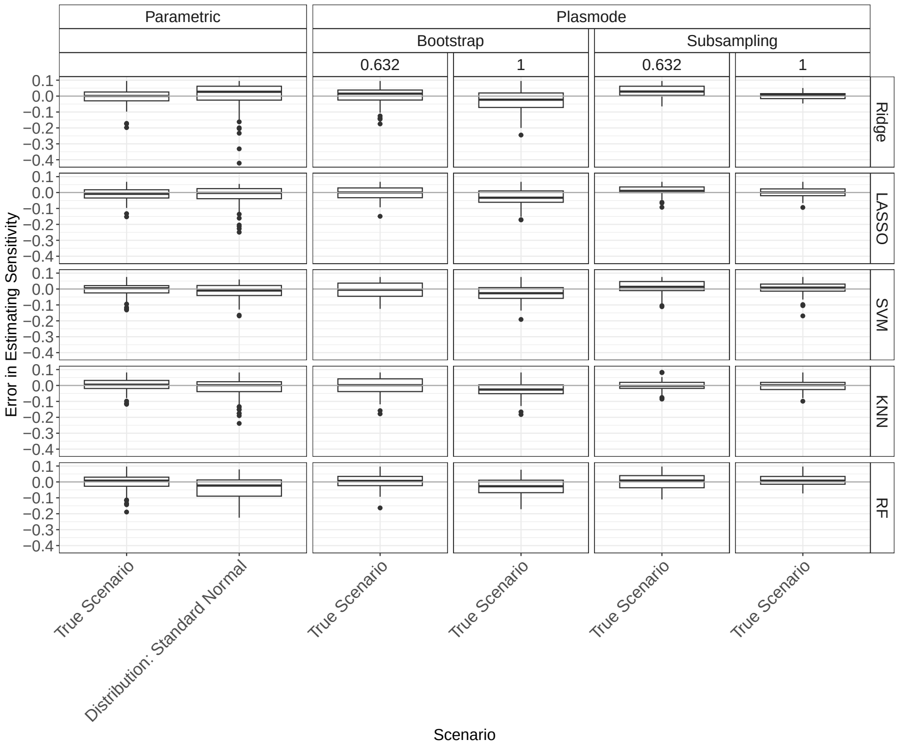

Fig F.4: Errors in the estimation of sensitivity in 100 iterations of a classification method comparison study per classifier for different simulation approaches with distribution misspecified as standard normal for parametric simulation for $p = 2$.

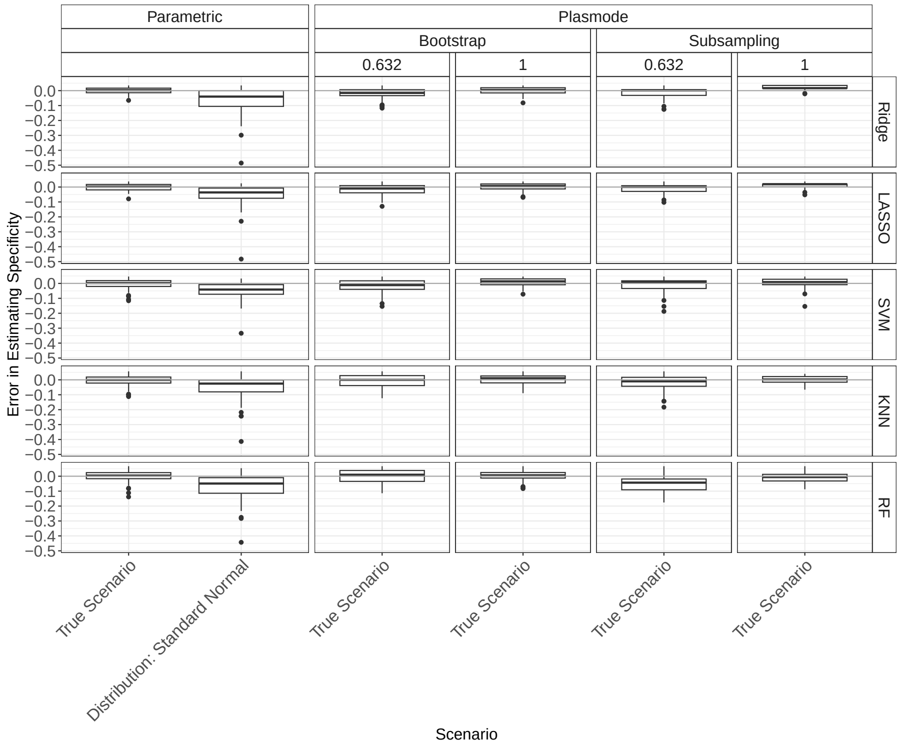

Fig F.5: Errors in the estimation of specificity in 100 iterations of a classification method comparison study per classifier for different simulation approaches with distribution misspecified as standard normal for parametric simulation for $p = 2$.

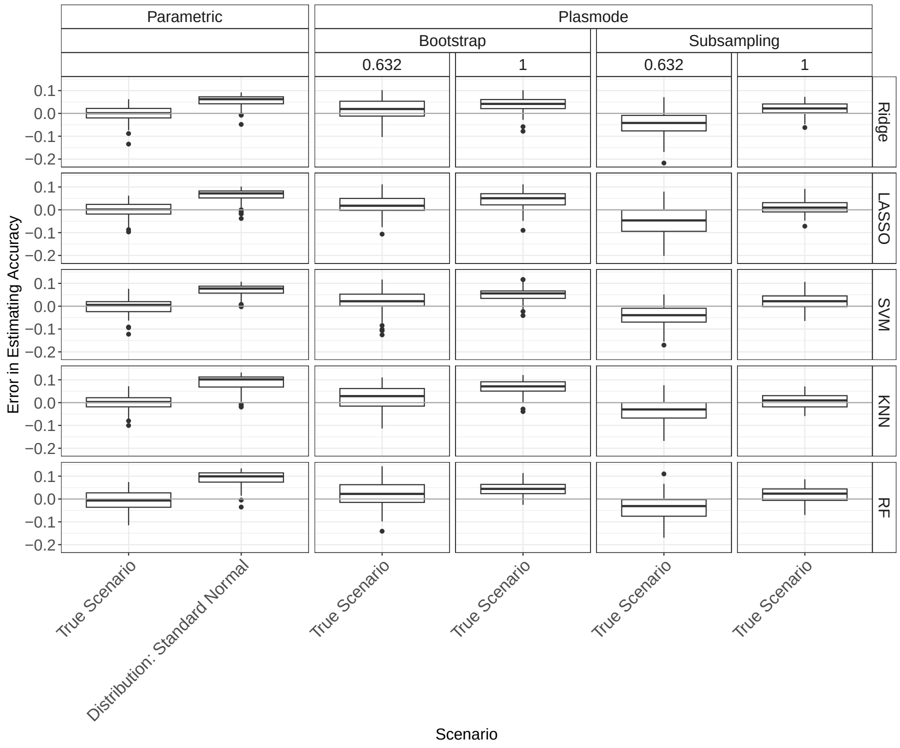

Fig F.6: Errors in the estimation of accuracy in 100 iterations of a classification method comparison study per classifier for different simulation approaches with distribution misspecified as standard normal for parametric simulation for $p = 10$.

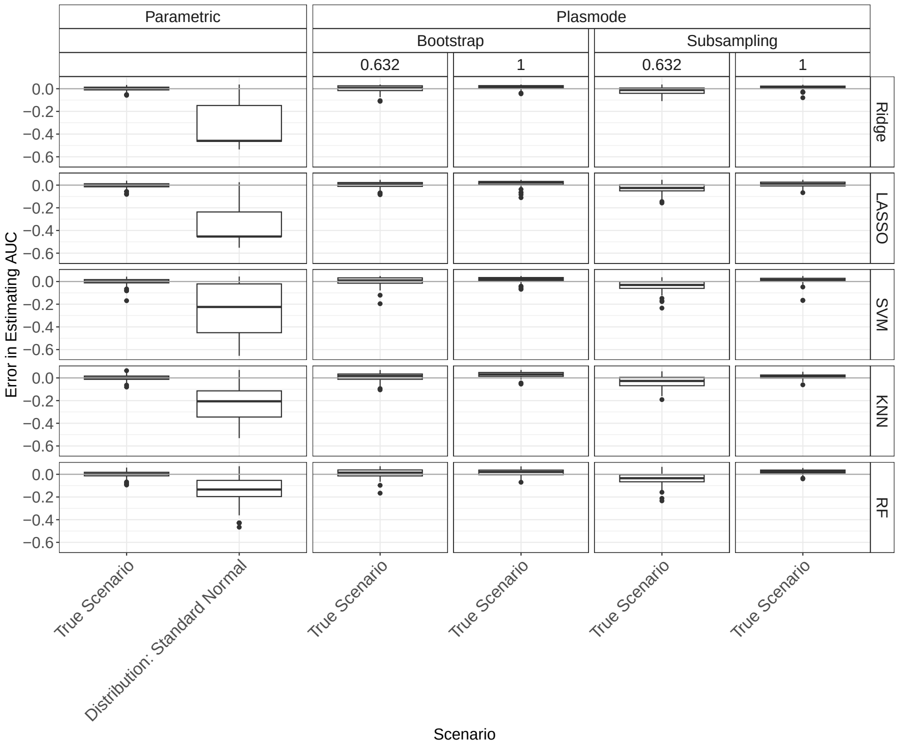

Fig F.7: Errors in the estimation of AUC in 100 iterations of a classification method comparison study per classifier for different simulation approaches with distribution misspecified as standard normal for parametric simulation for $p = 10$.

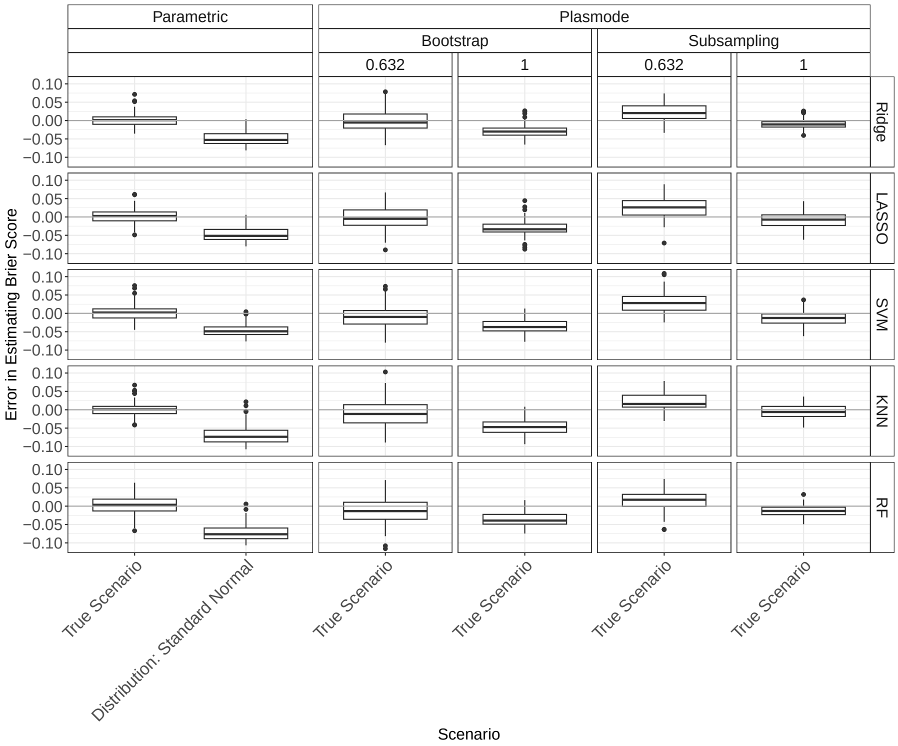

Fig F.8: Errors in the estimation of the Brier score in 100 iterations of a classification method comparison study per classifier for different simulation approaches with distribution misspecified as standard normal for parametric simulation for $p = 10$.

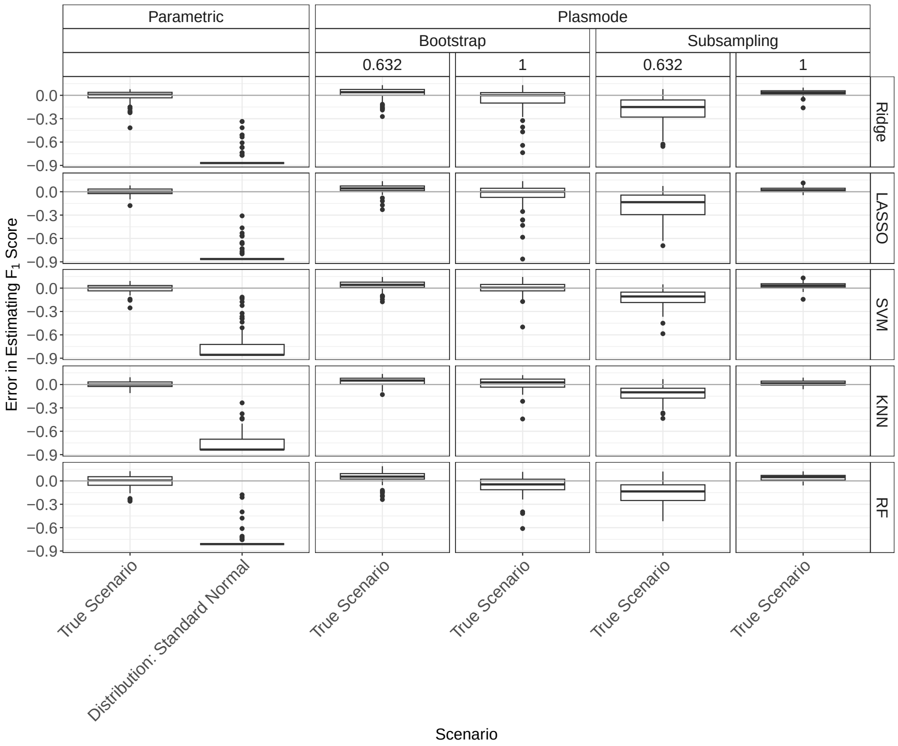

Fig F.9: Errors in the estimation of the F_1-score in 100 iterations of a classification method comparison study per classifier for different simulation approaches with distribution misspecified as standard normal for parametric simulation for $p = 10$.

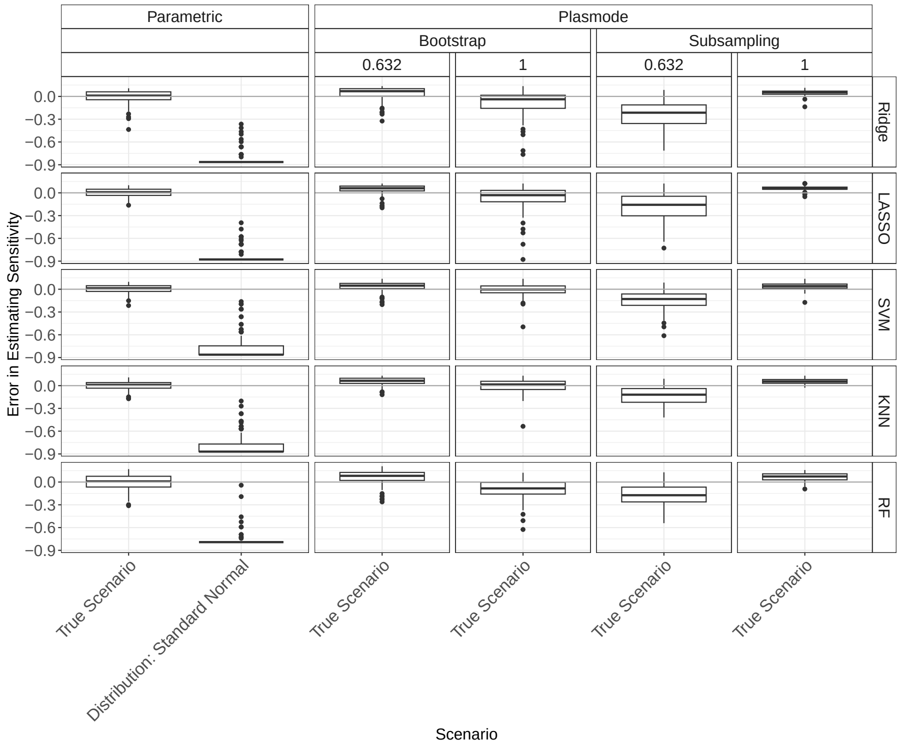

Fig F.10: Errors in the estimation of sensitivity in 100 iterations of a classification method comparison study per classifier for different simulation approaches with distribution misspecified as standard normal for parametric simulation for $p = 10$.

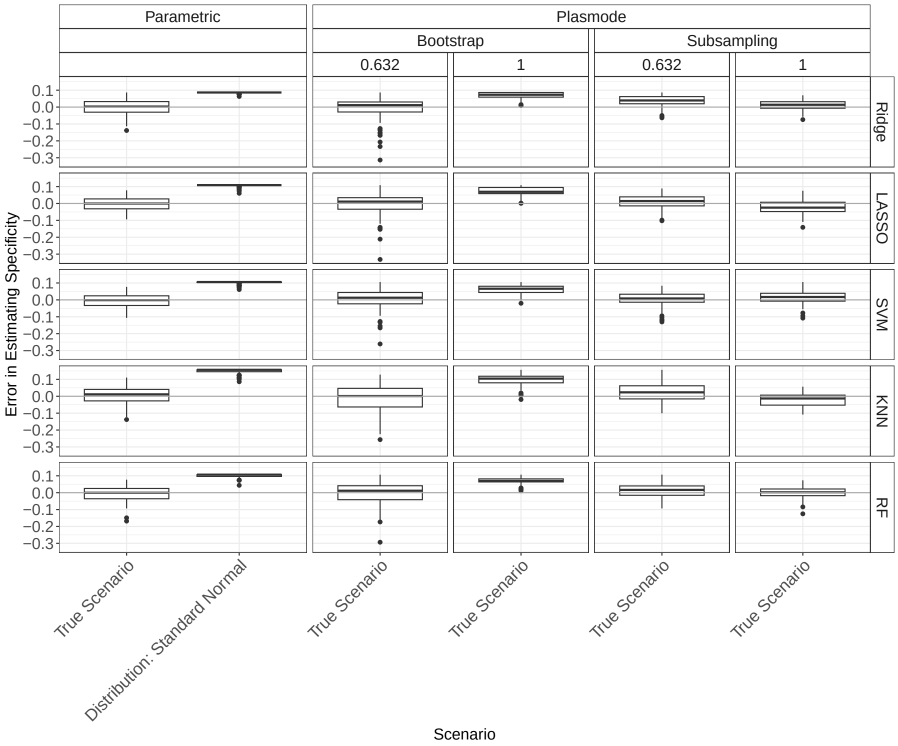

Fig F.11: Errors in the estimation of specificity in 100 iterations of a classification method comparison study per classifier for different simulation approaches with distribution misspecified as standard normal for parametric simulation for $p = 10$.

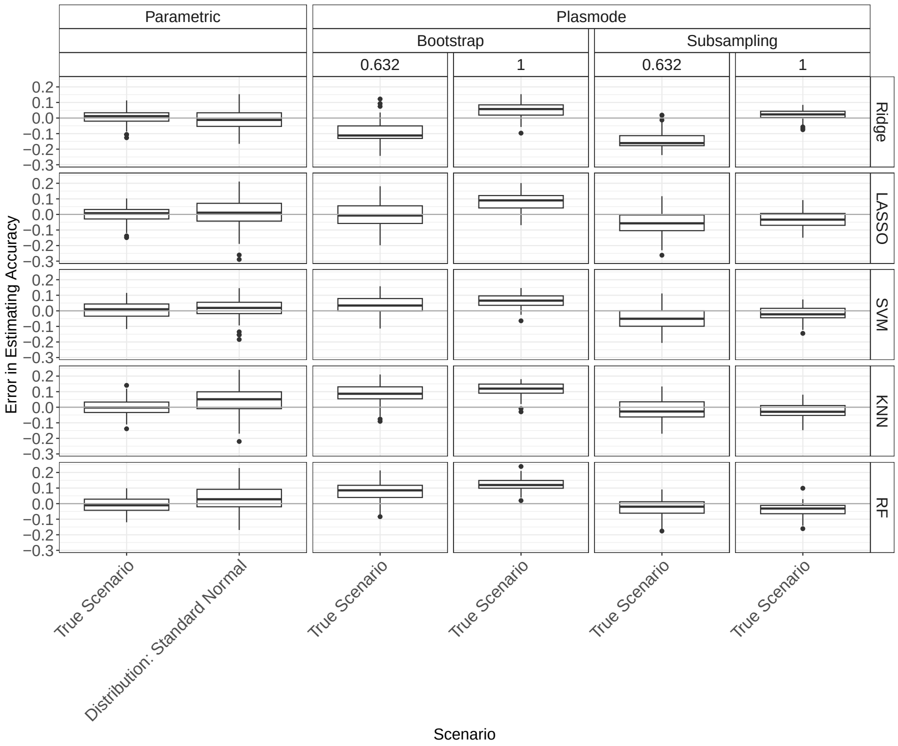

Fig F.12: Errors in the estimation of accuracy in 100 iterations of a classification method comparison study per classifier for different simulation approaches with distribution misspecified as standard normal for parametric simulation for $p = 50$.

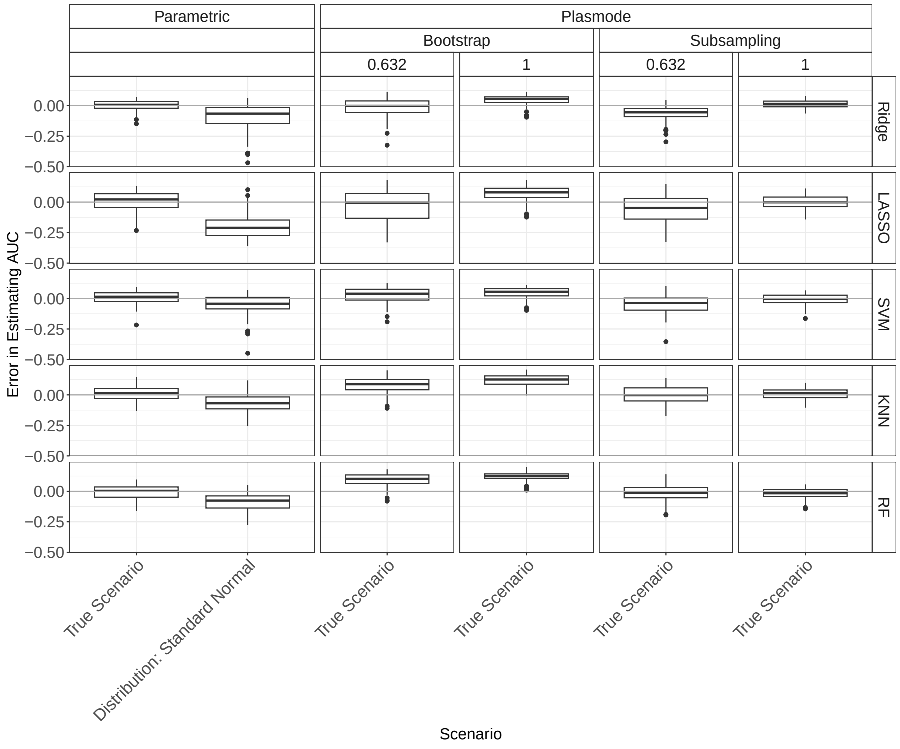

Fig F.13: Errors in the estimation of AUC in 100 iterations of a classification method comparison study per classifier for different simulation approaches with distribution misspecified as standard normal for parametric simulation for $p = 50$.

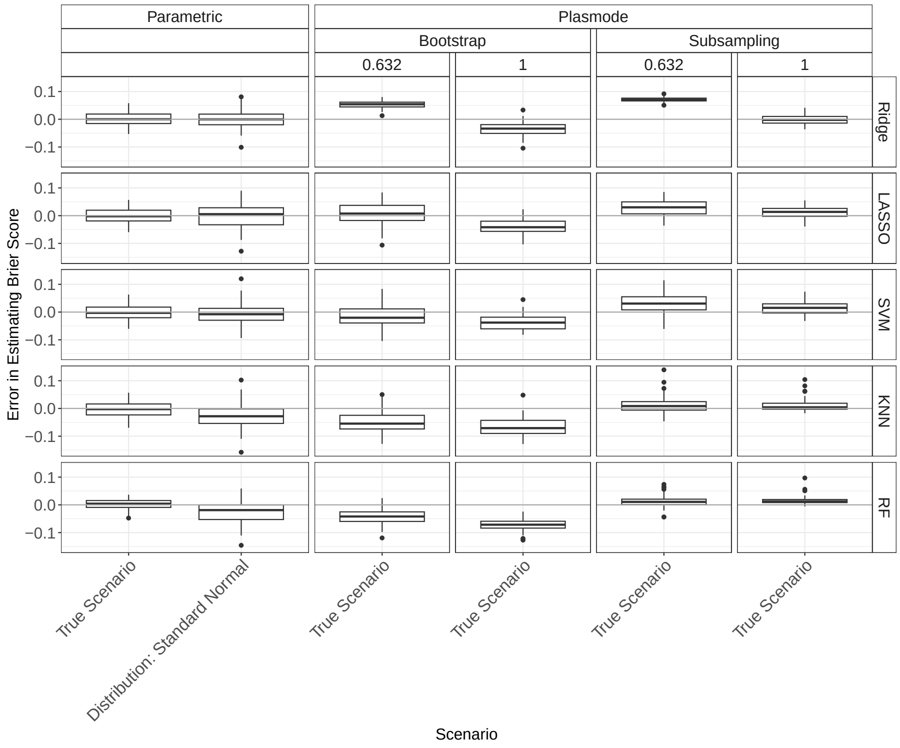

Fig F.14: Errors in the estimation of the Brier score in 100 iterations of a classification method comparison study per classifier for different simulation approaches with distribution misspecified as standard normal for parametric simulation for $p = 50$.

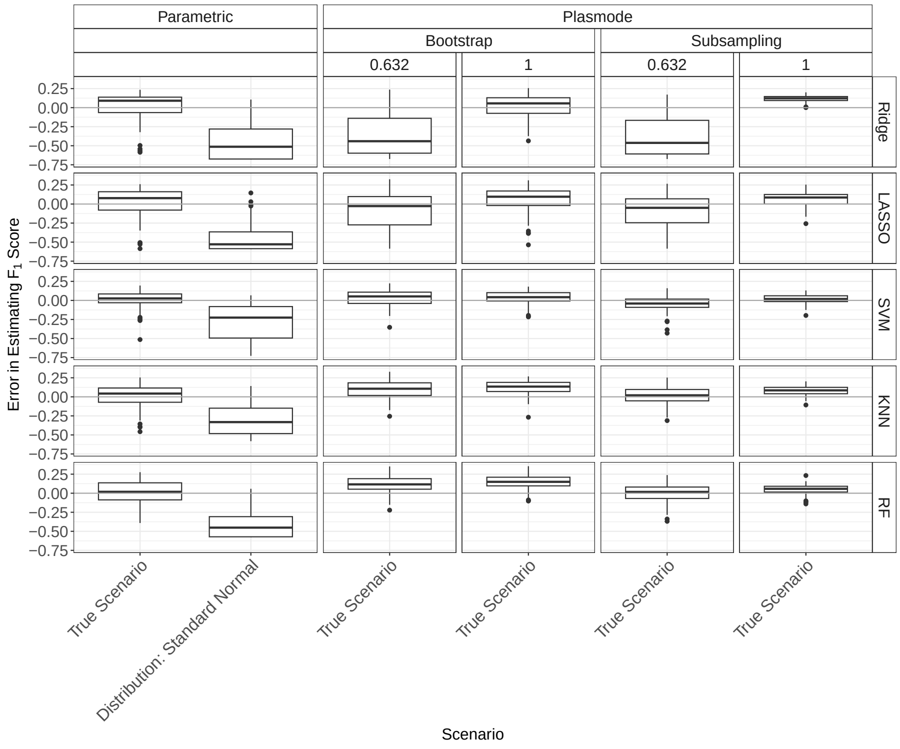

Fig F.15: Errors in the estimation of the F_1-score in 100 iterations of a classification method comparison study per classifier for different simulation approaches with distribution misspecified as standard normal for parametric simulation for $p = 50$.

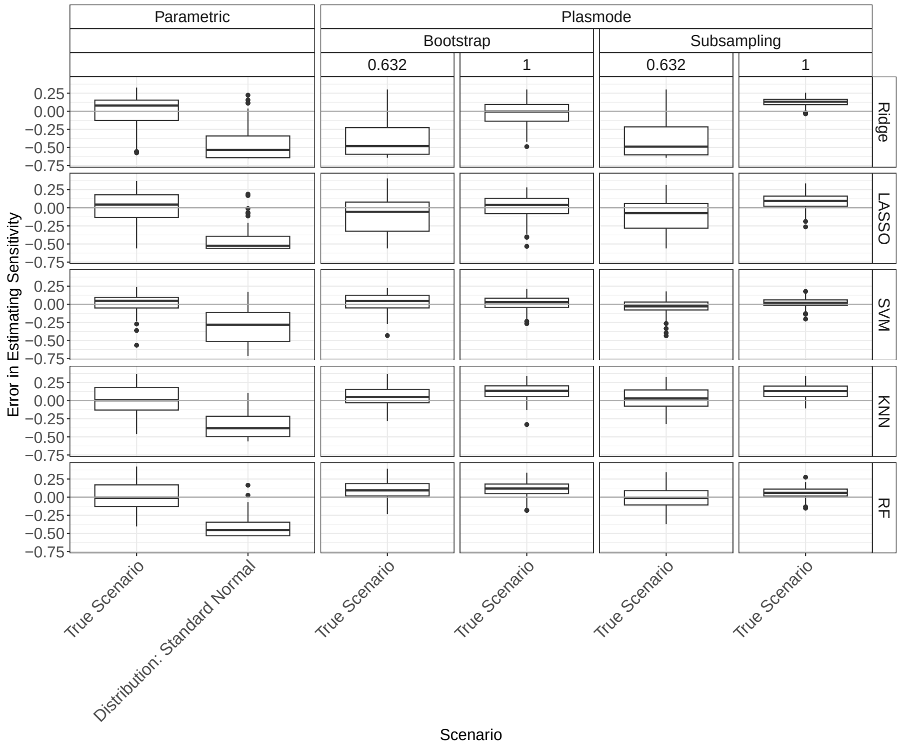

Fig F.16: Errors in the estimation of sensitivity in 100 iterations of a classification method comparison study per classifier for different simulation approaches with distribution misspecified as standard normal for parametric simulation for $p = 50$.

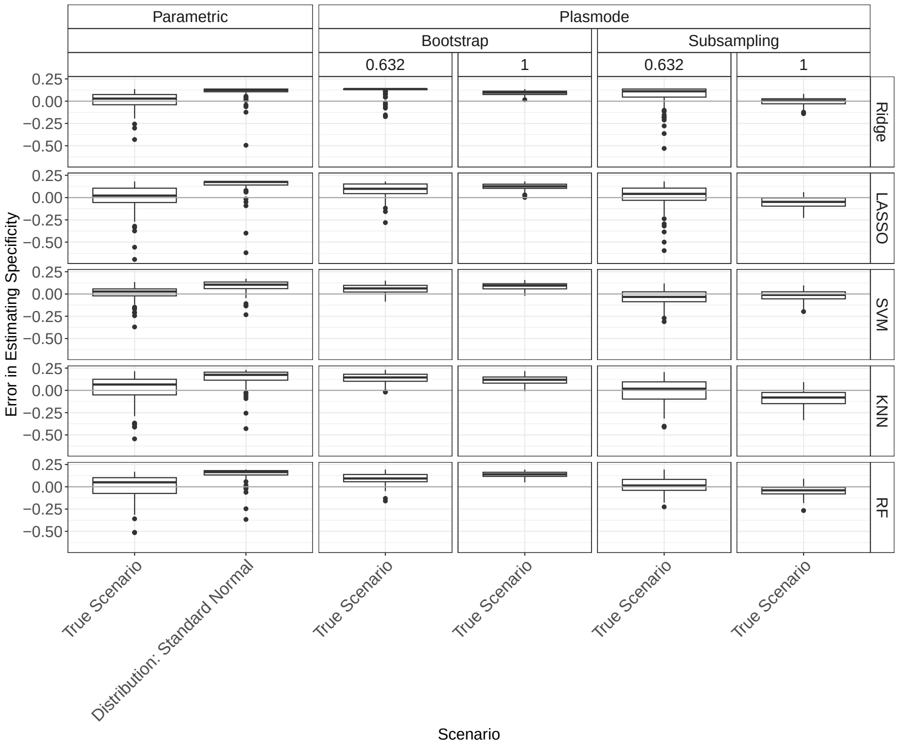

Fig F.17: Errors in the estimation of specificity in 100 iterations of a classification method comparison study per classifier for different simulation approaches with distribution misspecified as standard normal for parametric simulation for $p = 50$.

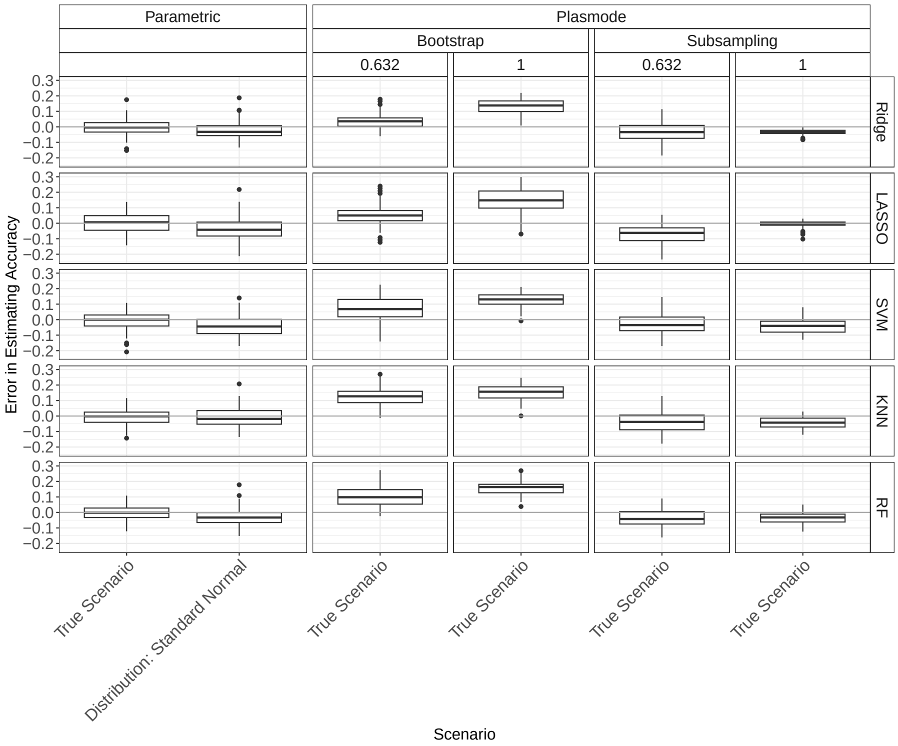

Fig F.18: Errors in the estimation of accuracy in 100 iterations of a classification method comparison study per classifier for different simulation approaches with distribution misspecified as standard normal for parametric simulation for $p = 150$.

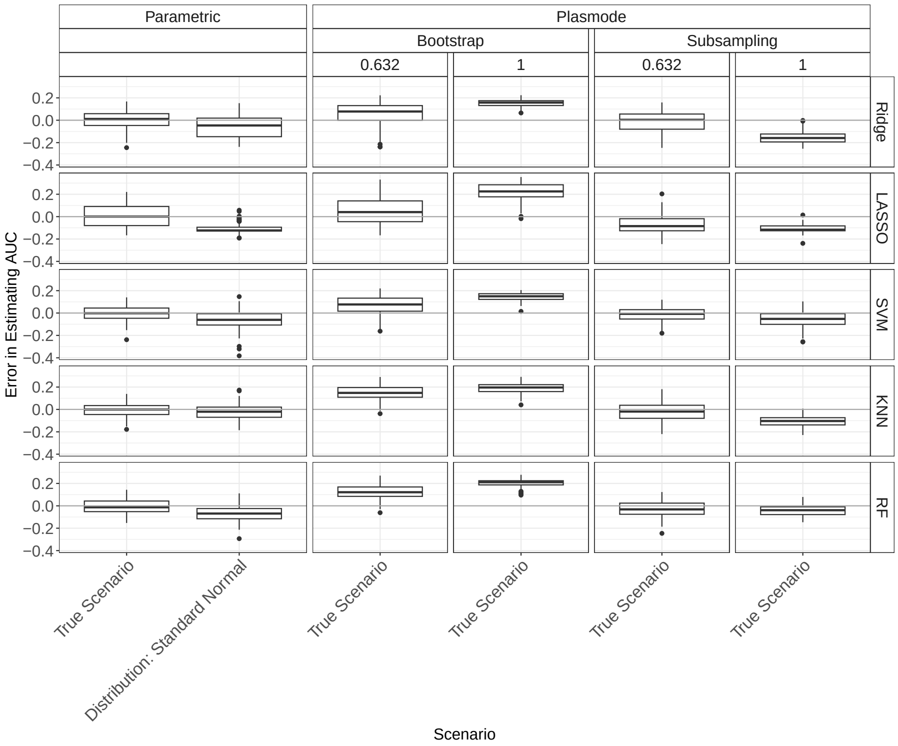

Fig F.19: Errors in the estimation of AUC in 100 iterations of a classification method comparison study per classifier for different simulation approaches with distribution misspecified as standard normal for parametric simulation for $p = 150$.

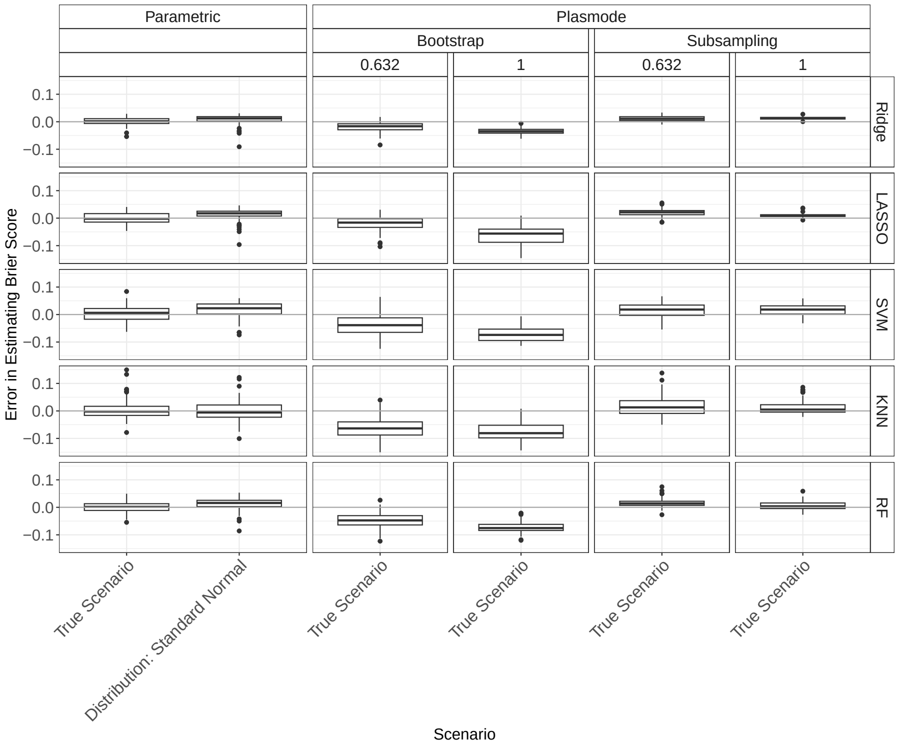

Fig F.20: Errors in the estimation of the Brier score in 100 iterations of a classification method comparison study per classifier for different simulation approaches with distribution misspecified as standard normal for parametric simulation for $p = 150$.

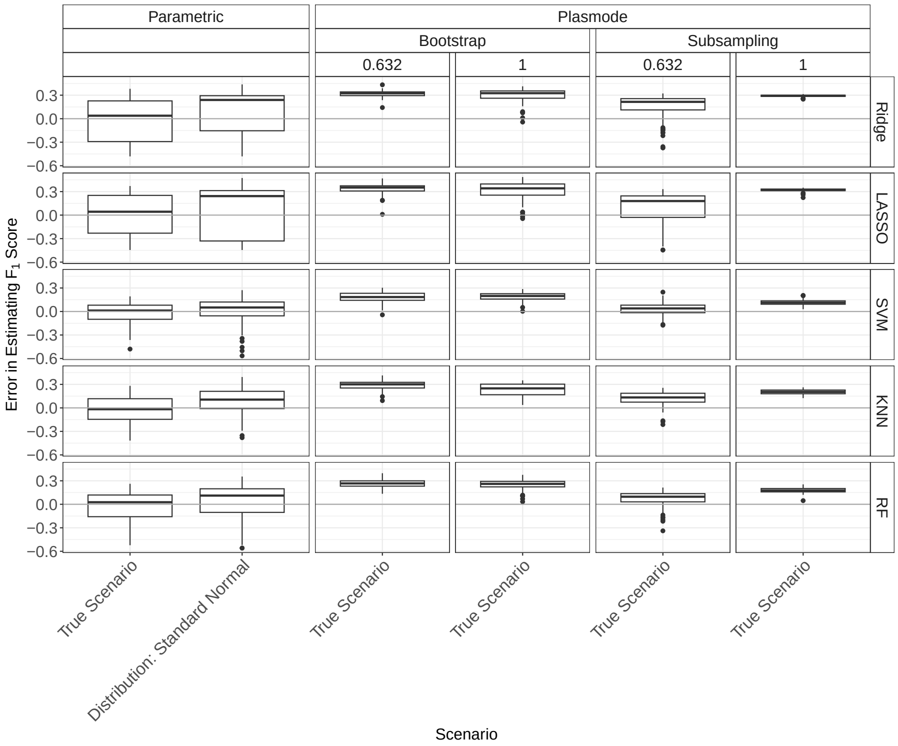

Fig F.21: Errors in the estimation of the F_1-score in 100 iterations of a classification method comparison study per classifier for different simulation approaches with distribution misspecified as standard normal for parametric simulation for $p = 150$.

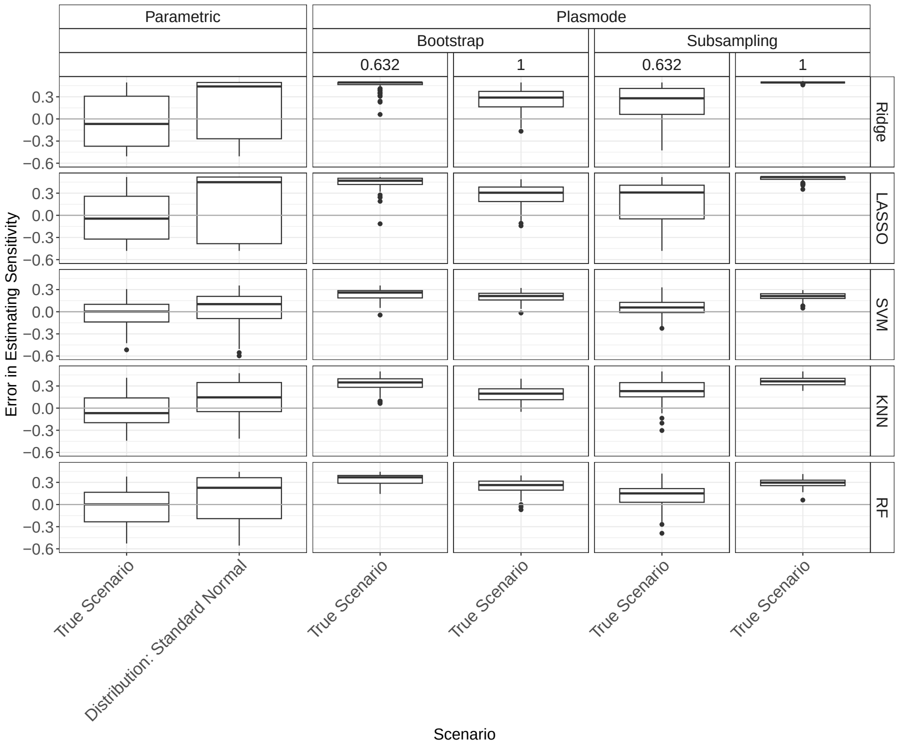

Fig F.22: Errors in the estimation of sensitivity in 100 iterations of a classification method comparison study per classifier for different simulation approaches with distribution misspecified as standard normal for parametric simulation for $p = 150$.

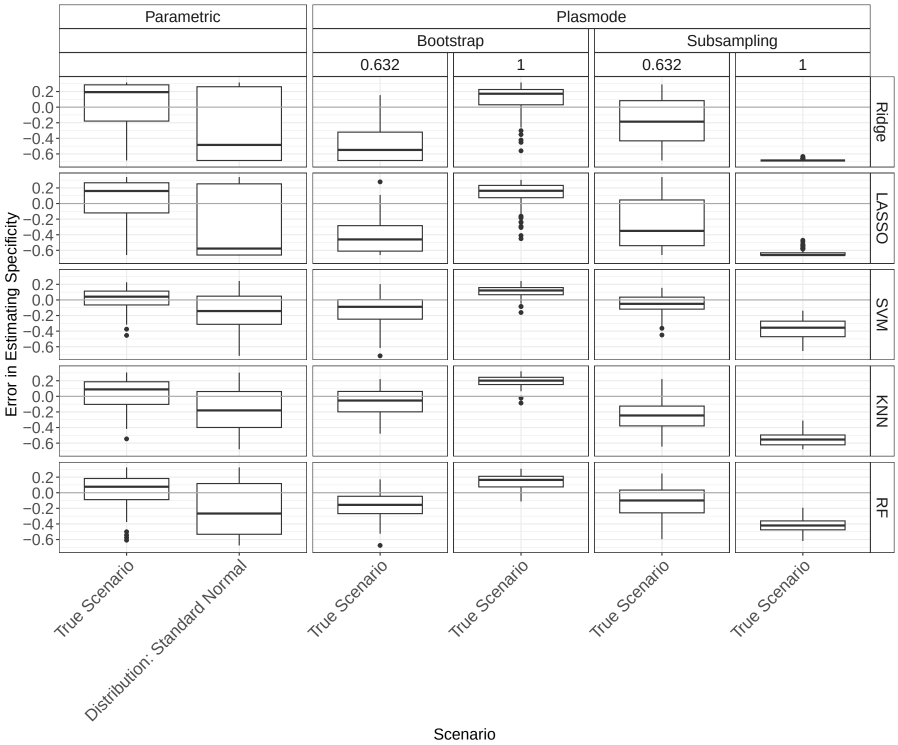

Fig F.23: Errors in the estimation of specificity in 100 iterations of a classification method comparison study per classifier for different simulation approaches with distribution misspecified as standard normal for parametric simulation for $p = 150$.

G OGM

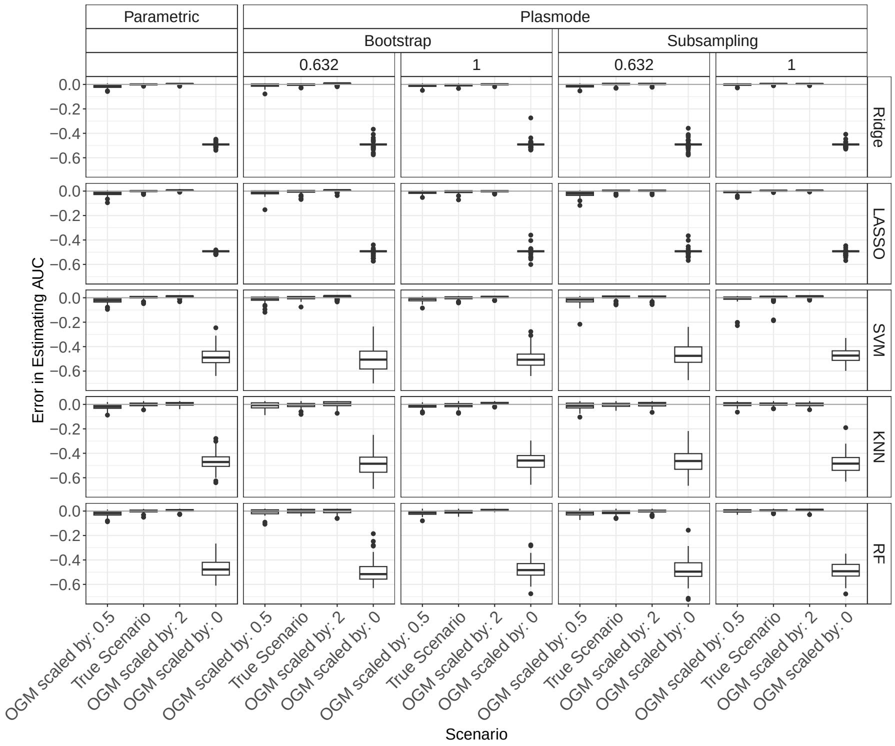

Fig G.1: Errors in the estimation of AUC in 100 iterations of a classification method comparison study per classifier for different simulation approaches with misspecifications of the OGM for $p = 2$.

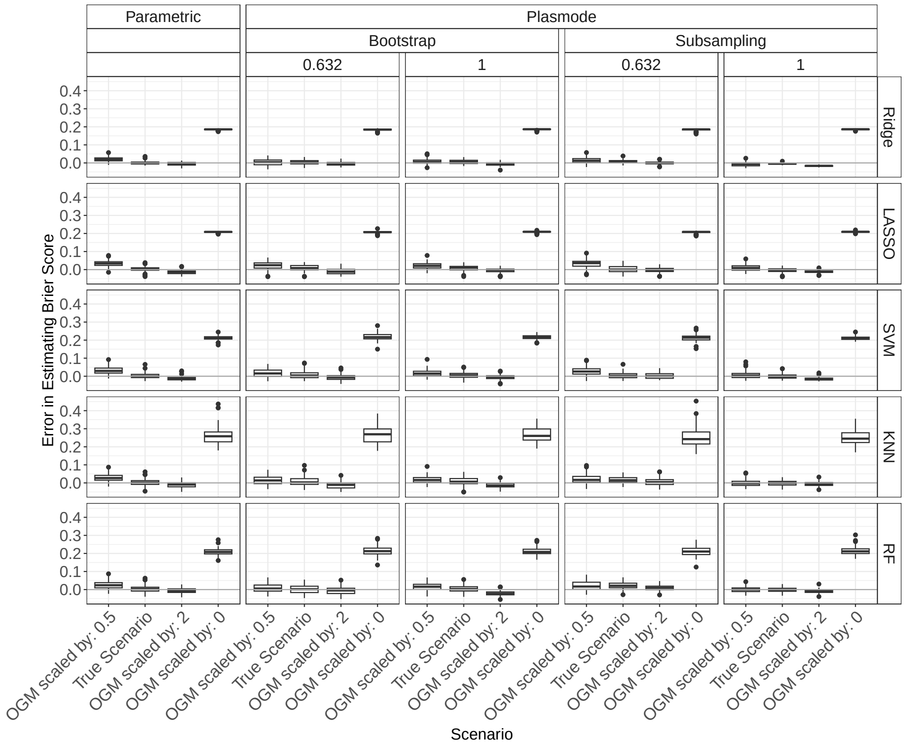

Fig G.2: Errors in the estimation of the Brier score in 100 iterations of a classification method comparison study per classifier for different simulation approaches with misspecifications of the OGM for $p = 2$.

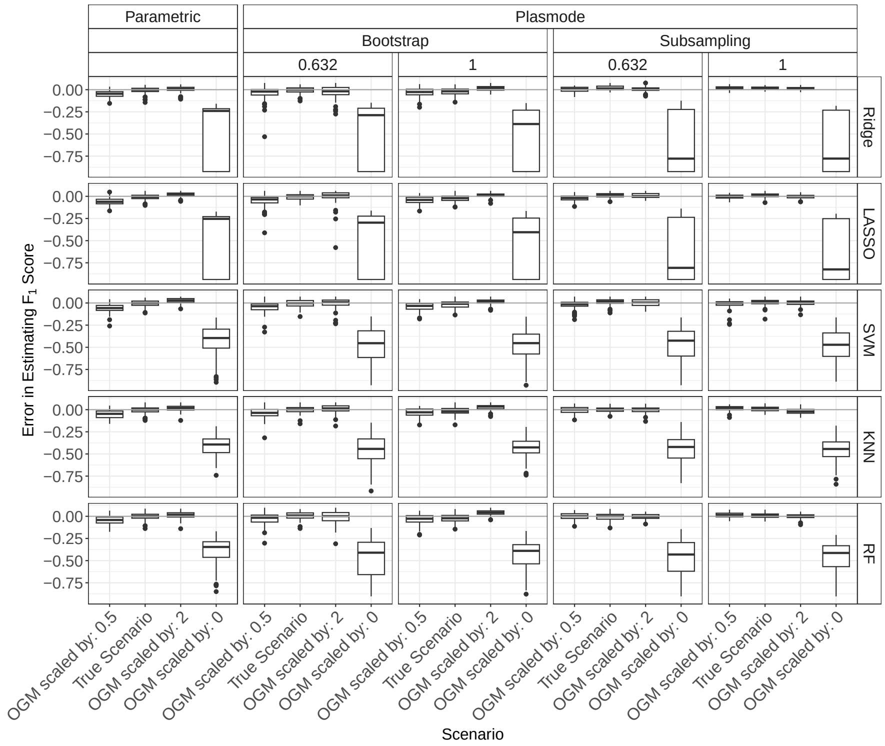

Fig G.3: Errors in the estimation of the F1 score in 100 iterations of a classification method comparison study per classifier for different simulation approaches with misspecifications of the OGM for $p = 2$.

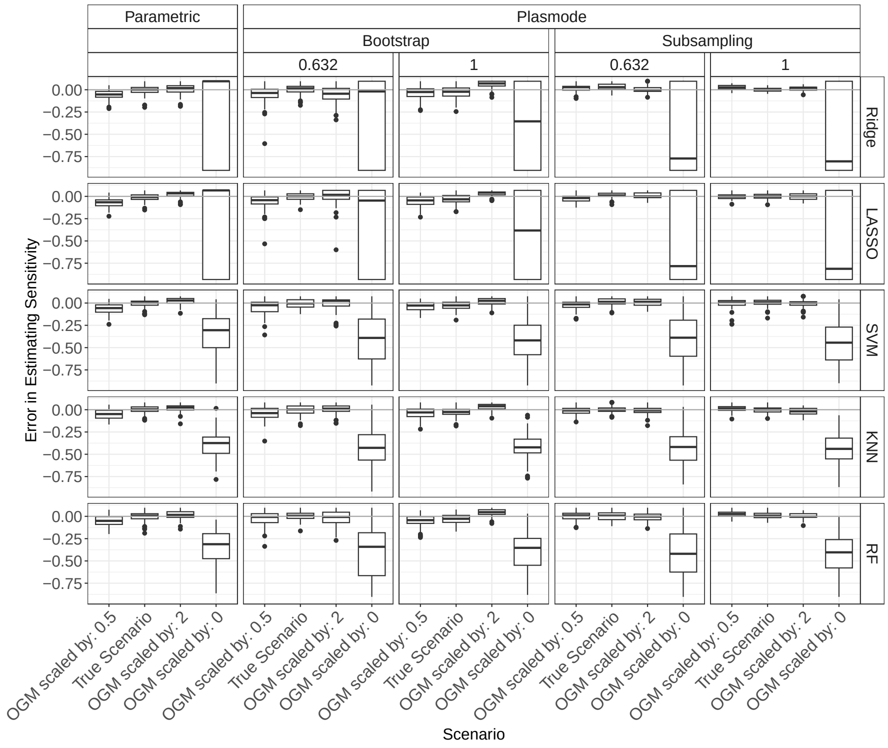

Fig G.4: Errors in the estimation of sensitivity in 100 iterations of a classification method comparison study per classifier for different simulation approaches with misspecifications of the OGM for $p = 2$.

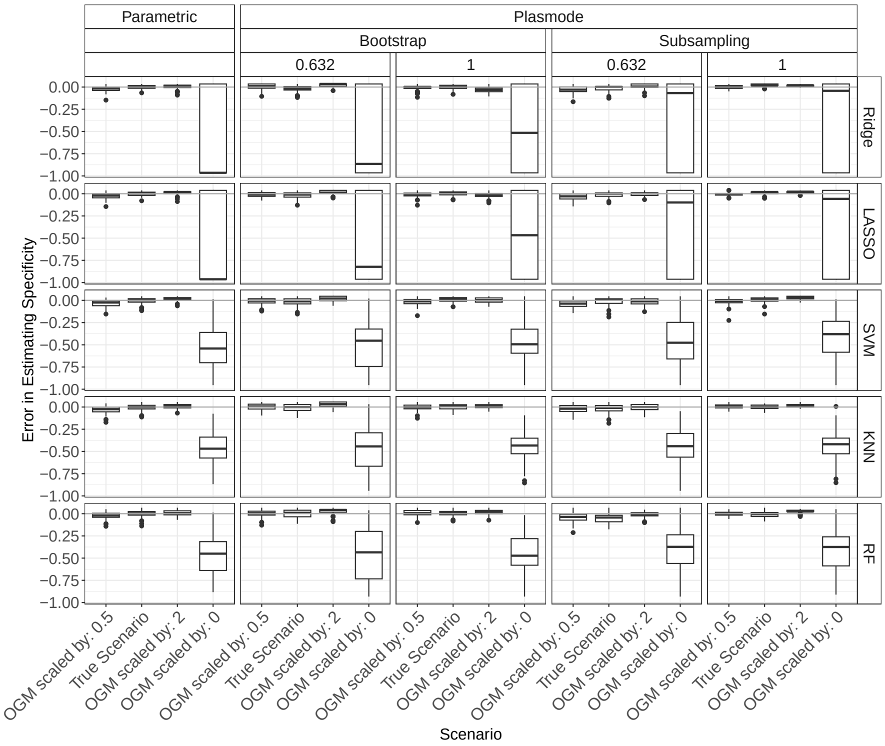

Fig G.5: Errors in the estimation of specificity in 100 iterations of a classification method comparison study per classifier for different simulation approaches with misspecifications of the OGM for $p = 2$.

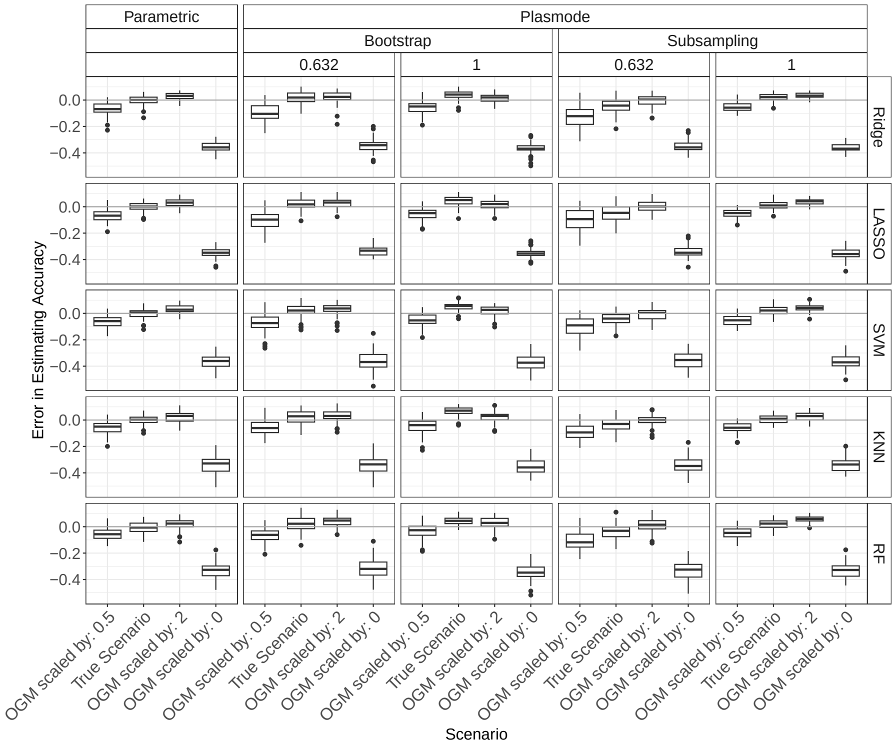

Fig G.6: Errors in the estimation of accuracy in 100 iterations of a classification method comparison study per classifier for different simulation approaches with misspecifications of the OGM for $p = 10$.

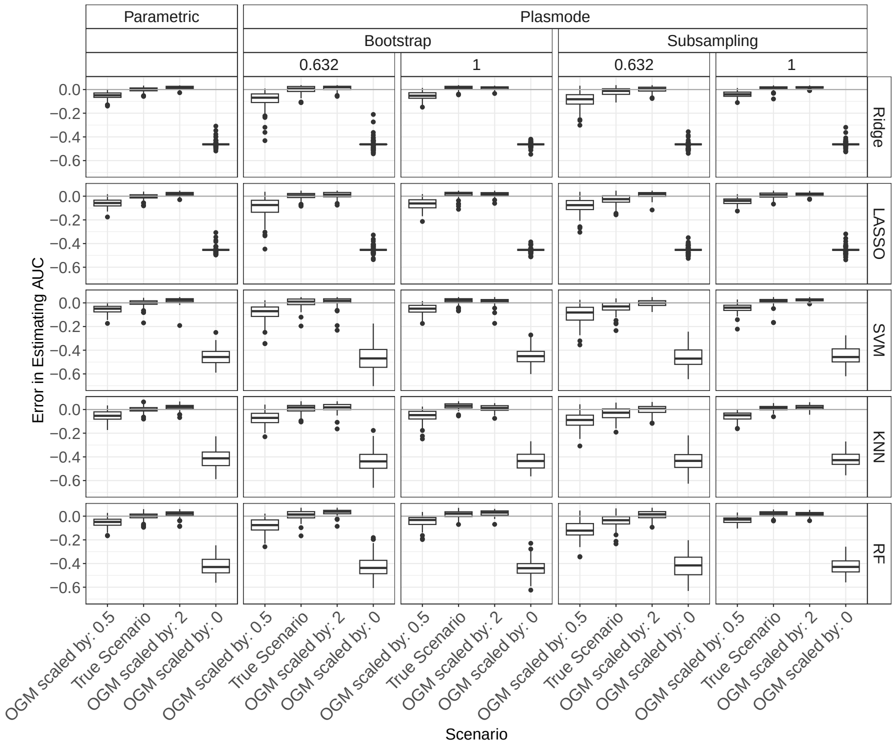

Fig G.7: Errors in the estimation of AUC in 100 iterations of a classification method comparison study per classifier for different simulation approaches with misspecifications of the OGM for $p = 10$.

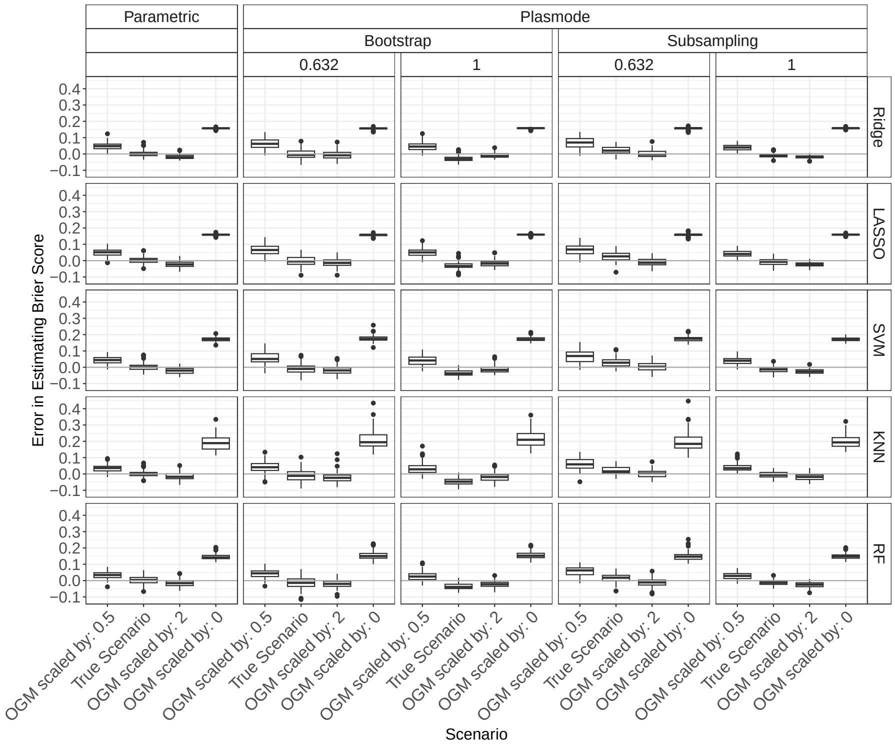

Fig G.8: Errors in the estimation of the Brier score in 100 iterations of a classification method comparison study per classifier for different simulation approaches with misspecifications of the OGM for $p = 10$.

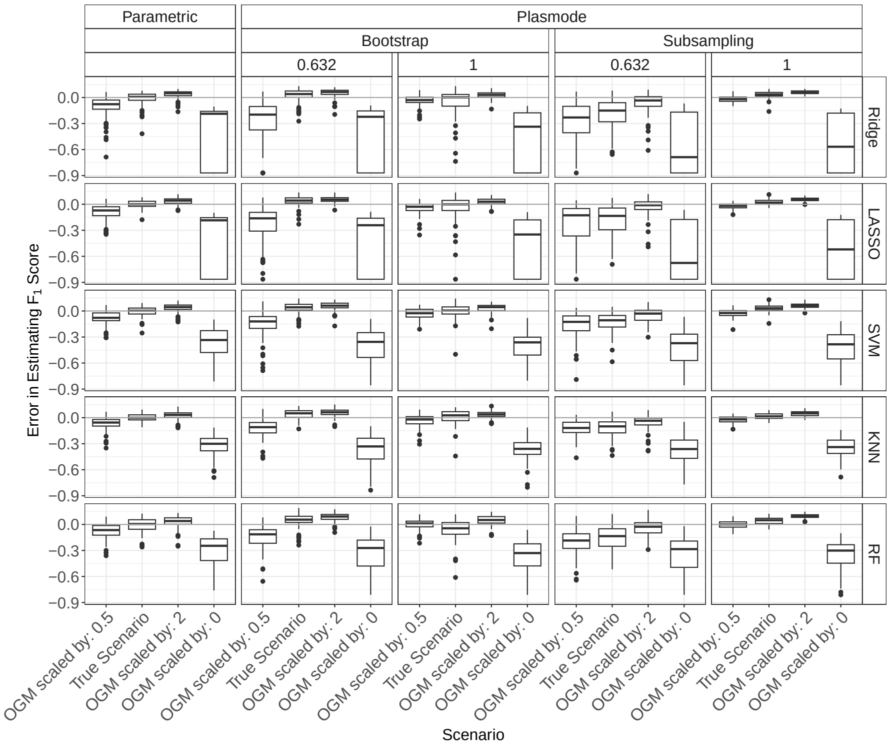

Fig G.9: Errors in the estimation of the F_1-score in 100 iterations of a classification method comparison study per classifier for different simulation approaches with misspecifications of the OGM for $p = 10$.

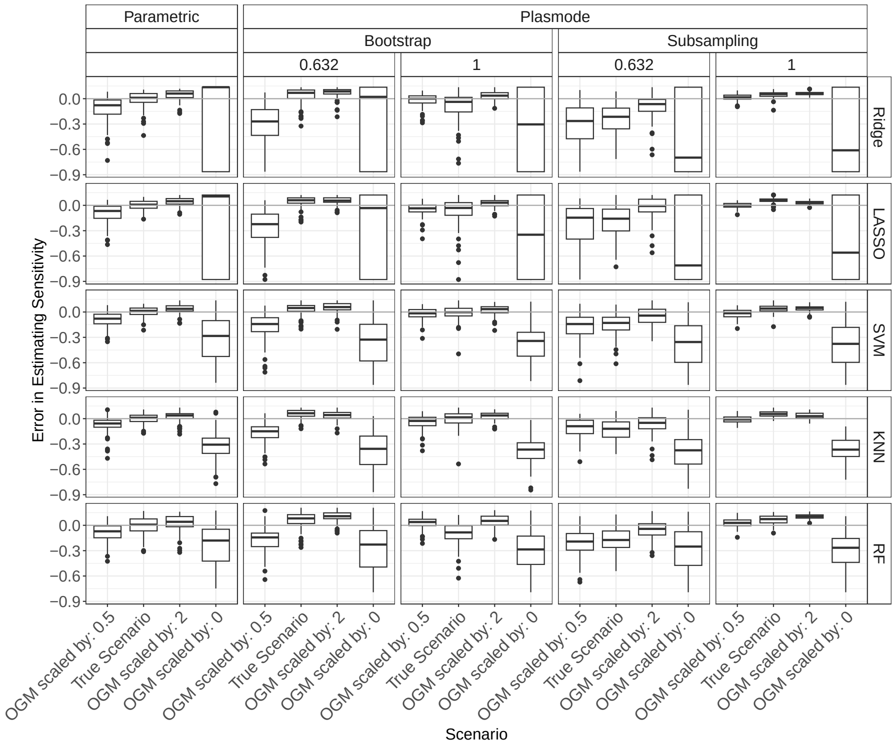

Fig G.10: Errors in the estimation of sensitivity in 100 iterations of a classification method comparison study per classifier for different simulation approaches with misspecifications of the OGM for $p = 10$.

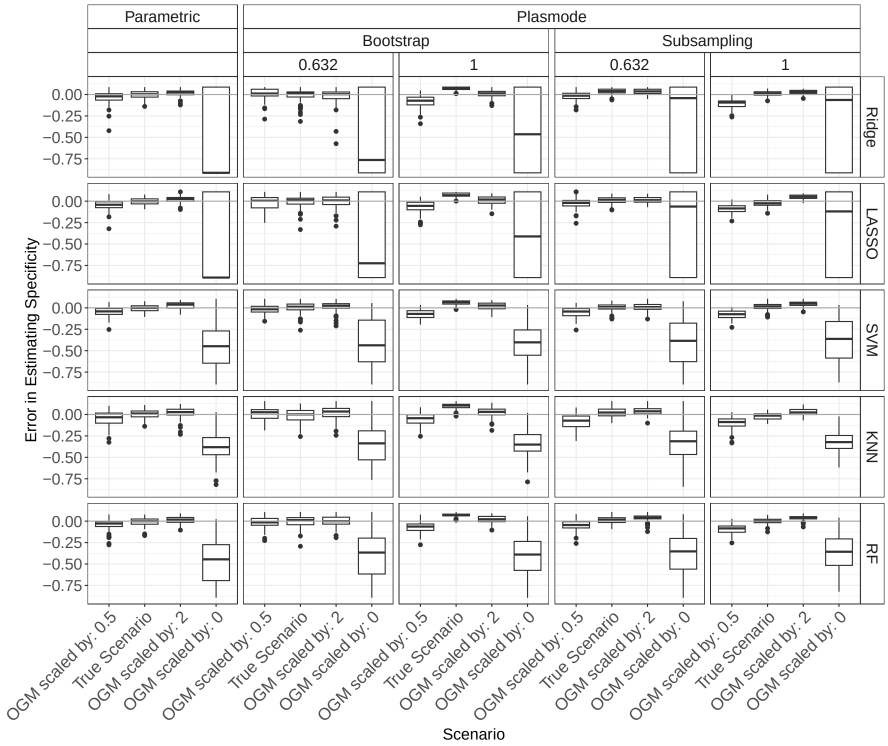

Fig G.11: Errors in the estimation of specificity in 100 iterations of a classification method comparison study per classifier for different simulation approaches with misspecifications of the OGM for $p = 10$.

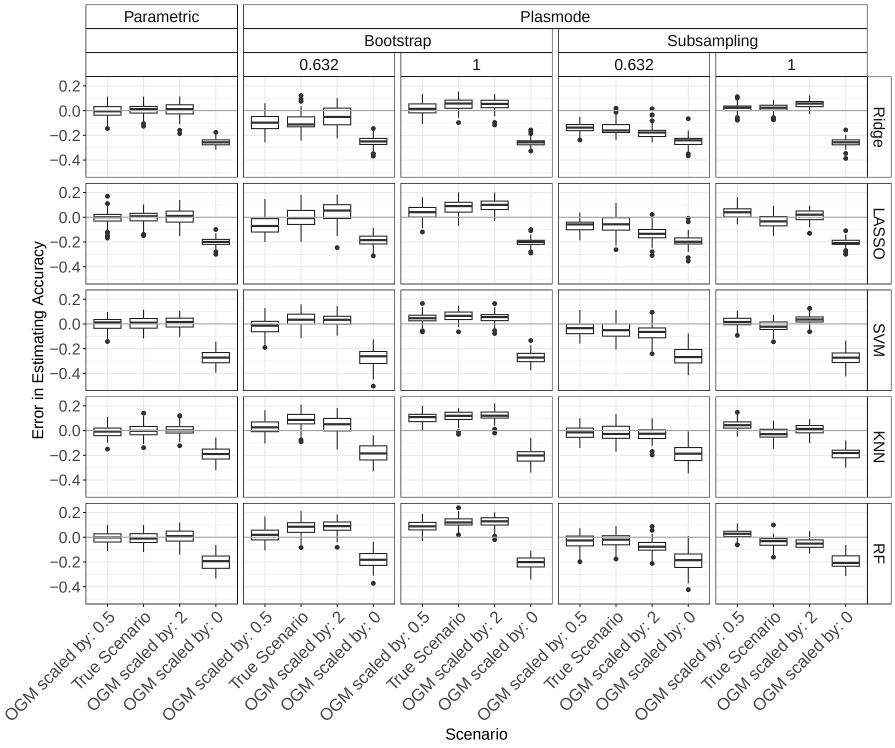

Fig G.12: Errors in the estimation of accuracy in 100 iterations of a classification method comparison study per classifier for different simulation approaches with misspecifications of the OGM for $p = 50$.

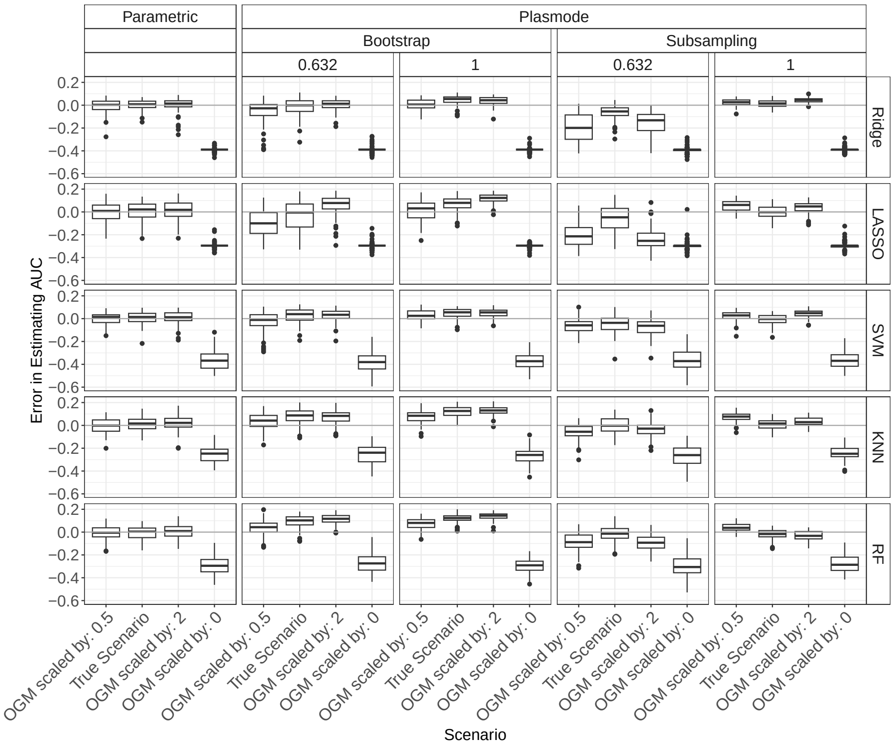

Fig G.13: Errors in the estimation of AUC in 100 iterations of a classification method comparison study per classifier for different simulation approaches with misspecifications of the OGM for $p = 50$.

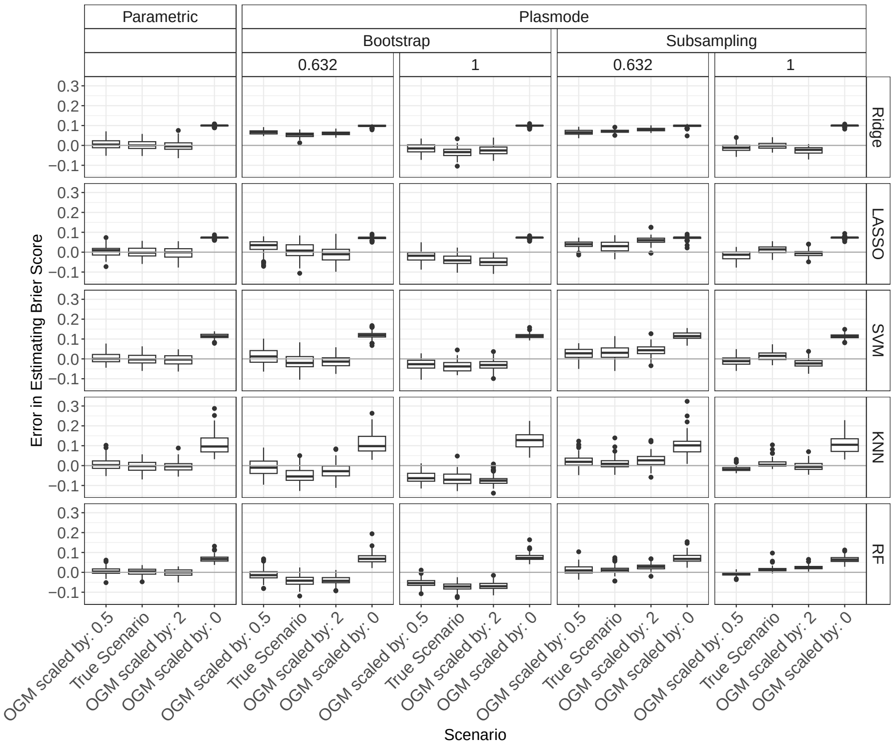

Fig G.14: Errors in the estimation of the Brier score in 100 iterations of a classification method comparison study per classifier for different simulation approaches with misspecifications of the OGM for $p = 50$.

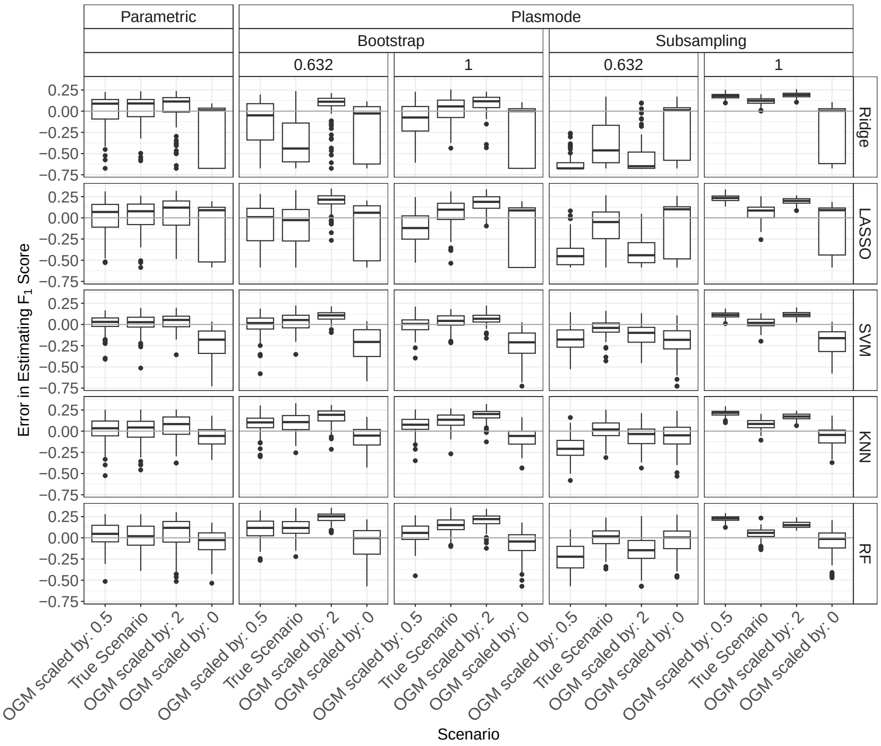

Fig G.15: Errors in the estimation of the F_1-score in 100 iterations of a classification method comparison study per classifier for different simulation approaches with misspecifications of the OGM for $p = 50$.

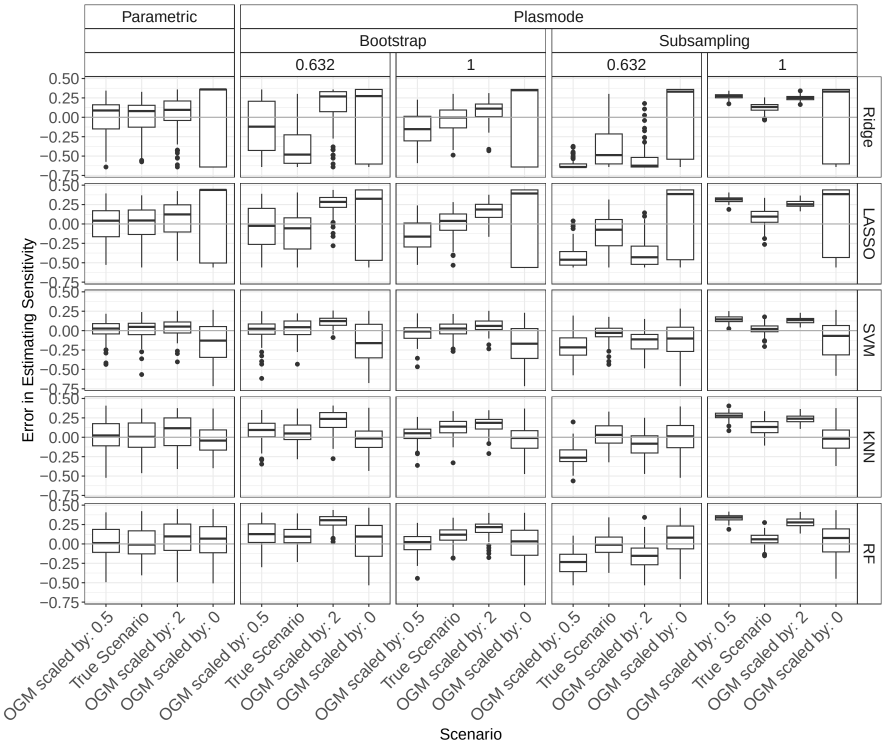

Fig G.16: Errors in the estimation of sensitivity in 100 iterations of a classification method comparison study per classifier for different simulation approaches with misspecifications of the OGM for $p = 50$.

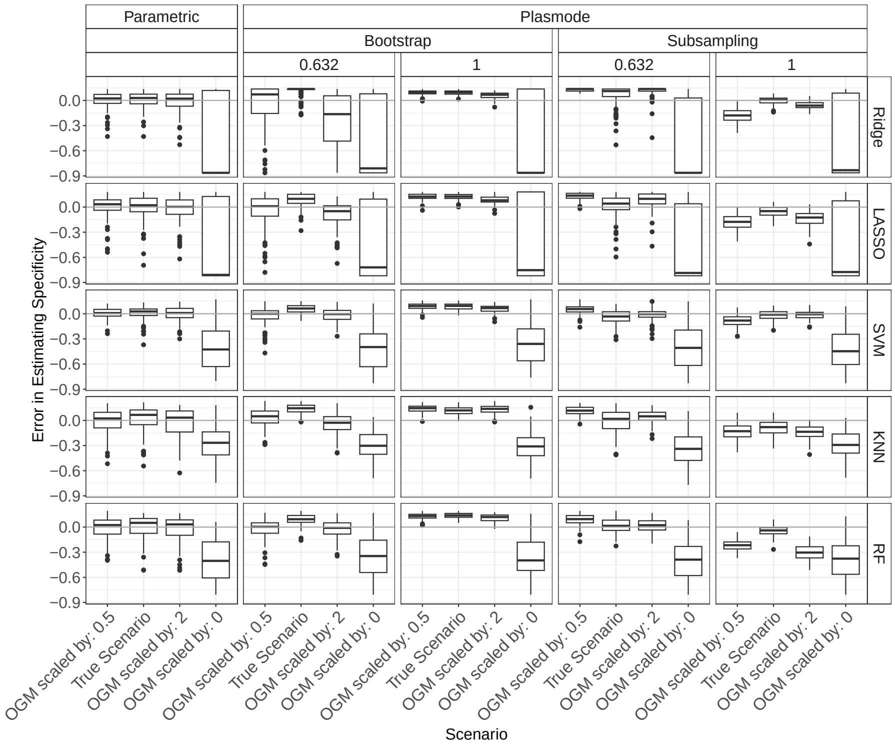

Fig G.17: Errors in the estimation of specificity in 100 iterations of a classification method comparison study per classifier for different simulation approaches with misspecifications of the OGM for $p = 50$.

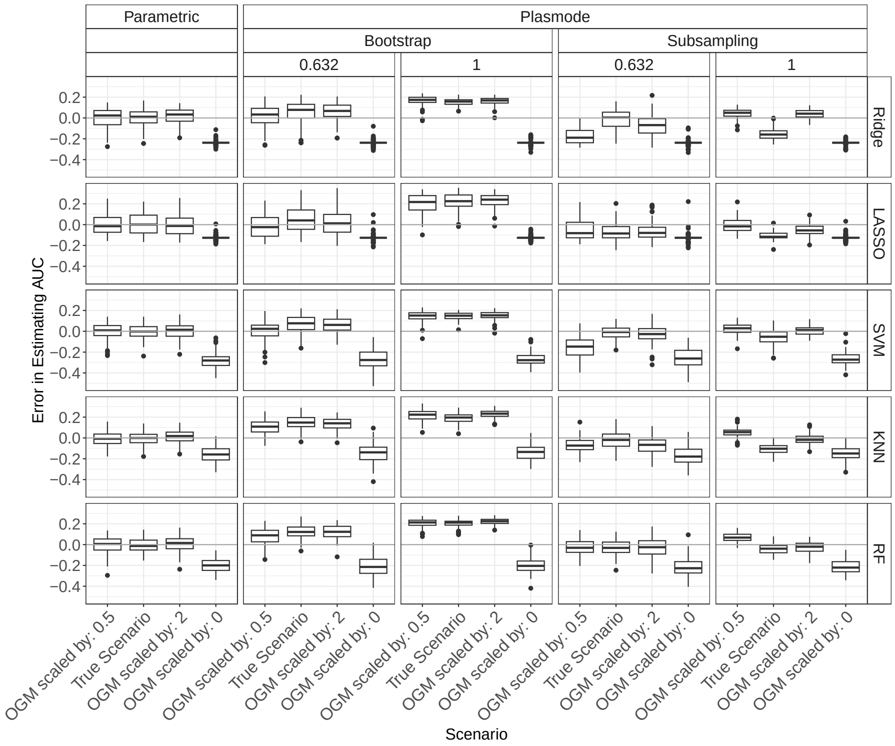

Fig G.18: Errors in the estimation of AUC in 100 iterations of a classification method comparison study per classifier for different simulation approaches with misspecifications of the OGM for $p = 150$.

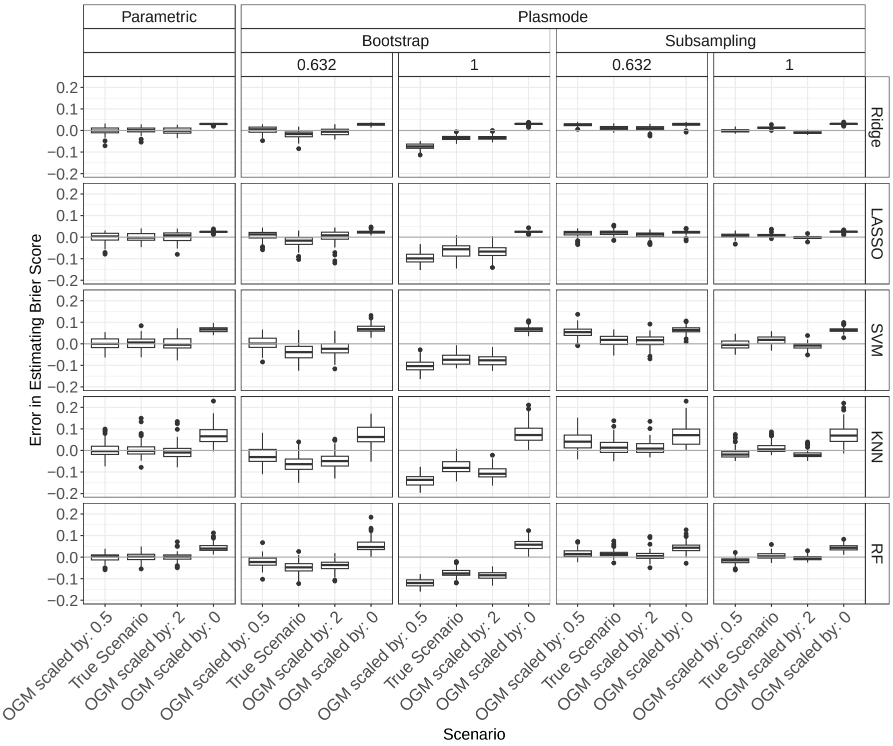

Fig G.19: Errors in the estimation of the Brier score in 100 iterations of a classification method comparison study per classifier for different simulation approaches with misspecifications of the OGM for $p = 150$.

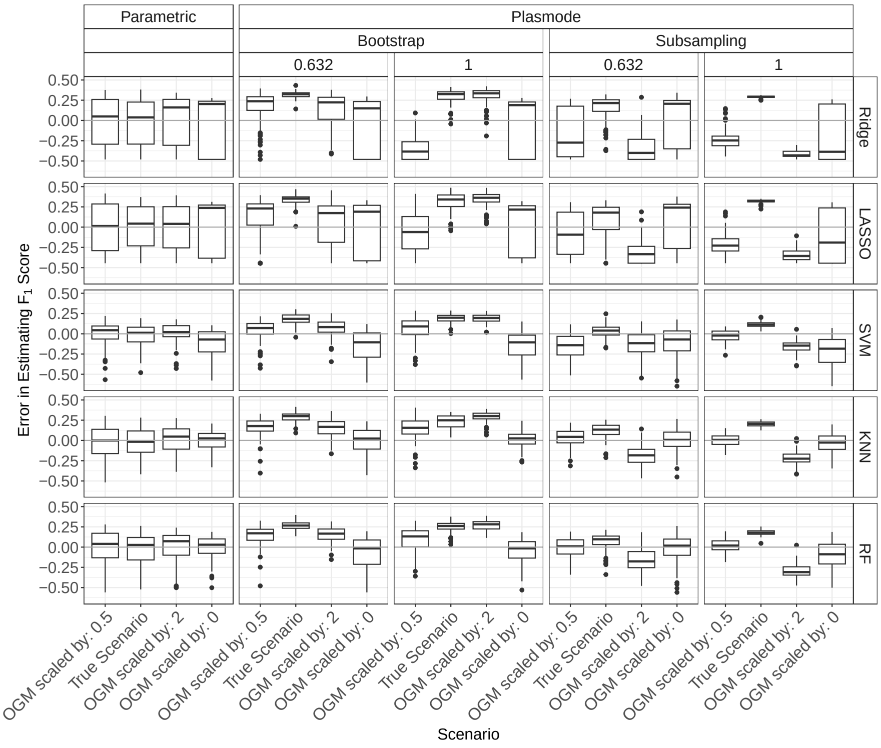

Fig G.20: Errors in the estimation of the F_1-score in 100 iterations of a classification method comparison study per classifier for different simulation approaches with misspecifications of the OGM for $p = 150$.

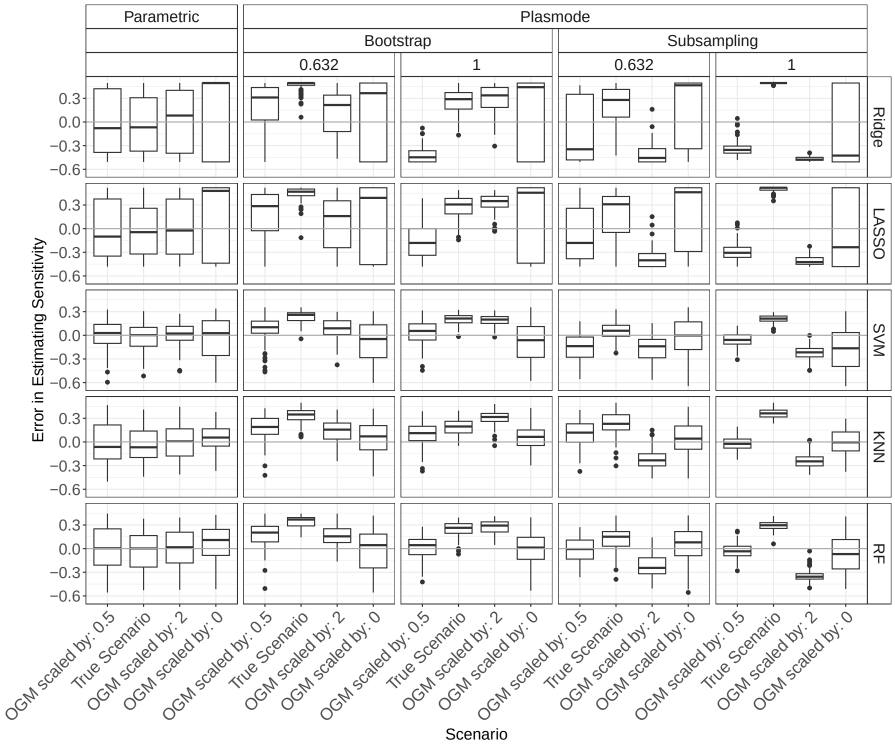

Fig G.21: Errors in the estimation of sensitivity in 100 iterations of a classification method comparison study per classifier for different simulation approaches with misspecifications of the OGM for $p = 150$.

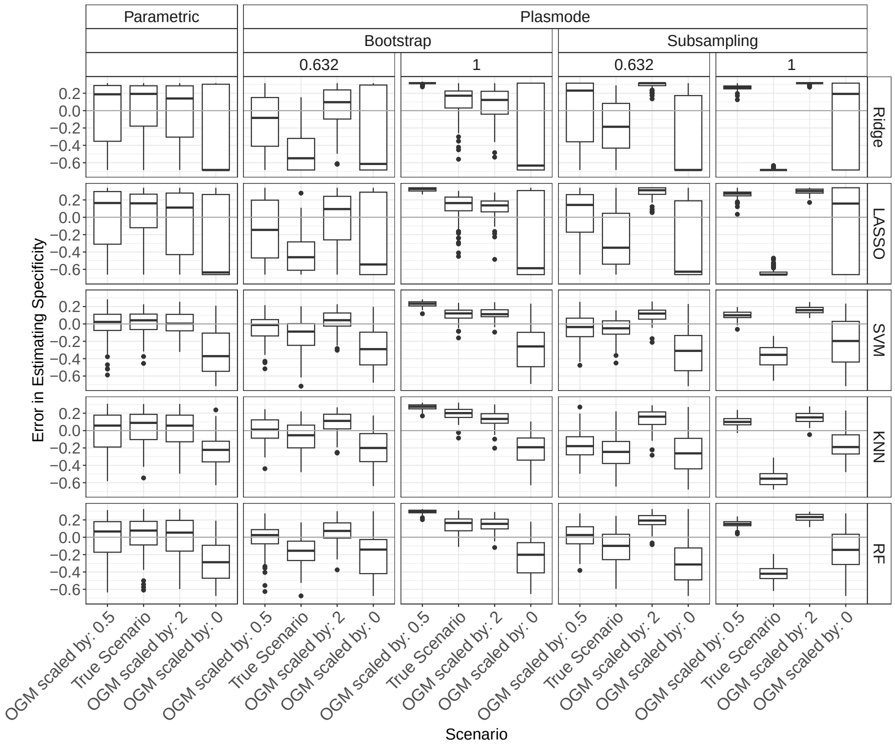

Fig G.22: Errors in the estimation of specificity in 100 iterations of a classification method comparison study per classifier for different simulation approaches with misspecifications of the OGM for $p = 150$.

H Kendall Distance

H.1 $p = 2$

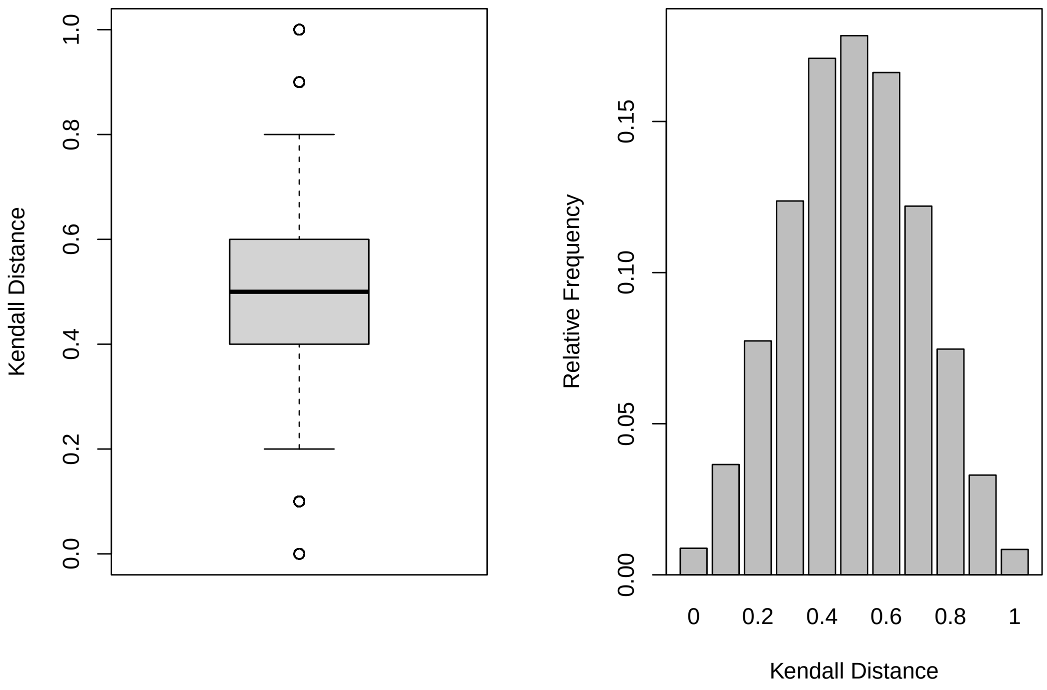

Fig H.1: Distribution of Kendall distance for random rankings. Two random permutations of the numbers $1, \ldots, 5$ are drawn and their Kendall distance is calculated. This process is repeated 10000 times.

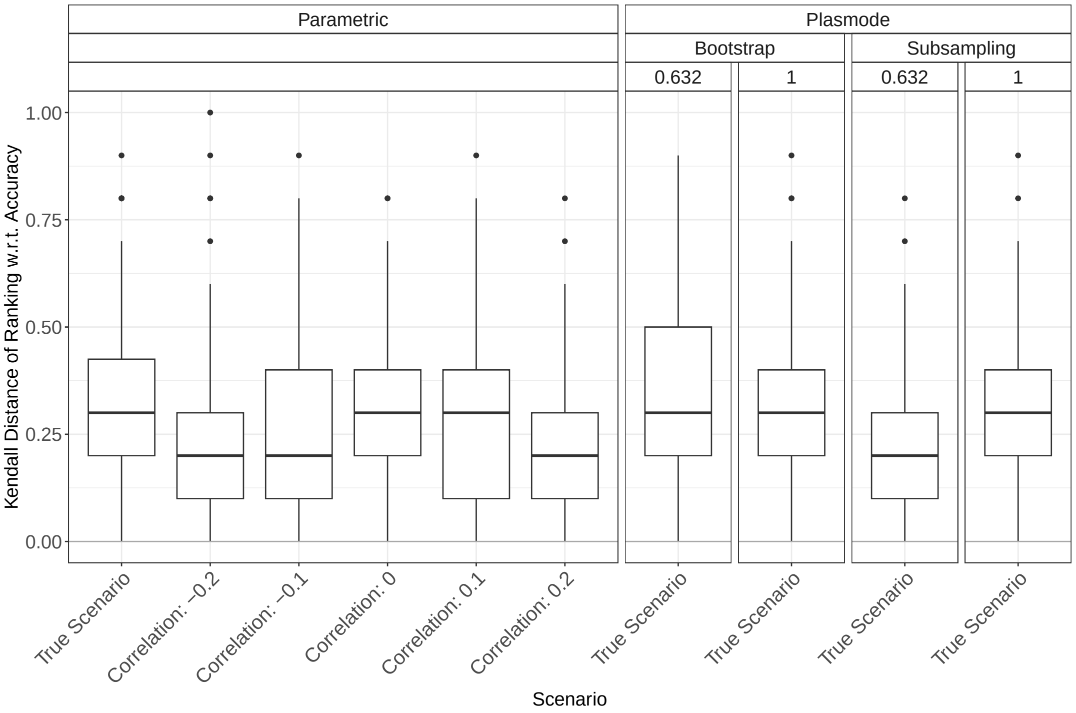

Fig H.2: Kendall distance of the simulated and true method ranking based on accuracy in 100 iterations of a classification method comparison study per classifier for different simulation approaches with misspecifications of the correlation for parametric simulation for $p = 2$.

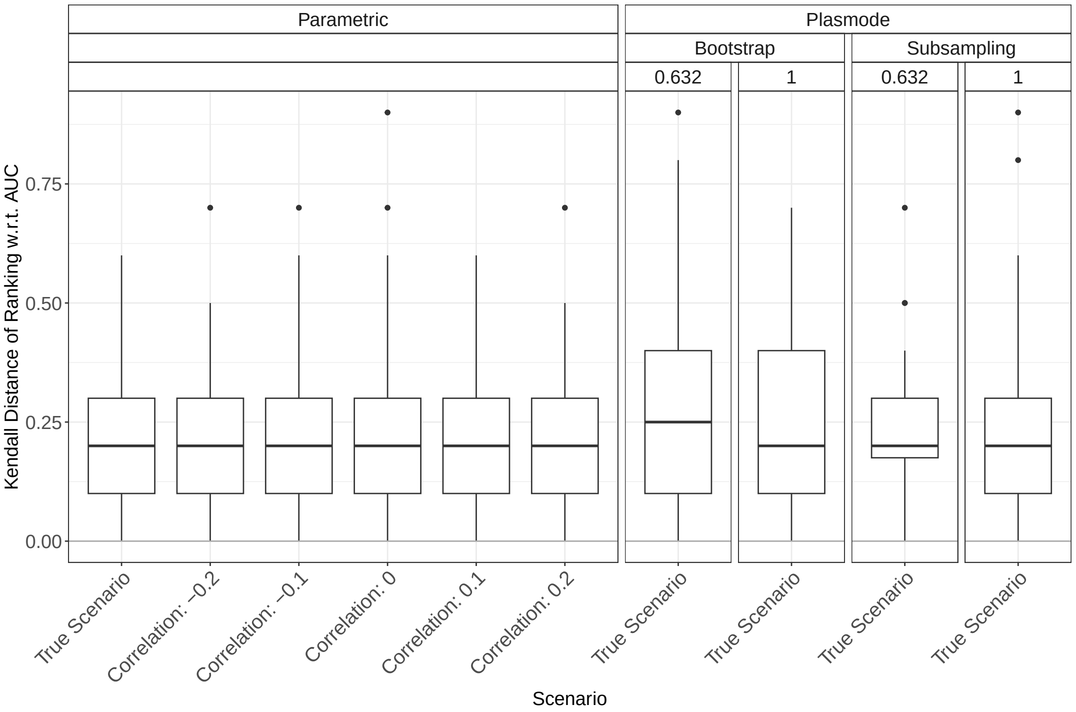

Fig H.3: Kendall distance of the simulated and true method ranking based on AUC in 100 iterations of a classification method comparison study per classifier for different simulation approaches with misspecifications of the correlation for parametric simulation for $p = 2$.

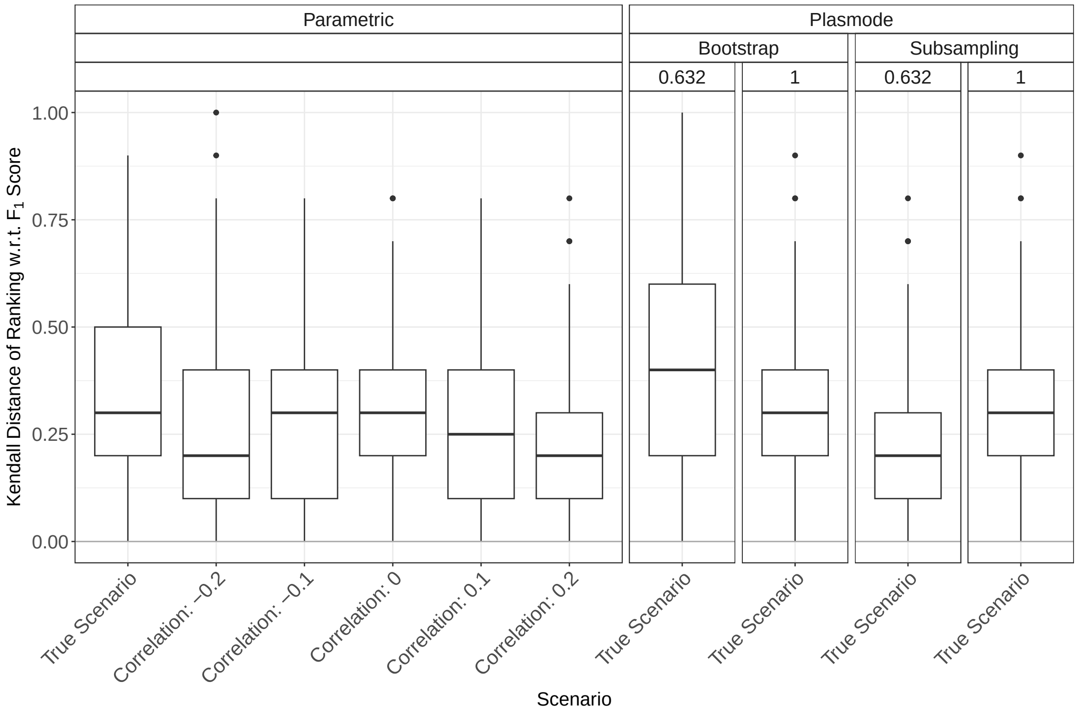

Fig H.4: Kendall distance of the simulated and true method ranking based on F_1-score in 100 iterations of a classification method comparison study per classifier for different simulation approaches with misspecifications of the correlation for parametric simulation for $p = 2$.

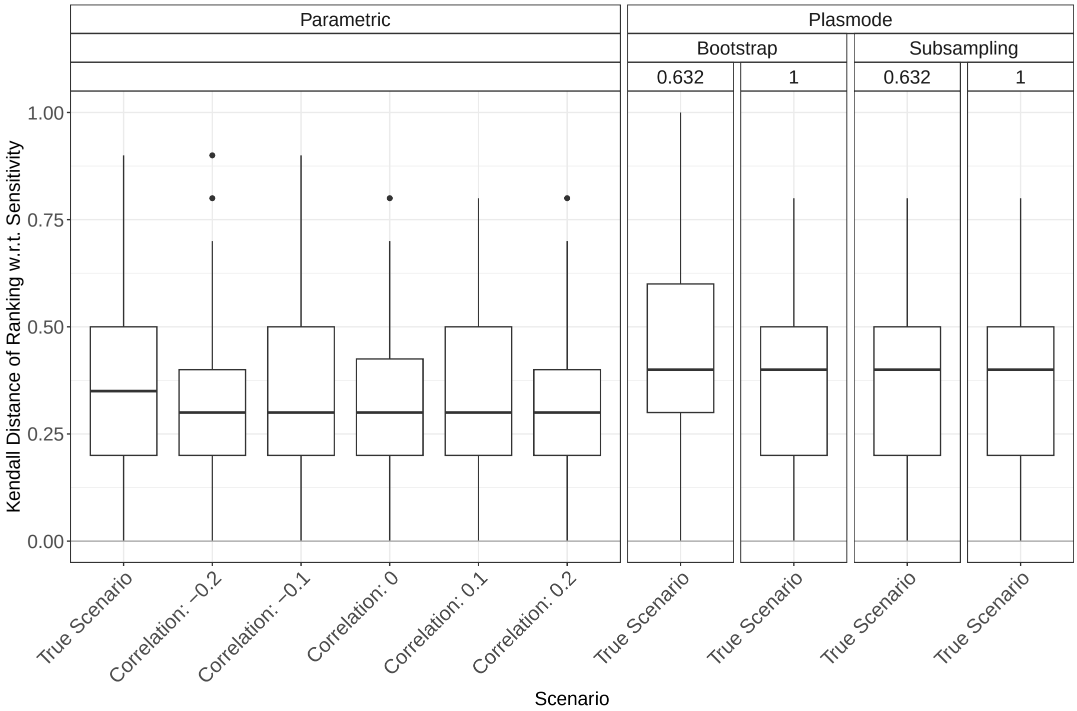

Fig H.5: Kendall distance of the simulated and true method ranking based on sensitivity in 100 iterations of a classification method comparison study per classifier for different simulation approaches with misspecifications of the correlation for parametric simulation for $p = 2$.

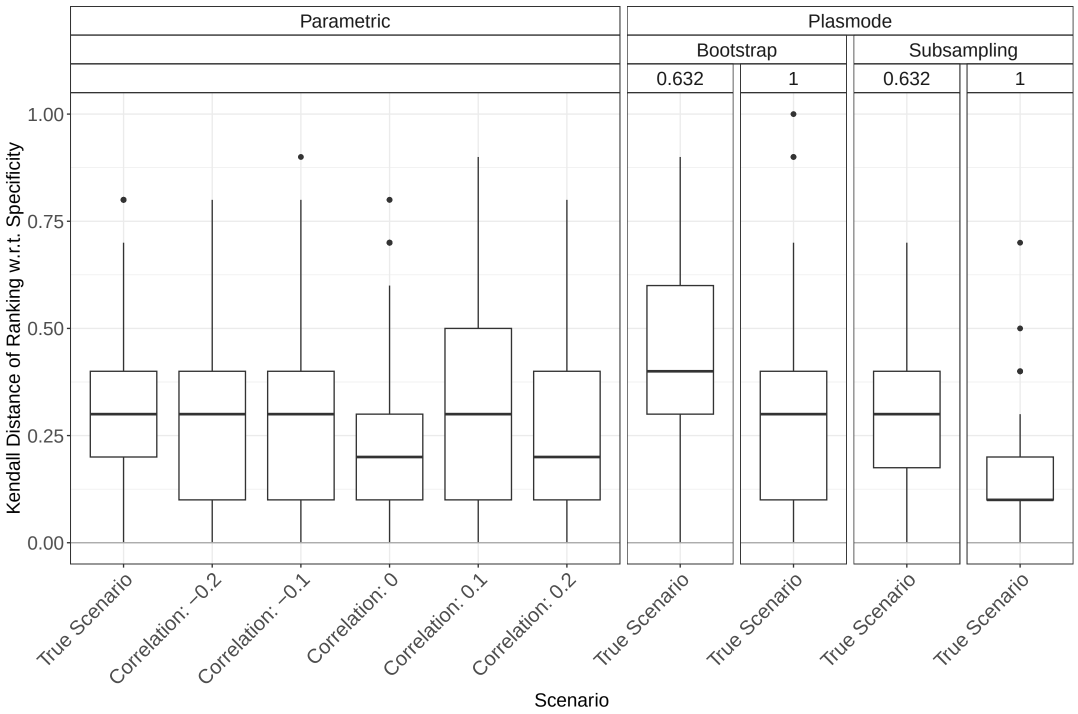

Fig H.6: Kendall distance of the simulated and true method ranking based on specificity in 100 iterations of a classification method comparison study per classifier for different simulation approaches with misspecifications of the correlation for parametric simulation for $p = 2$.

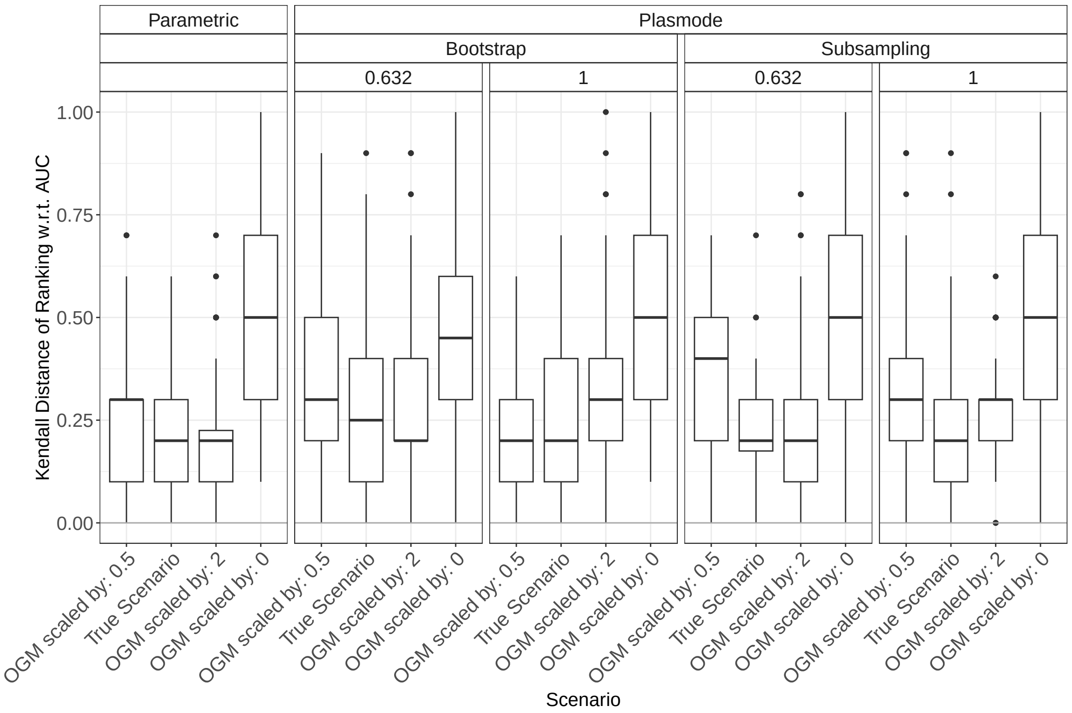

Fig H.7: Kendall distance of the simulated and true method ranking based on AUC in 100 iterations of a classification method comparison study per classifier for different simulation approaches with misspecifications of the OGM for $p = 2$.

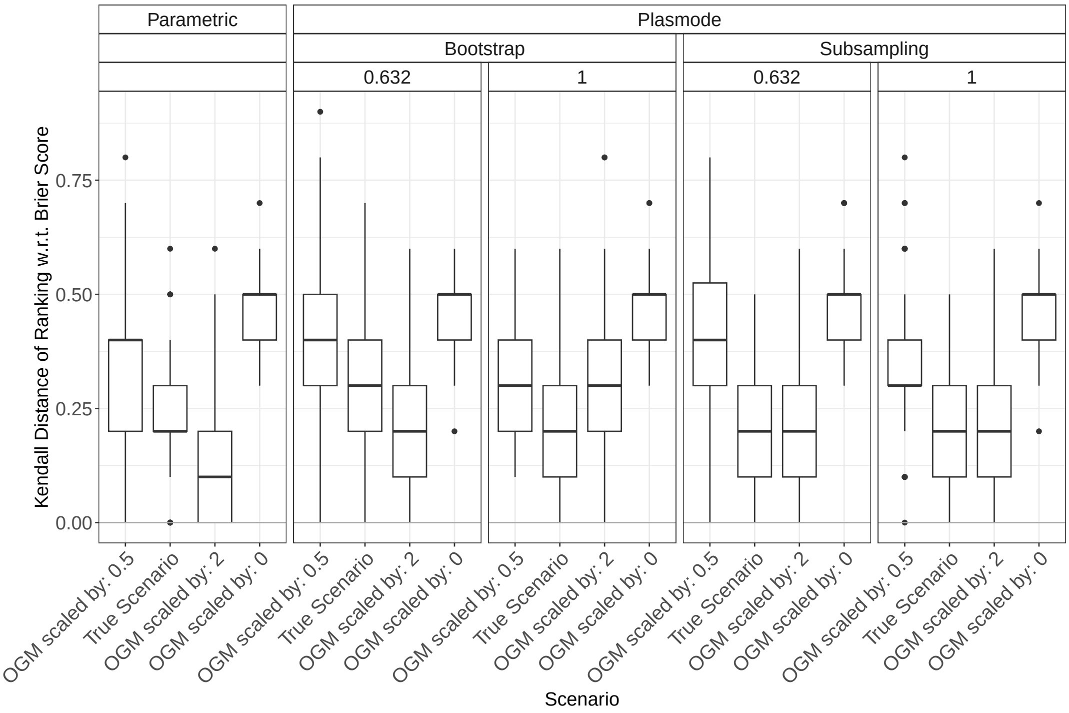

Fig H.8: Kendall distance of the simulated and true method ranking based on the Brier score in 100 iterations of a classification method comparison study per classifier for different simulation approaches with misspecifications of the OGM for $p = 2$.

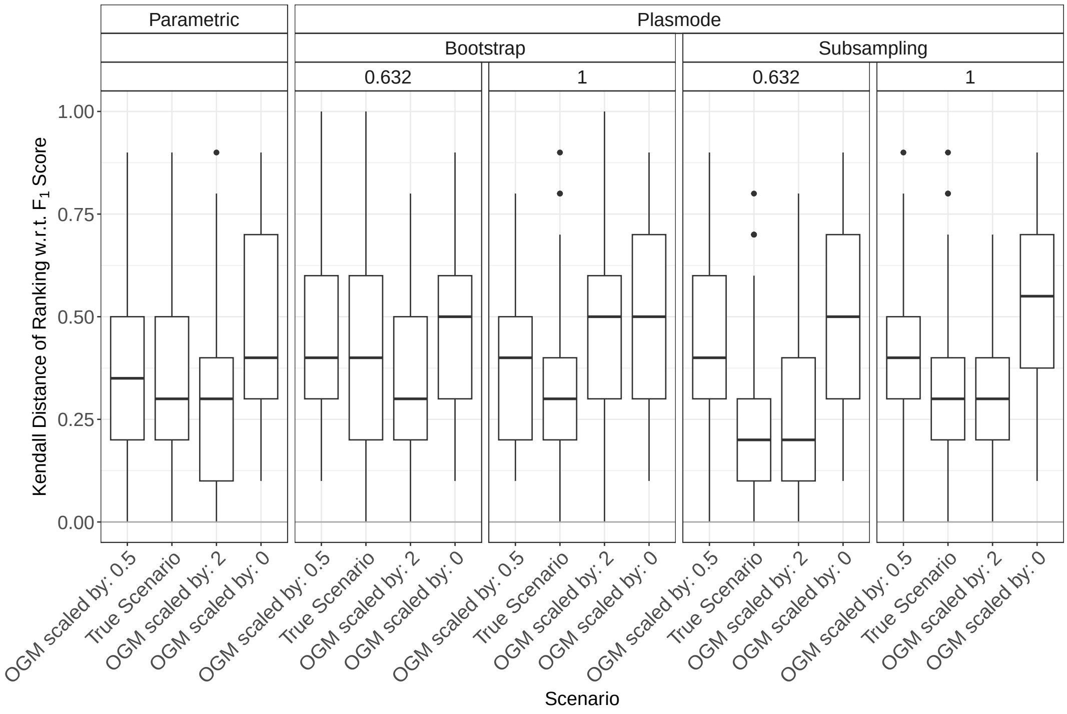

Fig H.9: Kendall distance of the simulated and true method ranking based on F_1-score in 100 iterations of a classification method comparison study per classifier for different simulation approaches with misspecifications of the OGM for parametric simulation for $p = 2$.

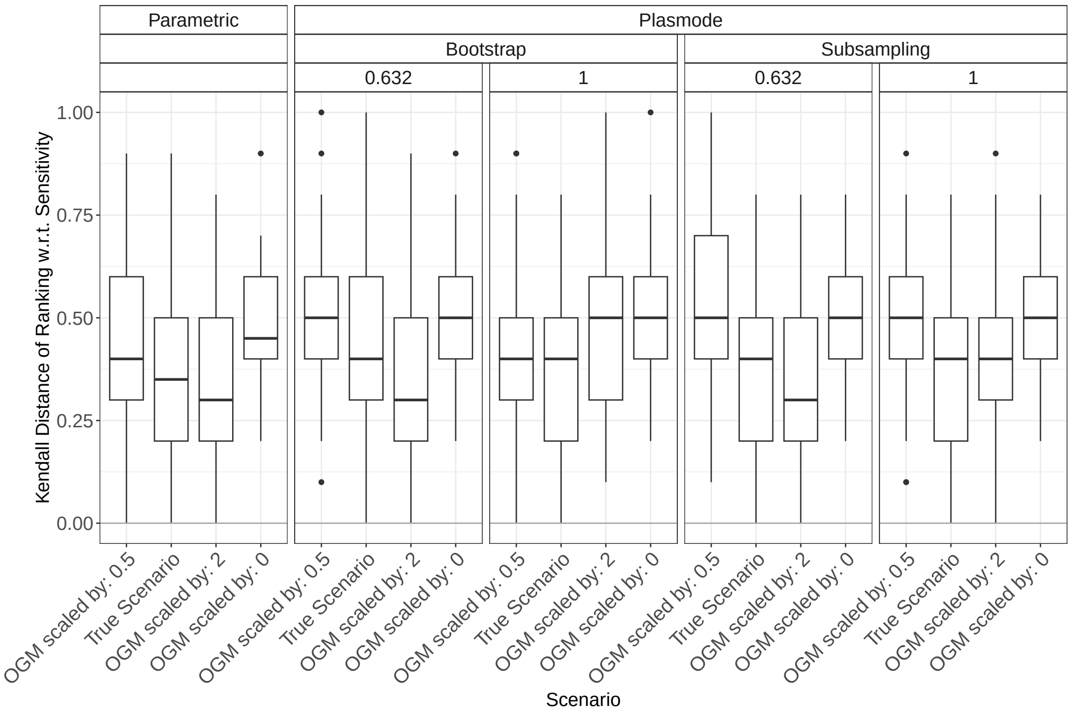

Fig H.10: Kendall distance of the simulated and true method ranking based on sensitivity in 100 iterations of a classification method comparison study per classifier for different simulation approaches with misspecifications of the OGM for $p = 2$.

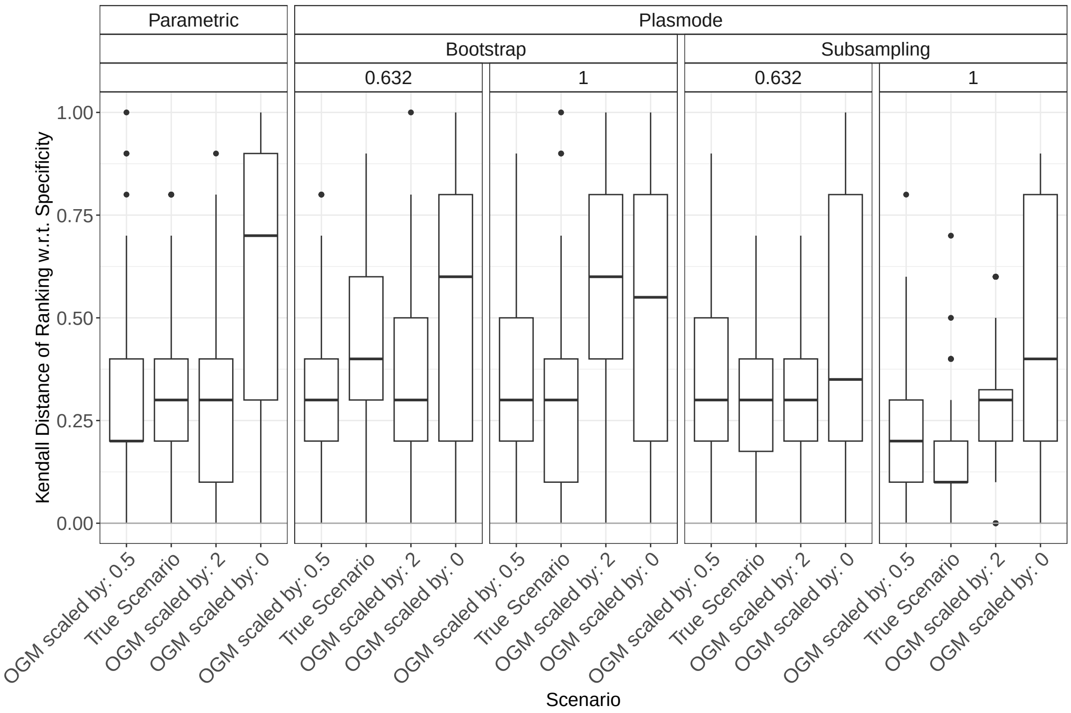

Fig H.11: Kendall distance of the simulated and true method ranking based on specificity in 100 iterations of a classification method comparison study per classifier for different simulation approaches with misspecifications of the OGM for $p = 2$.

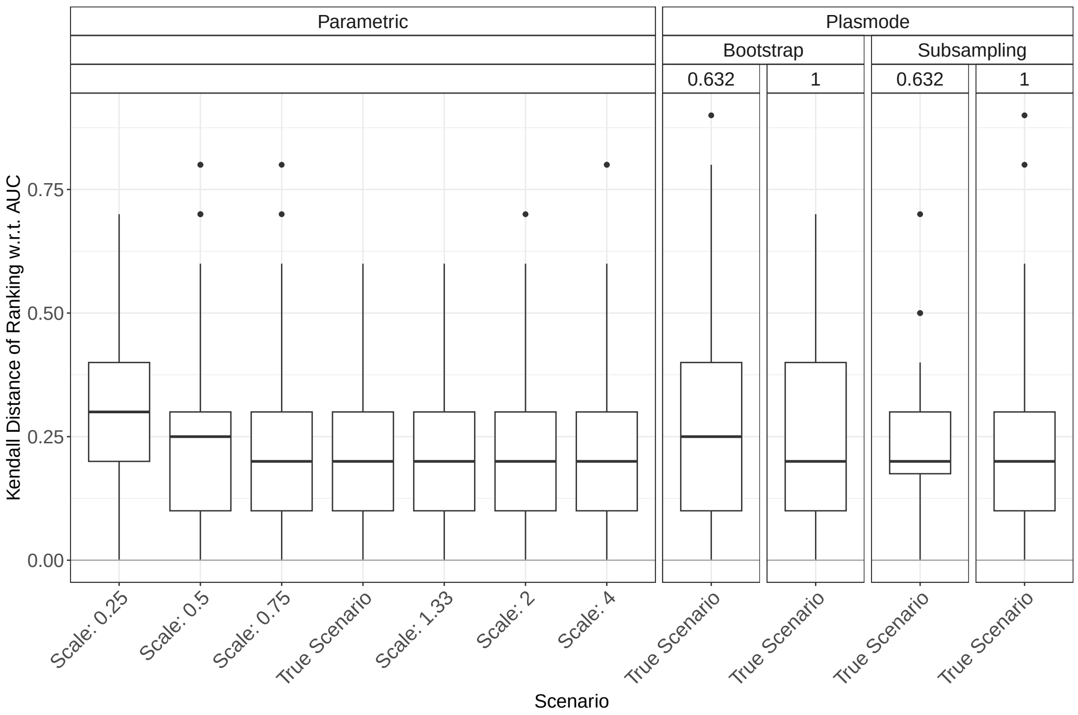

Fig H.12: Kendall distance of the simulated and true method ranking based on AUC in 100 iterations of a classification method comparison study per classifier for different simulation approaches with misspecifications of the scale for parametric simulation for $p = 2$.

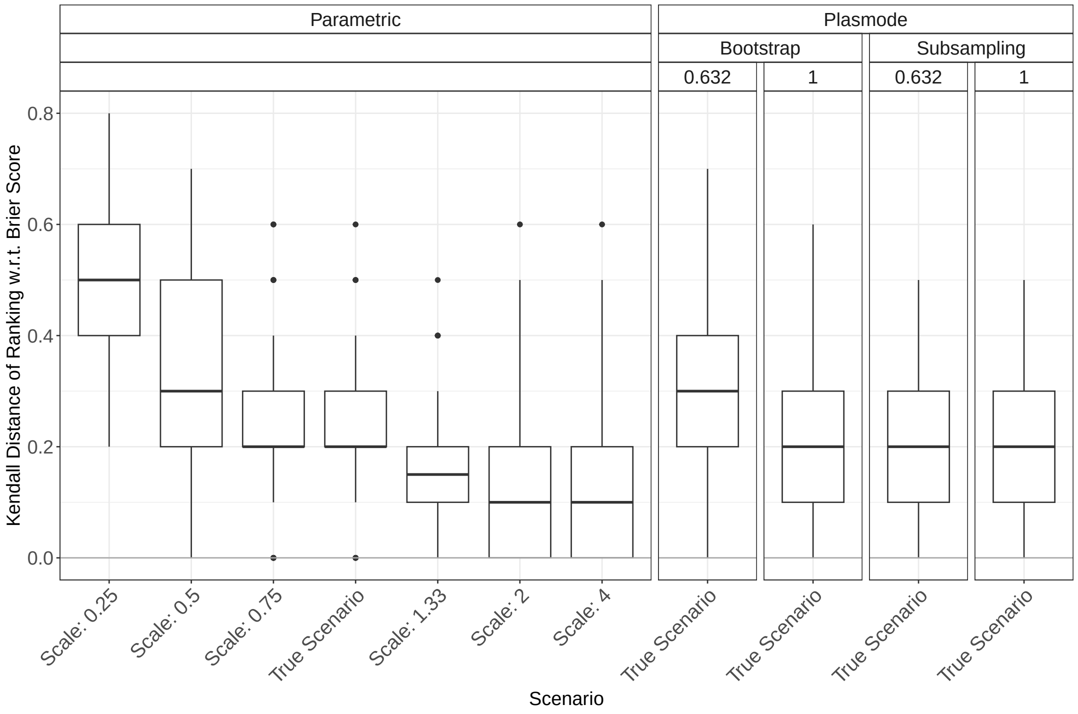

Fig H.13: Kendall distance of the simulated and true method ranking based on the Brier score in 100 iterations of a classification method comparison study per classifier for different simulation approaches with misspecifications of the scale for parametric simulation for $p = 2$.

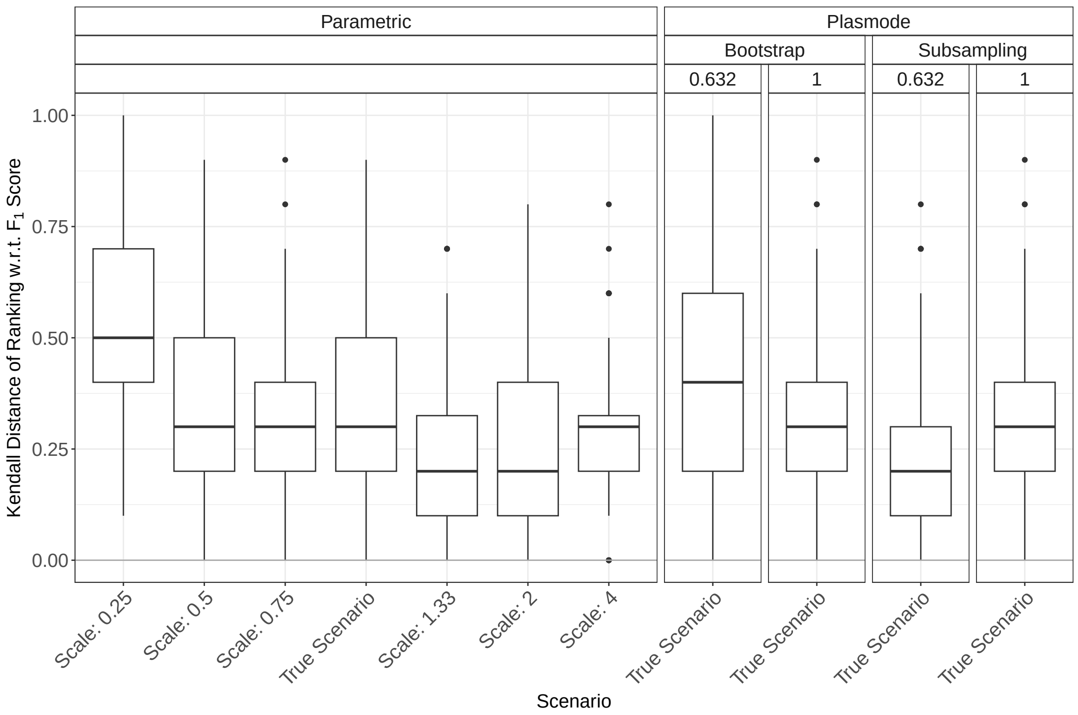

Fig H.14: Kendall distance of the simulated and true method ranking based on F_1-score in 100 iterations of a classification method comparison study per classifier for different simulation approaches with misspecifications of the scale for parametric simulation for $p = 2$.

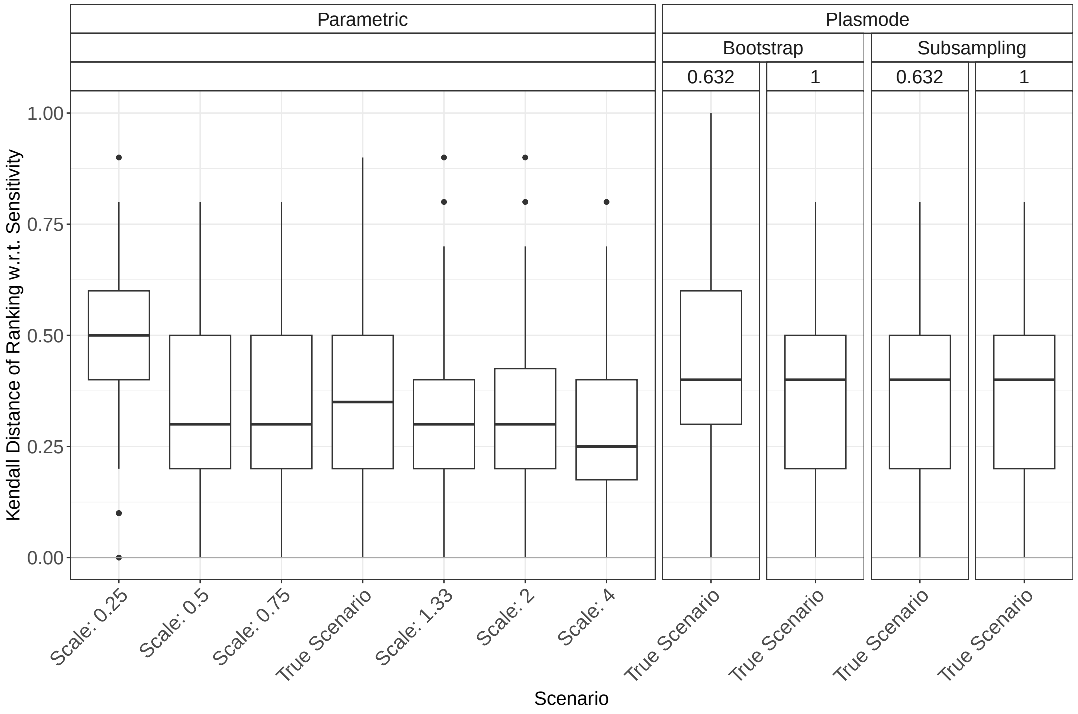

Fig H.15: Kendall distance of the simulated and true method ranking based on sensitivity in 100 iterations of a classification method comparison study per classifier for different simulation approaches with misspecifications of the scale for parametric simulation for $p = 2$.

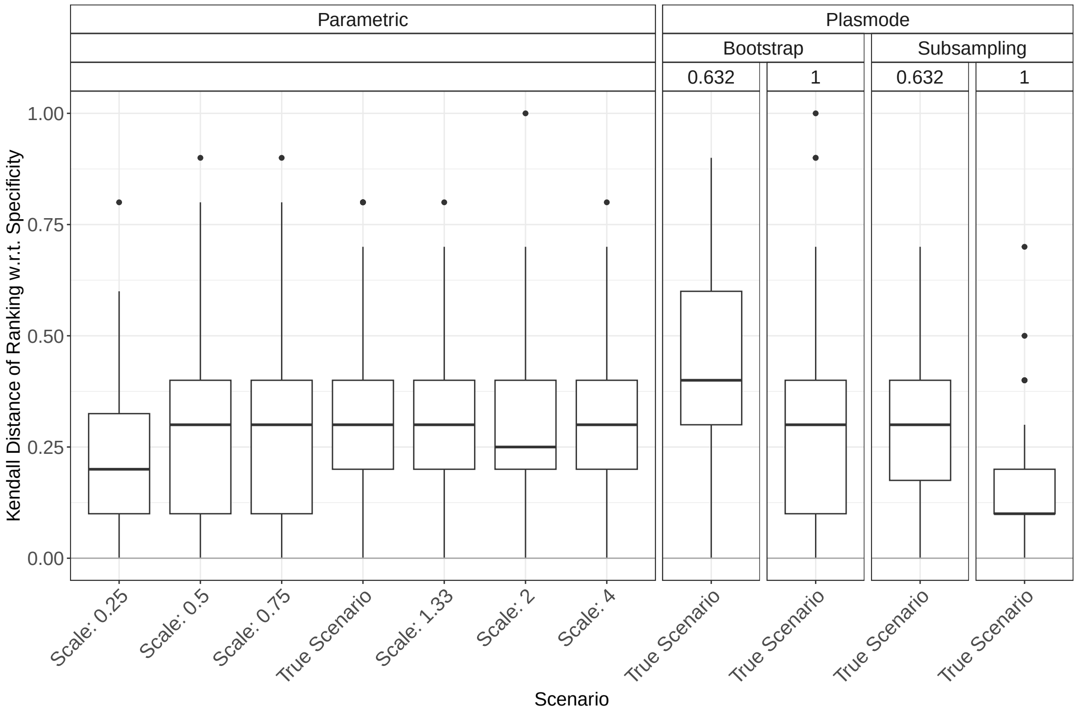

Fig H.16: Kendall distance of the simulated and true method ranking based on specificity in 100 iterations of a classification method comparison study per classifier for different simulation approaches with misspecifications of the scale for parametric simulation for $p = 2$.

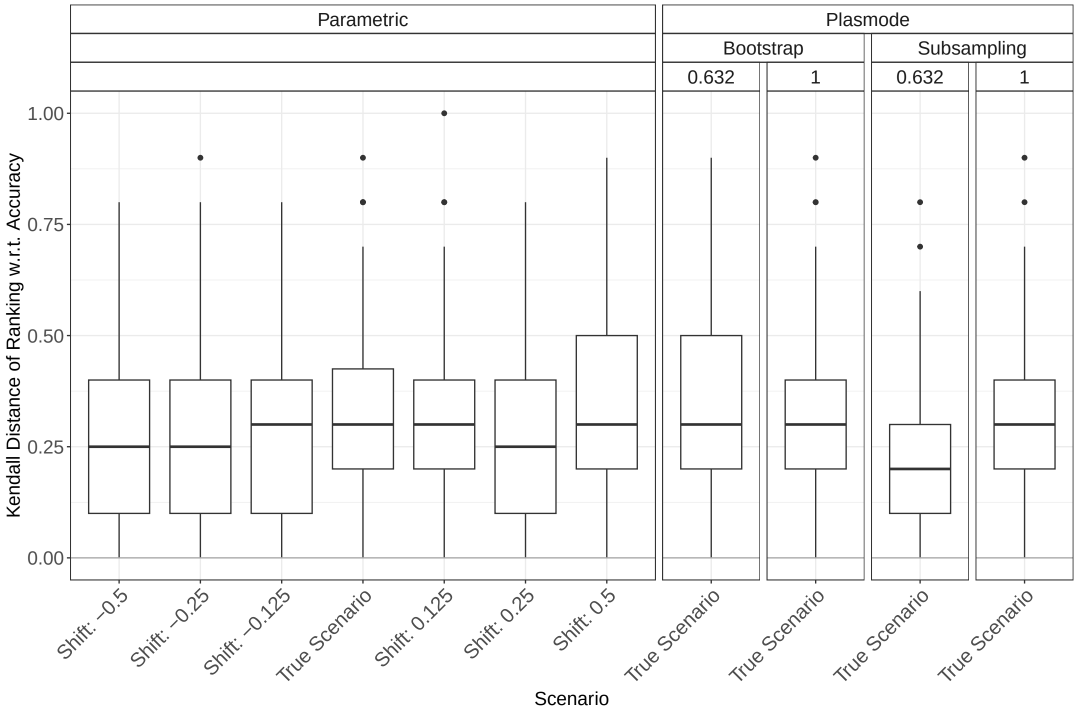

Fig H.17: Kendall distance of the simulated and true method ranking based on accuracy in 100 iterations of a classification method comparison study per classifier for different simulation approaches with misspecifications of the shift for parametric simulation for $p = 2$.

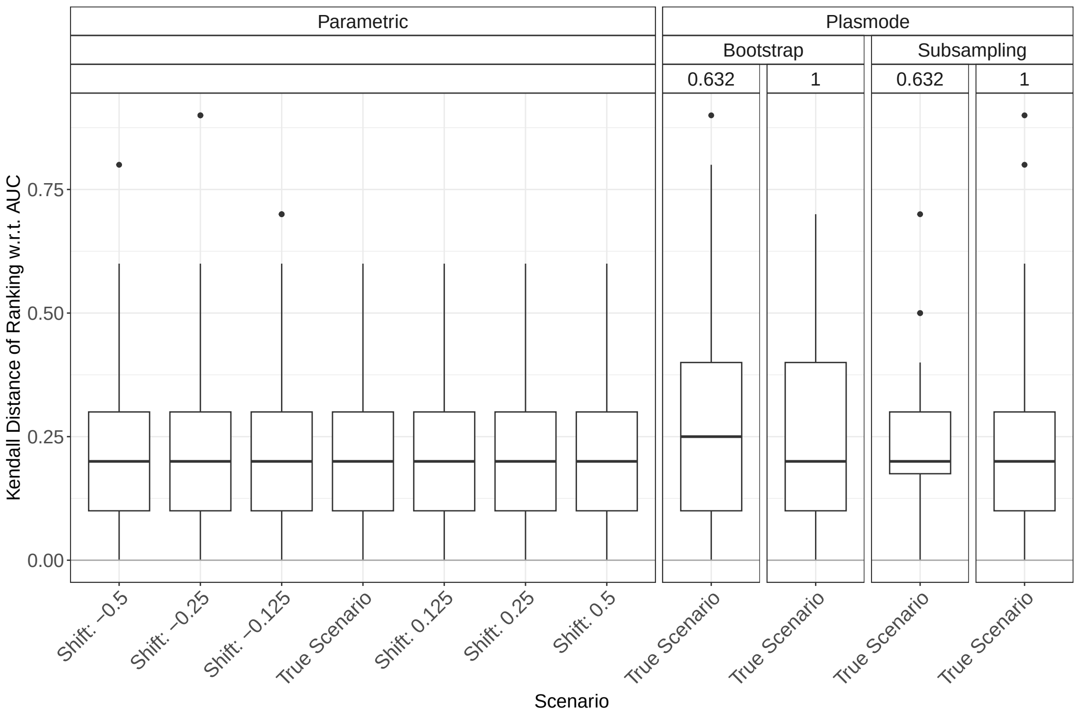

Fig H.18: Kendall distance of the simulated and true method ranking based on AUC in 100 iterations of a classification method comparison study per classifier for different simulation approaches with misspecifications of the shift for parametric simulation for $p = 2$.

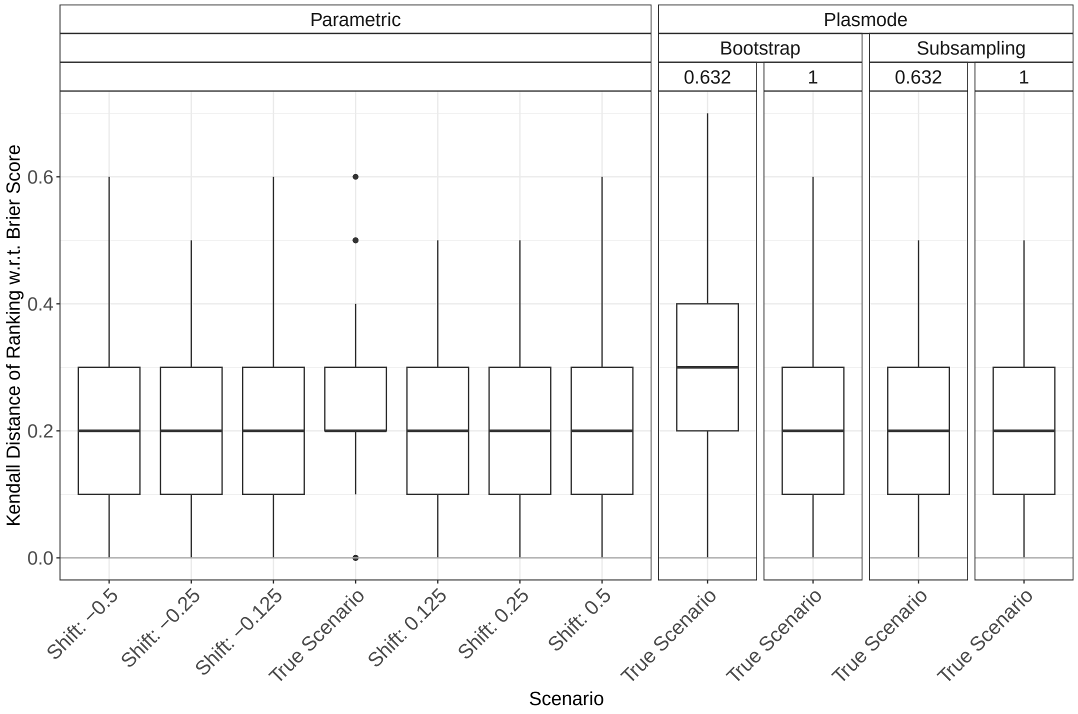

Fig H.19: Kendall distance of the simulated and true method ranking based on the Brier score in 100 iterations of a classification method comparison study per classifier for different simulation approaches with misspecifications of the shift for parametric simulation for $p = 2$.

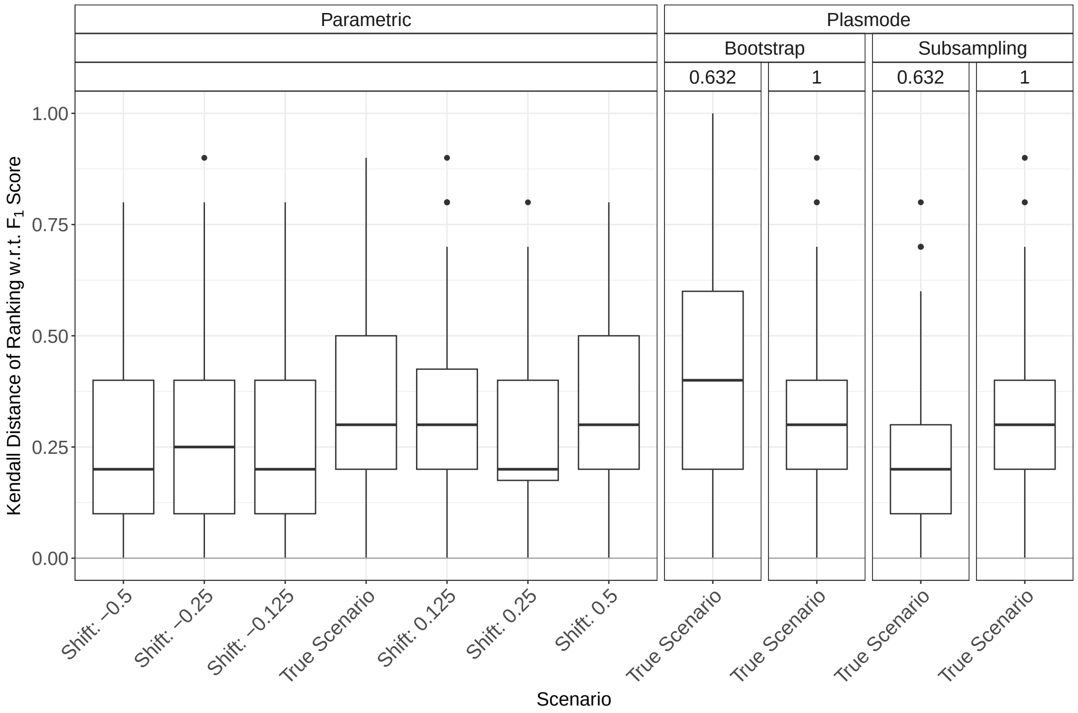

Fig H.20: Kendall distance of the simulated and true method ranking based on F_1-score in 100 iterations of a classification method comparison study per classifier for different simulation approaches with misspecifications of the shift for parametric simulation for $p = 2$.

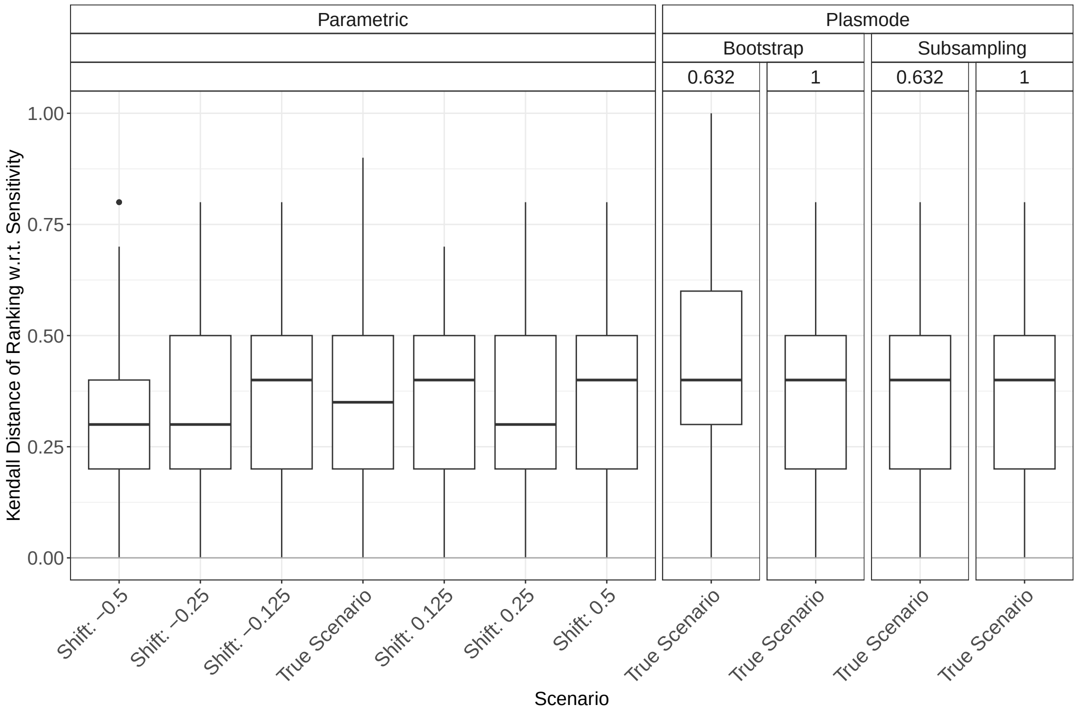

Fig H.21: Kendall distance of the simulated and true method ranking based on sensitivity in 100 iterations of a classification method comparison study per classifier for different simulation approaches with misspecifications of the shift for parametric simulation for $p = 2$.

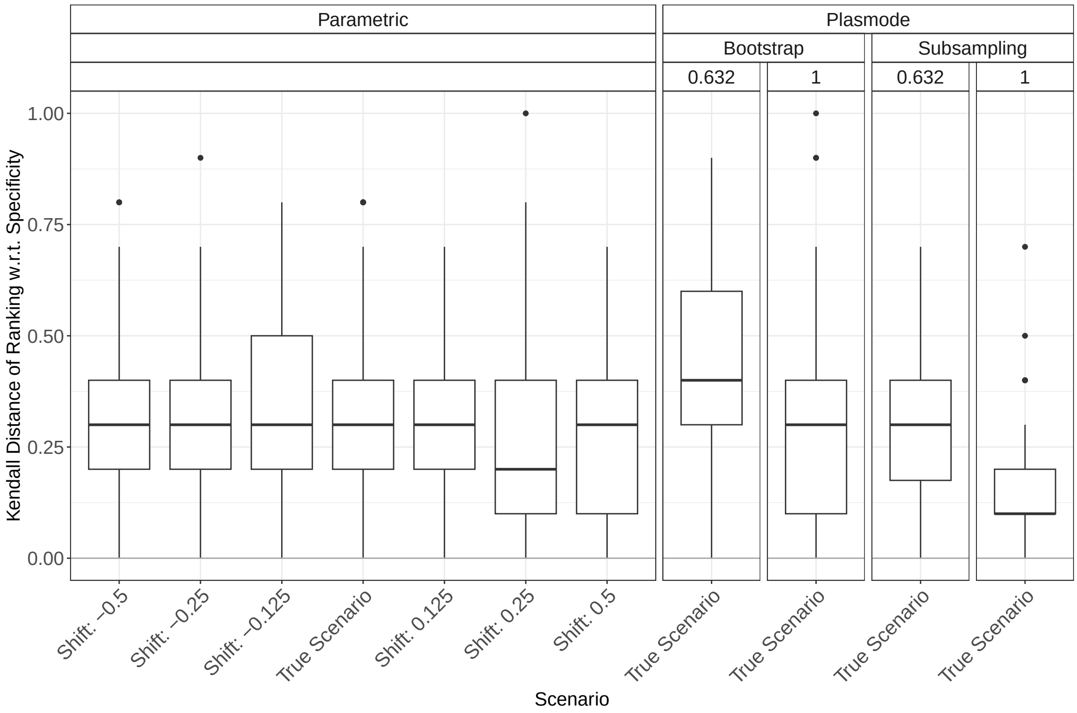

Fig H.22: Kendall distance of the simulated and true method ranking based on specificity in 100 iterations of a classification method comparison study per classifier for different simulation approaches with misspecifications of the shift for parametric simulation for $p = 2$.

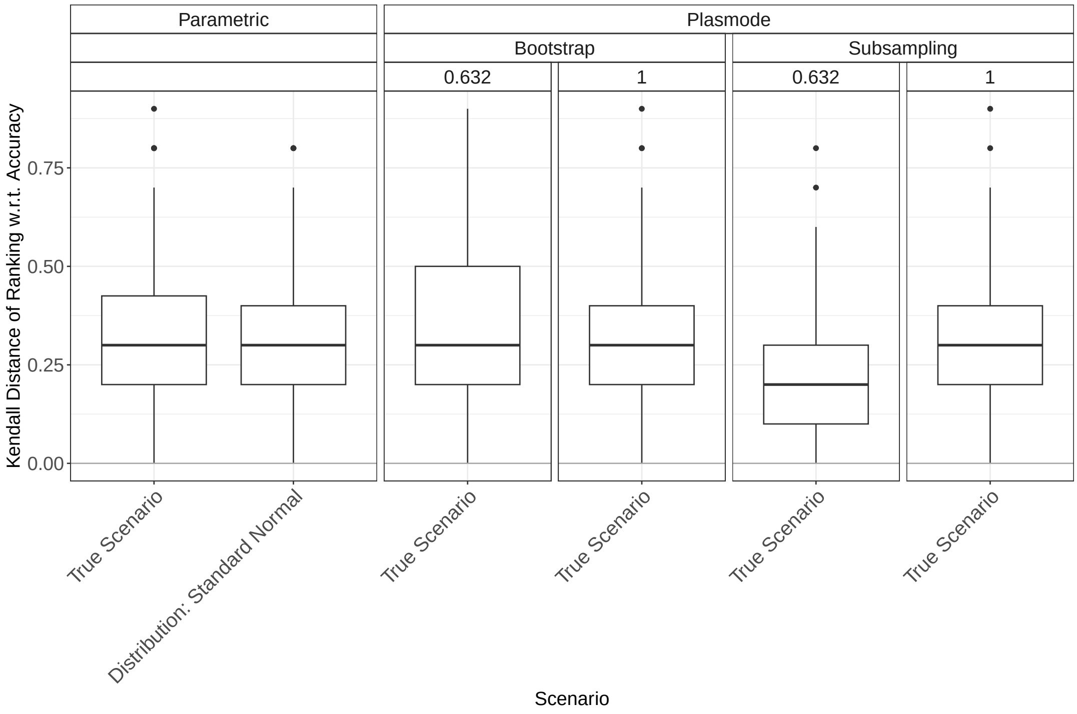

Fig H.23: Kendall distance of the simulated and true method ranking based on accuracy in 100 iterations of a classification method comparison study per classifier for different simulation approaches with misspecification of the distribution as standard normal for parametric simulation for $p = 2$.

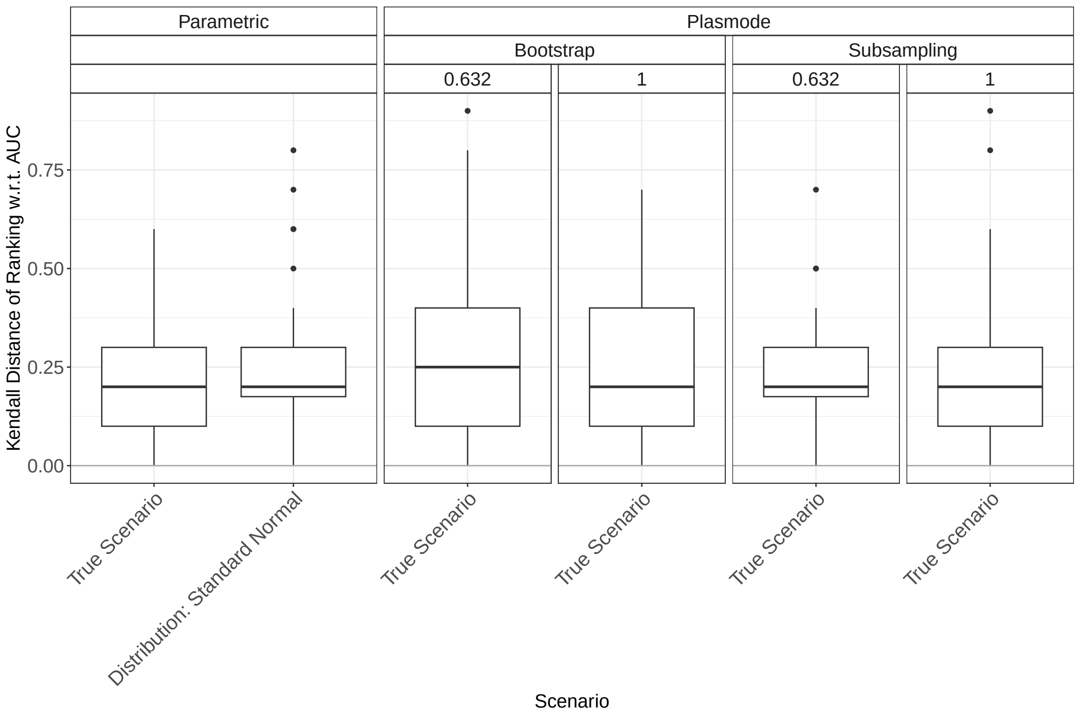

Fig H.24: Kendall distance of the simulated and true method ranking based on AUC in 100 iterations of a classification method comparison study per classifier for different simulation approaches with misspecification of the distribution as standard normal for parametric simulation for $p = 2$.

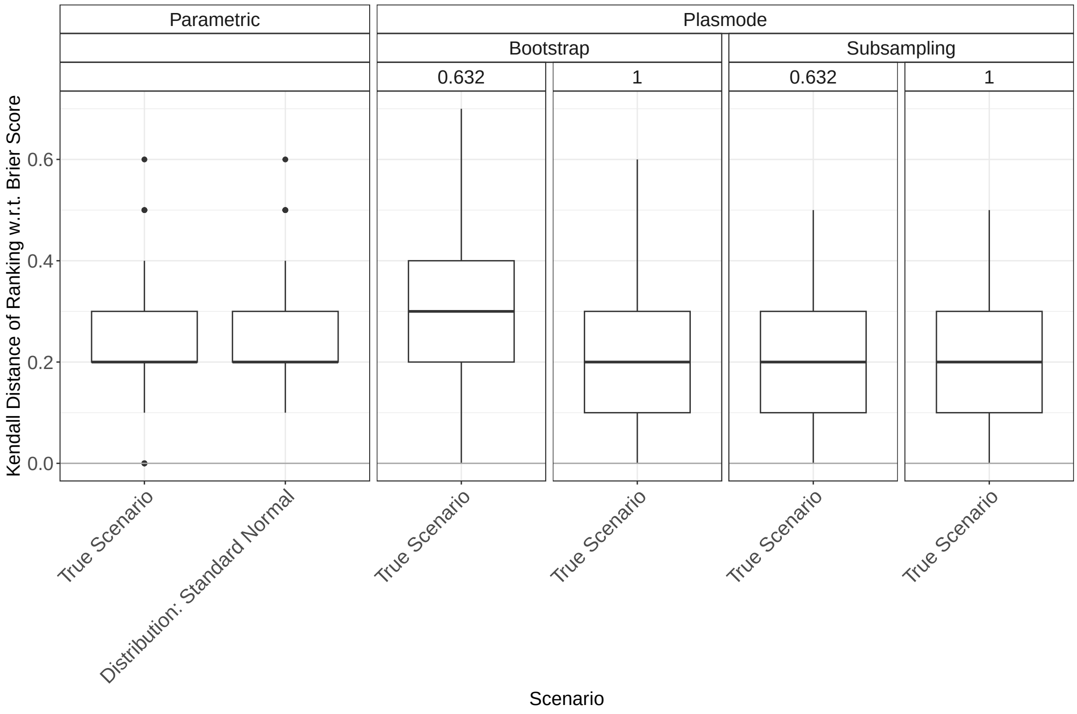

Fig H.25: Kendall distance of the simulated and true method ranking based on the Brier score in 100 iterations of a classification method comparison study per classifier for different simulation approaches with misspecification of the distribution as standard normal for parametric simulation for $p = 2$.

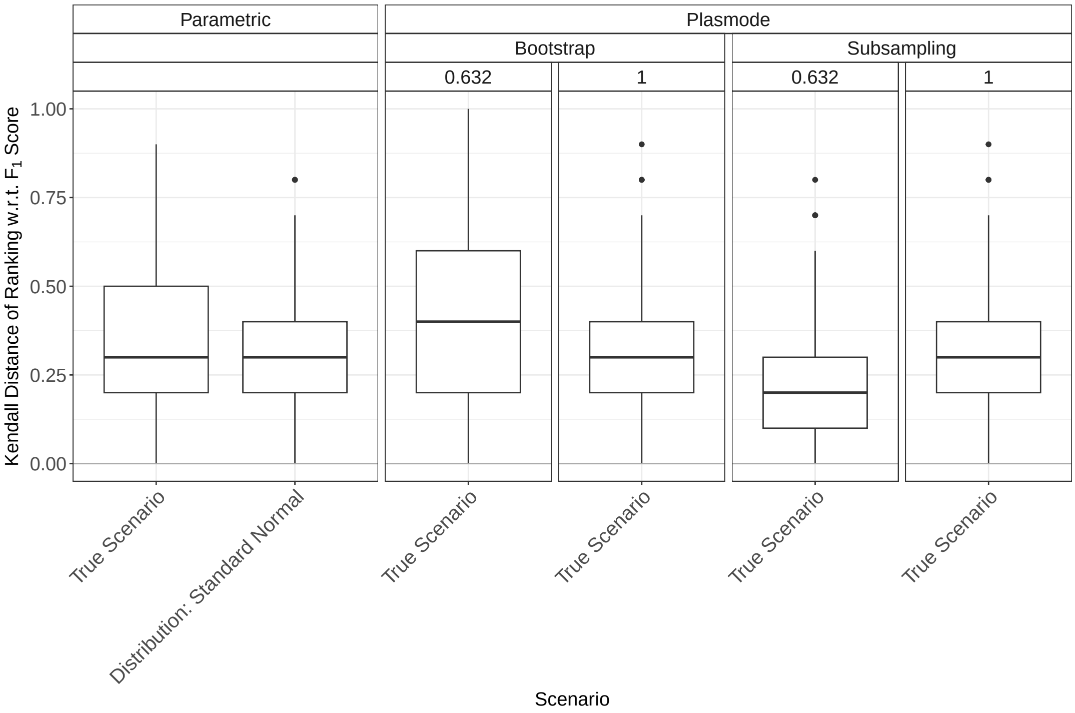

Fig H.26: Kendall distance of the simulated and true method ranking based on F_1-score in 100 iterations of a classification method comparison study per classifier for different simulation approaches with misspecification of the distribution as standard normal for parametric simulation for $p = 2$.

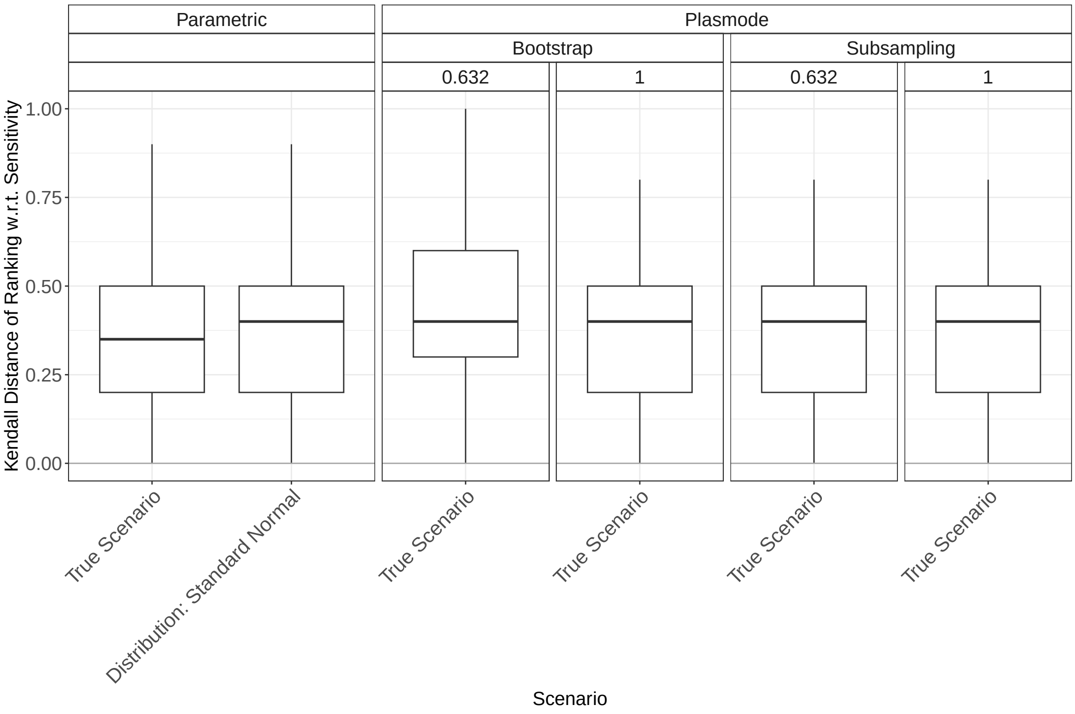

Fig H.27: Kendall distance of the simulated and true method ranking based on sensitivity in 100 iterations of a classification method comparison study per classifier for different simulation approaches with misspecification of the distribution as standard normal for parametric simulation for $p = 2$.

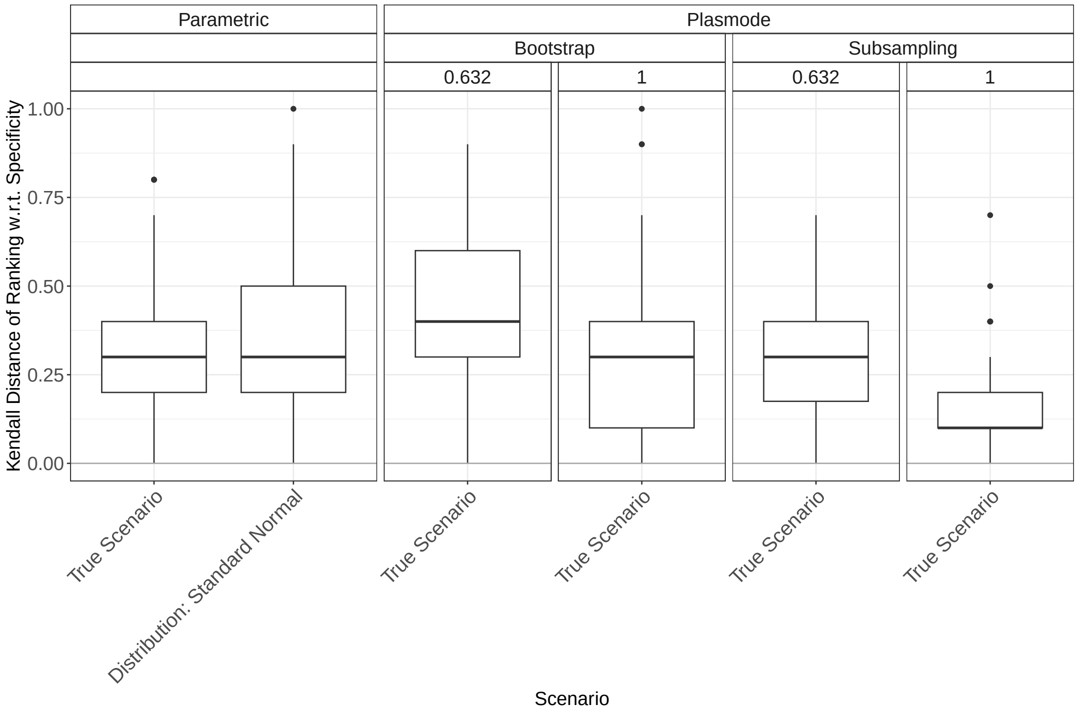

Fig H.28: Kendall distance of the simulated and true method ranking based on specificity in 100 iterations of a classification method comparison study per classifier for different simulation approaches with misspecification of the distribution as standard normal for parametric simulation for $p = 2$.

H.2 $p = 10$

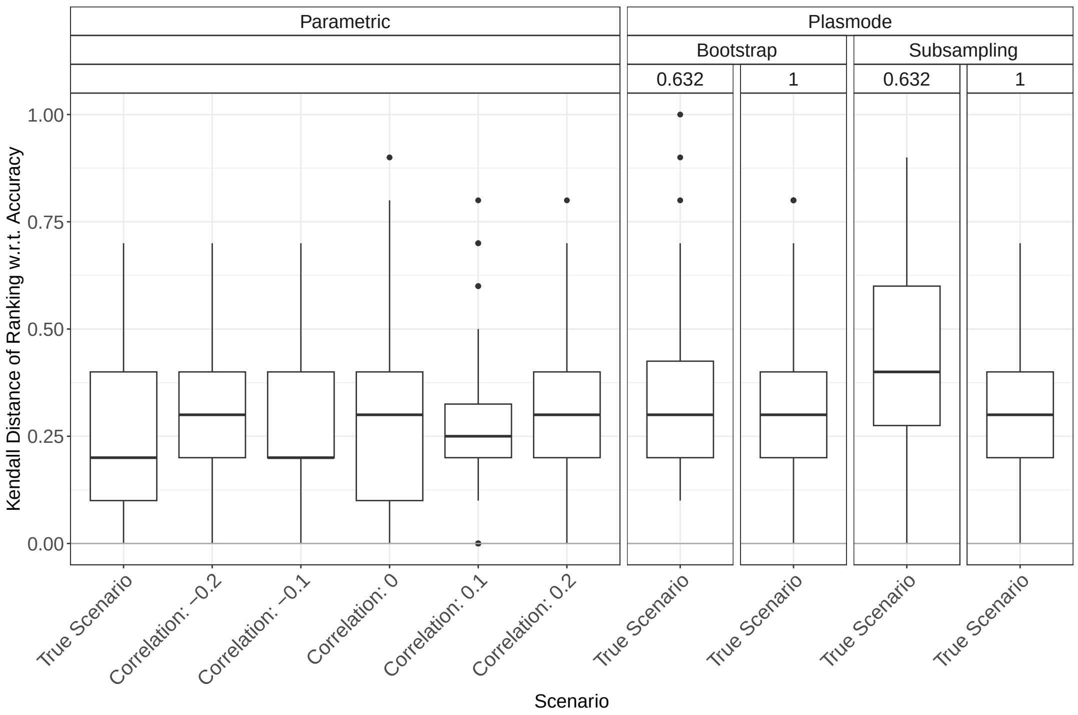

Fig H.29: Kendall distance of the simulated and true method ranking based on accuracy in 100 iterations of a classification method comparison study per classifier for different simulation approaches with misspecifications of the correlation for parametric simulation for $p = 10$.

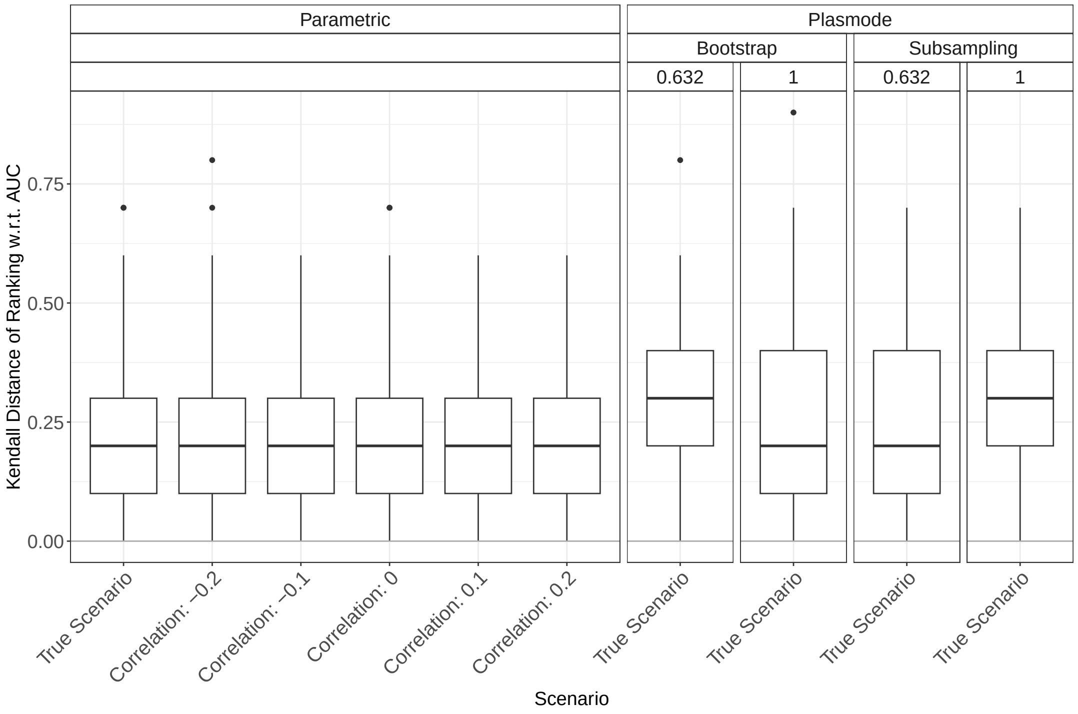

Fig H.30: Kendall distance of the simulated and true method ranking based on AUC in 100 iterations of a classification method comparison study per classifier for different simulation approaches with misspecifications of the correlation for parametric simulation for $p = 10$.

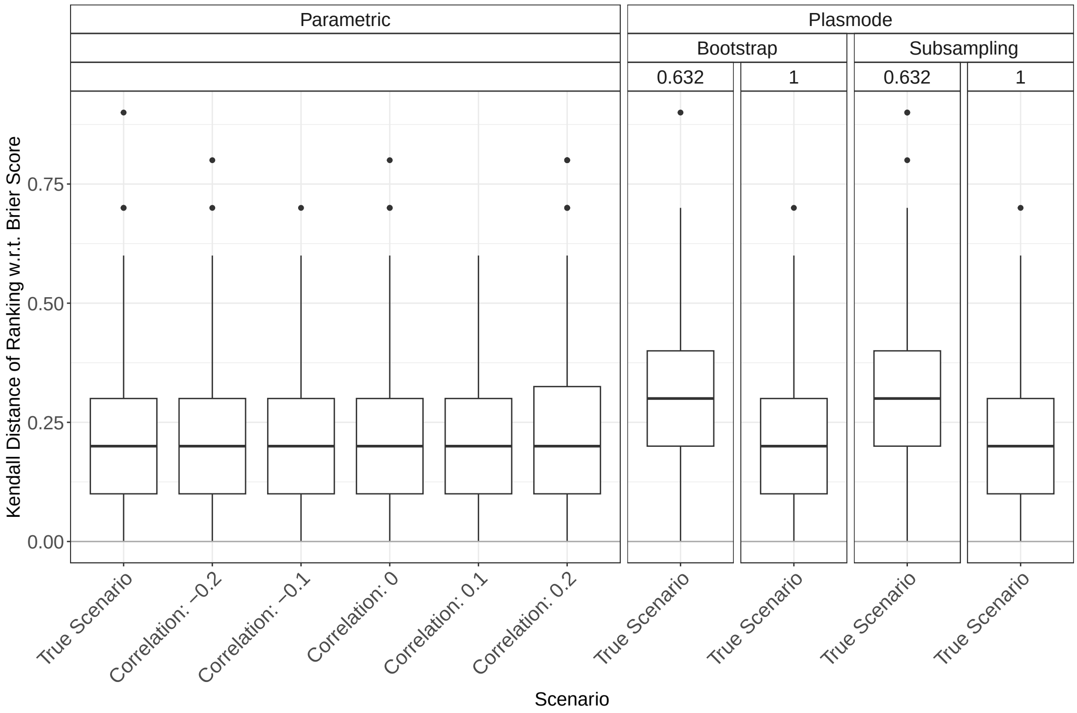

Fig H.31: Kendall distance of the simulated and true method ranking based on the Brier score in 100 iterations of a classification method comparison study per classifier for different simulation approaches with misspecifications of the correlation for parametric simulation for $p = 10$.

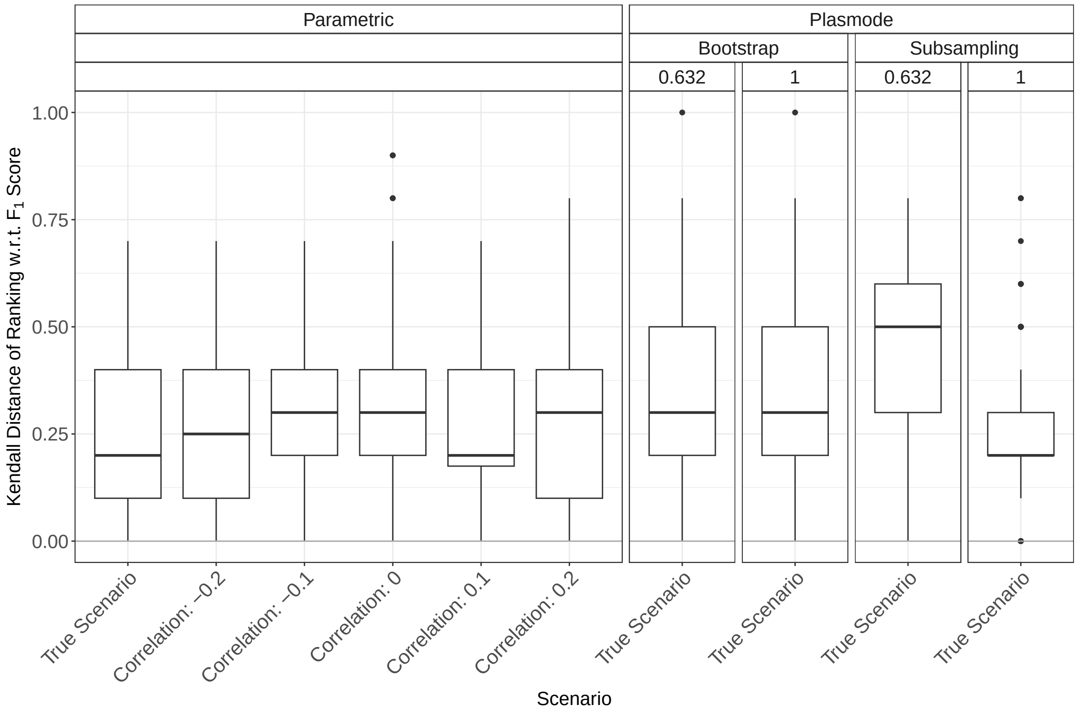

Fig H.32: Kendall distance of the simulated and true method ranking based on F_1-score in 100 iterations of a classification method comparison study per classifier for different simulation approaches with misspecifications of the correlation for parametric simulation for $p = 10$.

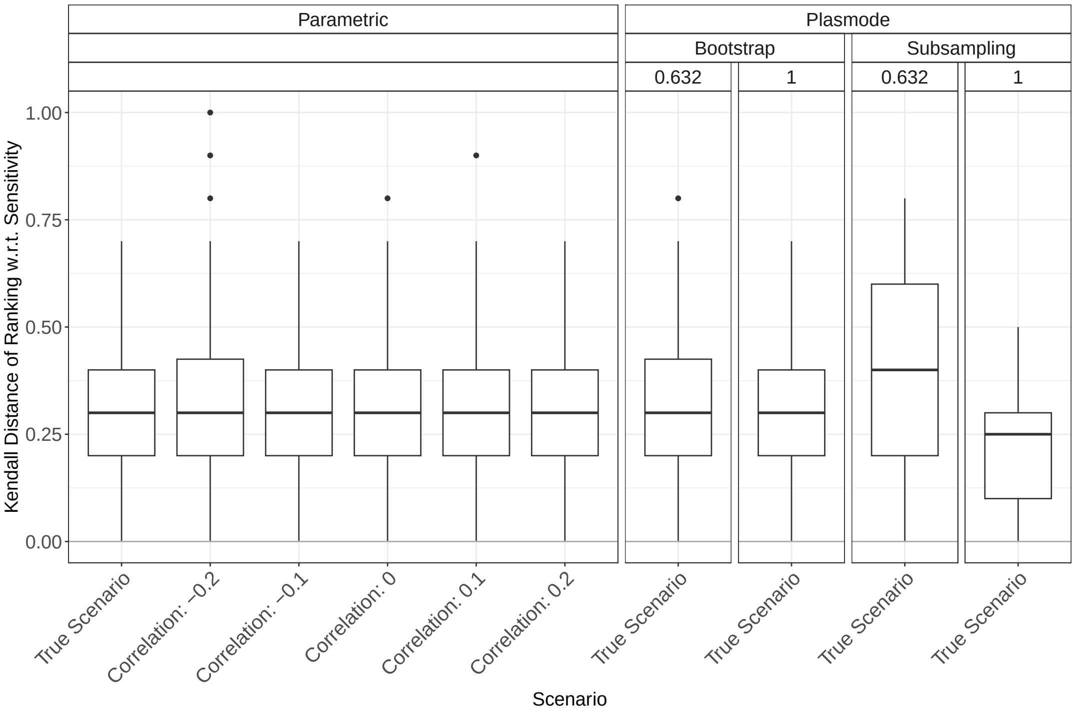

Fig H.33: Kendall distance of the simulated and true method ranking based on sensitivity in 100 iterations of a classification method comparison study per classifier for different simulation approaches with misspecifications of the correlation for parametric simulation for $p = 10$.

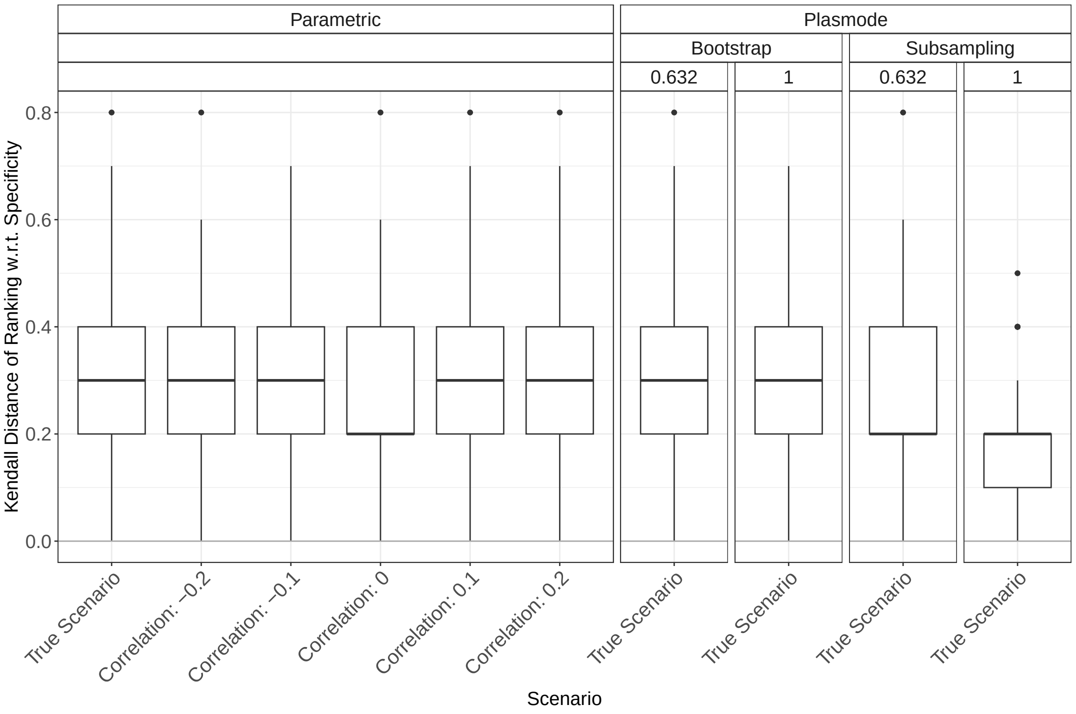

Fig H.34: Kendall distance of the simulated and true method ranking based on specificity in 100 iterations of a classification method comparison study per classifier for different simulation approaches with misspecifications of the correlation for parametric simulation for $p = 10$.

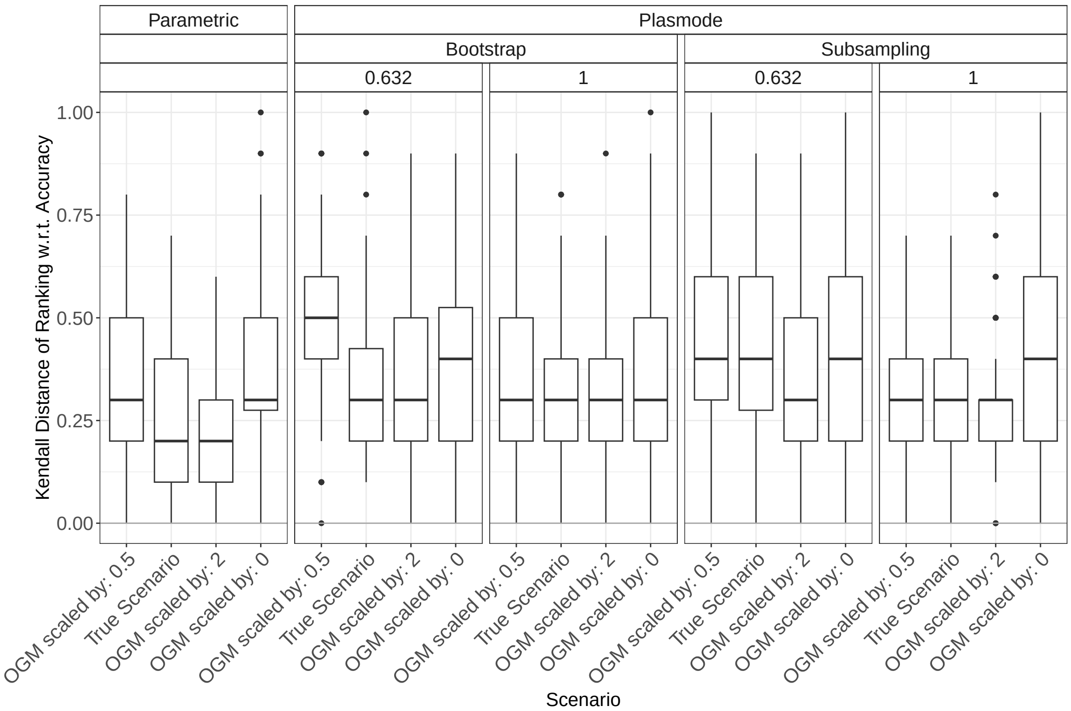

Fig H.35: Kendall distance of the simulated and true method ranking based on accuracy in 100 iterations of a classification method comparison study per classifier for different simulation approaches with misspecifications of the OGM for $p = 10$.

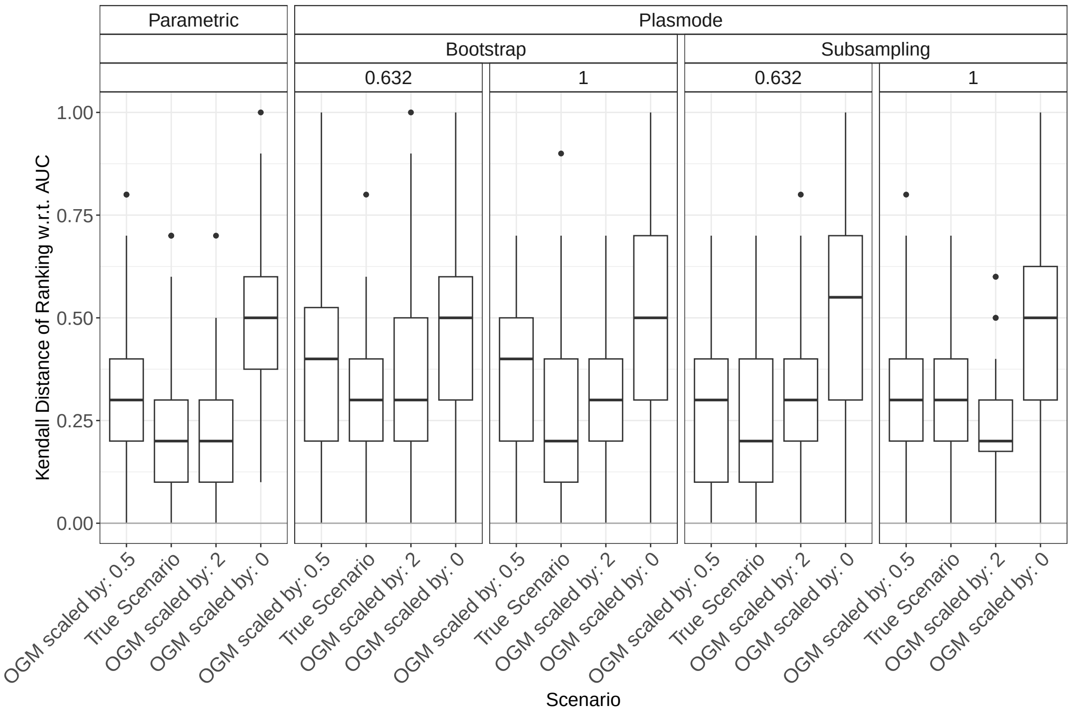

Fig H.36: Kendall distance of the simulated and true method ranking based on AUC in 100 iterations of a classification method comparison study per classifier for different simulation approaches with misspecifications of the OGMfor $p = 10$.

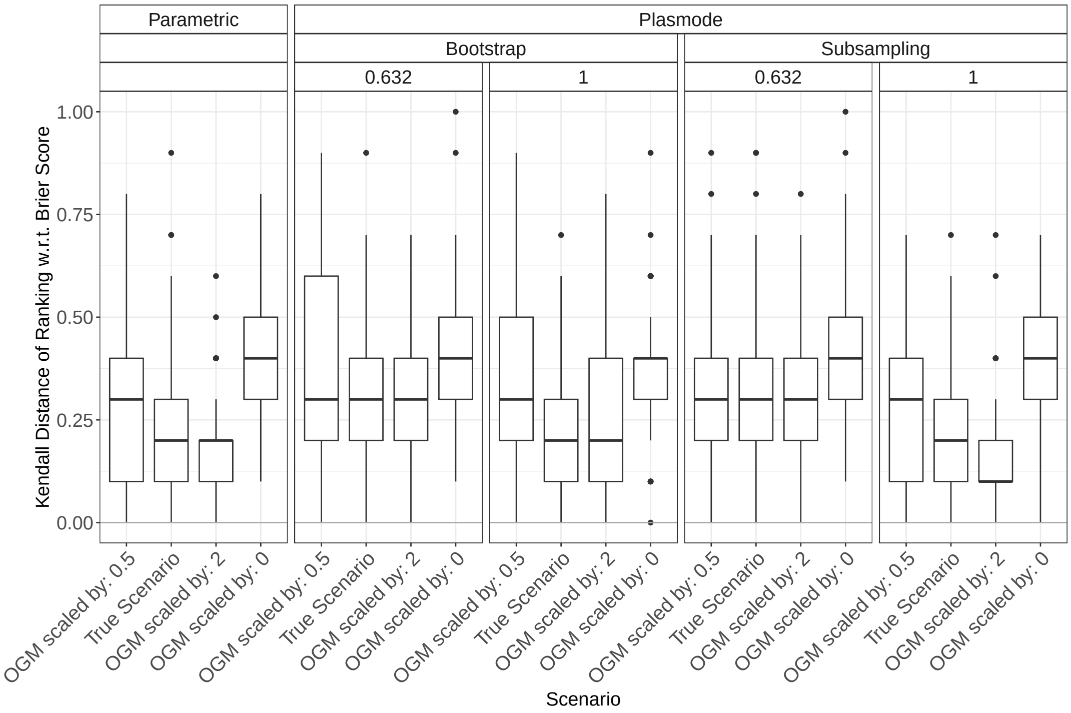

Fig H.37: Kendall distance of the simulated and true method ranking based on the Brier score in 100 iterations of a classification method comparison study per classifier for different simulation approaches with misspecifications of the OGM for $p = 10$.

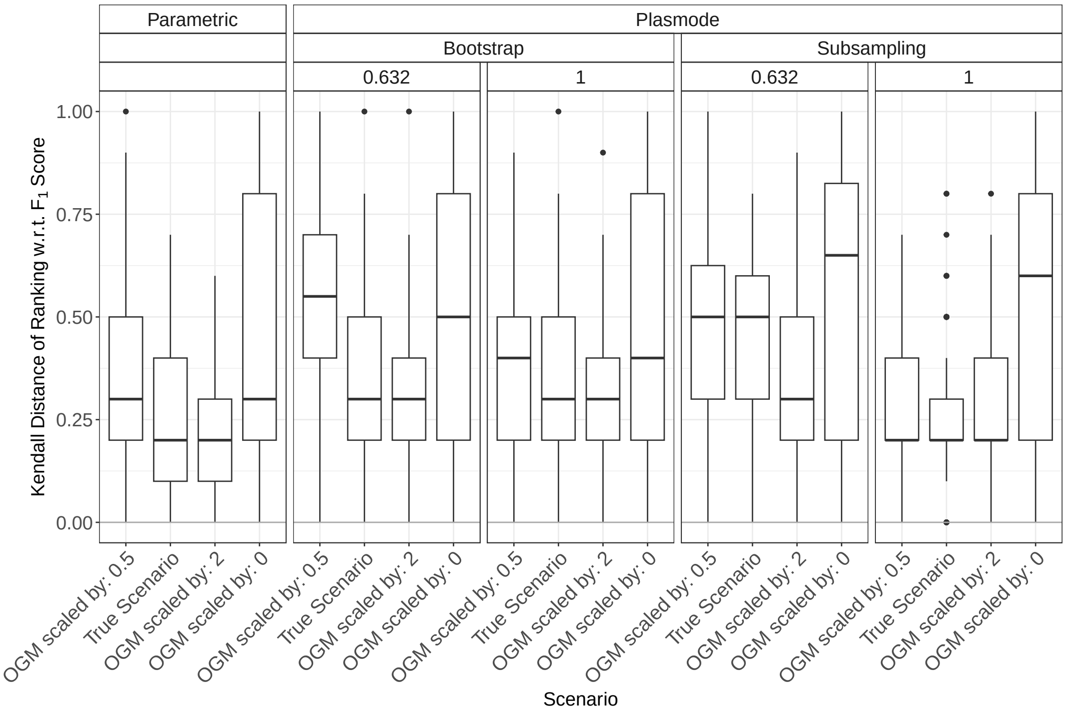

Fig H.38: Kendall distance of the simulated and true method ranking based on F_1-score in 100 iterations of a classification method comparison study per classifier for different simulation approaches with misspecifications of the correlation for parametric simulation for $p = 10$.

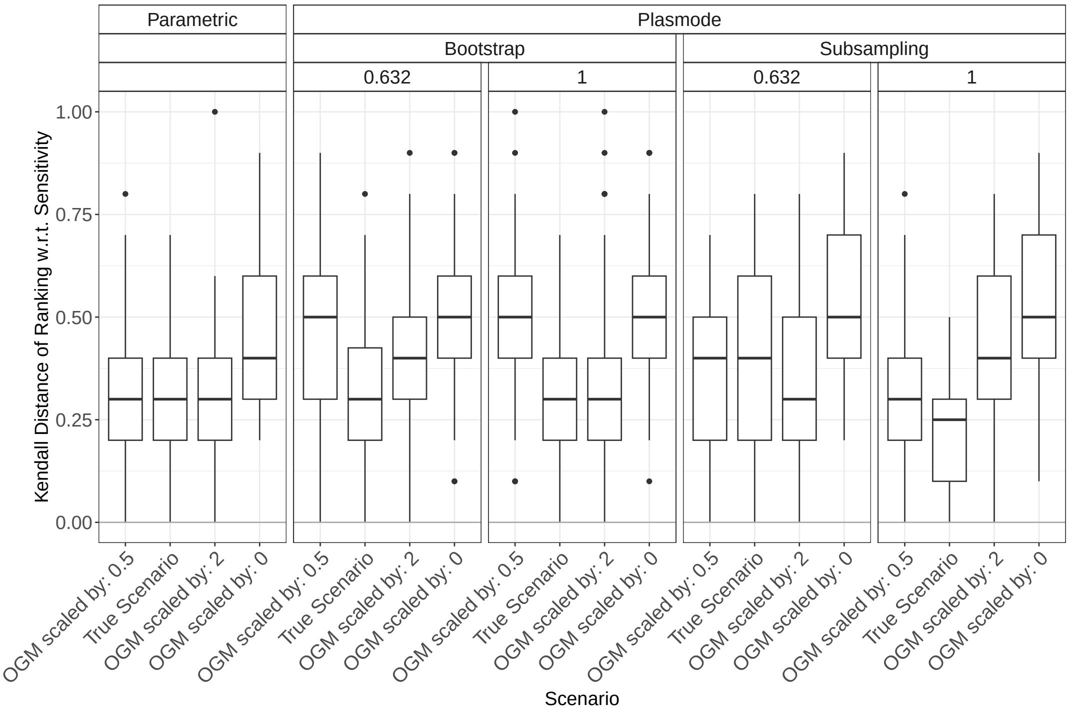

Fig H.39: Kendall distance of the simulated and true method ranking based on sensitivity in 100 iterations of a classification method comparison study per classifier for different simulation approaches with misspecifications of the OGM for $p = 10$.

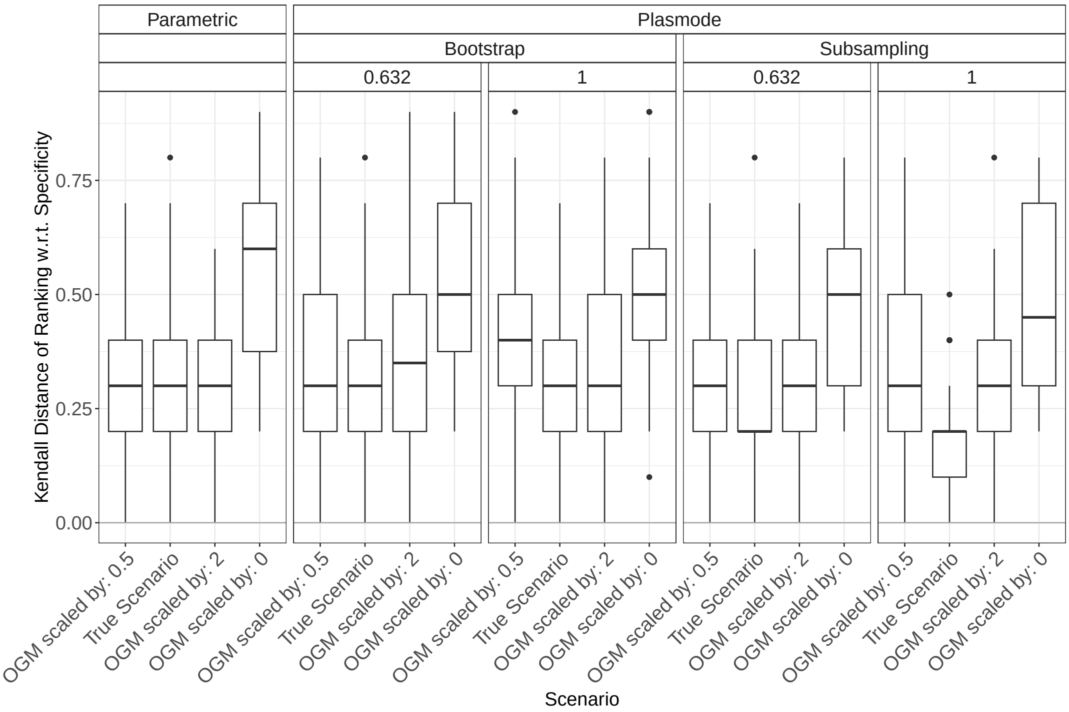

Fig H.40: Kendall distance of the simulated and true method ranking based on specificity in 100 iterations of a classification method comparison study per classifier for different simulation approaches with misspecifications of the OGM for $p = 10$.

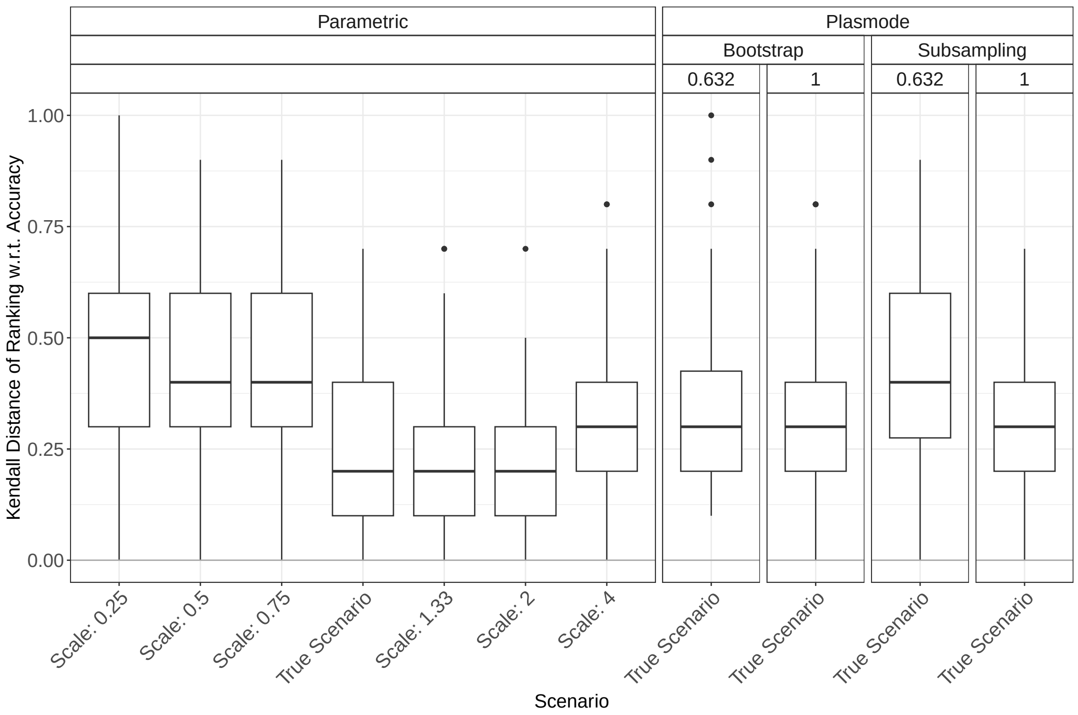

Fig H.41: Kendall distance of the simulated and true method ranking based on accuracy in 100 iterations of a classification method comparison study per classifier for different simulation approaches with misspecifications of the scale for parametric simulation for $p = 10$.

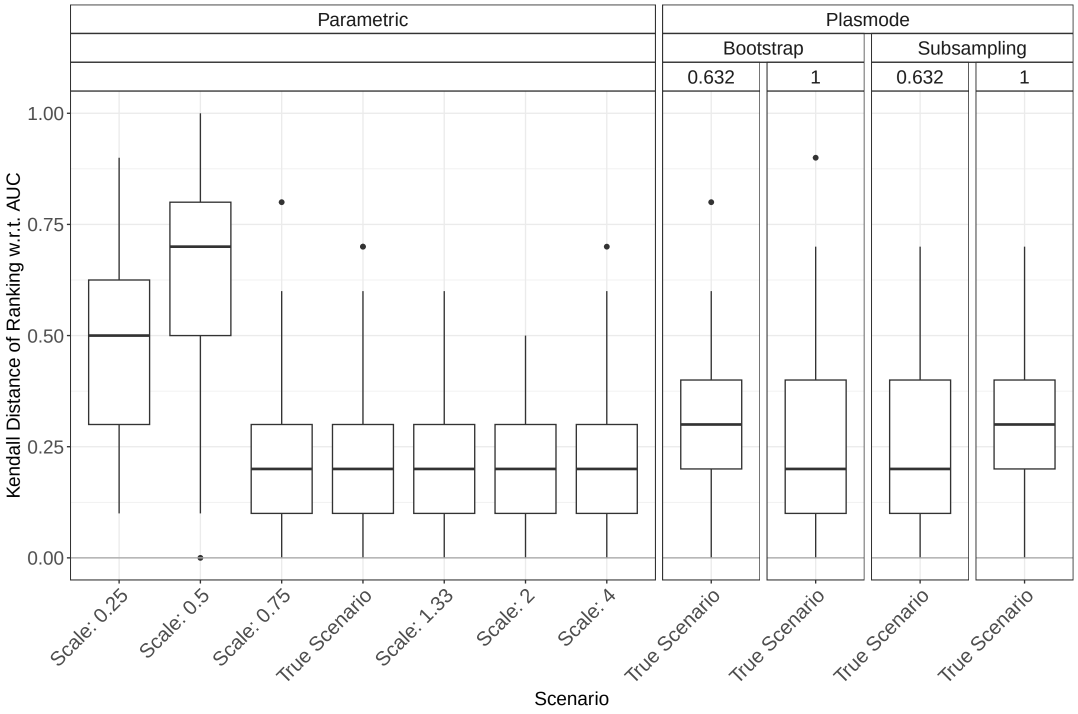

Fig H.42: Kendall distance of the simulated and true method ranking based on AUC in 100 iterations of a classification method comparison study per classifier for different simulation approaches with misspecifications of the scale for parametric simulation for $p = 10$.

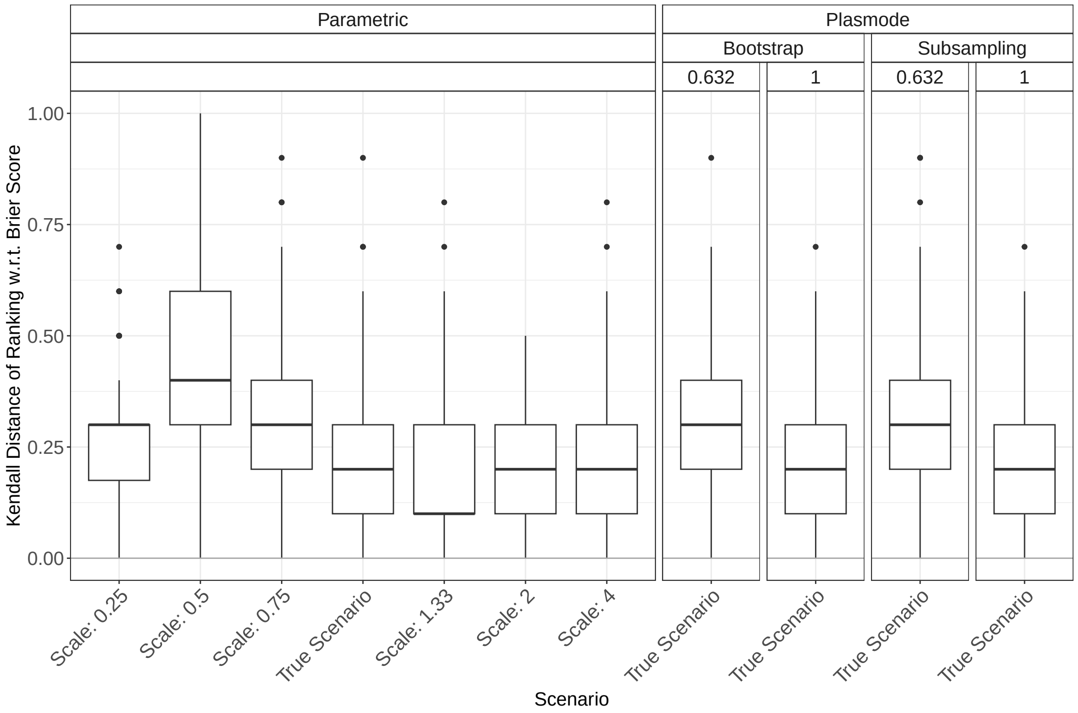

Fig H.43: Kendall distance of the simulated and true method ranking based on the Brier score in 100 iterations of a classification method comparison study per classifier for different simulation approaches with misspecifications of the scale for parametric simulation for $p = 10$.

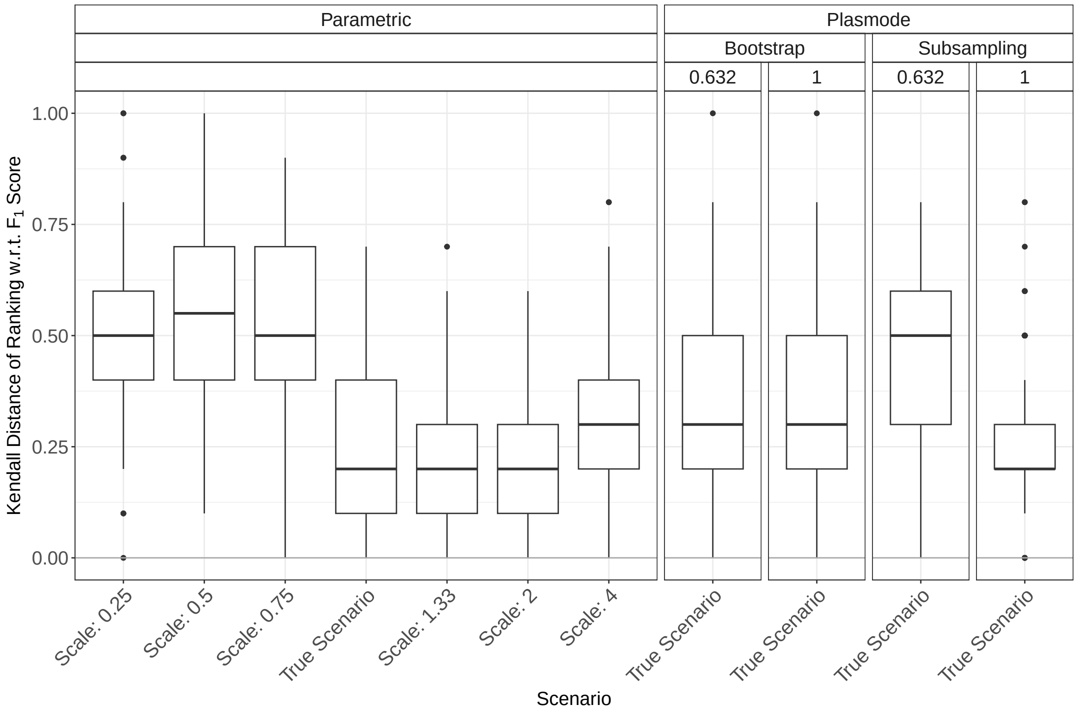

Fig H.44: Kendall distance of the simulated and true method ranking based on F_1-score in 100 iterations of a classification method comparison study per classifier for different simulation approaches with misspecifications of the scale for parametric simulation for $p = 10$.

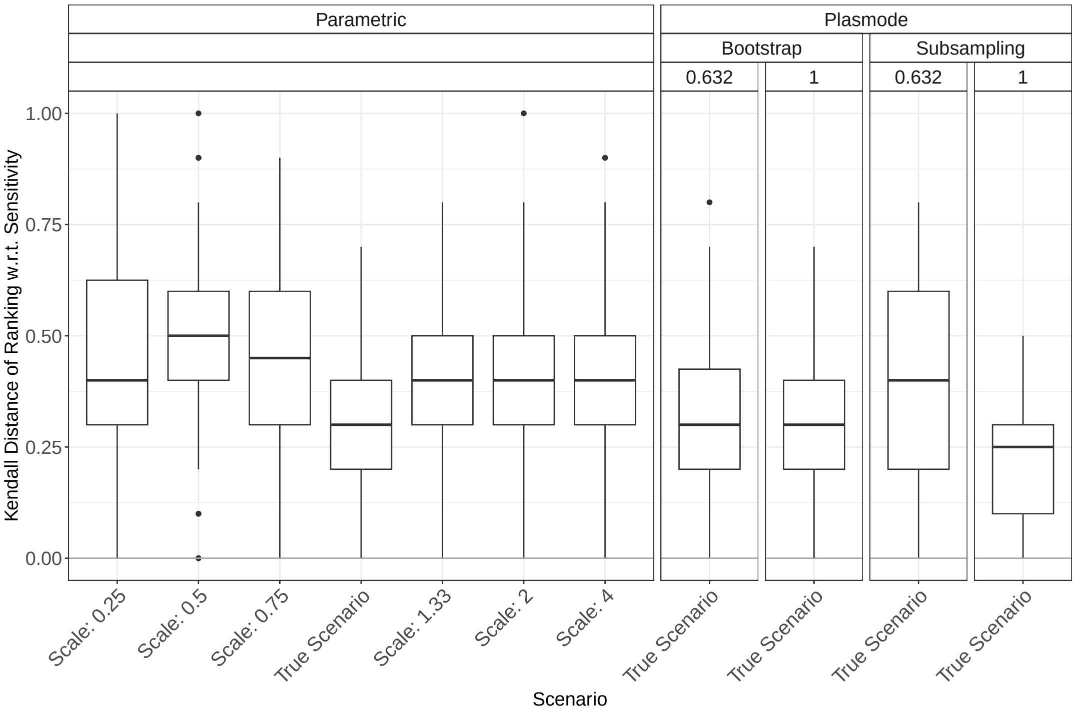

Fig H.45: Kendall distance of the simulated and true method ranking based on sensitivity in 100 iterations of a classification method comparison study per classifier for different simulation approaches with misspecifications of the scale for parametric simulation for $p = 10$.

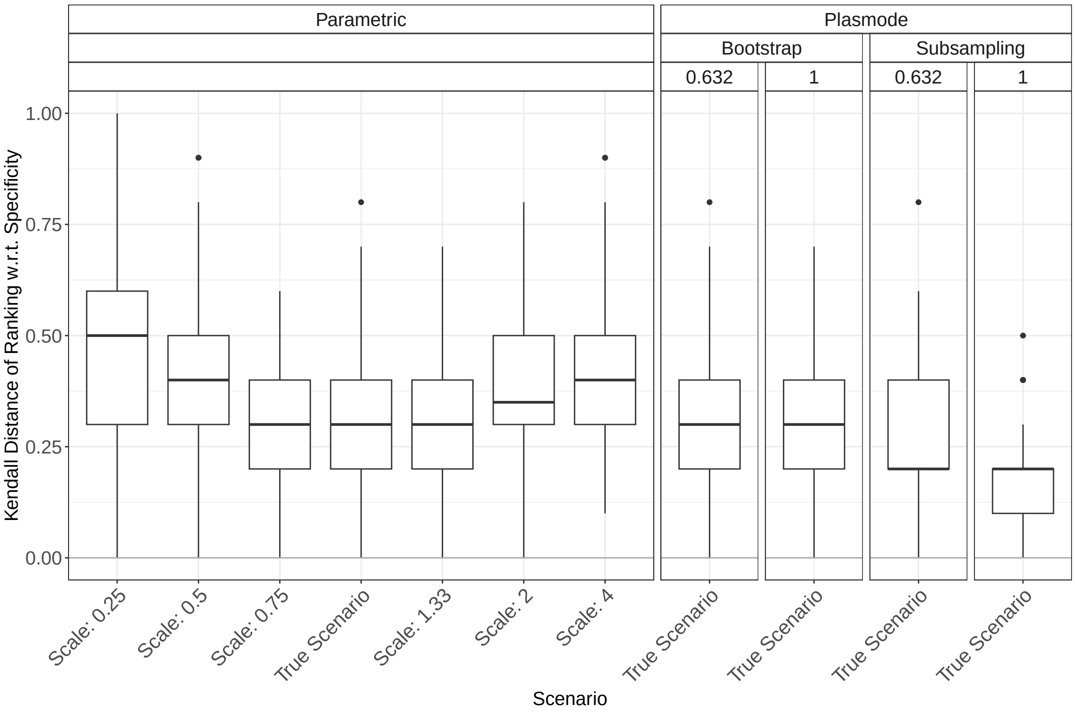

Fig H.46: Kendall distance of the simulated and true method ranking based on specificity in 100 iterations of a classification method comparison study per classifier for different simulation approaches with misspecifications of the scale for parametric simulation for $p = 10$.

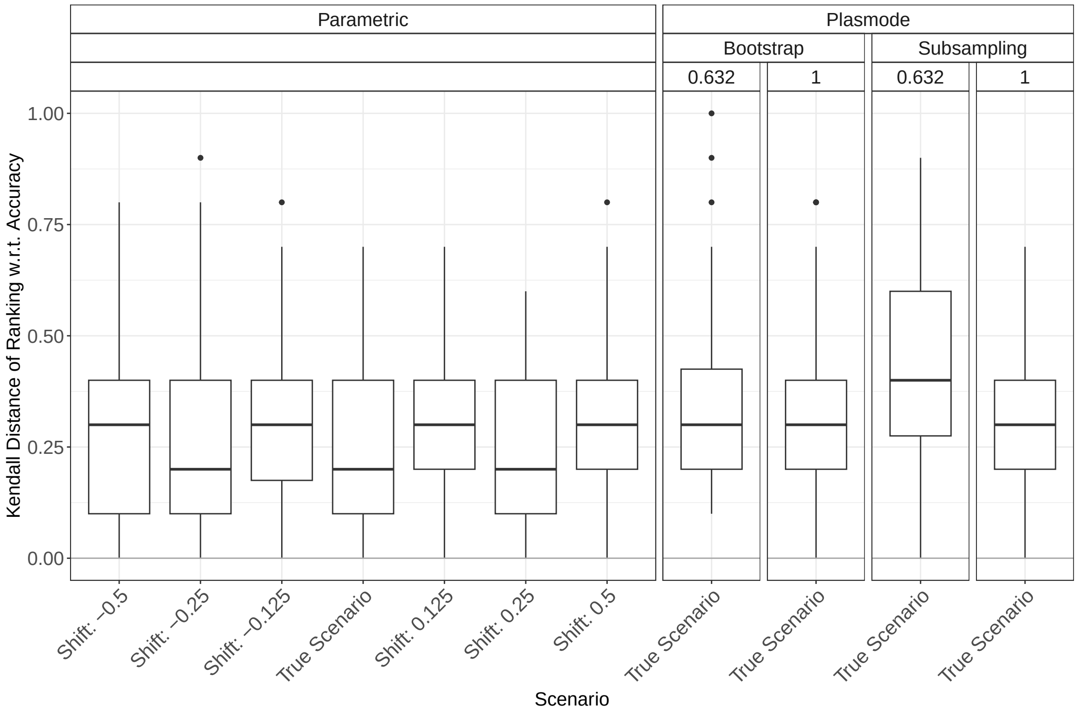

Fig H.47: Kendall distance of the simulated and true method ranking based on accuracy in 100 iterations of a classification method comparison study per classifier for different simulation approaches with misspecifications of the shift for parametric simulation for $p = 10$.

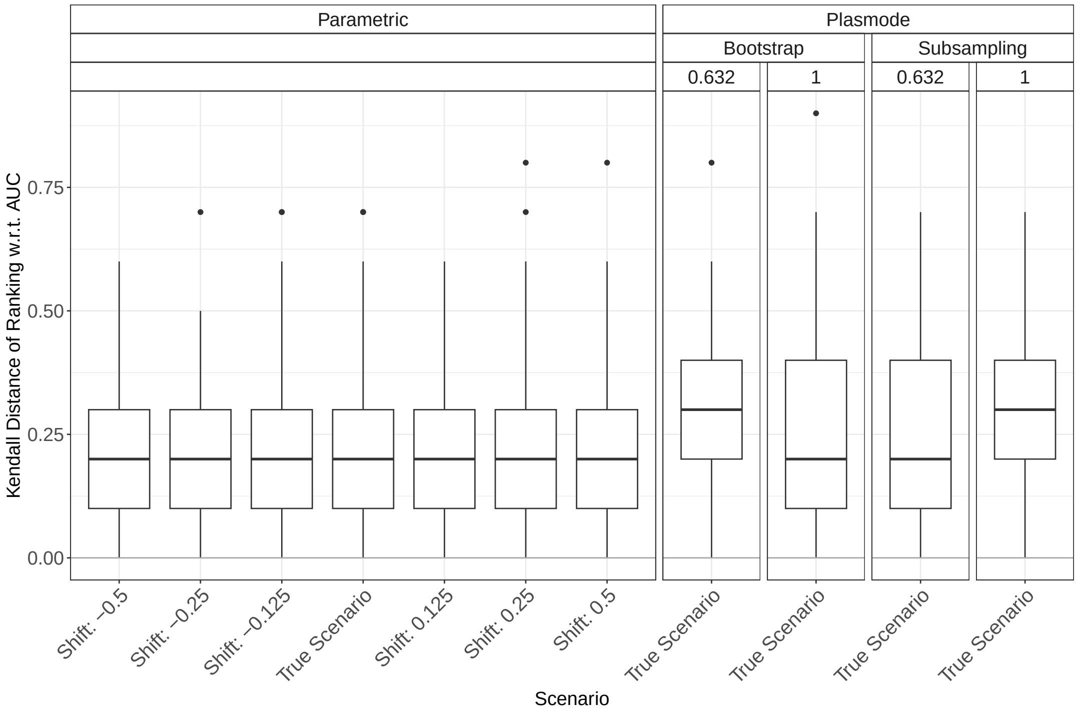

Fig H.48: Kendall distance of the simulated and true method ranking based on AUC in 100 iterations of a classification method comparison study per classifier for different simulation approaches with misspecifications of the shift for parametric simulation for $p = 10$.

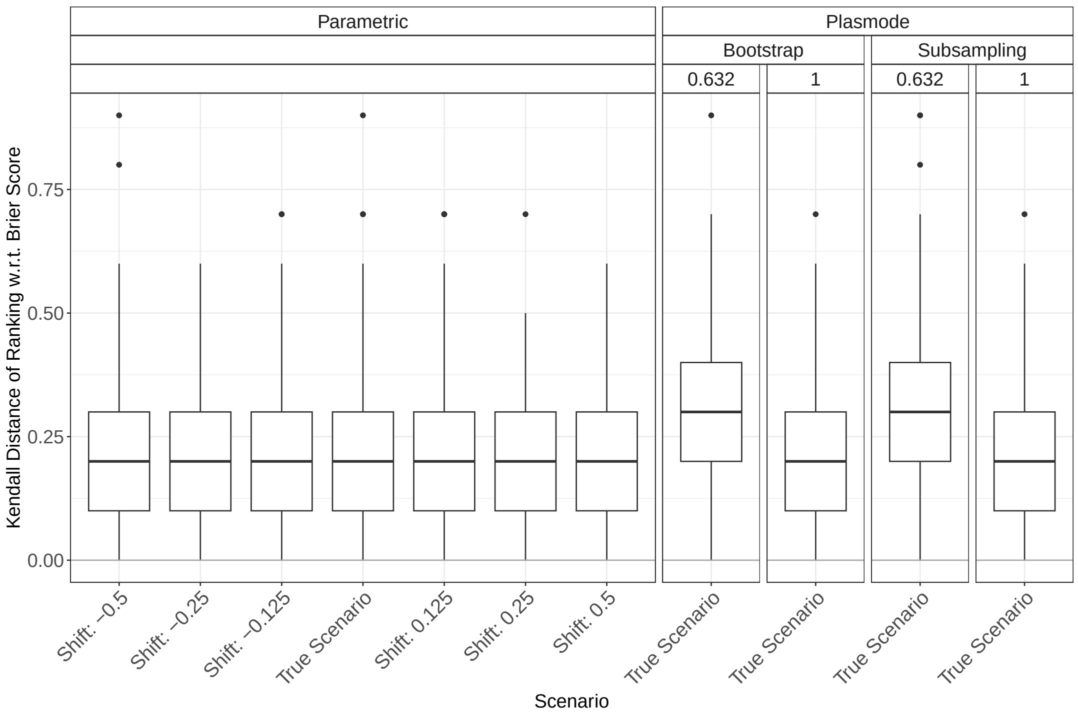

Fig H.49: Kendall distance of the simulated and true method ranking based on the Brier score in 100 iterations of a classification method comparison study per classifier for different simulation approaches with misspecifications of the shift for parametric simulation for $p = 10$.

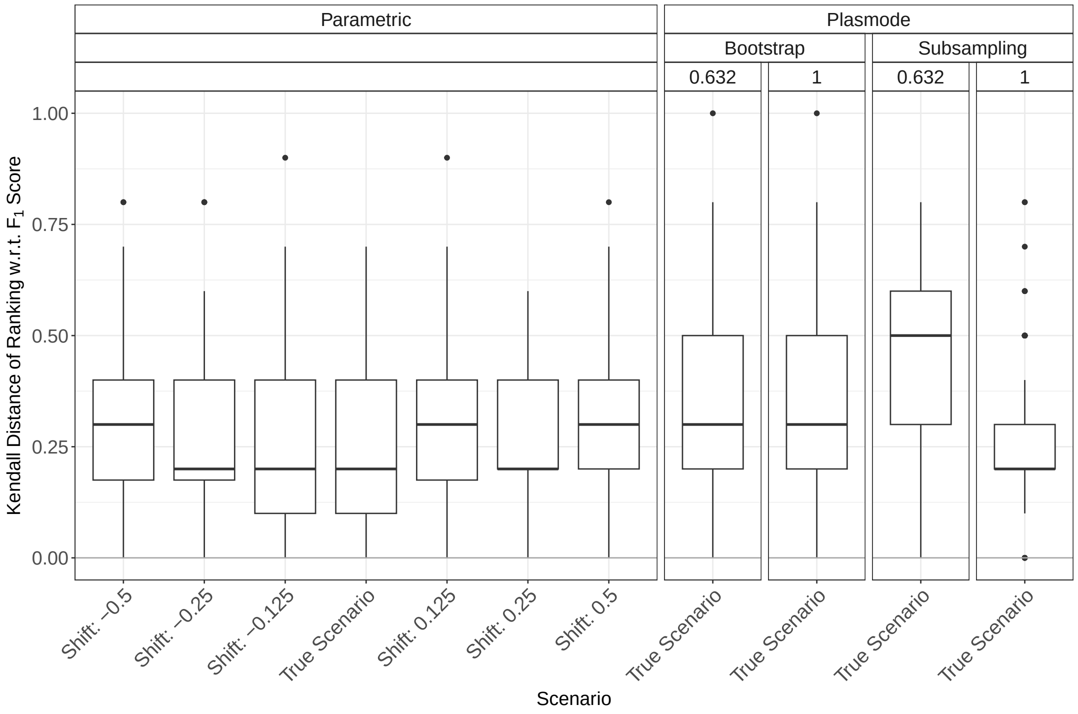

Fig H.50: Kendall distance of the simulated and true method ranking based on F_1-score in 100 iterations of a classification method comparison study per classifier for different simulation approaches with misspecifications of the shift for parametric simulation for $p = 10$.

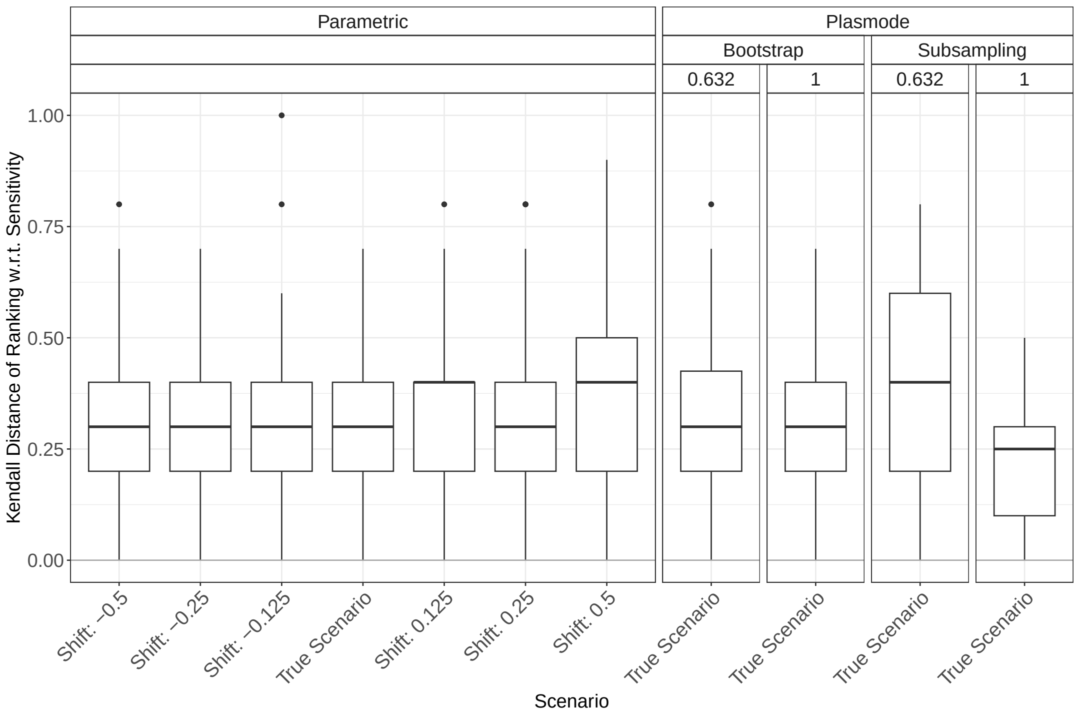

Fig H.51: Kendall distance of the simulated and true method ranking based on sensitivity in 100 iterations of a classification method comparison study per classifier for different simulation approaches with misspecifications of the shift for parametric simulation for $p = 10$.

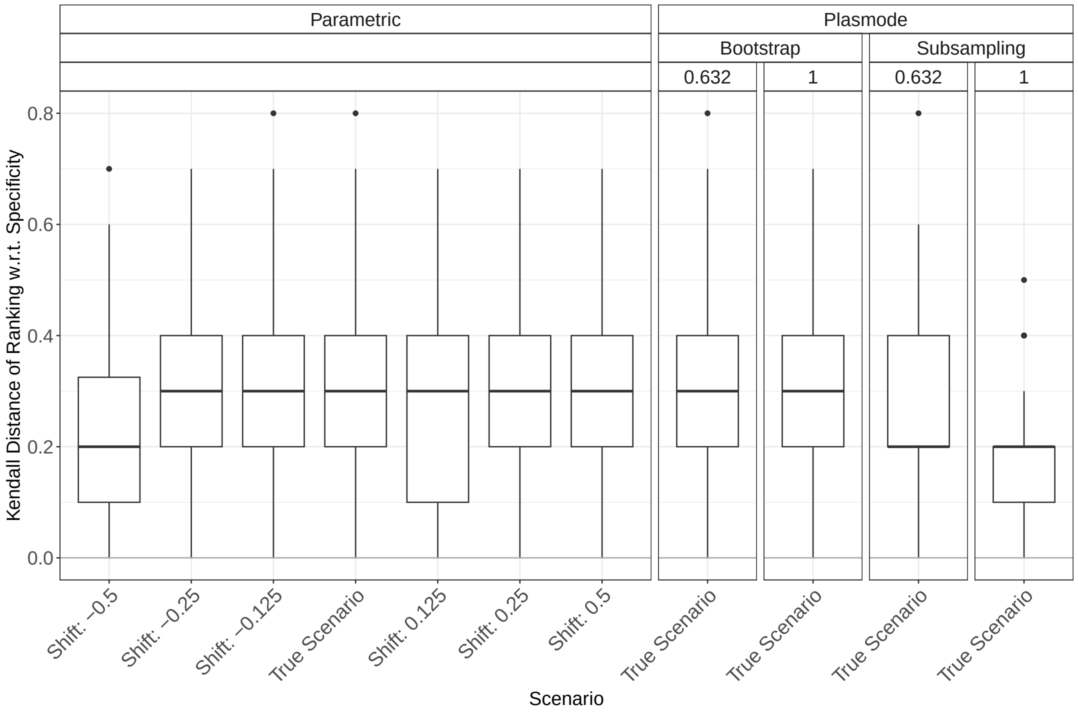

Fig H.52: Kendall distance of the simulated and true method ranking based on specificity in 100 iterations of a classification method comparison study per classifier for different simulation approaches with misspecifications of the shift for parametric simulation for $p = 10$.

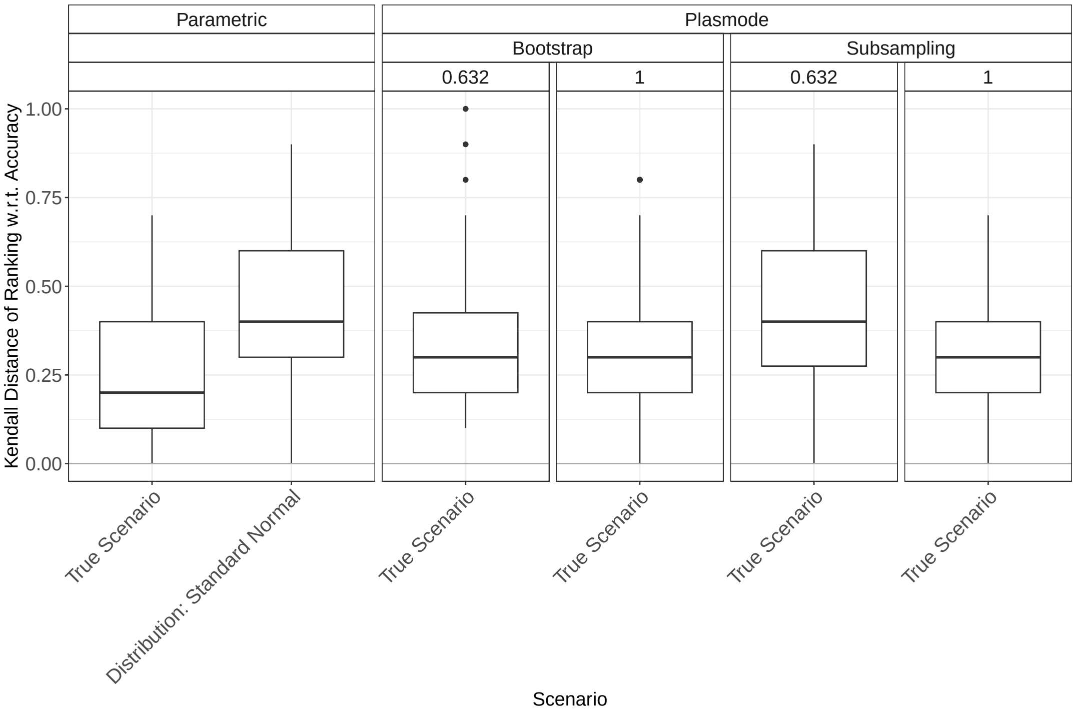

Fig H.53: Kendall distance of the simulated and true method ranking based on accuracy in 100 iterations of a classification method comparison study per classifier for different simulation approaches with misspecification of the distribution as standard normal for parametric simulation for $p = 10$.

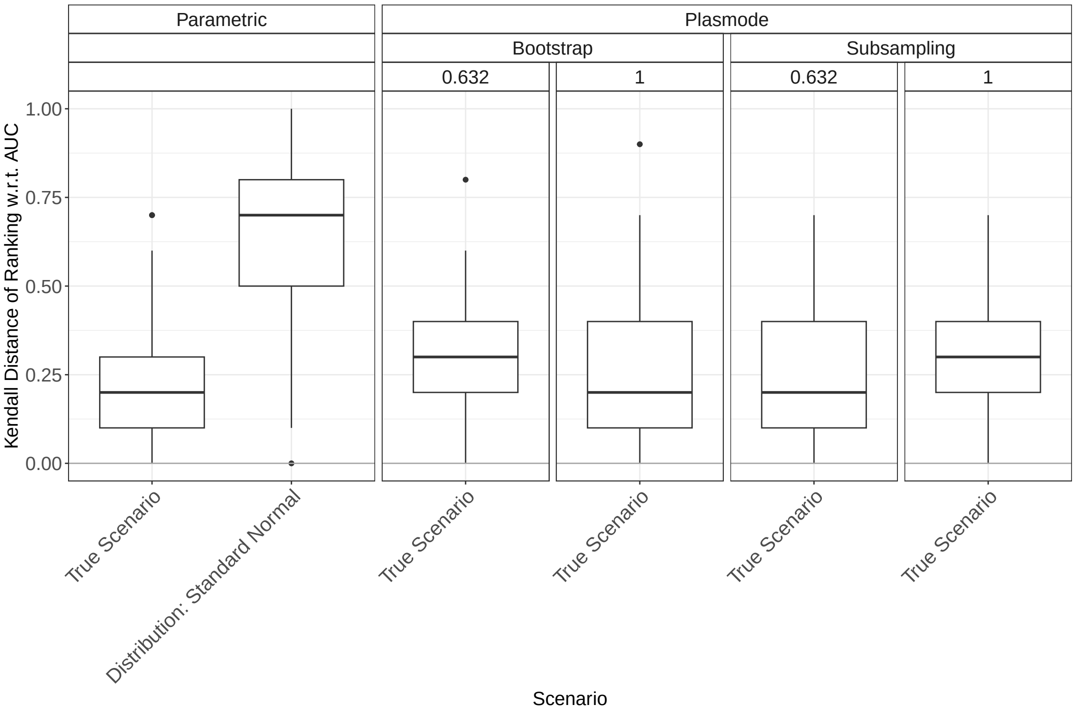

Fig H.54: Kendall distance of the simulated and true method ranking based on AUC in 100 iterations of a classification method comparison study per classifier for different simulation approaches with misspecification of the distribution as standard normal for parametric simulation for $p = 10$.

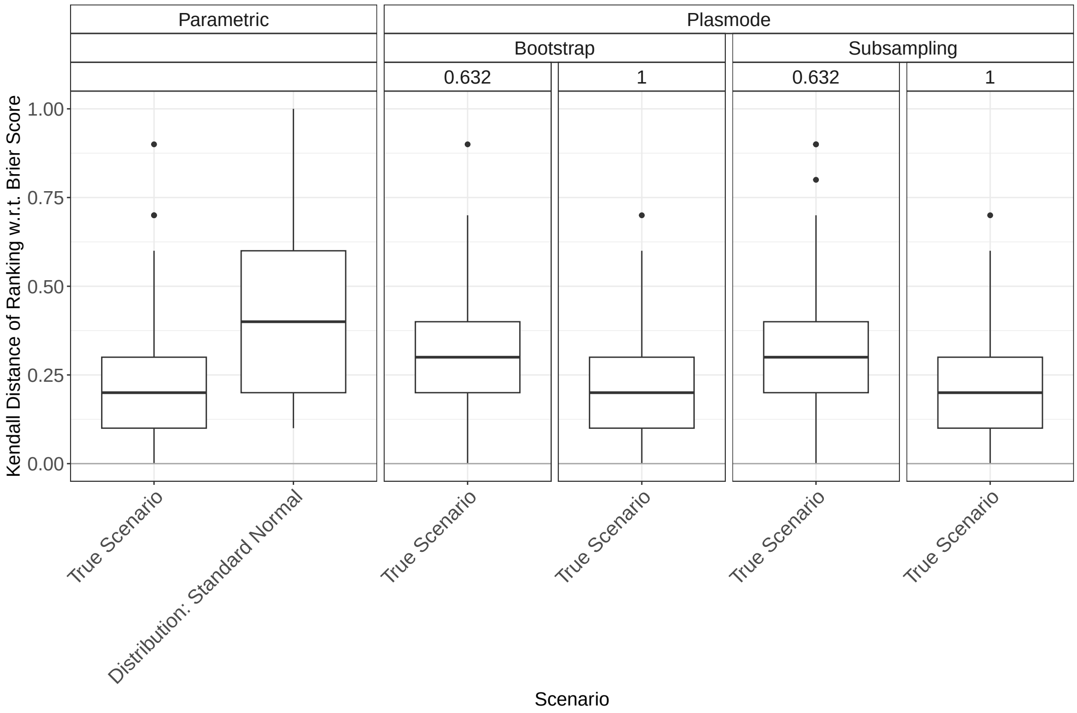

Fig H.55: Kendall distance of the simulated and true method ranking based on the Brier score in 100 iterations of a classification method comparison study per classifier for different simulation approaches with misspecification of the distribution as standard normal for parametric simulation for $p = 10$.

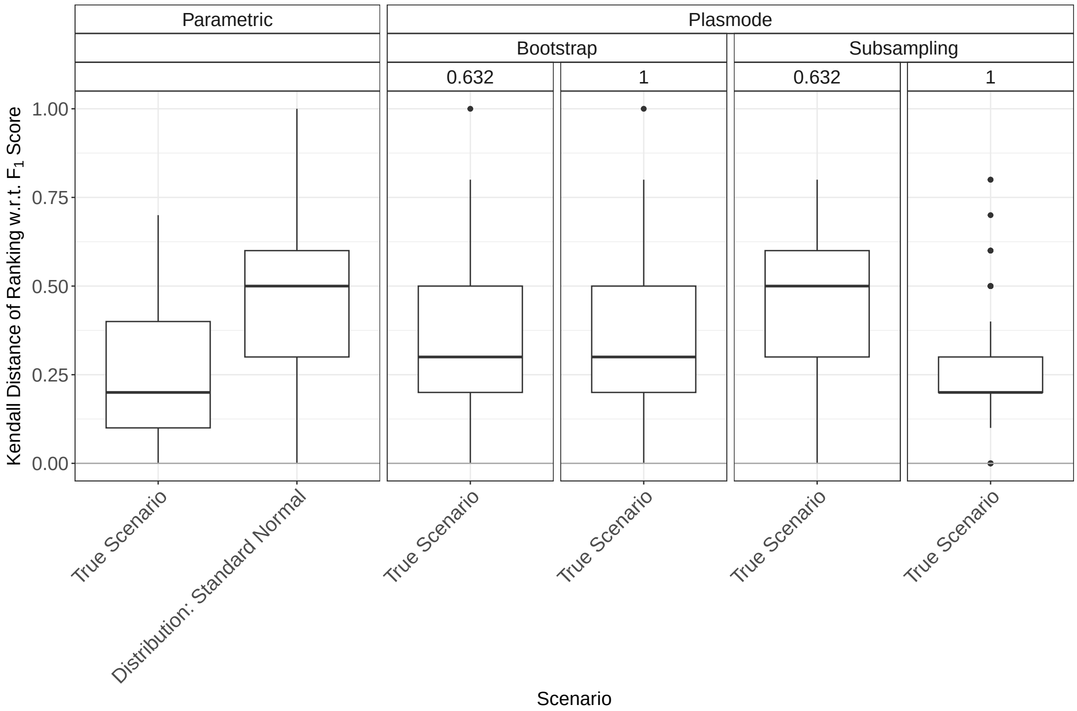

Fig H.56: Kendall distance of the simulated and true method ranking based on F_1-score in 100 iterations of a classification method comparison study per classifier for different simulation approaches with misspecification of the distribution as standard normal for parametric simulation for $p = 10$.

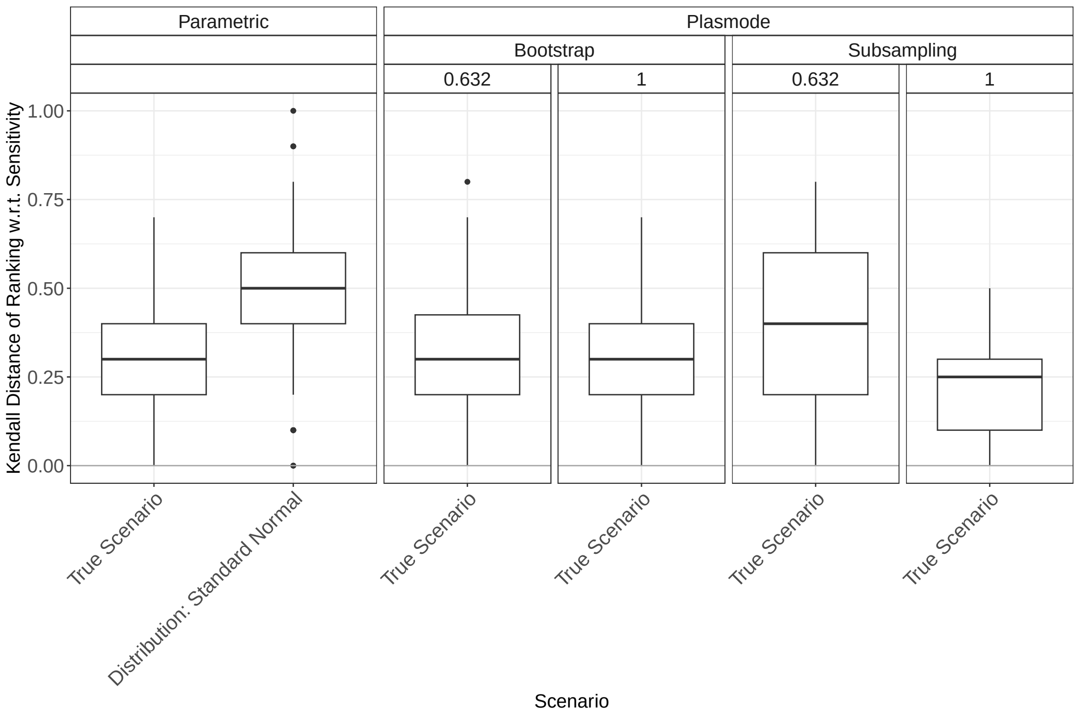

Fig H.57: Kendall distance of the simulated and true method ranking based on sensitivity in 100 iterations of a classification method comparison study per classifier for different simulation approaches with misspecification of the distribution as standard normal for parametric simulation for $p = 10$.

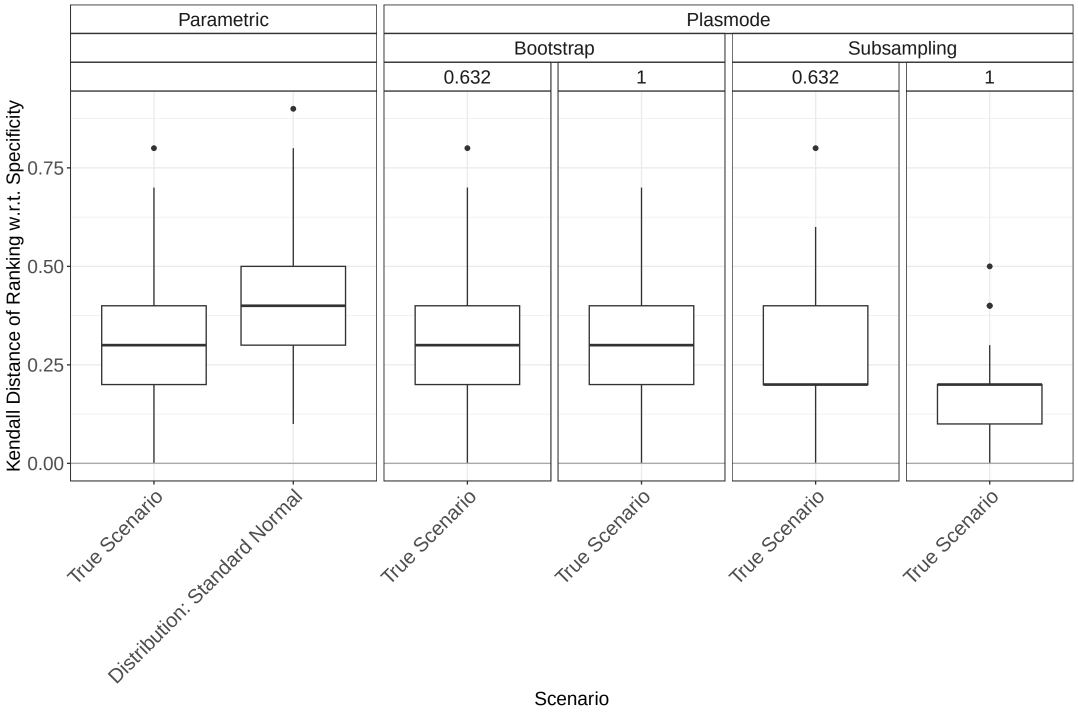

Fig H.58: Kendall distance of the simulated and true method ranking based on specificity in 100 iterations of a classification method comparison study per classifier for different simulation approaches with misspecification of the distribution as standard normal for parametric simulation for $p = 10$.

H.3 $p = 50$

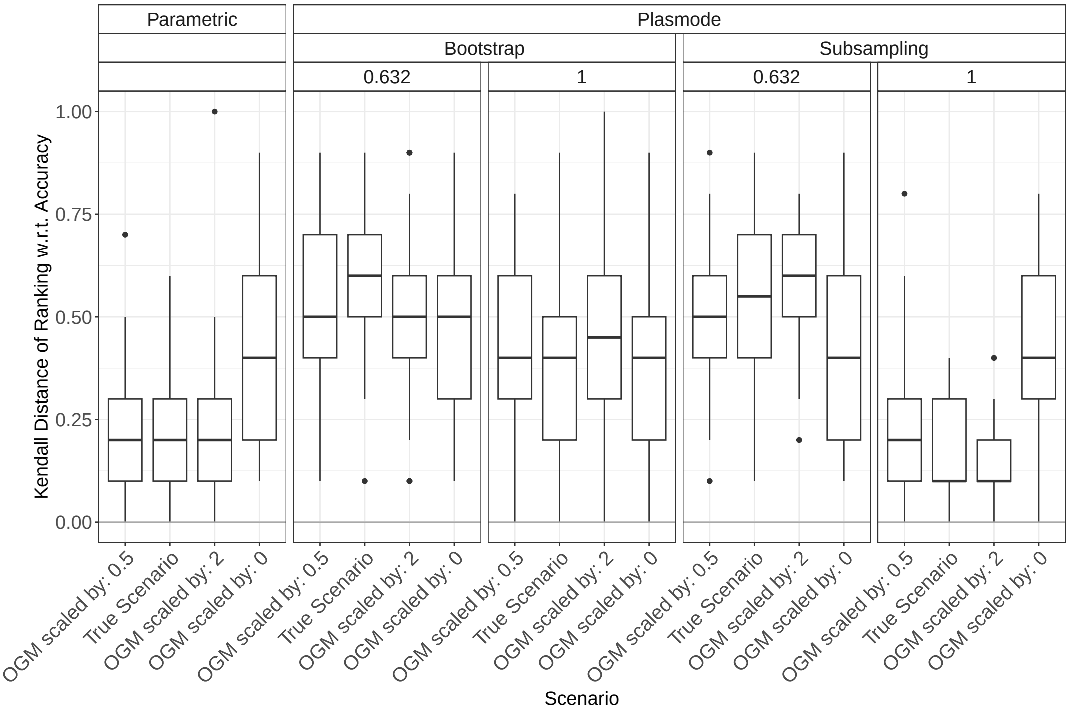

Fig H.59: Kendall distance of the simulated and true method ranking based on accuracy in 100 iterations of a classification method comparison study per classifier for different simulation approaches with misspecifications of the OGM for $p = 50$.

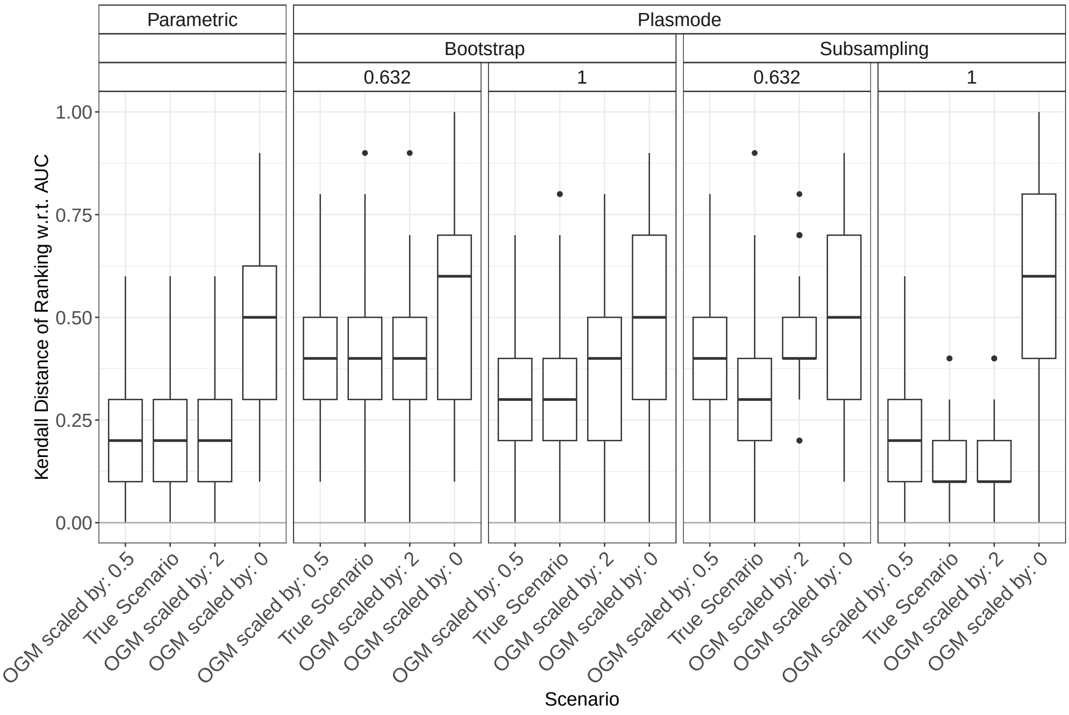

Fig H.60: Kendall distance of the simulated and true method ranking based on AUC in 100 iterations of a classification method comparison study per classifier for different simulation approaches with misspecifications of the OGMfor $p = 50$.

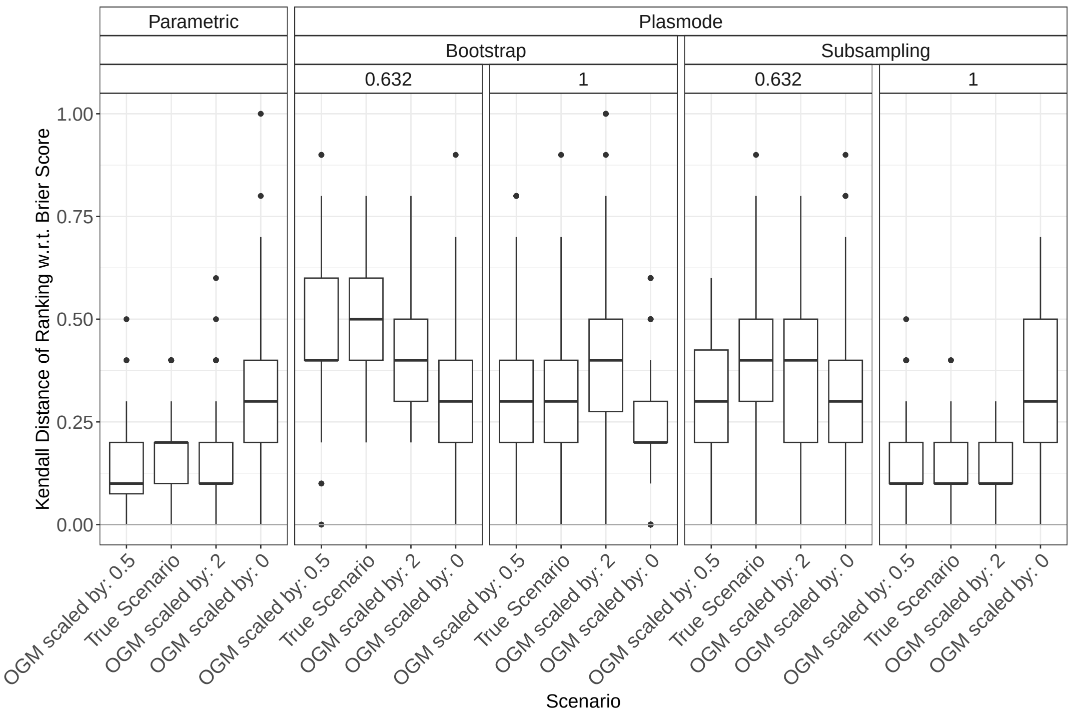

Fig H.61: Kendall distance of the simulated and true method ranking based on the Brier score in 100 iterations of a classification method comparison study per classifier for different simulation approaches with misspecifications of the OGM for $p = 50$.

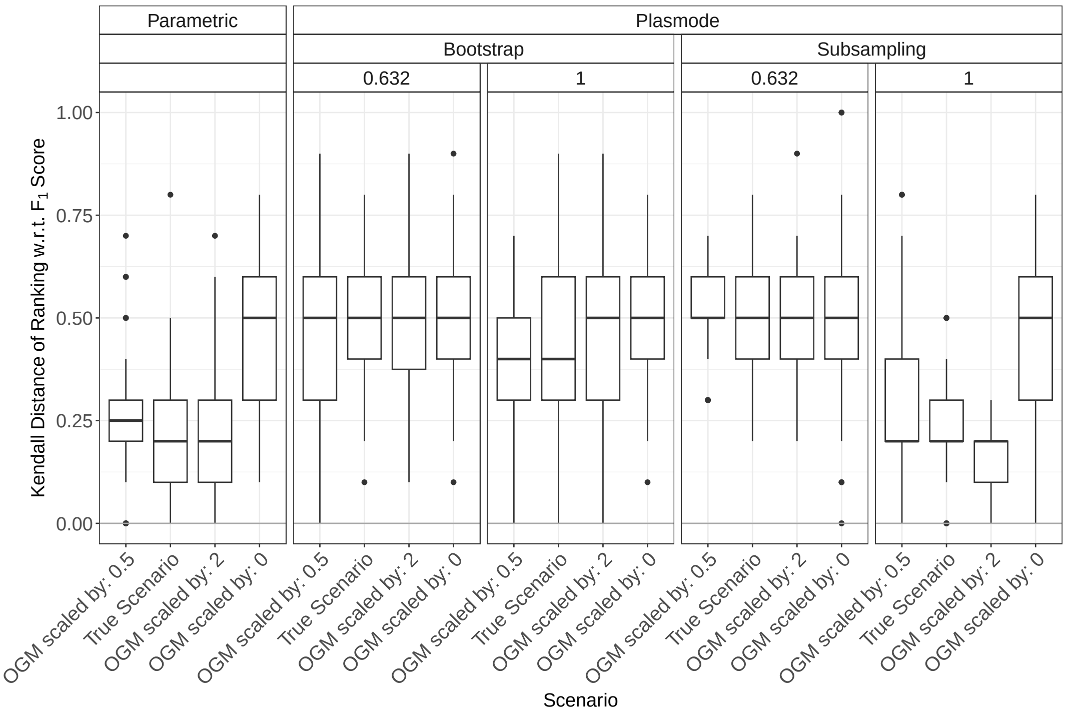

Fig H.62: Kendall distance of the simulated and true method ranking based on F_1-score in 100 iterations of a classification method comparison study per classifier for different simulation approaches with misspecifications of the correlation for parametric simulation for $p = 50$.

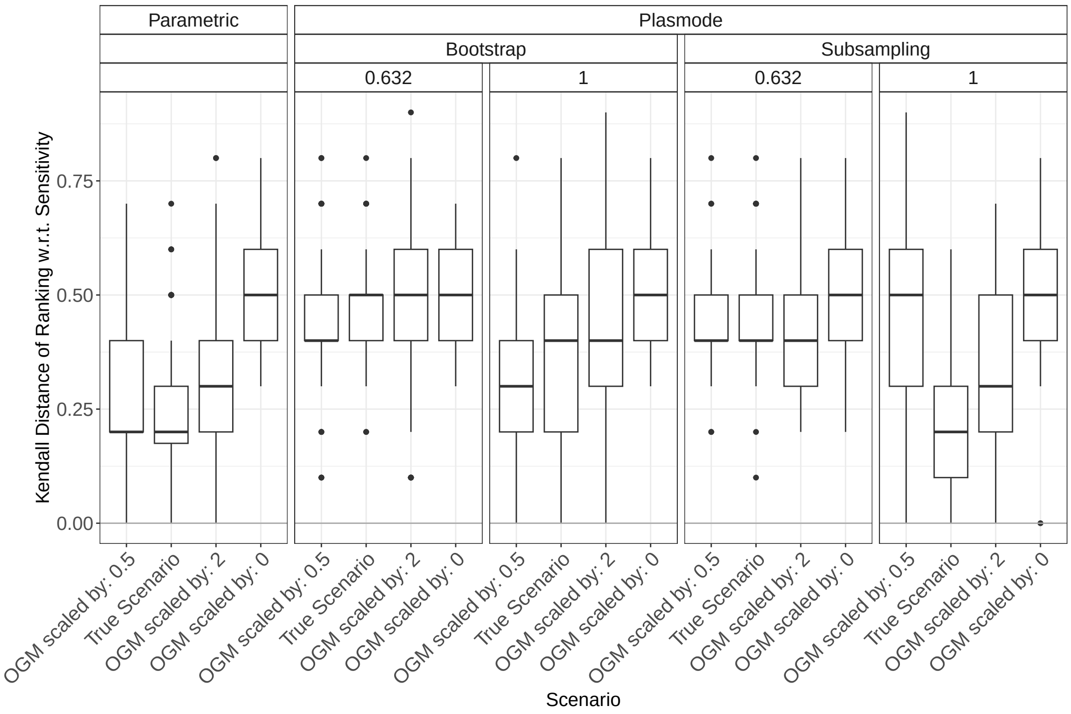

Fig H.63: Kendall distance of the simulated and true method ranking based on sensitivity in 100 iterations of a classification method comparison study per classifier for different simulation approaches with misspecifications of the OGM for $p = 50$.

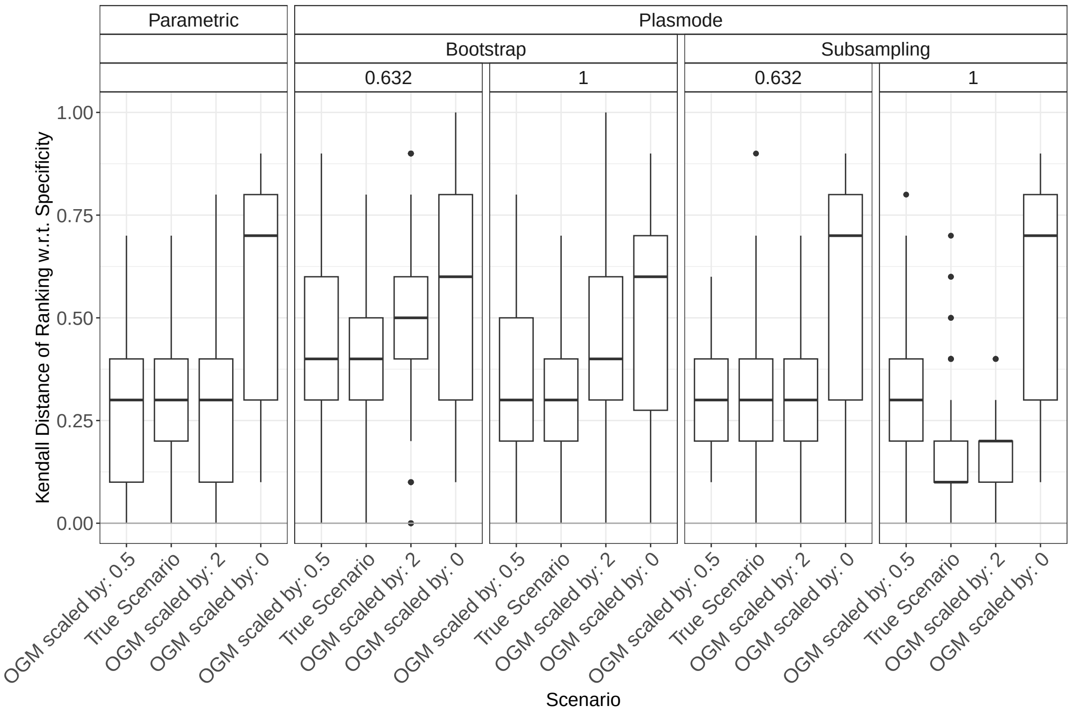

Fig H.64: Kendall distance of the simulated and true method ranking based on specificity in 100 iterations of a classification method comparison study per classifier for different simulation approaches with misspecifications of the OGM for $p = 50$.

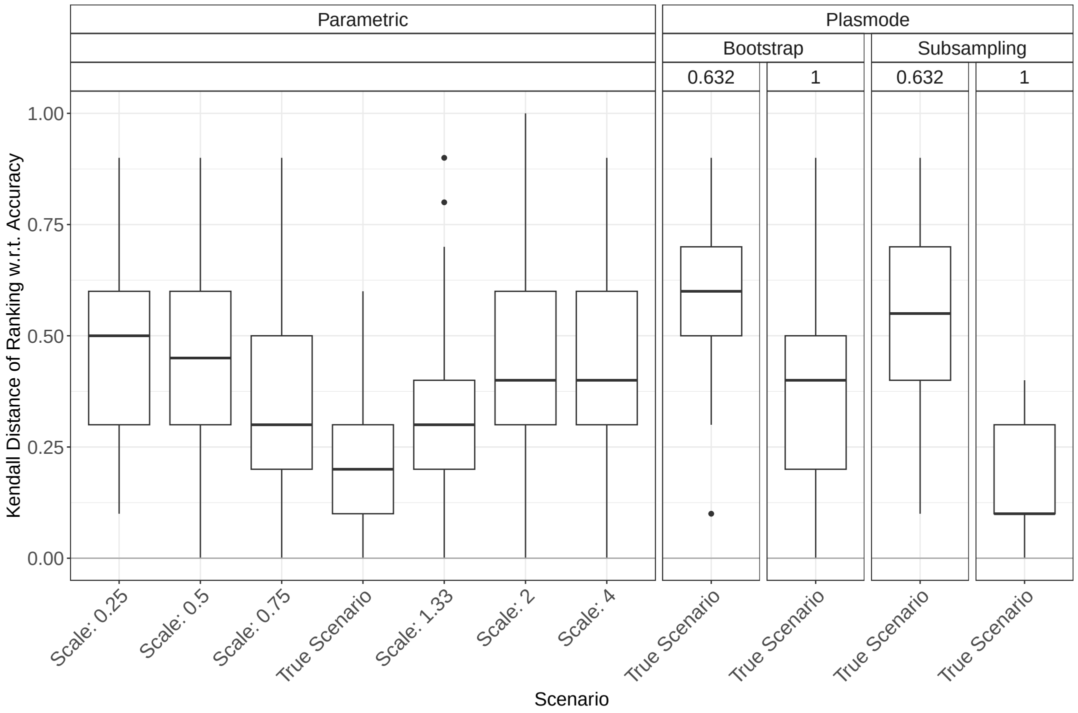

Fig H.65: Kendall distance of the simulated and true method ranking based on accuracy in 100 iterations of a classification method comparison study per classifier for different simulation approaches with misspecifications of the scale for parametric simulation for $p = 50$.

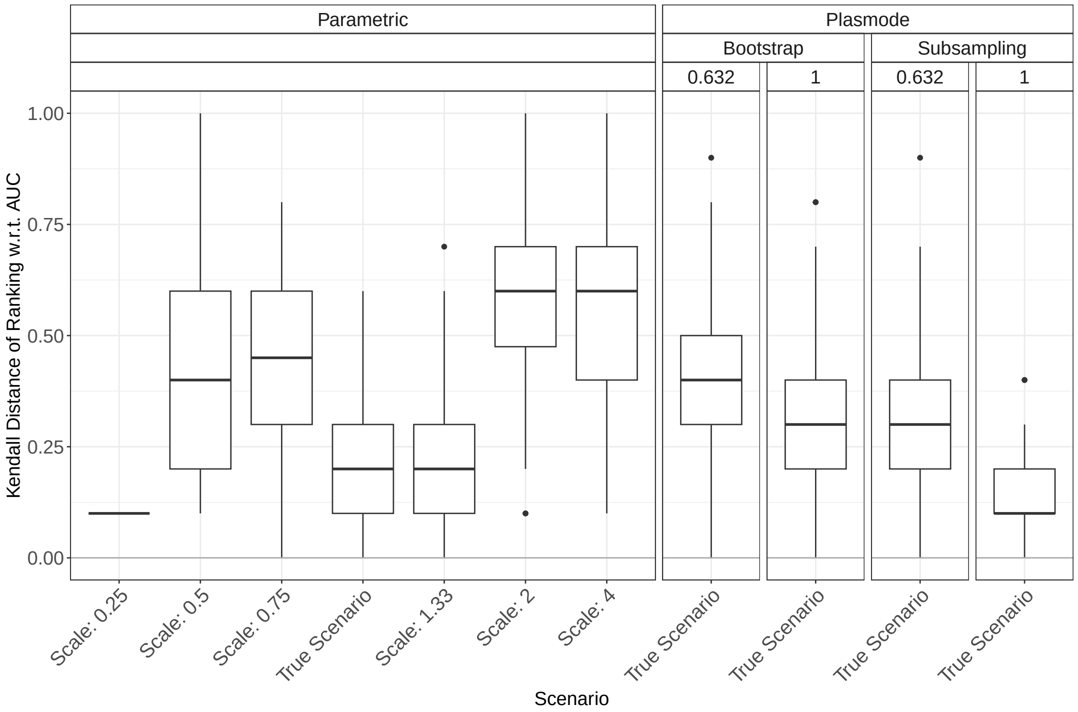

Fig H.66: Kendall distance of the simulated and true method ranking based on AUC in 100 iterations of a classification method comparison study per classifier for different simulation approaches with misspecifications of the scale for parametric simulation for $p = 50$.

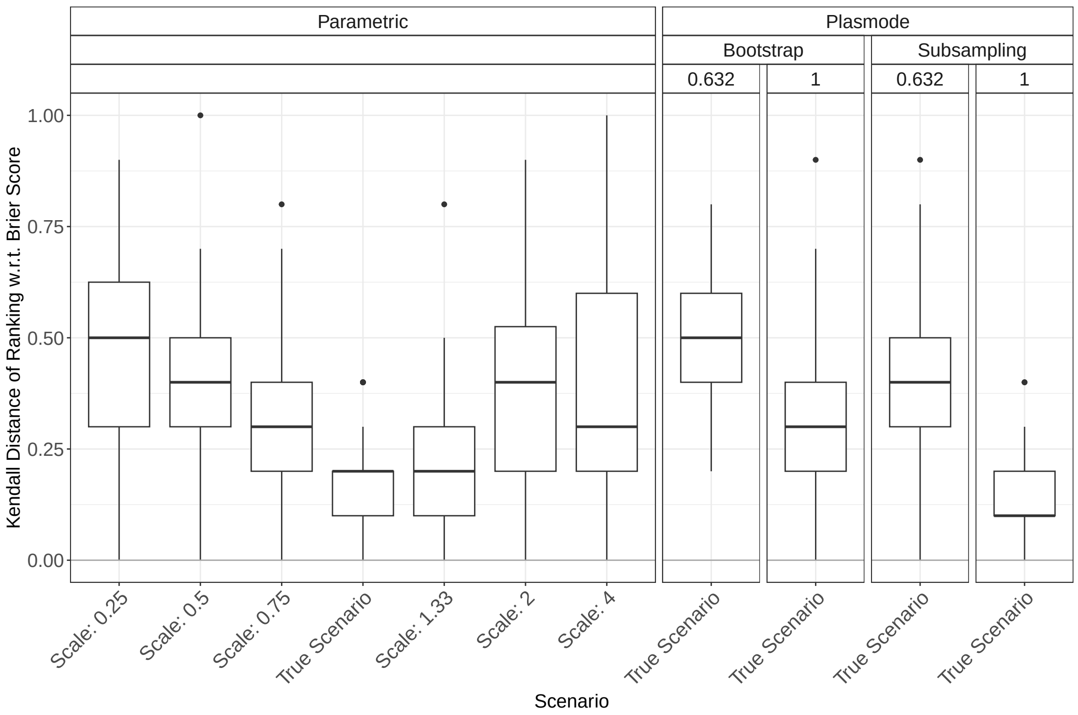

Fig H.67: Kendall distance of the simulated and true method ranking based on the Brier score in 100 iterations of a classification method comparison study per classifier for different simulation approaches with misspecifications of the scale for parametric simulation for $p = 50$.

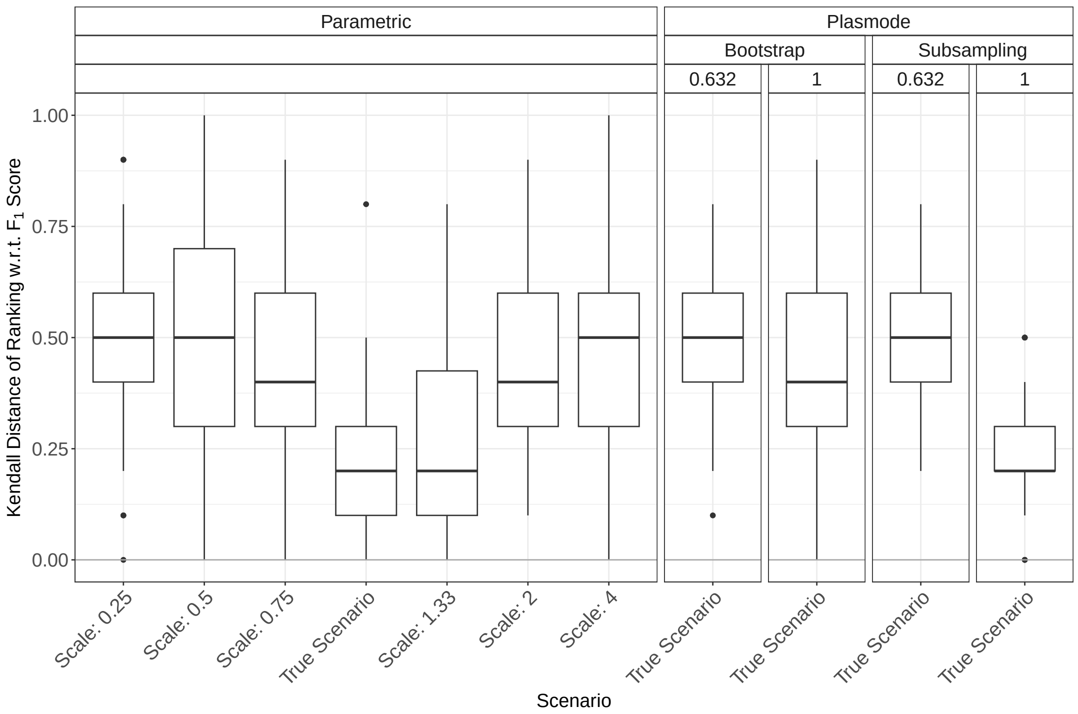

Fig H.68: Kendall distance of the simulated and true method ranking based on F_1-score in 100 iterations of a classification method comparison study per classifier for different simulation approaches with misspecifications of the scale for parametric simulation for $p = 50$.

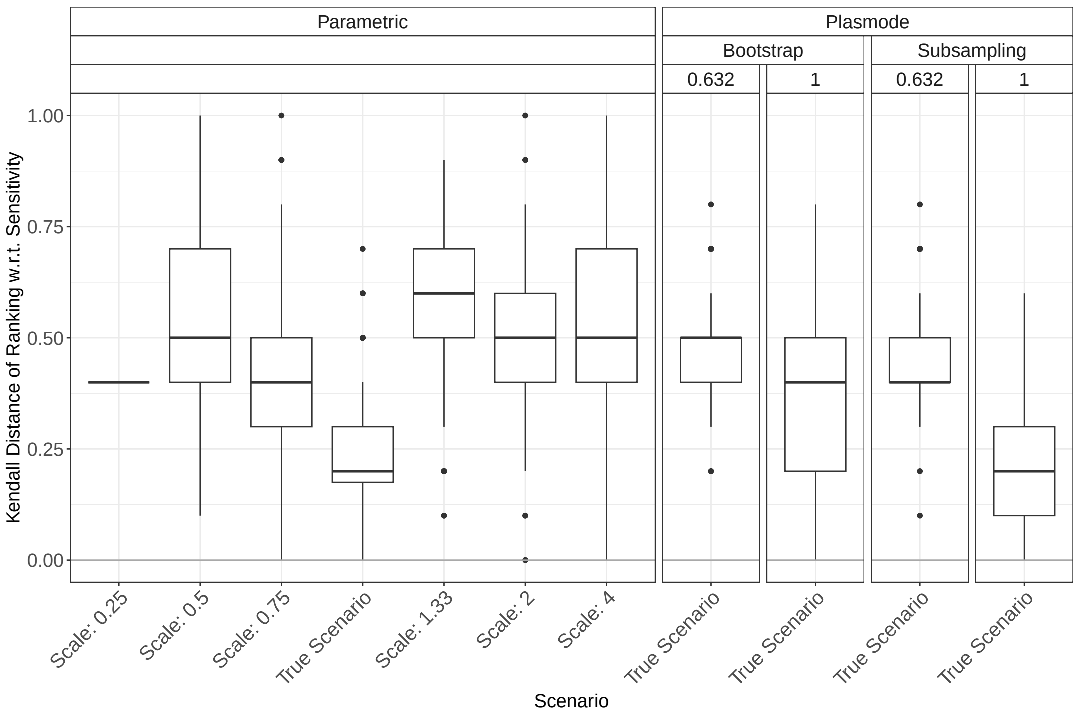

Fig H.69: Kendall distance of the simulated and true method ranking based on sensitivity in 100 iterations of a classification method comparison study per classifier for different simulation approaches with misspecifications of the scale for parametric simulation for $p = 50$.

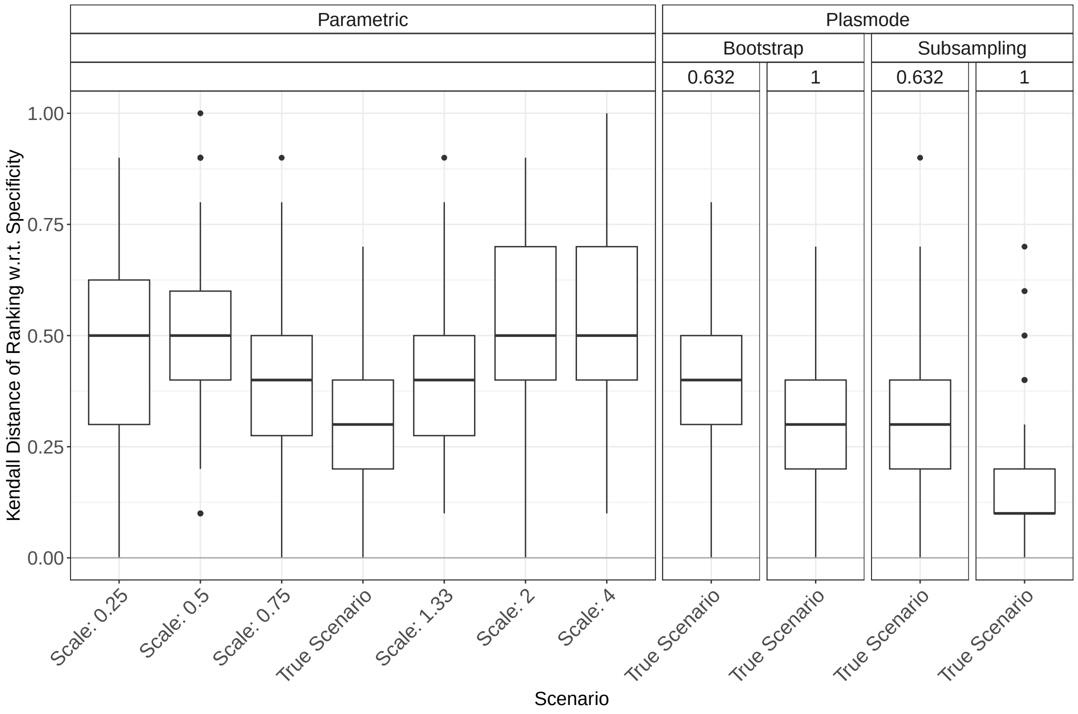

Fig H.70: Kendall distance of the simulated and true method ranking based on specificity in 100 iterations of a classification method comparison study per classifier for different simulation approaches with misspecifications of the scale for parametric simulation for $p = 50$.

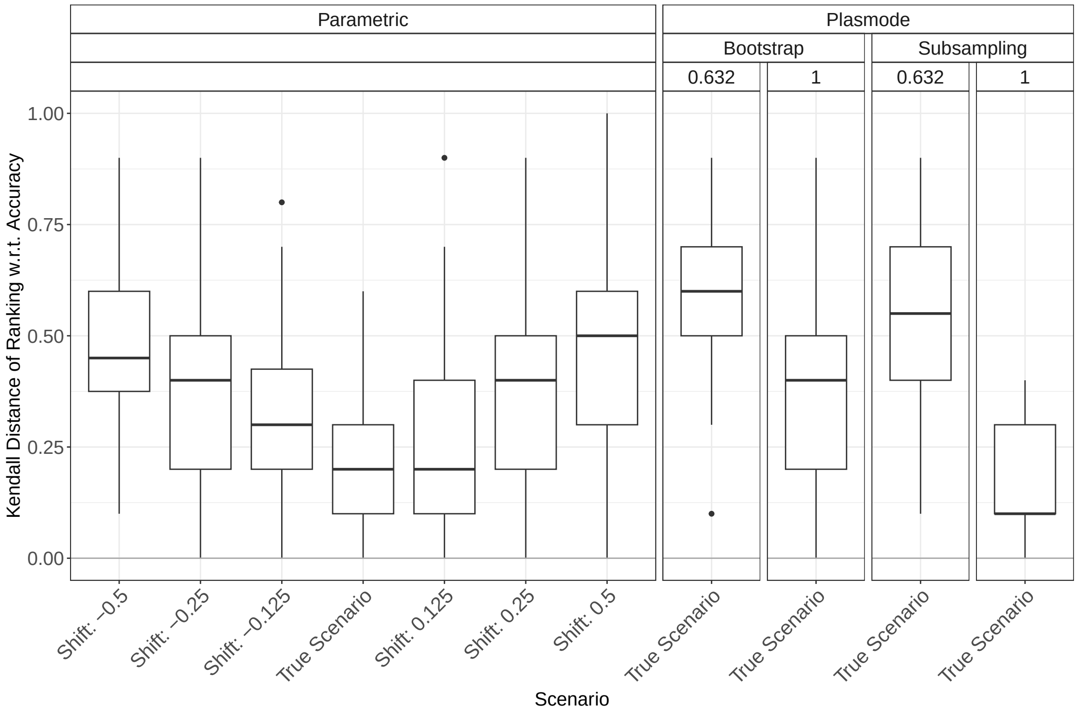

Fig H.71: Kendall distance of the simulated and true method ranking based on accuracy in 100 iterations of a classification method comparison study per classifier for different simulation approaches with misspecifications of the shift for parametric simulation for $p = 50$.

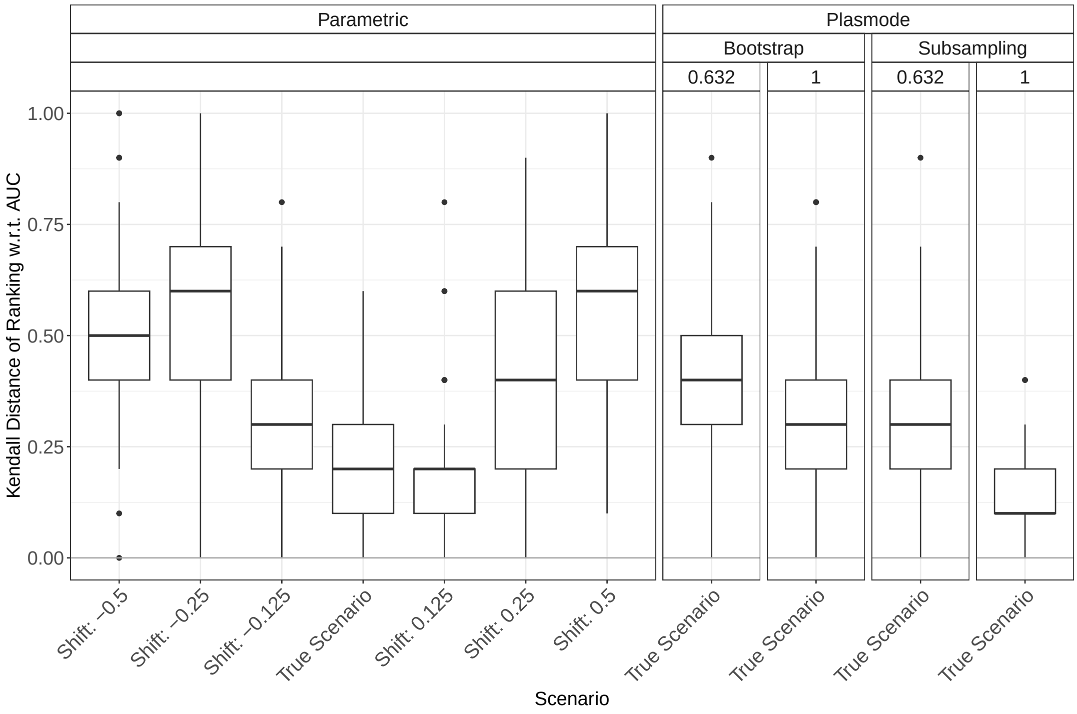

Fig H.72: Kendall distance of the simulated and true method ranking based on AUC in 100 iterations of a classification method comparison study per classifier for different simulation approaches with misspecifications of the shift for parametric simulation for $p = 50$.

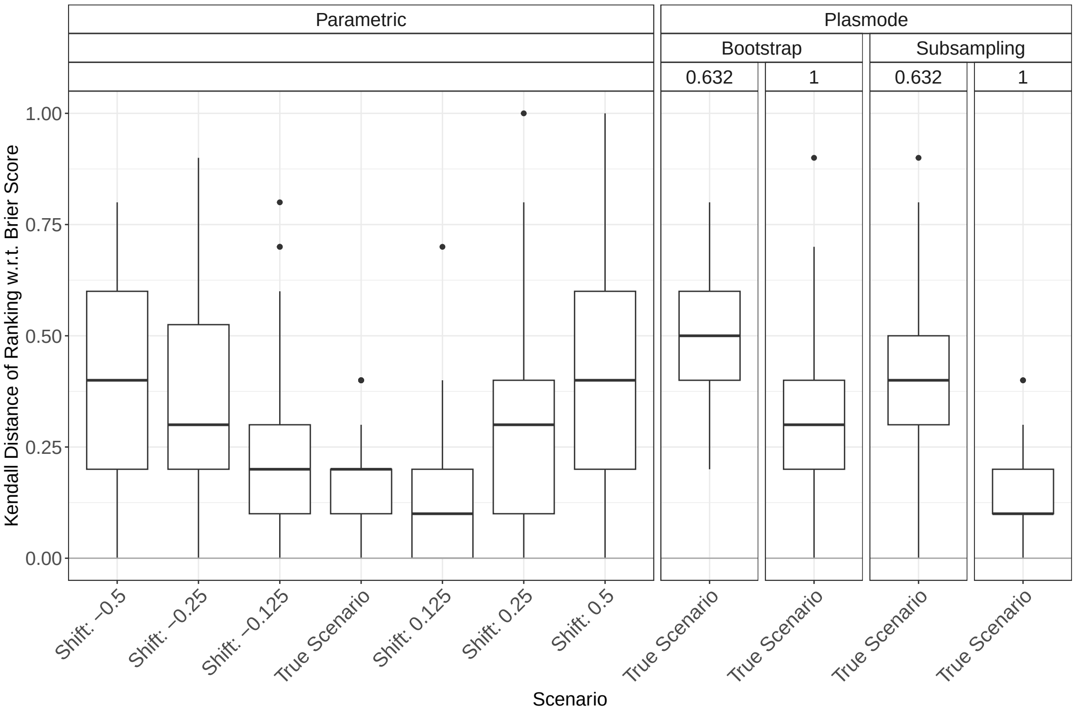

Fig H.73: Kendall distance of the simulated and true method ranking based on the Brier score in 100 iterations of a classification method comparison study per classifier for different simulation approaches with misspecifications of the shift for parametric simulation for $p = 50$.

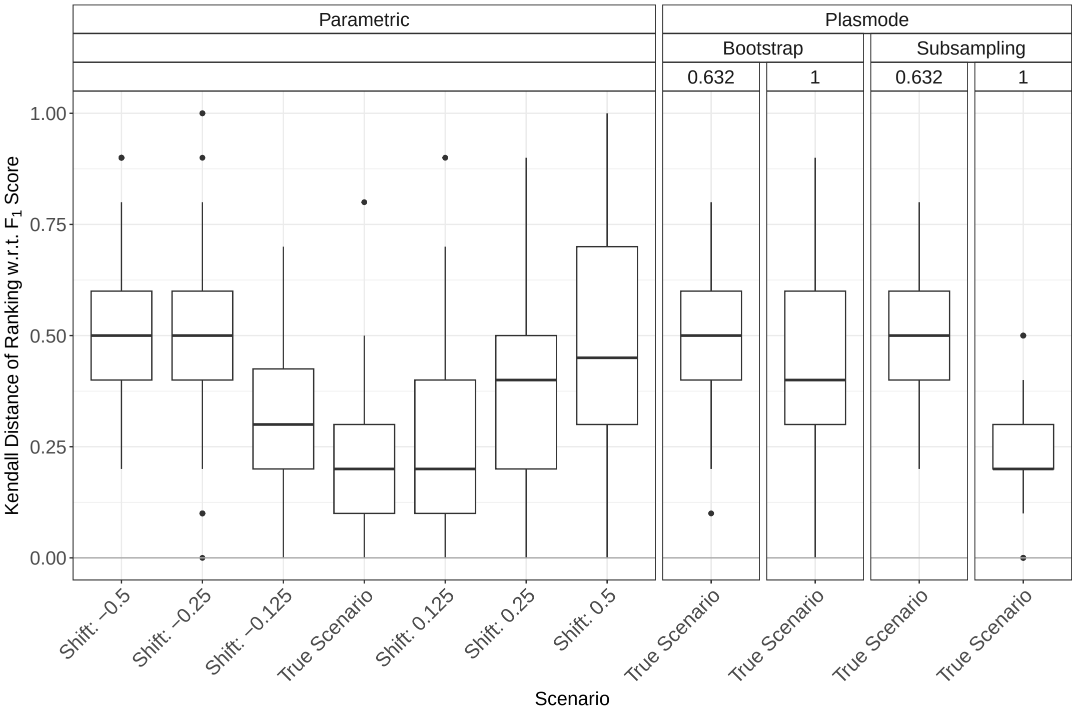

Fig H.74: Kendall distance of the simulated and true method ranking based on F_1-score in 100 iterations of a classification method comparison study per classifier for different simulation approaches with misspecifications of the shift for parametric simulation for $p = 50$.

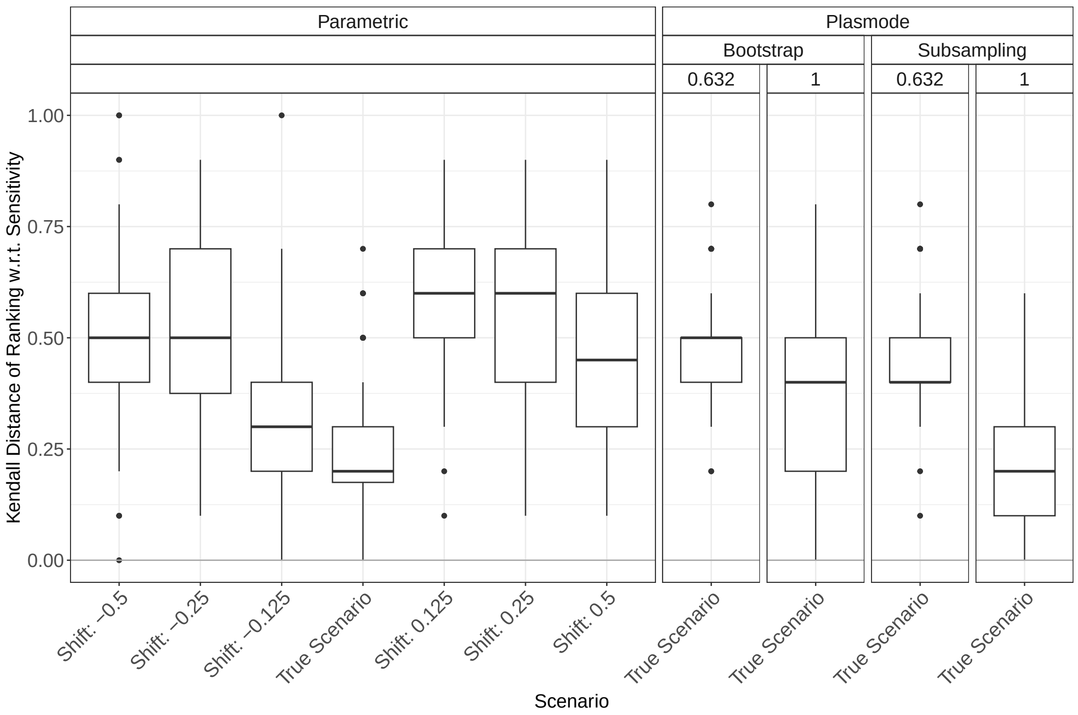

Fig H.75: Kendall distance of the simulated and true method ranking based on sensitivity in 100 iterations of a classification method comparison study per classifier for different simulation approaches with misspecifications of the shift for parametric simulation for $p = 50$.

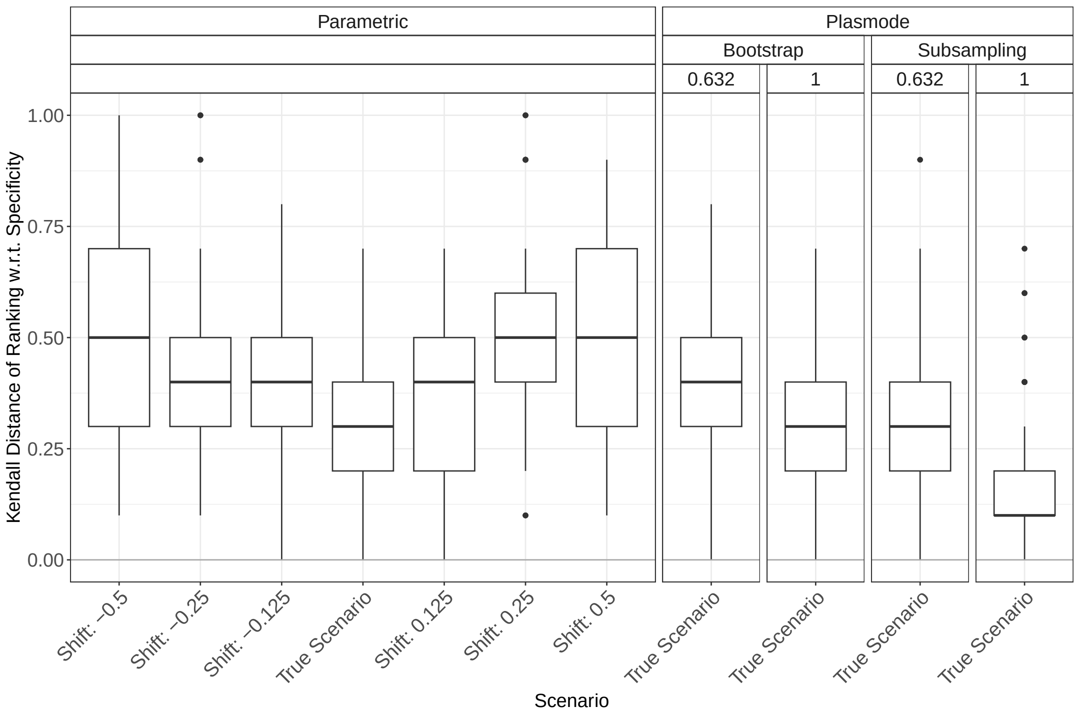

Fig H.76: Kendall distance of the simulated and true method ranking based on specificity in 100 iterations of a classification method comparison study per classifier for different simulation approaches with misspecifications of the shift for parametric simulation for $p = 50$.

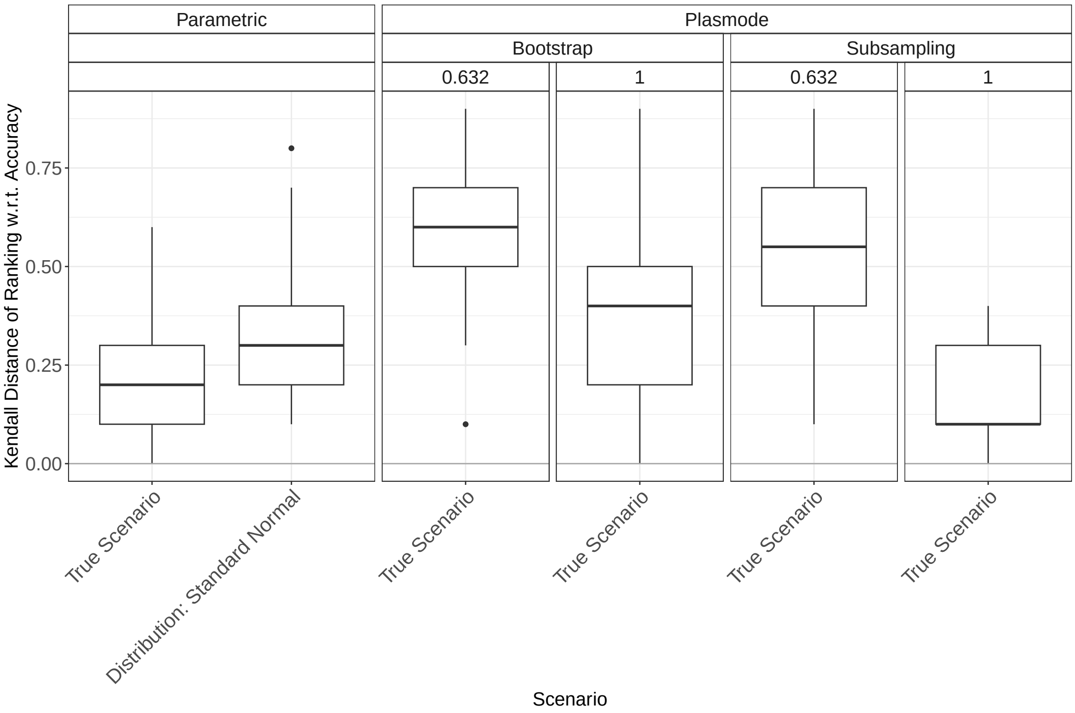

Fig H.77: Kendall distance of the simulated and true method ranking based on accuracy in 100 iterations of a classification method comparison study per classifier for different simulation approaches with misspecification of the distribution as standard normal for parametric simulation for $p = 50$.

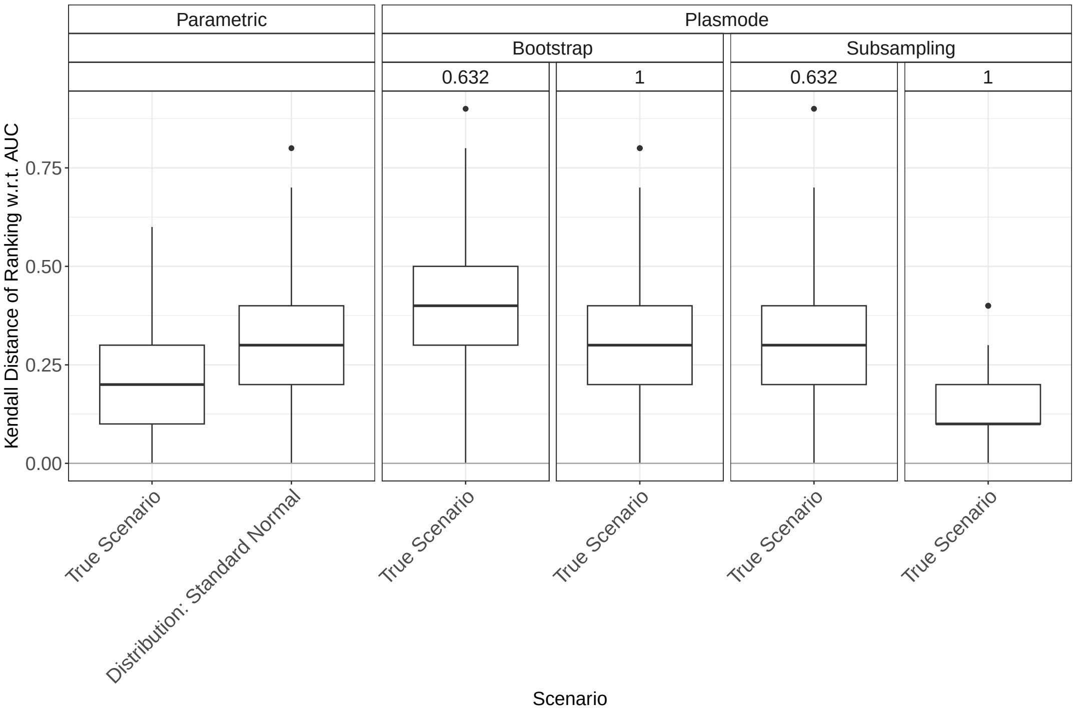

Fig H.78: Kendall distance of the simulated and true method ranking based on AUC in 100 iterations of a classification method comparison study per classifier for different simulation approaches with misspecification of the distribution as standard normal for parametric simulation for $p = 50$.

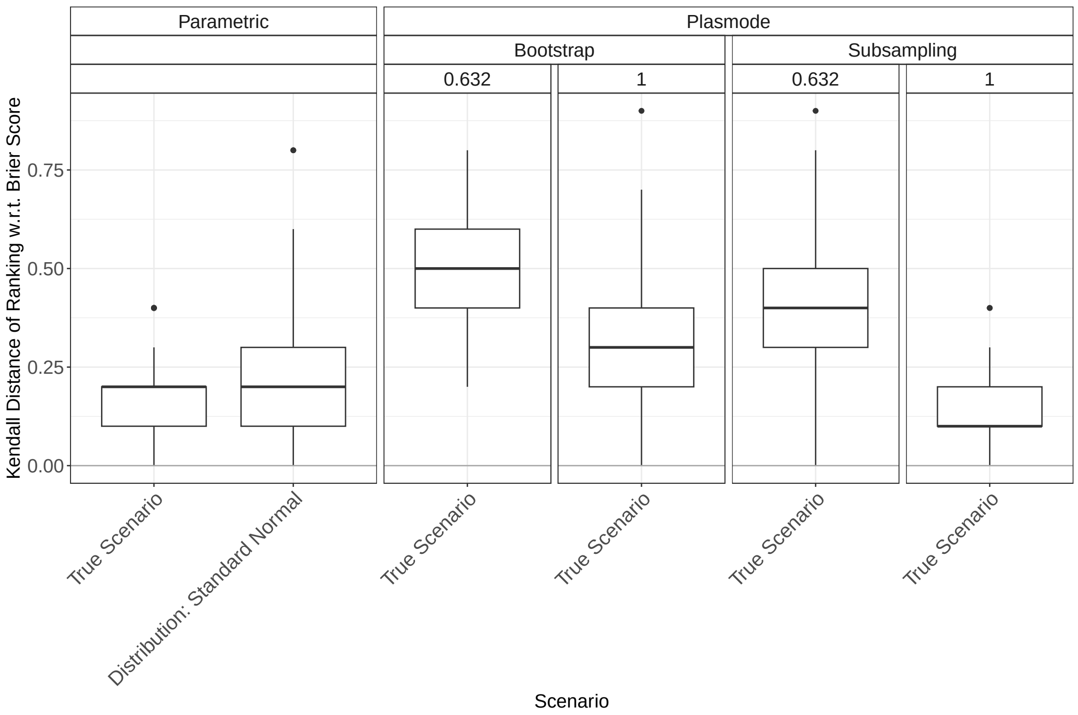

Fig H.79: Kendall distance of the simulated and true method ranking based on the Brier score in 100 iterations of a classification method comparison study per classifier for different simulation approaches with misspecification of the distribution as standard normal for parametric simulation for $p = 50$.

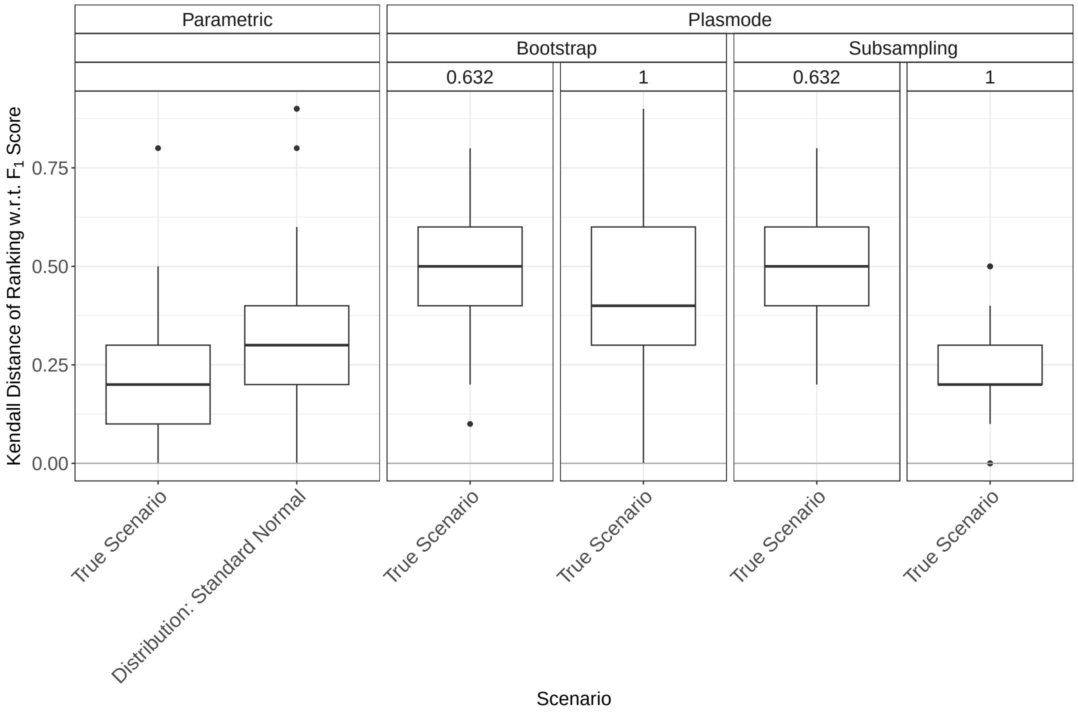

Fig H.80: Kendall distance of the simulated and true method ranking based on F_1-score in 100 iterations of a classification method comparison study per classifier for different simulation approaches with misspecification of the distribution as standard normal for parametric simulation for $p = 50$.

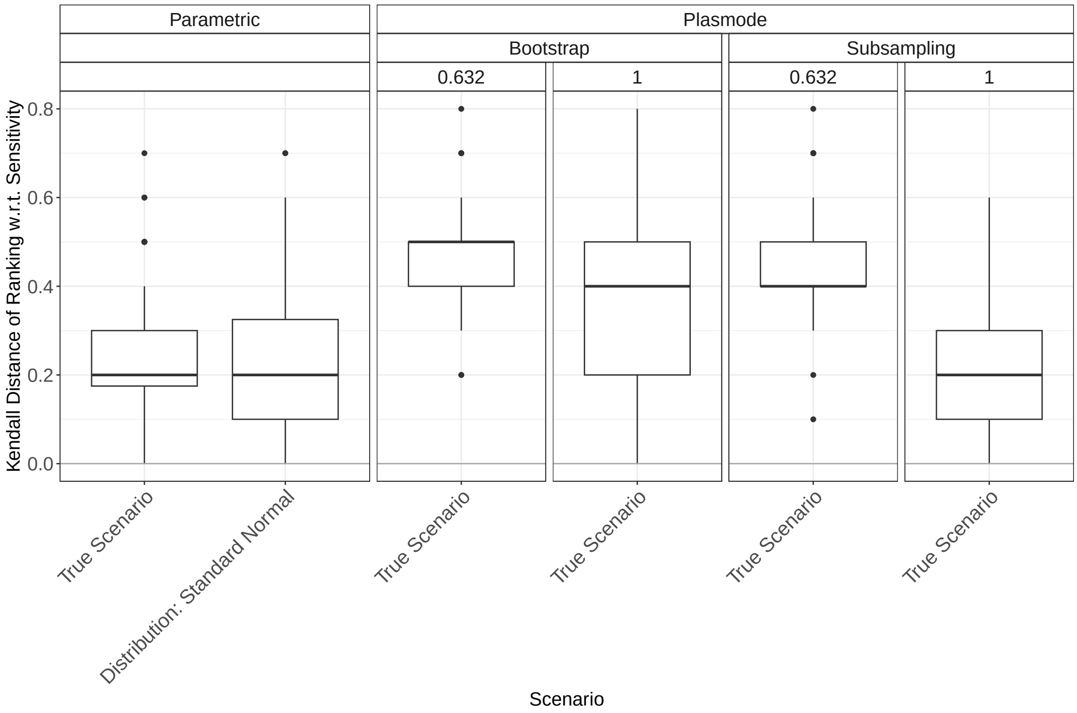

Fig H.81: Kendall distance of the simulated and true method ranking based on sensitivity in 100 iterations of a classification method comparison study per classifier for different simulation approaches with misspecification of the distribution as standard normal for parametric simulation for $p = 50$.

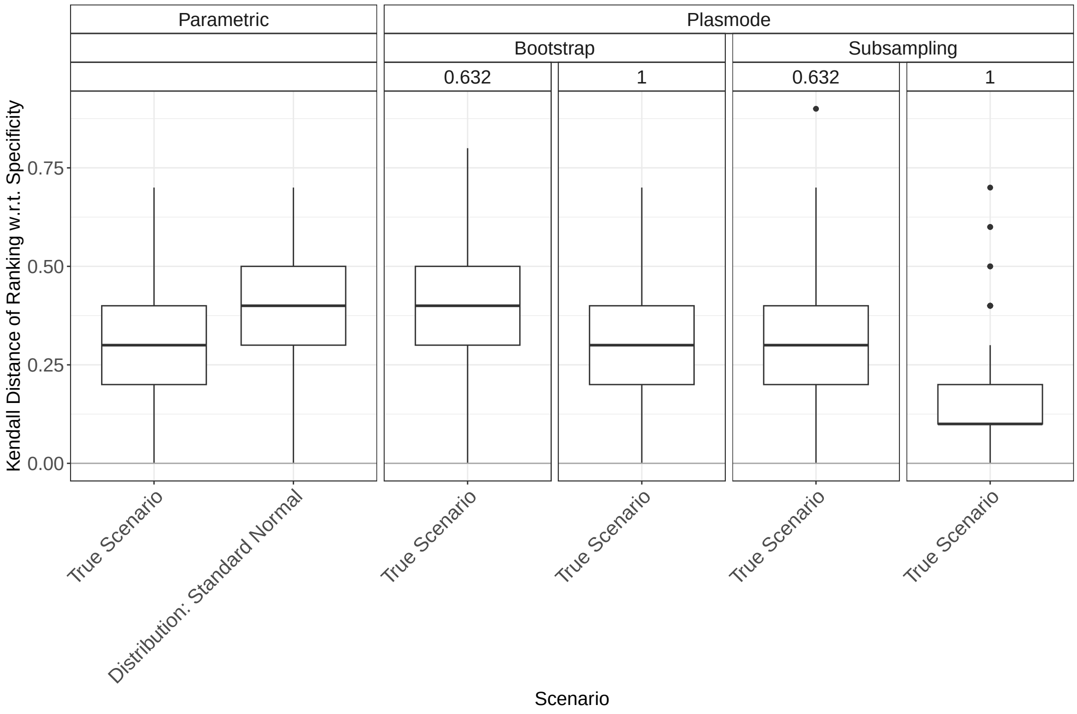

Fig H.82: Kendall distance of the simulated and true method ranking based on specificity in 100 iterations of a classification method comparison study per classifier for different simulation approaches with misspecification of the distribution as standard normal for parametric simulation for $p = 50$.

H.4 $p = 150$

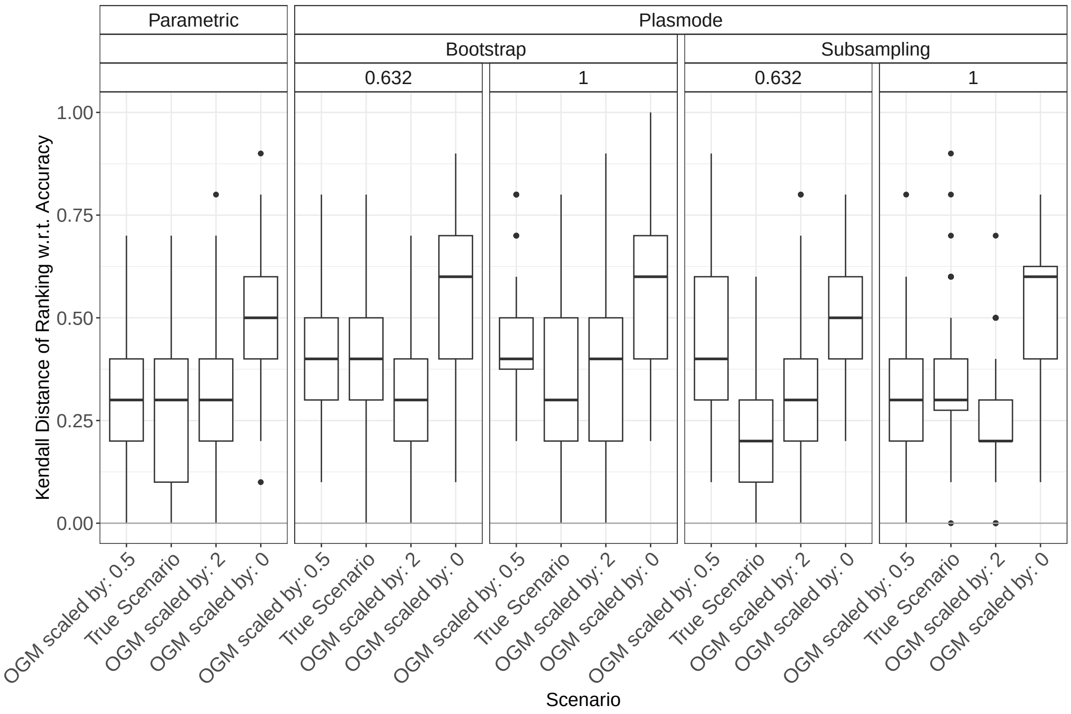

Fig H.83: Kendall distance of the simulated and true method ranking based on accuracy in 100 iterations of a classification method comparison study per classifier for different simulation approaches with misspecifications of the OGM for $p = 150$.

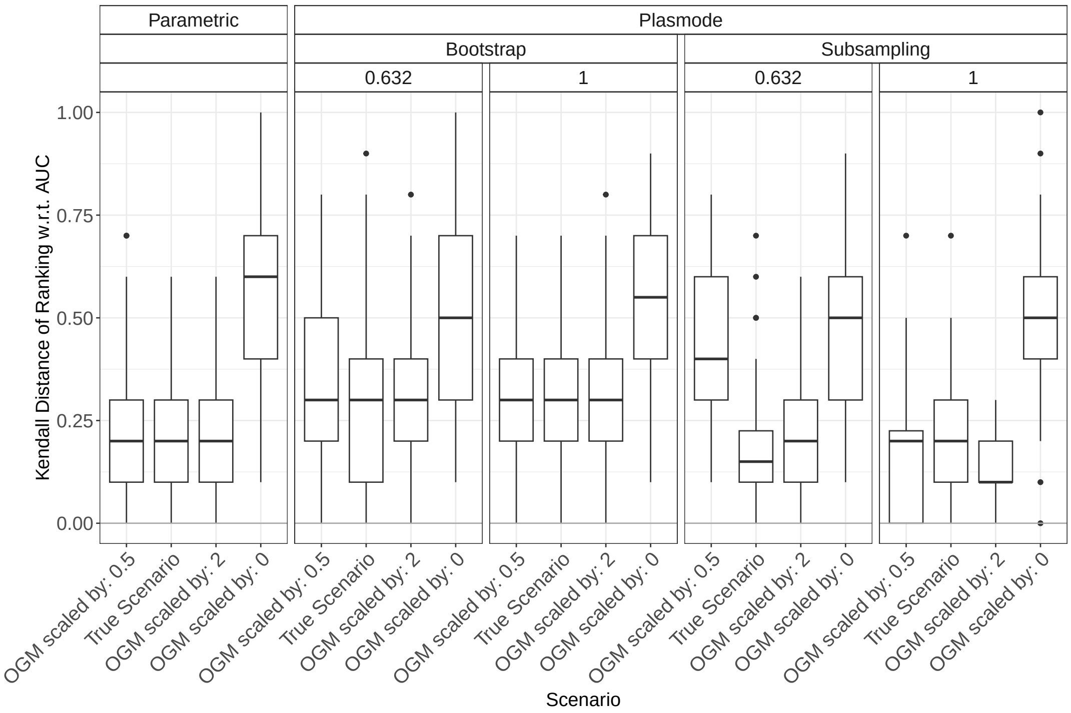

Fig H.84: Kendall distance of the simulated and true method ranking based on AUC in 100 iterations of a classification method comparison study per classifier for different simulation approaches with misspecifications of the OGMfor $p = 150$.

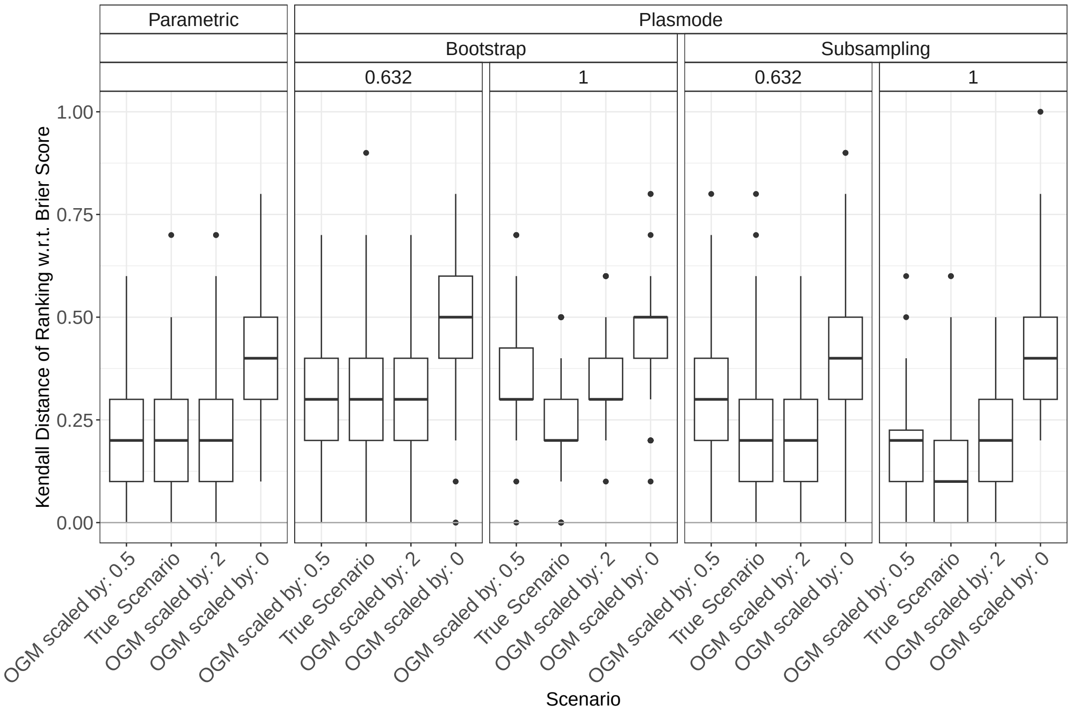

Fig H.85: Kendall distance of the simulated and true method ranking based on the Brier score in 100 iterations of a classification method comparison study per classifier for different simulation approaches with misspecifications of the OGM for $p = 150$.

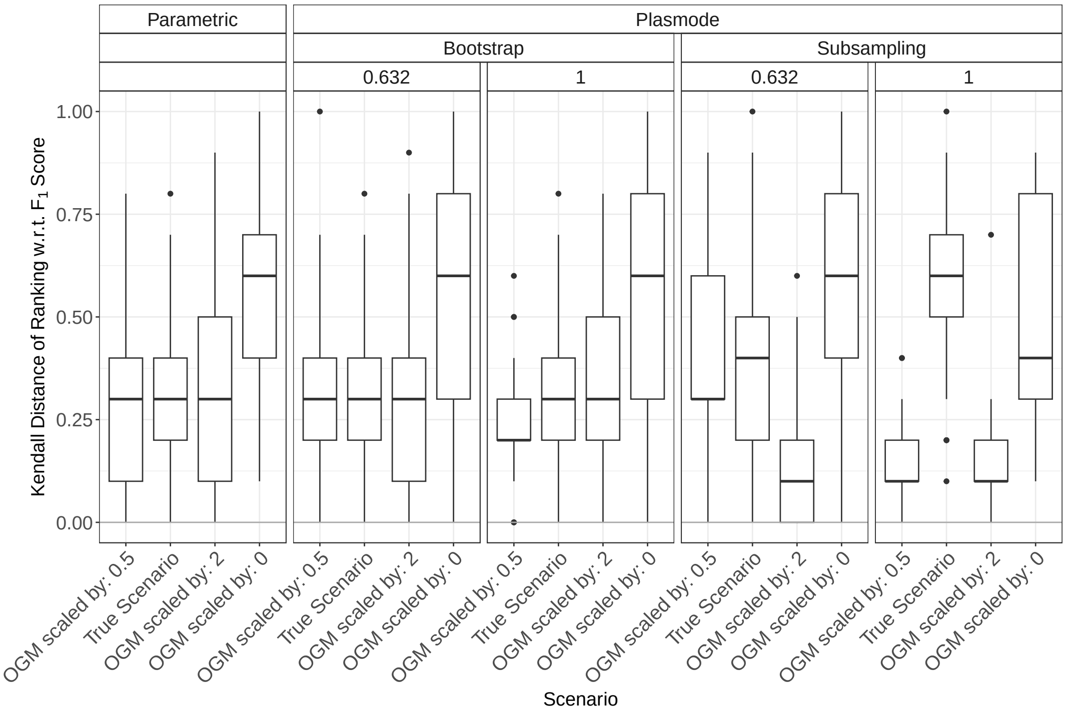

Fig H.86: Kendall distance of the simulated and true method ranking based on F_1-score in 100 iterations of a classification method comparison study per classifier for different simulation approaches with misspecifications of the correlation for parametric simulation for $p = 150$.

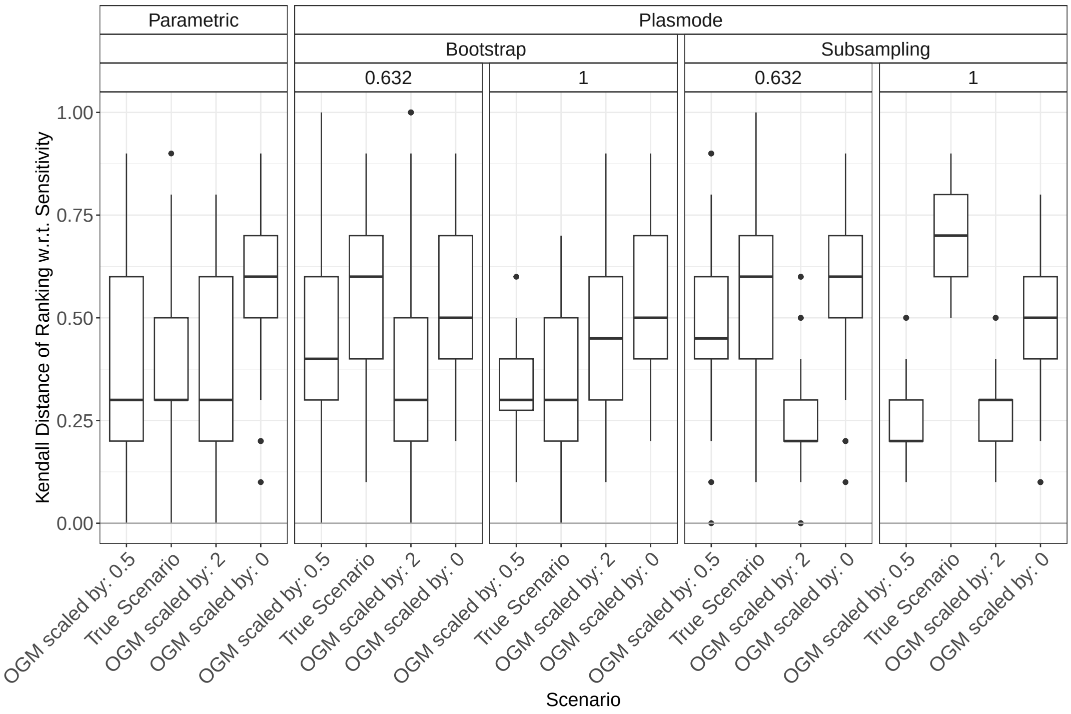

Fig H.87: Kendall distance of the simulated and true method ranking based on sensitivity in 100 iterations of a classification method comparison study per classifier for different simulation approaches with misspecifications of the OGM for $p = 150$.

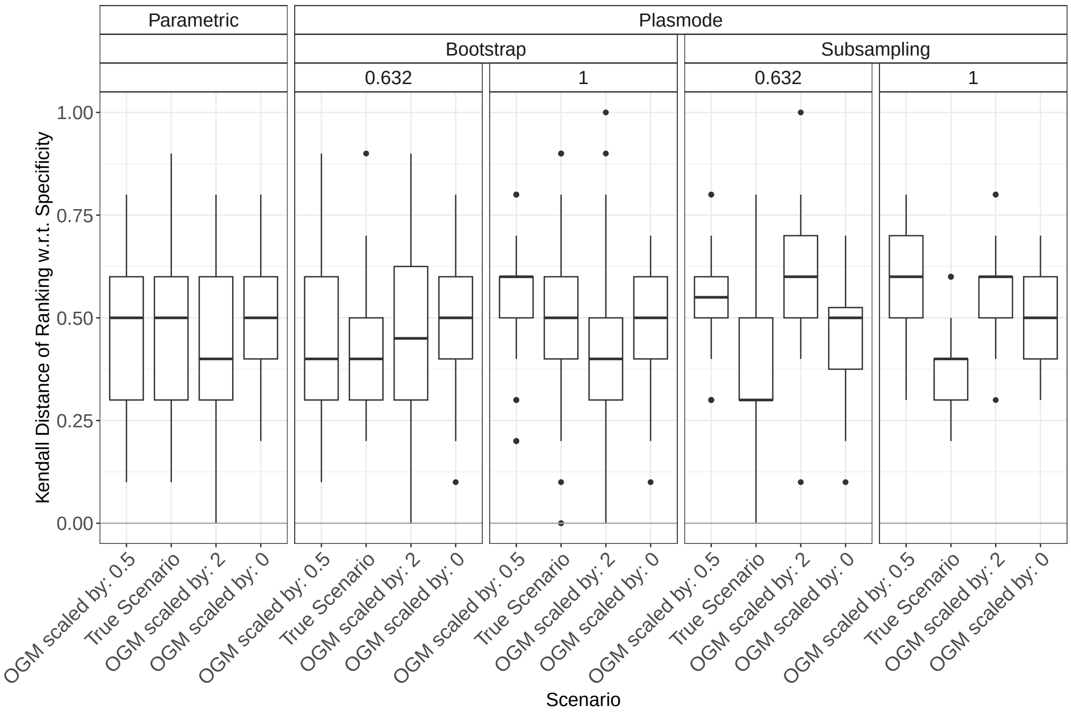

Fig H.88: Kendall distance of the simulated and true method ranking based on specificity in 100 iterations of a classification method comparison study per classifier for different simulation approaches with misspecifications of the OGM for $p = 150$.

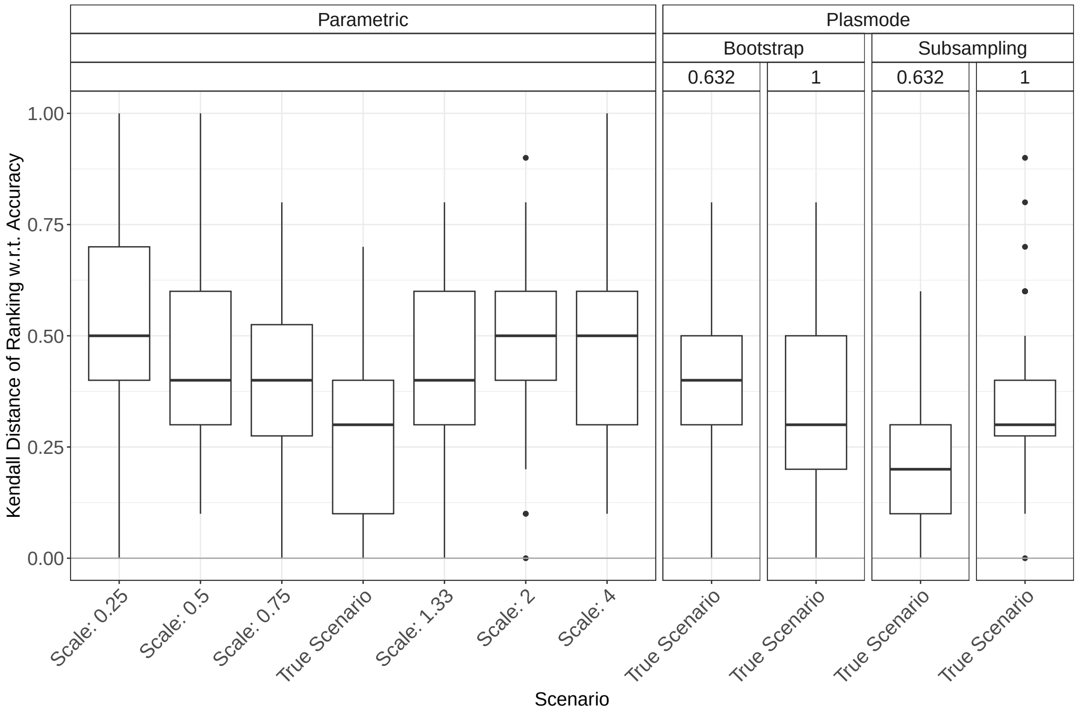

Fig H.89: Kendall distance of the simulated and true method ranking based on accuracy in 100 iterations of a classification method comparison study per classifier for different simulation approaches with misspecifications of the scale for parametric simulation for $p = 150$.

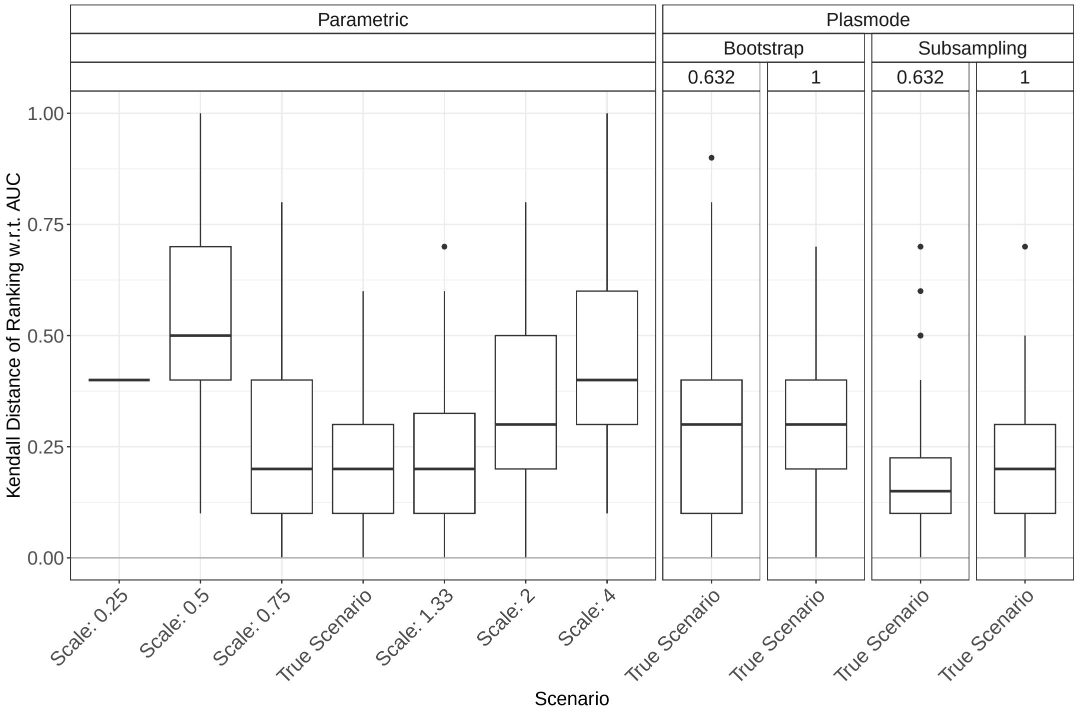

Fig H.90: Kendall distance of the simulated and true method ranking based on AUC in 100 iterations of a classification method comparison study per classifier for different simulation approaches with misspecifications of the scale for parametric simulation for $p = 150$.

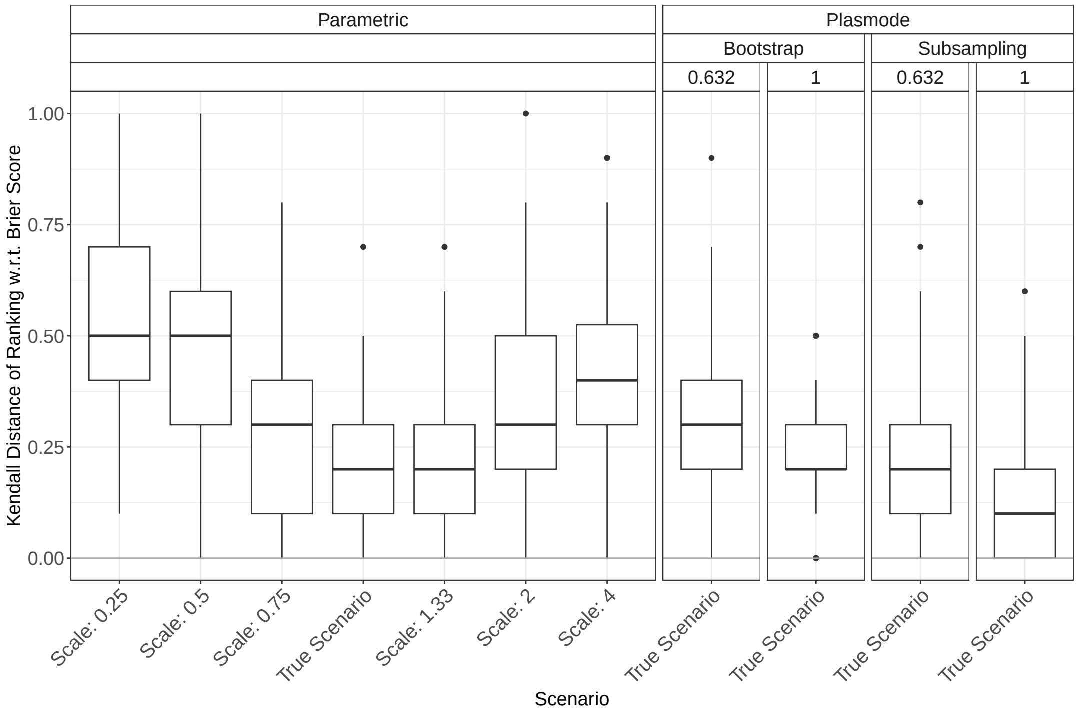

Fig H.91: Kendall distance of the simulated and true method ranking based on the Brier score in 100 iterations of a classification method comparison study per classifier for different simulation approaches with misspecifications of the scale for parametric simulation for $p = 150$.

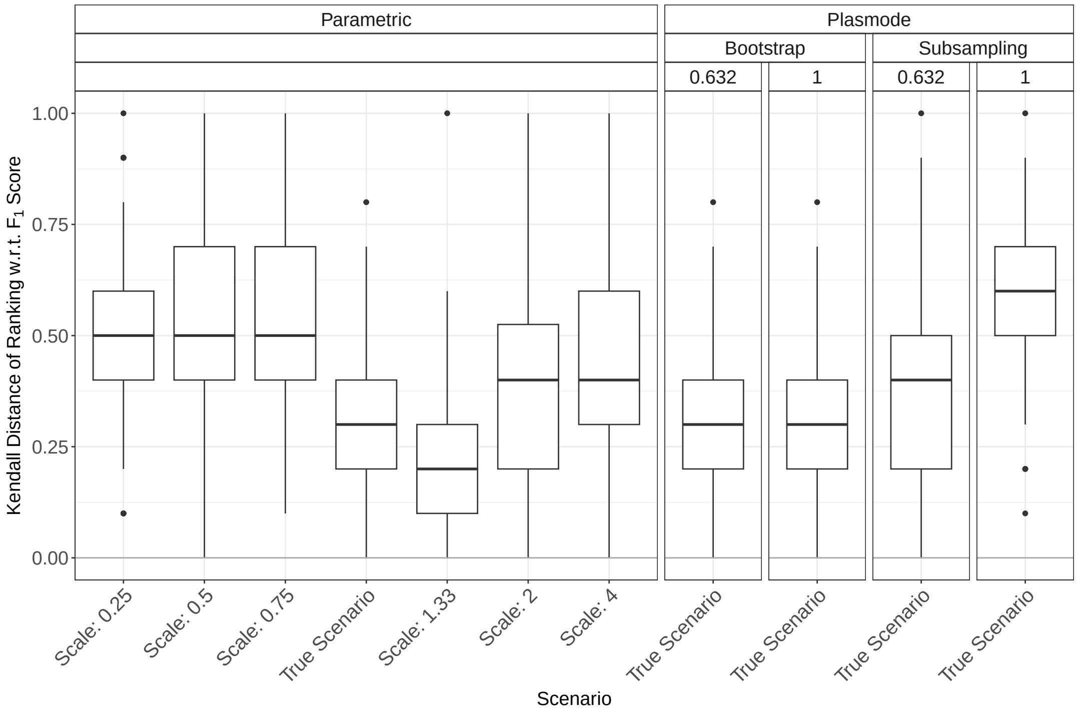

Fig H.92: Kendall distance of the simulated and true method ranking based on F_1-score in 100 iterations of a classification method comparison study per classifier for different simulation approaches with misspecifications of the scale for parametric simulation for $p = 150$.

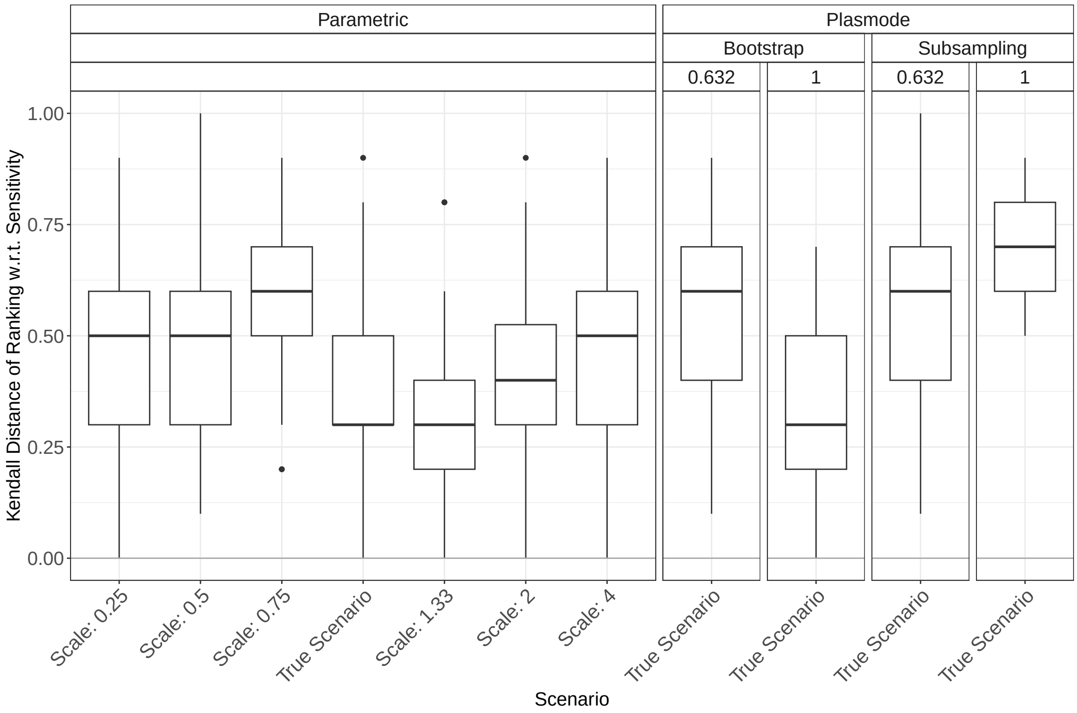

Fig H.93: Kendall distance of the simulated and true method ranking based on sensitivity in 100 iterations of a classification method comparison study per classifier for different simulation approaches with misspecifications of the scale for parametric simulation for $p = 150$.

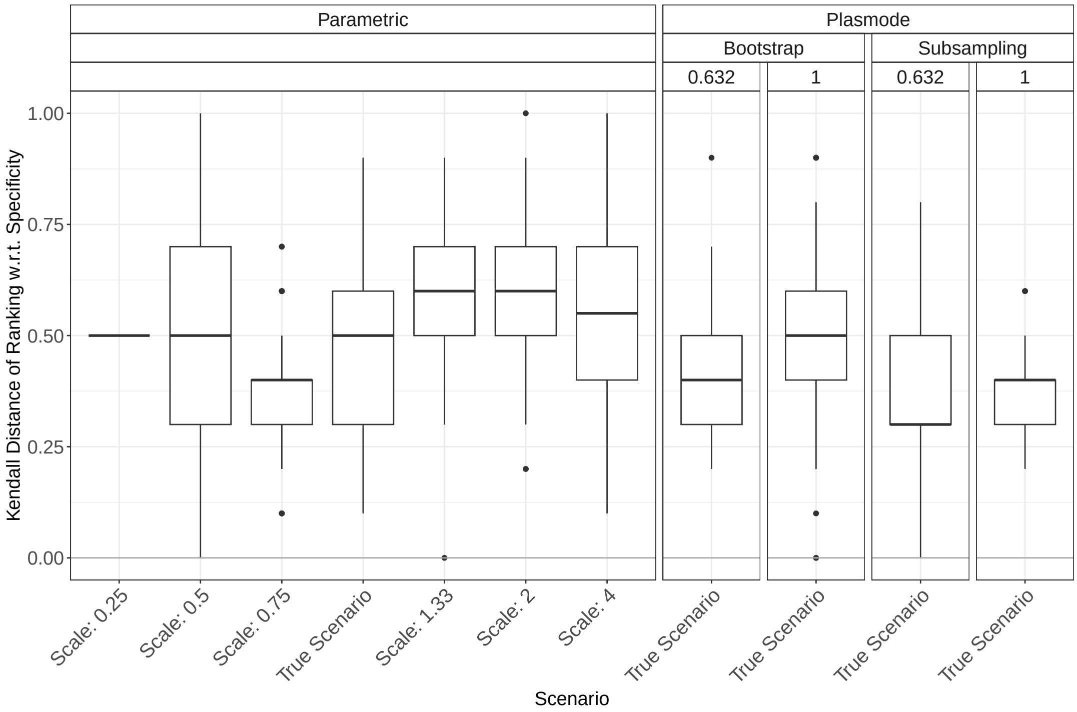

Fig H.94: Kendall distance of the simulated and true method ranking based on specificity in 100 iterations of a classification method comparison study per classifier for different simulation approaches with misspecifications of the scale for parametric simulation for $p = 150$.

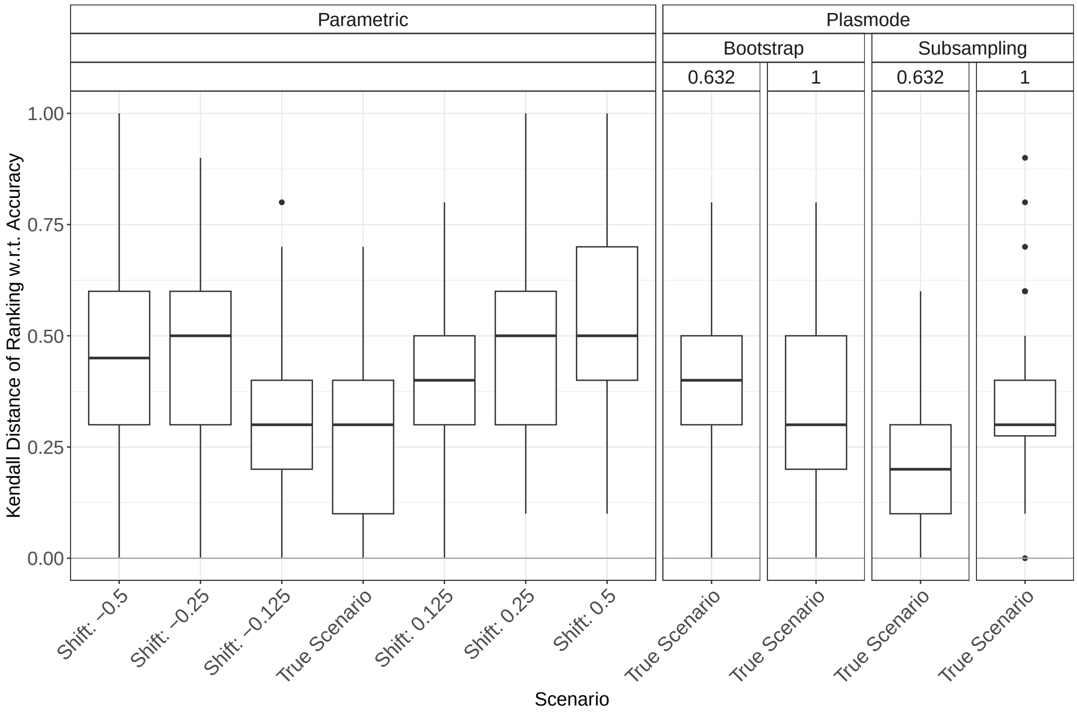

Fig H.95: Kendall distance of the simulated and true method ranking based on accuracy in 100 iterations of a classification method comparison study per classifier for different simulation approaches with misspecifications of the shift for parametric simulation for $p = 150$.

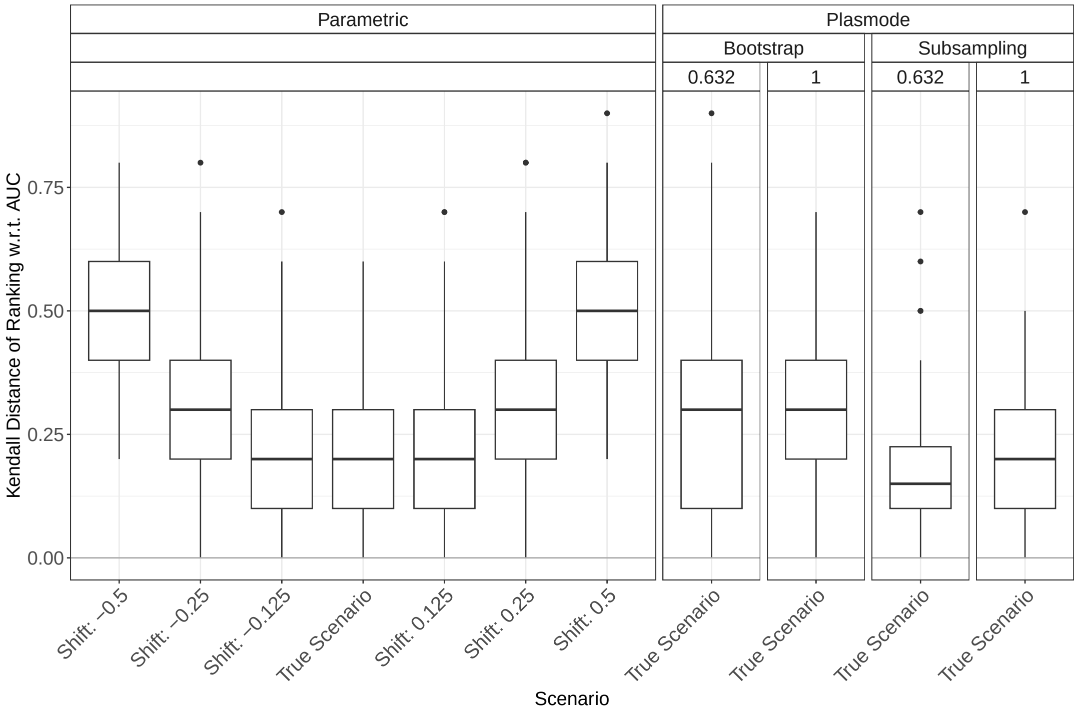

Fig H.96: Kendall distance of the simulated and true method ranking based on AUC in 100 iterations of a classification method comparison study per classifier for different simulation approaches with misspecifications of the shift for parametric simulation for $p = 150$.

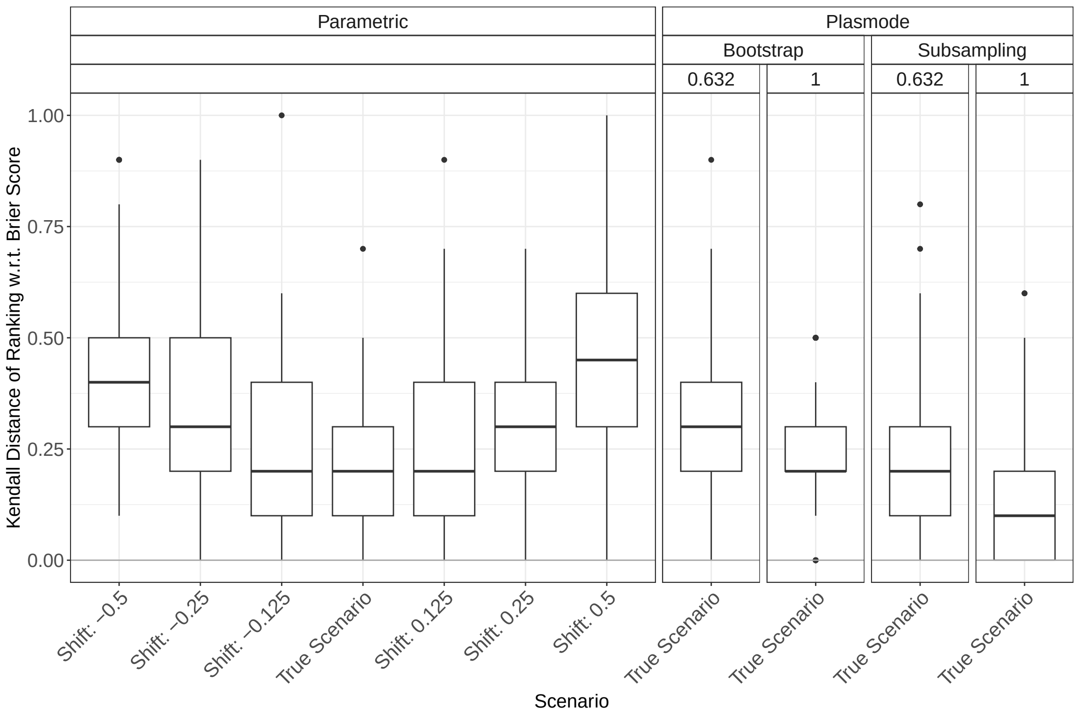

Fig H.97: Kendall distance of the simulated and true method ranking based on the Brier score in 100 iterations of a classification method comparison study per classifier for different simulation approaches with misspecifications of the shift for parametric simulation for $p = 150$.

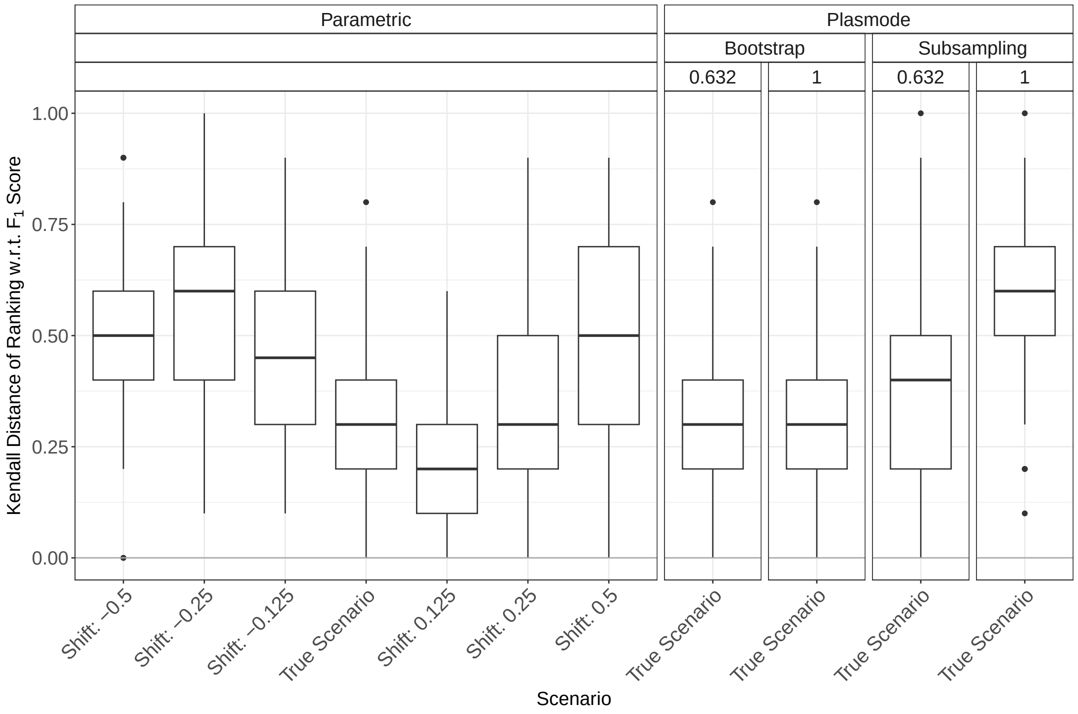

Fig H.98: Kendall distance of the simulated and true method ranking based on F_1-score in 100 iterations of a classification method comparison study per classifier for different simulation approaches with misspecifications of the shift for parametric simulation for $p = 150$.

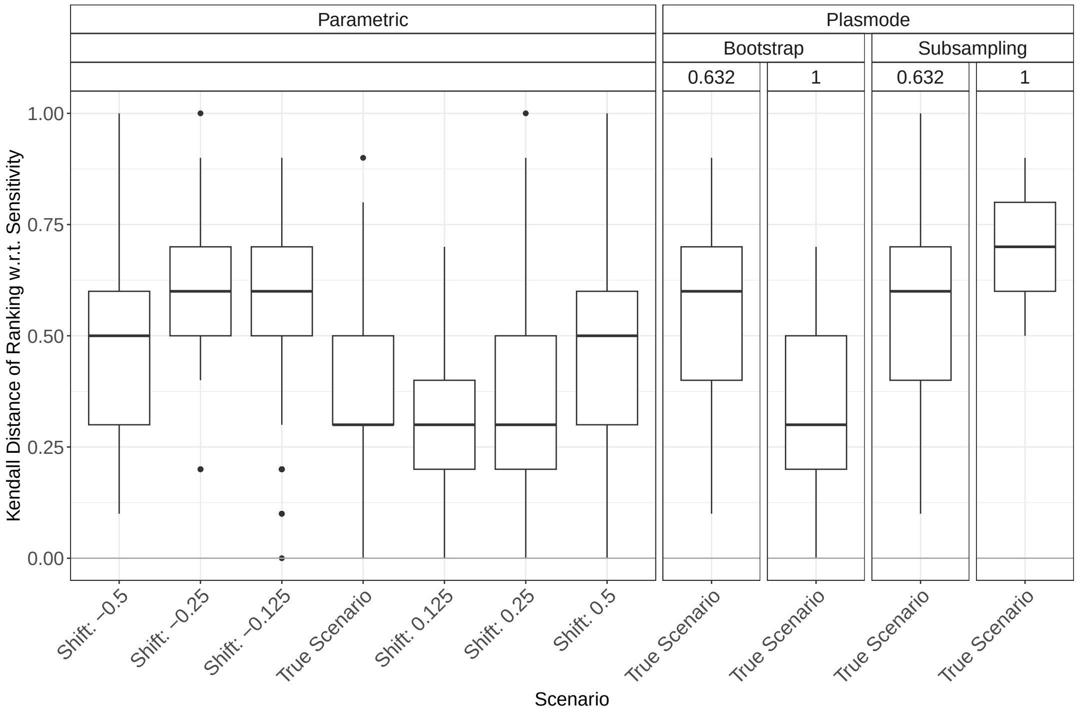

Fig H.99: Kendall distance of the simulated and true method ranking based on sensitivity in 100 iterations of a classification method comparison study per classifier for different simulation approaches with misspecifications of the shift for parametric simulation for $p = 150$.

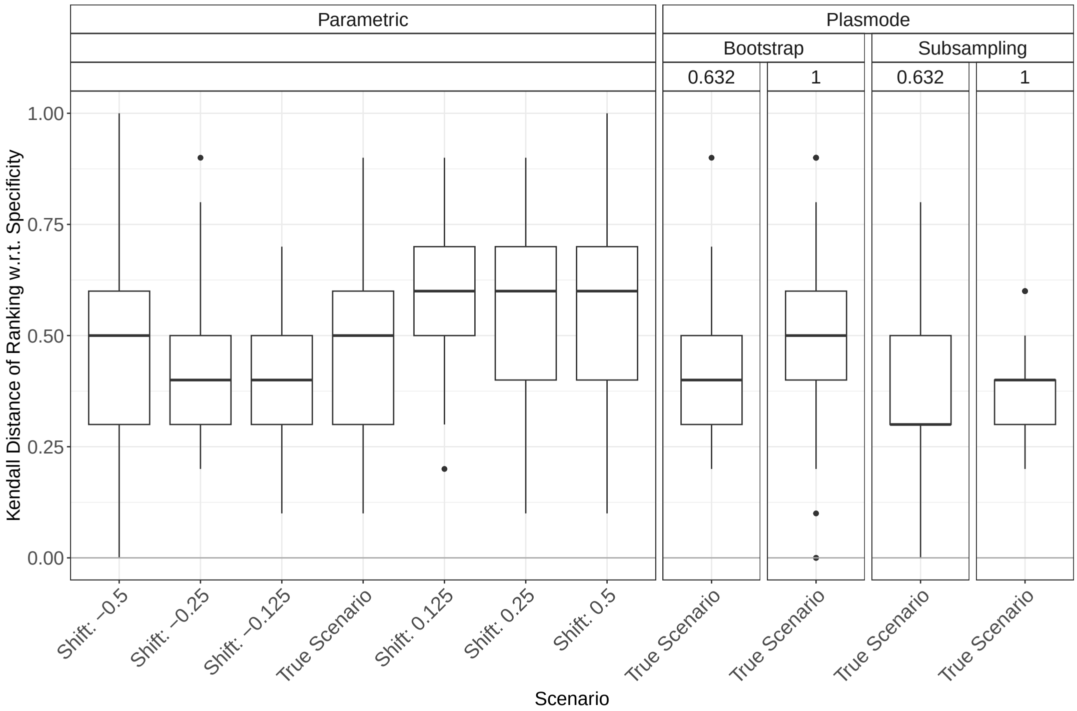

Fig H.100: Kendall distance of the simulated and true method ranking based on specificity in 100 iterations of a classification method comparison study per classifier for different simulation approaches with misspecifications of the shift for parametric simulation for $p = 150$.

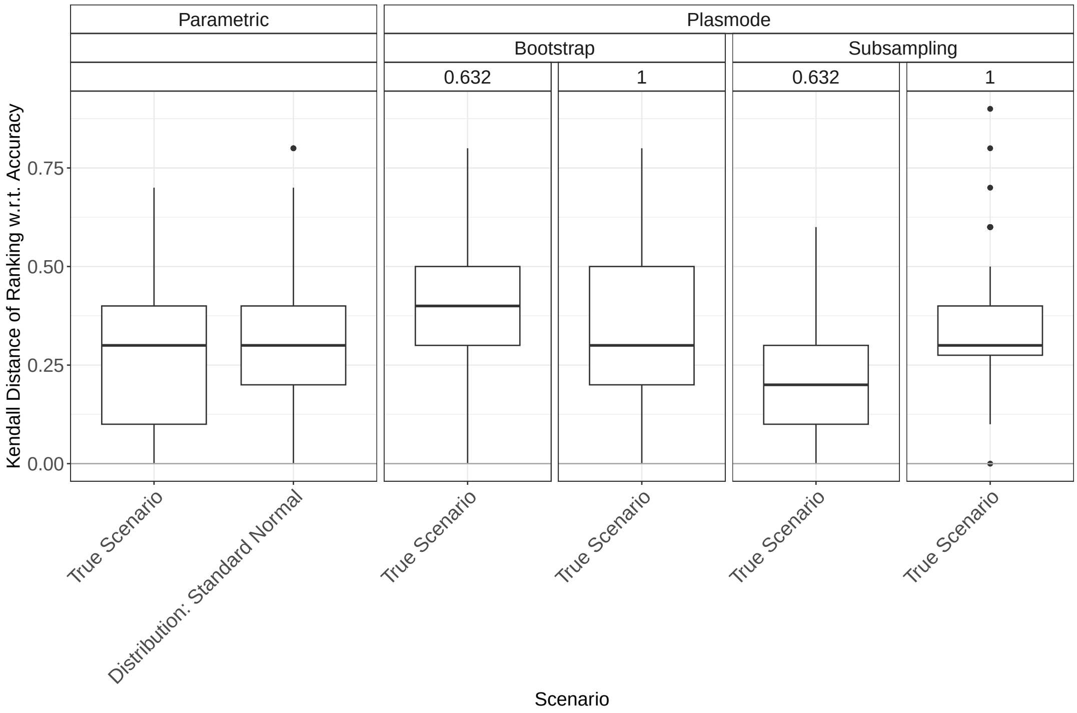

Fig H.101: Kendall distance of the simulated and true method ranking based on accuracy in 100 iterations of a classification method comparison study per classifier for different simulation approaches with misspecification of the distribution as standard normal for parametric simulation for $p = 150$.

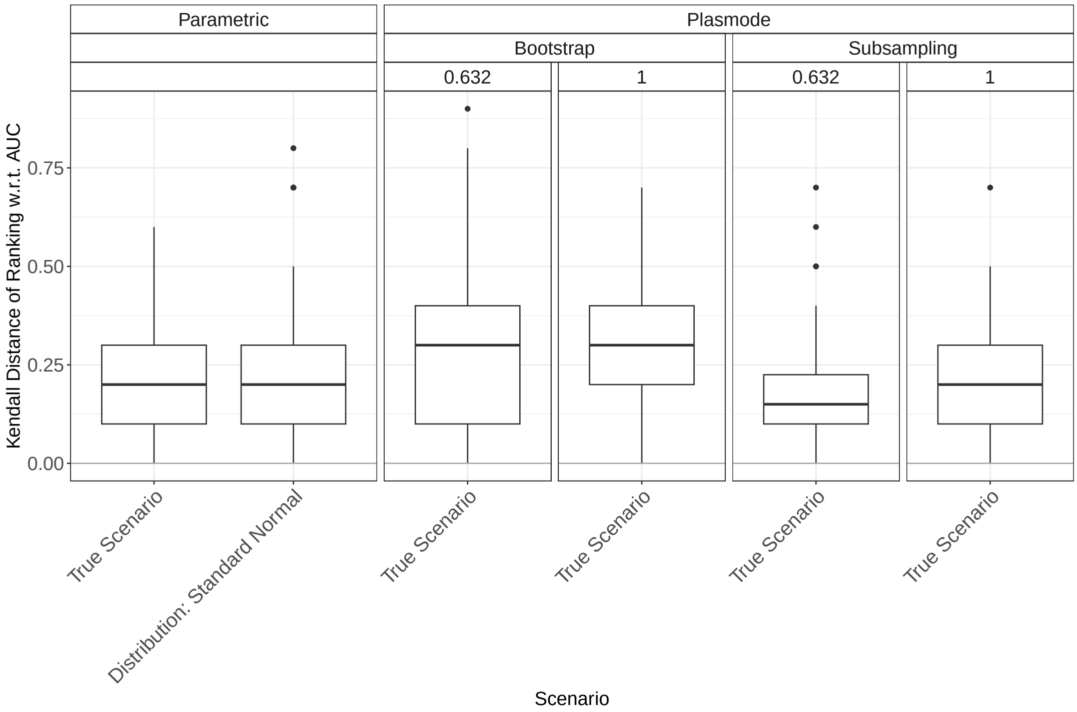

Fig H.102: Kendall distance of the simulated and true method ranking based on AUC in 100 iterations of a classification method comparison study per classifier for different simulation approaches with misspecification of the distribution as standard normal for parametric simulation for $p = 150$.

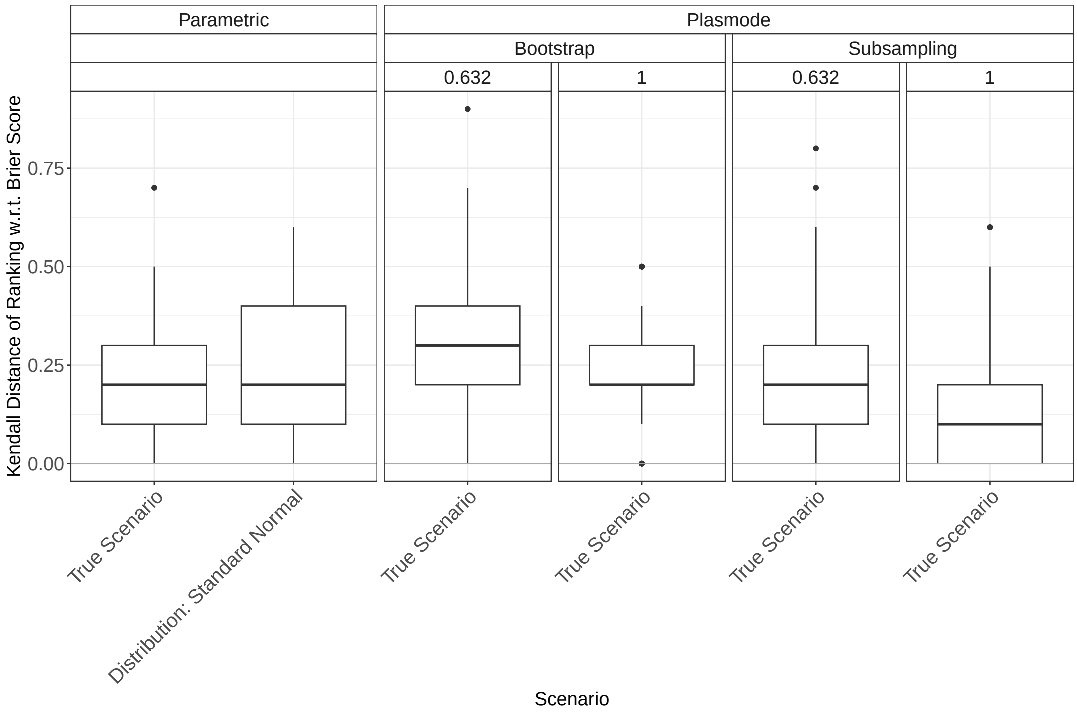

Fig H.103: Kendall distance of the simulated and true method ranking based on the Brier score in 100 iterations of a classification method comparison study per classifier for different simulation approaches with misspecification of the distribution as standard normal for parametric simulation for $p = 150$.

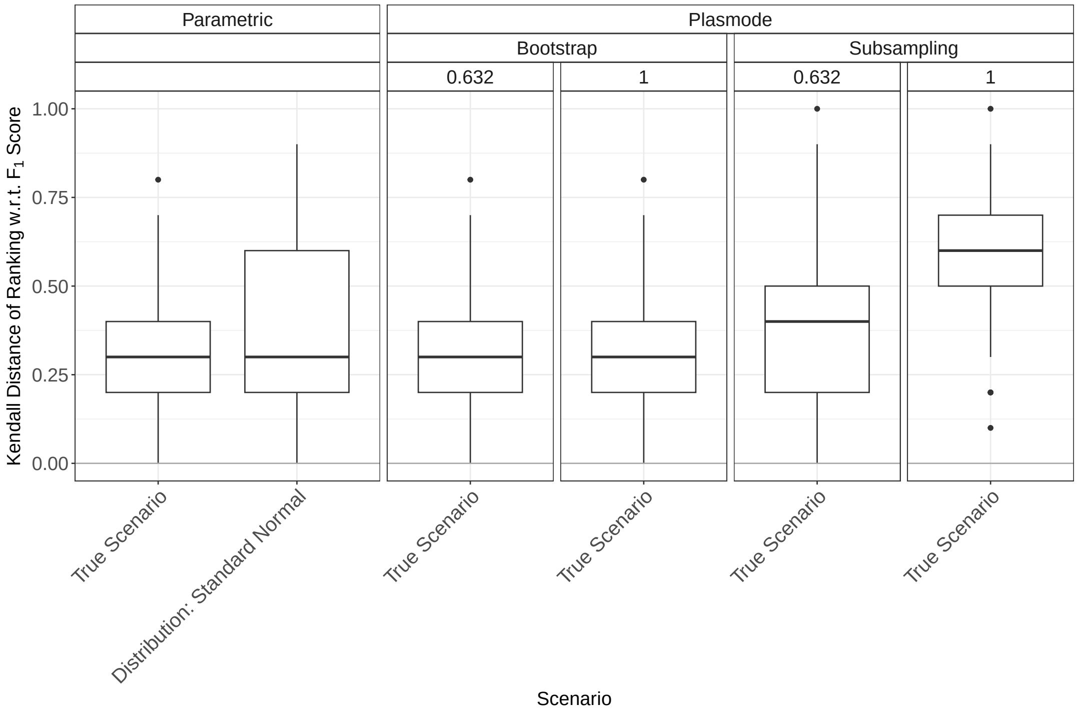

Fig H.104: Kendall distance of the simulated and true method ranking based on F_1-score in 100 iterations of a classification method comparison study per classifier for different simulation approaches with misspecification of the distribution as standard normal for parametric simulation for $p = 150$.

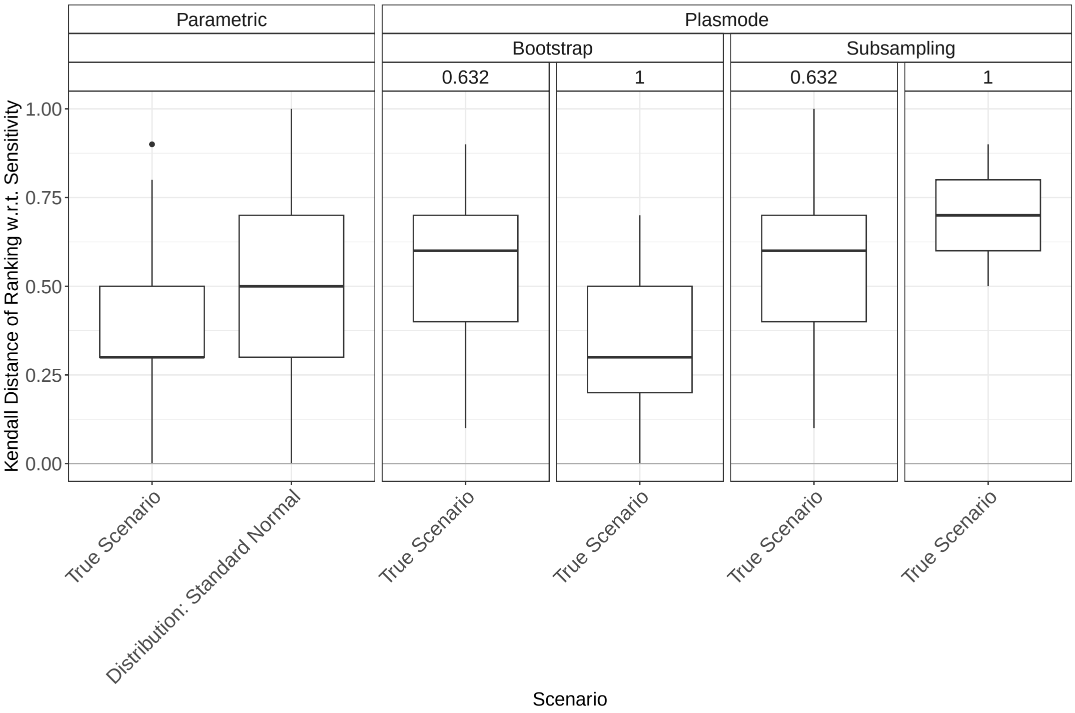

Fig H.105: Kendall distance of the simulated and true method ranking based on sensitivity in 100 iterations of a classification method comparison study per classifier for different simulation approaches with misspecification of the distribution as standard normal for parametric simulation for $p = 150$.

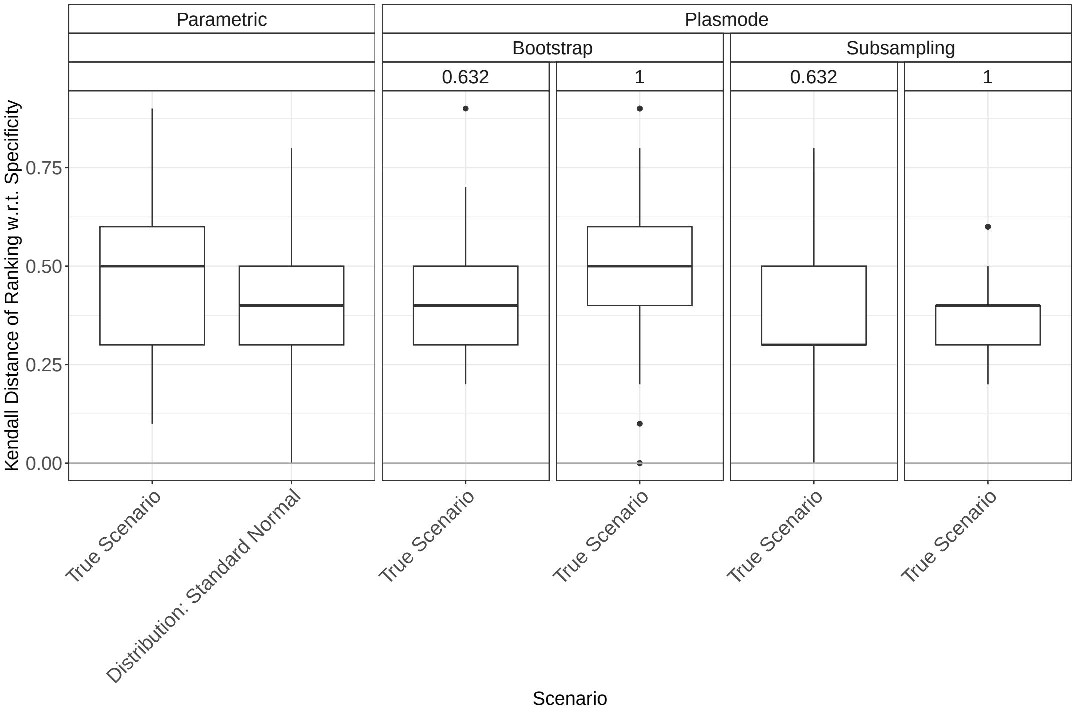

Fig H.106: Kendall distance of the simulated and true method ranking based on specificity in 100 iterations of a classification method comparison study per classifier for different simulation approaches with misspecification of the distribution as standard normal for parametric simulation for $p = 150$.
